# Evolution of the expansin superfamily in bryophytes and green algae

**DOI:** 10.1007/s00709-026-02200-2

**Published:** 2026-04-23

**Authors:** Maddisyn E. Behney, Tyani Orta, Robert E. Carey

**Affiliations:** 1 Program in Biochemistry & Molecular Biology, Lebanon Valley College, Annville, PA USA; 2 Biology Department, Lebanon Valley College, Annville, PA USA

**Keywords:** Expansin, Gene family evolution, Bryophytes, Green algae

## Abstract

Expansins are a superfamily of non-enzymatic proteins that mediate plant cell wall loosening and play essential roles in growth and development. The expansin superfamily consists of four families: EXPA, EXPB, EXLA, and EXLB. Although the expansin gene families have been well characterized in angiosperms, their evolutionary history in early-diverging land plants and green algae remains incompletely resolved. This study examines expansin superfamilies in two liverworts (*Marchantia polymorpha* and *Conocephalum conicum*), a hornwort (*Anthoceros agrestis*), a moss (*Ceratodon purpureus*), and two green algal species (*Chara braunii* and *Spirogloea muscicola*). Expansin genes were identified from these organisms using BLAST searches. These newly assembled gene families were then analyzed to determine the relationships between them and gain insight into early expansin evolution. No EXLA or EXLB genes were found in bryophytes or green algae. Bryophytes contain EXPA genes; however, EXPBs were not detected in liverworts and are present only in mosses and hornworts. Although green algal expansins share key characteristics of both EXPA and EXPB genes, evidence suggests they are not members of either family. The data presented here raises interesting questions about the timing of EXPA and EXPB evolution in land plants.

**Supplementary Information:**

The online version contains supplementary material available at 10.1007/s00709-026-02200-2.

## Introduction

Expansins are a superfamily of non-enzymatic proteins critical for pH-dependent plant cell wall extension (McQueen-Mason et al. 1992). Processes such as fruit ripening, seed germination, pollen tube growth, and general cell growth rely on expansin-mediated cell wall modification (Rose et al. 1997; Chen and Bradford 2000; Cho and Cosgrove 2000; Valdivia et al. 2007). Expansins are composed of approximately 270 amino acids, have a cleaved signal peptide, and a two-domain structure, which includes an N-terminal domain and a C-terminal domain (Cosgrove 2024). Within each domain, there are conserved amino acids and amino acid motifs that appear important for expansin function (Cosgrove et al. 2002).

The expansin superfamily consists of four families: EXPA, EXPB, EXLA, and EXLB (Sampedro and Cosgrove 2005). EXPAs and EXPBs have been experimentally demonstrated to have cell wall loosening activity (Cosgrove 1989; Li et al. 1993). EXLBs have an unknown function and are included in the superfamily based on sequence similarity (Sampedro and Cosgrove 2005). EXLAs, although previously included based only on sequence similarity, have recently been shown to promote cell wall extension under acidic conditions (Men et al. 2026). Previous analyses have indicated that the common ancestor of all angiosperms likely had a minimum of 12 EXPAs, 2 EXPBs, 1 EXLA and 2 EXLBs (Sampedro et al. 2005). These clades have been shown to be highly conserved amongst all angiosperms studied so far (Sampedro et al. 2006; Seader et al. 2016; Wang et al. 2024).

Although lycophytes have conserved EXPA and EXPB sequences, they lack EXLA and EXLB genes. The common ancestor of lycophytes and angiosperms is believed to have a minimum of 2 EXPAs and 1 EXPB (Carey et al. 2013).

The common ancestor of mosses and angiosperms appears to also have had a minimum of 2 EXPAs and 1 EXPB. Moss expansins are far more divergent when compared to either angiosperm or lycophyte expansins, indicating a more independent evolutionary history. Despite this divergence, moss expansins still share the structure and common motifs associated with expansins (Carey and Cosgrove 2007).

To further elucidate the history of expansins in early diverging land plant lineages, we have extended these analyses to two liverworts (*Marchantia polymorpha* and *Conocephalum conicum*), a hornwort (*Anthoceros agrestis*), and an additional moss (*Ceratodon purpureus*). We have also expanded this analysis to two green algae species (*Chara braunii* and *Spirogloea muscicola*). *C. braunii* and *S. muscicola* were chosen as representative organisms for multicellular and unicellular green algae, respectively. *S. muscicola* belongs to the Zygnematophyceae, and *C. braunii* belongs to the Charophyceae, both of which belong to the division Charophyta within Streptophyta. Charophyta are considered to be sister to land plants, and this phylogenetic position makes *C. braunii* and *S. muscicola* valuable models for expansin research and for investigating how the expansin gene family evolved as plants transitioned to terrestrial life (Ferranti and Delwiche 2024).

## Materials and methods

### Identifying expansin superfamilies

#### Marchantia (liverwort)

The *M. polymorpha* expansin superfamily was assembled from MpTak v5.1 of MarpolBase using tBLASTx with known *A. thaliana* expansin sequences as queries (Sampedro et al. 2005; Montgomery et al. 2020).

#### Conocephalum (liverwort)

The *C. conicum* expansin superfamily was assembled from genome assembly ASM5008447v1 in NCBI with NCBI tBLASTx using known *A. thaliana* expansin sequences as queries. (Sampedro et al. 2005; Sayers et al. 2025).

#### Ceratodon (moss)

The *C. purpureus* expansin superfamily was assembled from genome assembly CpurpureusR40_1_0 using Phytozome BLASTn with known *P. patens* and *A. thaliana* expansin sequences as queries (Sampedro et al. 2005; Carey and Cosgrove 2007; Goodstein et al. 2012; Carey et al. 2021). Additional sequences (CpEXPA22, CpEXPB4, CpEXPB5) were gathered from the genome with NCBI tBLASTx with known *P. patens* expansin sequences as queries (Carey and Cosgrove 2007; Sayers et al. 2025).

#### Anthoceros (hornwort)

The *A. agrestis* expansin superfamily was assembled from the [Bonn] genome assembly using *A. thaliana* as queries (Sampedro et al. 2005; Li et al. 2020.). Blast searches of the *A. agrestis* genome were performed locally using BLAST + 2.15 (Camacho et al. 2009).

#### Chara (green algae)

The *C. braunii* expansin superfamily was assembled from the *Chara braunii* S276 genome using PhycoCosm BLASTn with known *P. patens* expansin sequences as queries (Carey and Cosgrove 2007; Nishiyama et al. 2018; Grigoriev et al. 2021).

#### Spirogloea (green algae)

The *S. muscicola* expansin superfamily was assembled from the *Spirogloea_muscicola*_CCAC_0214 genome using PhycoCosm BLASTn with known *P. patens* expansin sequences as queries (Carey and Cosgrove 2007; Cheng et al. 2019; Grigoriev et al. 2021).

### Sequence alignments

The protein sequences encoded by the expansin superfamilies of *M. polymorpha*,* C. conicum*,* A. agrestis*,* C. purpureus*,* C. braunii*, and *S. muscicola* were aligned with selected angiosperms, lycophytes, and bryophytes. Three alignments were constructed for phylogenetic analysis and may be found in Supplementary File 1. The first alignment contained the EXPA sequences of *M. polymorpha*, *C. conicum*,* A. agrestis*, and *C. purpureus*, along with known *P. patens* EXPAs, *S. moellendorffii* EXPAs, and Arabidopsis-rice EXPA sequences representing EXPA clades I-XII (Carey and Cosgrove 2007; Carey et al. 2013; Sampedro et al. 2005). *P. patens* PpEXPA20 was excluded from the alignment due to a long C-terminal extension that degrades the alignment. *C. braunii* EXPs 8, 10, and 11 and PpEXPB1 were also included in the alignment to root the resulting tree. The second alignment included EXPB sequences of *A. agrestis* and *C. purpureus*, along with known *P. patens* EXPBs, *S. moellendorffii* EXPBs, and representative Arabidopsis-rice sequences of EXPB, EXLA, and EXLB clades (Sampedro et al. 2005; Carey and Cosgrove 2007; Carey et al. 2013). Included in the alignment were *C. braunii* EXPs 8, 10, and 11 and PpEXPA1 to help root and resolve the tree. The third alignment constructed contained representative bryophyte, lycophyte, and Arabidopsis-rice EXPA and EXPB genes of along with expansin sequences from *C. braunii* and *S. muscicola* (Sampedro et al. 2005; Carey and Cosgrove 2007; Carey et al. 2013). Such an alignment was necessary to analyze the relationship between algae expansins and terrestrial plant expansins. Alignments were created using the default parameters of the MUSCLE algorithm within the Unipro UGENE software package (Okonechnikov et al. 2012). Alignments were then trimmed at a conserved tryptophan upstream of the expansin HATFYG motif and a conserved phenylalanine near the C-terminus.

### Phylogenetic analysis

Phylogenetic trees were constructed using MrBayes v3.2.5 (Ronquist et al. 2012). Priors for the Bayesian trees were as follows: Jones amino acid model, gamma estimation, 7,000,000 generations, two runs of five Markov chains each – burnin as indicated in figure legends. The consensus trees were visualized using Figtree, where the bryophyte EXPA tree was manually rooted at *C. braunii* sequences CbEXP8, 10, and 11, and the EXPB tree was manually rooted at the *C. braunii* sequences CbEXP8, 10, and 11 (Rambaut 2018). Although CbEXP8, 10, and 11 may not be the true root for either the EXPA or EXPB tree, such *C. braunii* sequences were chosen to facilitate comparison between the two trees. The algae EXP tree was left unrooted. Bayesian phylogenetic analysis was used to be consistent with previous studies of expansin evolution. (Sampedro et al. 2005; Carey and Cosgrove 2007; Carey et al. 2013; Seader et al. 2016)

### Poisson corrected distances

For analysis of algal expansins, an alignment of algae EXPs, bryophyte EXPAs and EXPBs, lycophyte EXPAs and EXPBs, and angiosperm EXPAs and EXPBs was created using the MUSCLE algorithm within Unipro UGENE and was trimmed as was done for phylogenetic tree construction (Okonechnikov et al. 2012). Poisson-corrected (PC) amino acid distances were calculated using MEGA11 with standard error calculated for these values using 500 bootstrap replicates (Tamura et al. 2021). Gaps were handled with complete deletion. The alignment used for PC distance analysis may be found in Supplementary File 1.

### Intron patterns

When possible, genomic sequences were compared to their corresponding cDNA sequences to determine intron patterns. If only genomic sequences were available, translated sequences were aligned with related translated cDNA sequences, and intron locations were estimated based on gaps within the alignment along with previous knowledge of expansin intron patterns.

### Sequence logo

Amino acid sequences for algae EXPs and terrestrial plant EXPAs and EXPBs were aligned using the default parameters of the MUSCLE algorithm within the Unipro UGENE software package (Okonechnikov et al. 2012). The alignment was trimmed as was done for phylogenetic tree construction. The alignment was then sub-divided into algae EXL and terrestrial plant EXPA and EXPB portions. The sequence logo was generated using WebLogo (Crooks et al. 2004). The alignment used for sequence logo analysis may be found in Supplementary File 1.

## Results

### The expansin superfamilies of bryophytes and algae

As is the case with all terrestrial plants studied thus far, the EXPA families within the bryophytes studied here are larger than the EXPB families (Cosgrove 2024). The lone exception to this is *A. agrestis*, which has more EXPBs than EXPAs. The EXPA families of *M. polymorpha* and *C. conicum* appear to be slightly expanded, with *M. polymorpha* having 38 EXPAs and *C. conicum* having 32. Interestingly, no EXPBs were found within either liverwort, despite being found in all mosses and hornworts studied so far (Carey and Cosgrove 2007). Both *C. braunii* and *S. muscicola* contained expansins; however, such expansins were determined to not belong to any of the four expansin families found in terrestrial plants. Such expansins have been labeled as “EXPs”, as they are expansins but do not share all of the same features of the terrestrial plant expansin families (Table 1).

**Table 1:** Size and composition of expansin gene families of selected plant species * At time of search (Sampedro et al. 2005) ^Δ^ At time of search (Carey et al. 2013) ^†^ At time of search (Carey and Cosgrove 2007)

	EXPA	EXPB	EXLA	EXLB	Total EXP
A. thaliana*	26	6	3	1	36
O. sativa*	33	18	4	1	56
*S. moellendorffii*^Δ^	15	2	0	0	17
*P. patens†*	28	7	0	0	35
*C. purpureus*	22	6	0	0	28
*A. agrestis*	7	8	0	0	15
*M. polymorpha*	38	0	0	0	38
*C. conicum*	32	0	0	0	32
*C. braunii*	0	0	0	0	22
*S. muscicola*	0	0	0	0	12

In agreement with previous studies of bryophytes and algae, none of the selected species contained EXLA or EXLB genes (Carey and Cosgrove 2007; Vannerum et al. 2011; Wang et al. 2024). Based on these results and findings from studies of seedless vascular plants, it is likely that the EXLA and EXLB families emerged after the divergence of pteridophytes (Carey et al. 2013).

In addition to complete sequences, possible pseudogenes were found in *C. conicum*. CcEXPA3 contains a frameshift near the expansin “VPC” motif, and CcEXPA23 contains a frameshift before the “TATN” expansin motif. Although they are possible pseudogenes, both expansins do not contain premature stop codons or other sequence degradation, and therefore still were included as expansins. CcEXPA3 sequence was adjusted by removing two bp fifteen amino acids downstream of the motif “VPC”. CcEXPA23 also had two bp removed from its sequence before the “TATN” expansin motif. Such adjustments were made to ensure the entire expansin coding sequence was in frame for alignment. Accession numbers may be found in Supplementary File 2.

### Phylogenetic analysis of bryophyte expansins

#### EXPA

**Fig. 1:**
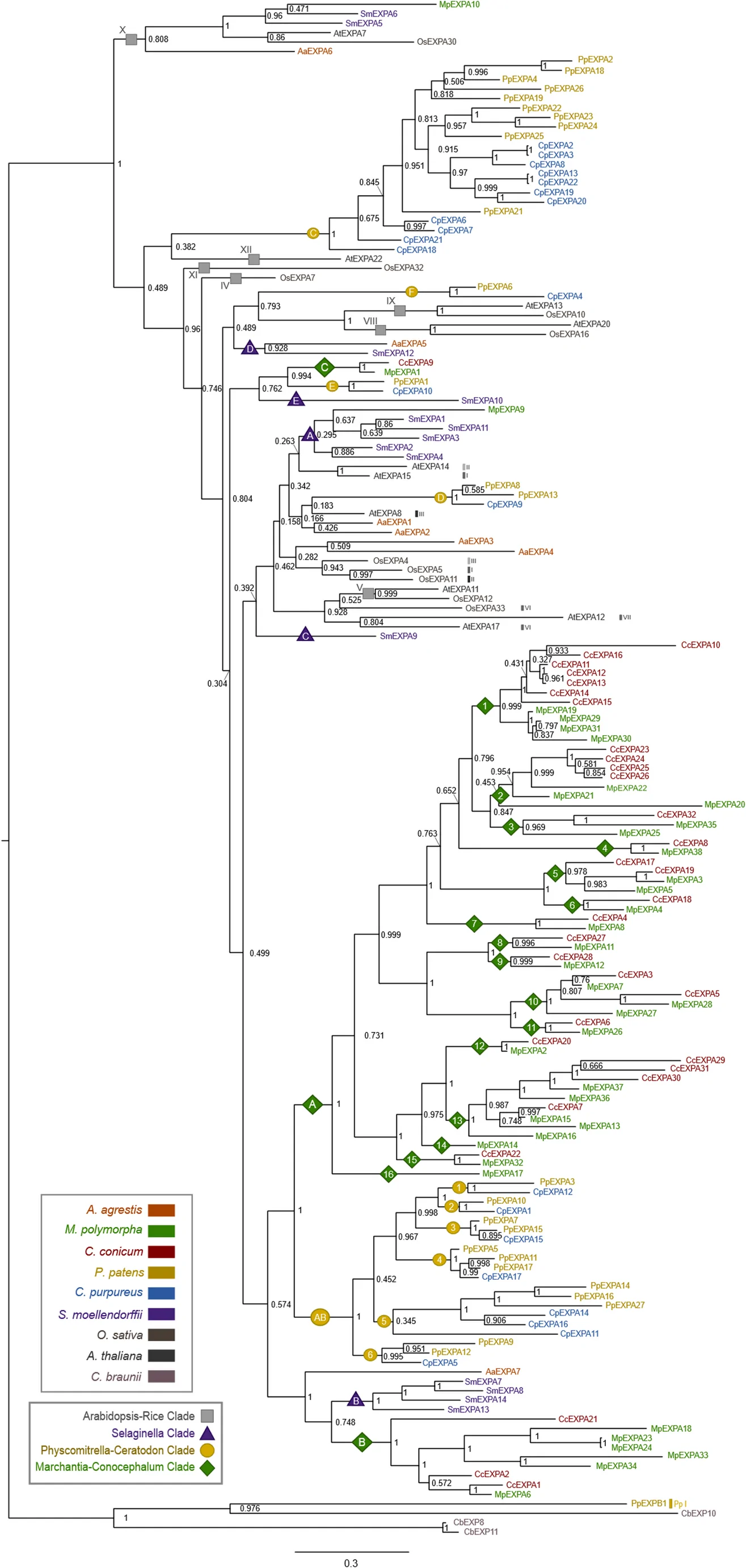
Bayesian tree for the *M. polymorpha* (liverwort), *C. conicum* (liverwort), *A. agrestis* (hornwort) and *C. purpureus* (moss) EXPA families with selected *A. thaliana* (angiosperm), *O. sativa* (angiosperm), *C. braunii* (green algae), *S. moellendorffii* (lycophyte), and *P. patens* (moss) sequences. Bryophyte and lycophyte EXPA clades are designated with Latin letters. Angiosperm EXPA clades are designated with Roman numerals. The tree was constructed from 7,000,000 generations and the burnin was set to 25%. The tree was manually rooted at the *C. braunii* sequences CbEXP8, 11 and 10

Most liverwort and moss EXPAs appear to largely group separately from the angiosperm-specific EXPAs. Following established nomenclature, bryophyte expansin clades are designated with Latin letters to distinguish them from spermatophyte clades, which use Roman numerals (Sampedro et al. 2005; Carey and Cosgrove 2007; Carey et al. 2013). A previous study of *P. patens* EXPAs divided the family into six clades named A-F (Carey and Cosgrove 2007). The addition of *C. purpureus* EXPA genes has further refined the structure of the moss EXPA family. *P. patens* clades A and B can be merged into one clade now named “AB”. This clade AB contains 6 subclades labeled AB1-AB6. For the liverworts, there appear to be three liverwort-specific clades, now called A, B, and C. Clade A can be divided into 16 subclades labeled A1-A16. MpEXPA14 is grouped with liverwort subclades A12 and A13 but does not appear to belong to either clade, therefore it has been given the clade designation A14. Liverwort clade B is sister to *S. moellendorffii* clade B. Liverwort clade C is sister to *P. patens* clade E, which appears to be a moss-liverwort specific clade consisting of MpEXPA1, CcEXPA9, PpEXPA1, and CpEXPA10 (Fig. 1).

A few bryophyte EXPAs group with known Angiosperm clades. *S. moellendorffii* EXPAs 5 and 6 along with MpEXPA10 and AaEXPA6 belong to Arabidopsis-rice clade X. Previously described *P. patens* clade F, containing PpEXPA6 and CpEXPA4, branches with Arabidopsis-rice clades VIII and IX. (Fig. 1).

As noted previously, most *A. agrestis* EXPAs group with vascular plant EXPAs, although with poor support. The only exception is AaEXPA7, which branches with liverwort clade B and *S. moellendorffii* clade B Overall, *A. agrestis* EXPAs do not group strongly with bryophyte-specific EXPA lineages. It is also important to note that the *A. agrestis* genome is one of the smallest among land plants, and this may contribute to the reduction of bryophyte-specific EXPAs (Li et al. 2020). Previous studies have shown a reduction in expansin family size due to adaptation to an aquatic lifestyle (Hepler et al. 2020). Gene members of clade X, for example, are lost in most aquatic angiosperm species, but are present in *A. agrestis* and *M. polymorpha*, suggesting that any lost clades in *A. agrestis* are likely bryophyte-specific.

#### EXPB

**Fig. 2:**
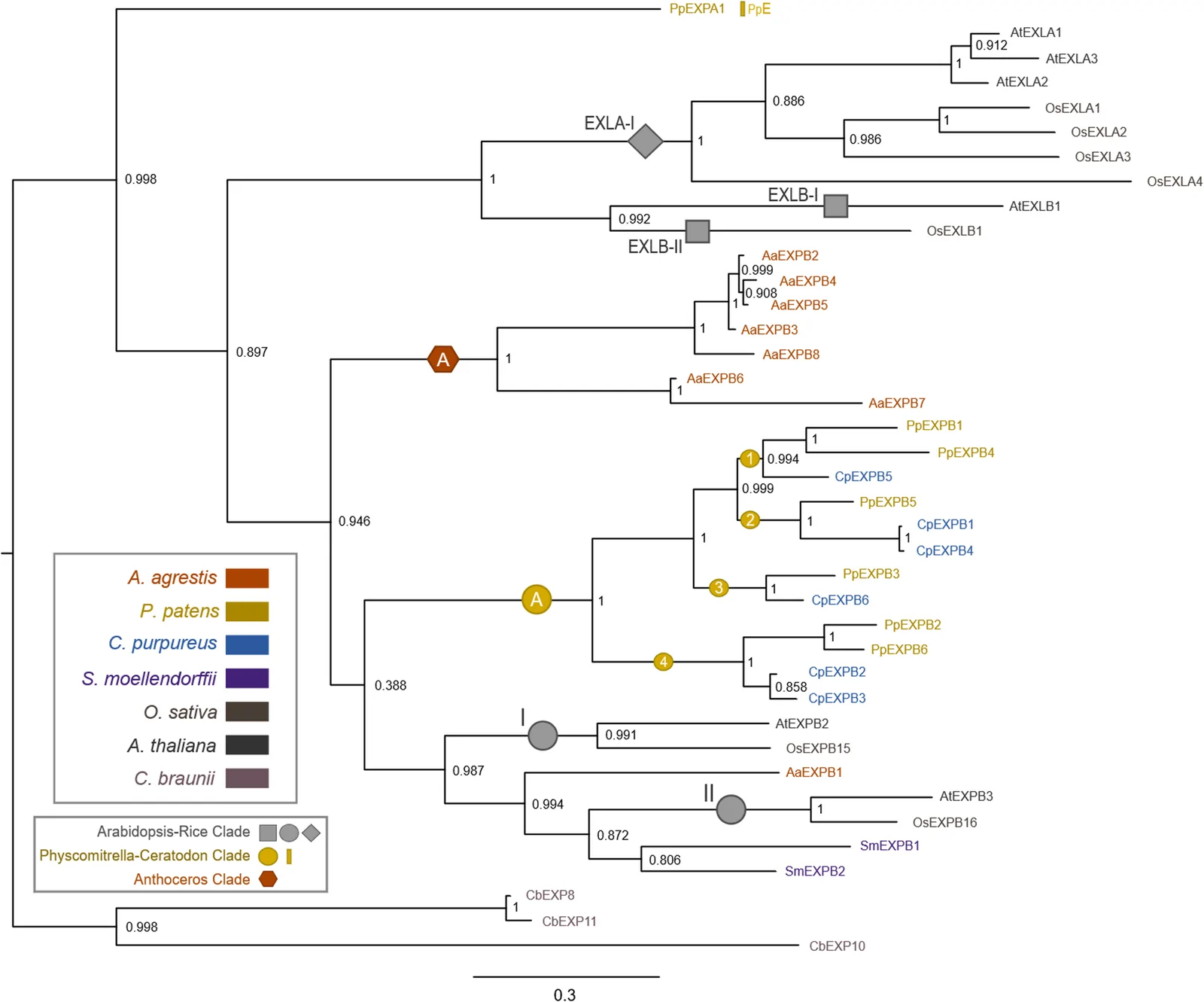
Bayesian tree for the *A. agrestis* (hornwort) and *C. purpureus* (liverwort) EXPB families with selected *A. thaliana* (angiosperm), *O. sativa* (angiosperm), *C. braunii* (green algae), *S. moellendorffii* (lycophyte), and *P. patens* (moss) sequences. Grey circles represent Arabidopsis-rice EXPB clades, grey squares represent Arabidopsis-rice EXLB clades, and a grey diamond represents Arabidopsis-rice EXLA clade I. Bryophyte EXPB clades are designated with Latin letters. Angiosperm EXPB, EXLA, and EXLB clades are designated with Roman numerals. The tree was constructed from 7,000,000 generations and the burnin was set to 25%. The tree was manually rooted at the *C. braunii* sequences CbEXP8, 11 and 10

AaEXPBs 2 through 8 group together forming an *A. agrestis*-specific clade, named clade A (Fig. 2). This clade is sister to all bryophyte and angiosperm EXPB clades (bpp = 0.946). AaEXPB1 groups sister to Arabidopsis-rice clade II (bpp = 0.994).

The inclusion of *C. purpureus* EXPB genes revealed internal structure of the EXPB family within mosses, with all six genes grouping with *P. patens*. Such grouping suggests a single moss-specific EXPB clade containing four subclades, labeled clade A. Each subclade contained at least one *P. patens* and *C. purpureus*, and have been named as 1, 2, 3, and 4 (Fig. 2).

### Phylogenetic analysis of algae expansins

**Fig. 3:**
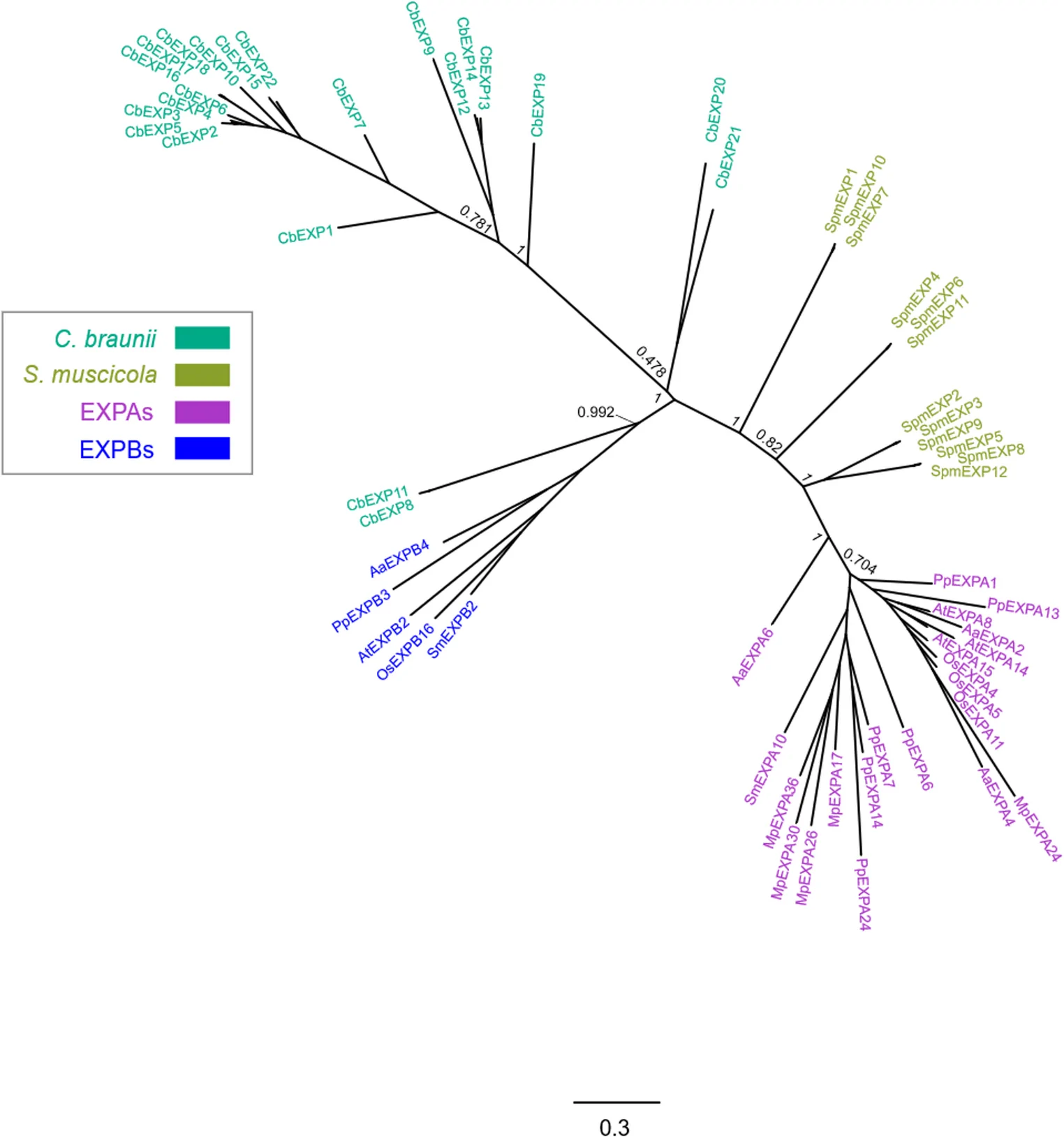
Unrooted Bayesian tree for the *C. braunii* (green algae) and *S. muscicola* (green algae) EXP families with selected bryophyte, lycophyte, and angiosperm EXPA and EXPB sequences. The tree was constructed from 7,000,000 generations and the burnin was set to 25%. Confidence levels are Bayesian posterior probabilities

Phylogenetic analysis of the algae EXPs was performed to aid in the understanding of the algae EXPs’ relationship to terrestrial plant EXPAs and EXPBs. Both *S. muscicola* and most *C. braunii* EXPs group separately from both EXPA and EXPB clades (Fig. 3). Although CbEXPs 8 and 11 group with the terrestrial plant EXPBs, sequence analysis suggests that they are not members of the EXPB family.

### Distances of algae expansins to terrestrial plant expansins and sequence logo analysis

**Fig. 4:**
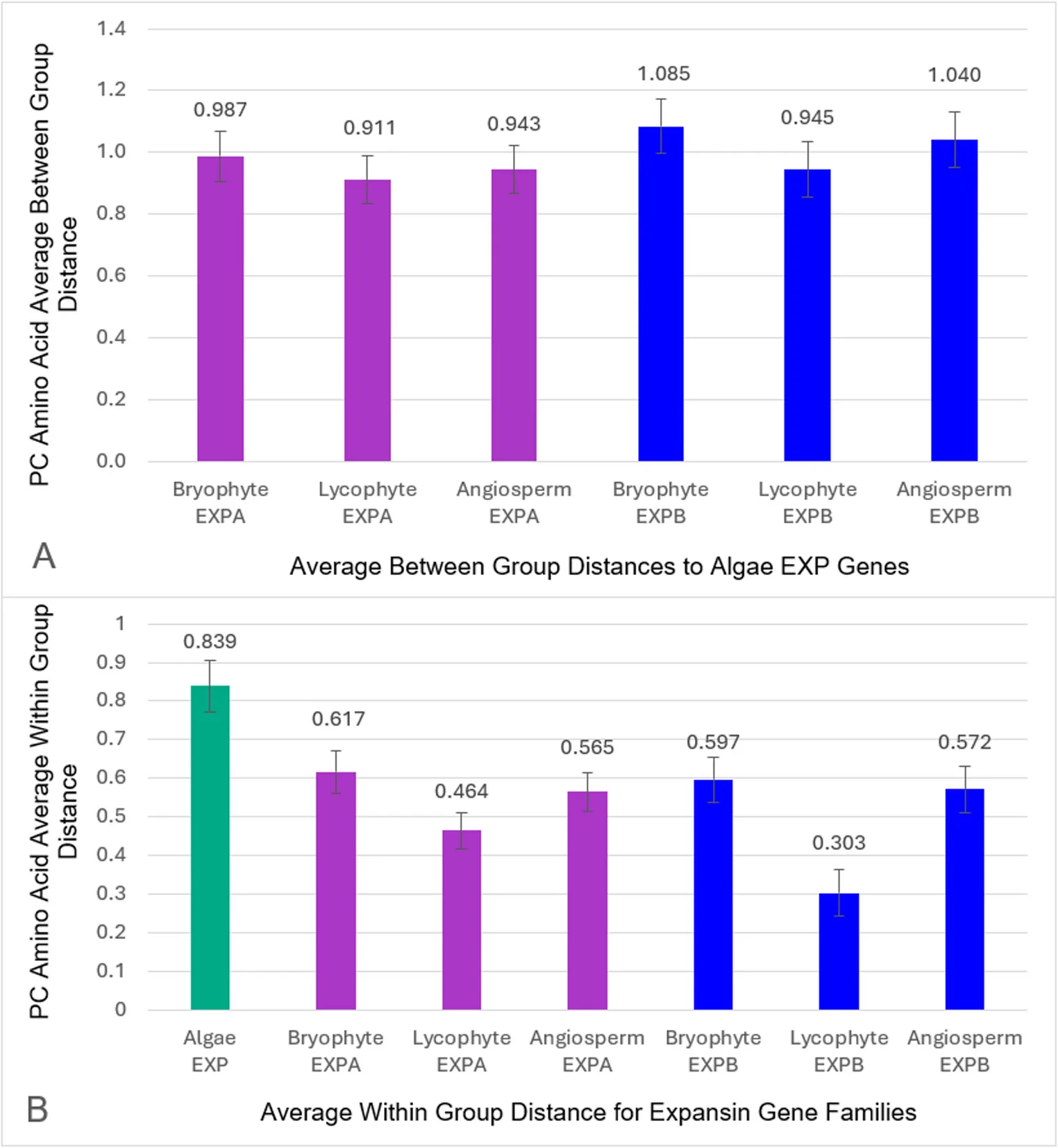
(**A**) Between-group average distances of algae EXP genes to terrestrial plant EXPA and EXPB genes. Values indicated are the average Poisson-corrected amino acid distance to the algae EXP family. Error bars are standard errors based on 500 bootstrap replicates. (**B**) Within-group average distances for algae EXPs and terrestrial plant EXPAs and EXPBs. Values indicated are the average within group Poisson-corrected amino acid distances. Error bars are standard errors based on 500 bootstrap replicates

Utilizing MEGA11, Poisson-corrected (PC) amino acid distances were calculated to better understand the relationship of algae EXPs to terrestrial plant EXPAs and EXPBs. Amino acid distances are pairwise calculations of the degree of difference between two sequences, with Poisson correction distance accounting for the rare occurrence of amino acids changing and then reverting to their original form over time. Algae EXPs have much higher within-group PC distances compared to the terrestrial plant expansins, possibly indicating greater genetic variation among algae expansins (Fig. 4).

**Fig. 5:**
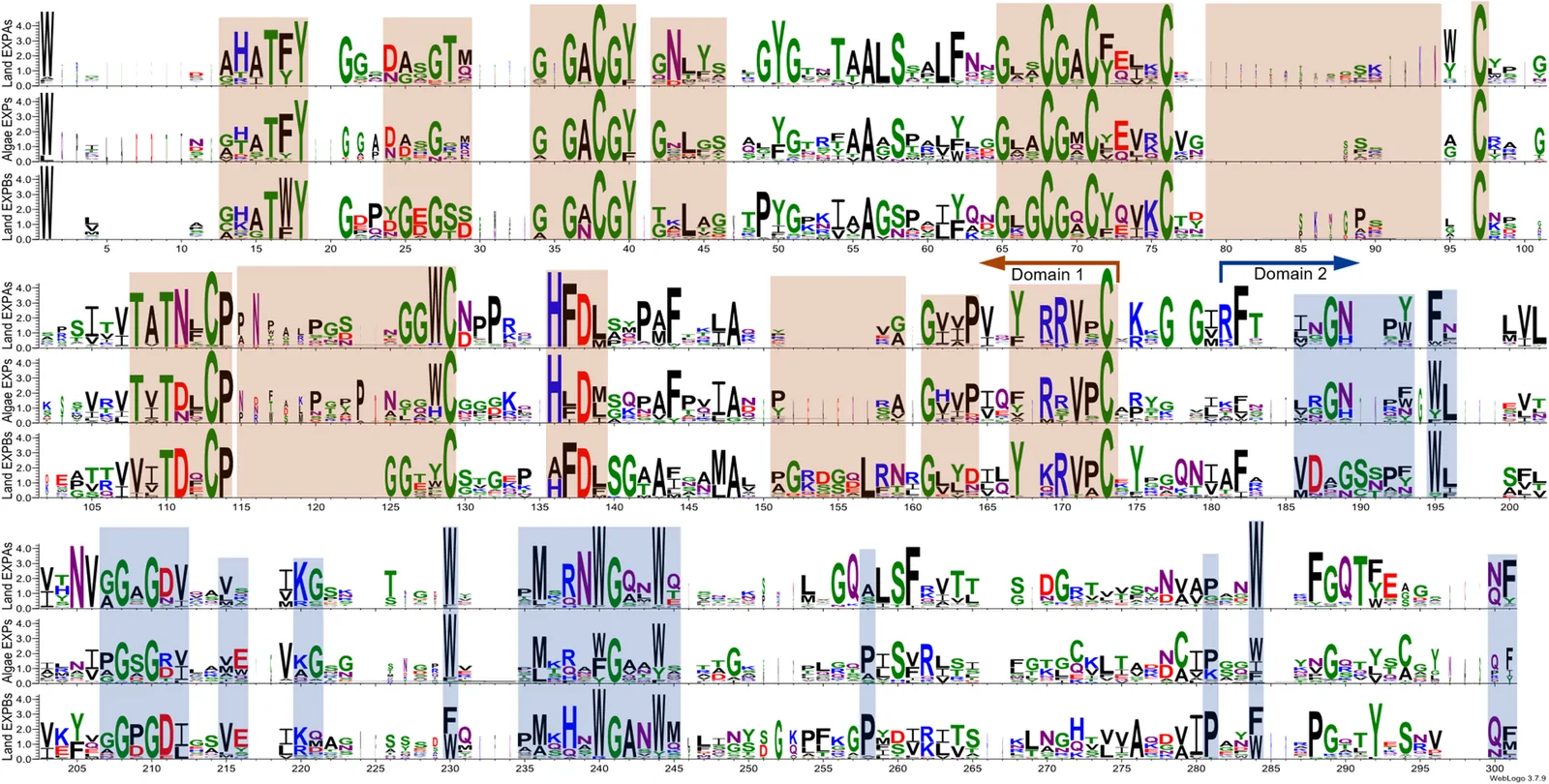
SeqLogo of the algae EXP, land plant EXPA, and land plant EXPB families. Each row draws comparisons for each family: the top lines are land plant EXPA sequences, the middle lines are algae EXP sequences, and the bottom lines are land plant EXPB sequences. The height of the letters indicates the frequency of the amino acid in that group of sequences. Highlighted residues represent conserved motifs or indels of the expansin families. Those highlighted in orange are within domain 1 of the expansin protein, and those highlighted in blue are within domain 2

Average between-group distances were calculated for algae EXPs to bryophyte, lycophyte, and angiosperm EXPA and EXPB families (Fig. 4). Algae EXPs share similar distances to all terrestrial plant EXPA and EXPB families, suggesting that they are not members of either expansin family. This is further supported by sequence logo analysis of algae EXPs and terrestrial plant EXPAs and EXPBs. The highlighted range of amino acid residues 24–29 shows algae EXPs share a similar motif to ‘DASGTM’ found only in EXPAs. Some algae EXPs also contain an insertion located at amino acid residues 116 to 129 similarly found in EXPAs. However, some algae EXPs have indels more similar to EXPBs at residue ranges 79–94, and 151–159. Algae EXP residues that are more similar to EXPAs include residue numbers 17 (F), 136 (H), 230 (W), 284 (W). Algae EXP residues more similar to EXPBs include residue numbers 195 (W) and 258 (P) (Fig. 5). Such analysis, along with the phylogenetic and PC-distance data for algae EXPs, suggests that the origin of these EXP genes predates the EXPA-EXPB split, and that they are not members of either family.

### Bryophyte and algae intron patterns

Along with having conserved amino acid sequences, past studies have shown that expansins have conserved intron patterns (Sampedro et al. 2005). It is hypothesized that the ancestral intron pattern for EXPAs consisted of introns A and B, while the likely ancestral pattern for EXPBs consisted of introns A, C, B, and F (Sampedro et al. 2005). The intron patterns found in *M. polymorpha*, *A. agrestis*, *C. purpureus*, and *C. conicum* support the hypothesized ancestral EXPA and EXPB patterns. Details on intron patterns in each species can be found in Supplementary File 3.

Unusual intron patterns were found in CpEXPB6 and CcEXPA15. CpEXPB6 contains introns A, C, and F along with two novel introns. One novel intron is found between intron C and where B is typically found, with the other novel intron being located after intron F. These novel introns have also been found in PpEXPB3, suggesting that PpEXPB3 and CpEXPB6 are orthologs (Carey and Cosgrove 2007). CcEXPA15 has an intron located where intron F is typically found, which may indicate intron F is ancestral to the EXPA family but has been lost in most lineages.

In an attempt to uncover the origins of expansin intron patterns, a survey of algae expansin intron patterns was undertaken. *C. braunii* EXPs 8 and 21 contain introns A and B, and CbEXP21 contains just intron B. CbEXP19 contains a novel intron around fifteen amino acids downstream from where intron F is found. Additionally, a novel intron located two amino acids before the highly conserved ‘HFD’ motif was found in 14 of the *C. braunii* expansin sequences. Such intron averages 1500 bp in length, and we have designated this intron as intron ‘H.’ Six *S. muscicola* expansin genes contain introns B and F, with half of those six also containing intron A and the other half containing intron C. Details on intron patterns of *C. braunii* and *S. muscicola* can be found in Supplementary File 3. Because *S. muscicola* expansin genes contain introns A, B, C, and F, this may indicate that all four of these introns are ancestral to EXPAs and EXPBs.

## Discussion

### Gene family compositions of bryophytes

**Fig. 6:**
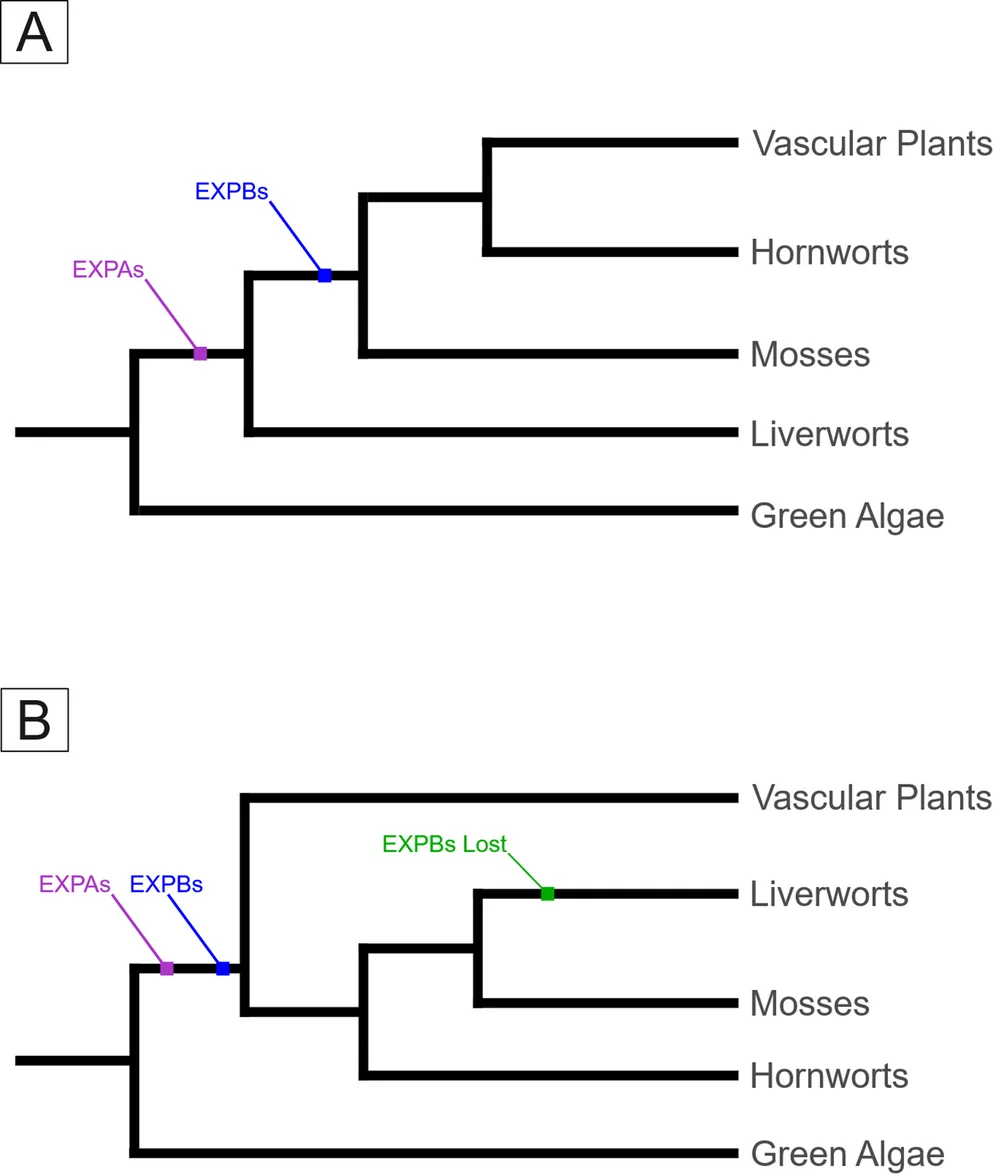
Species phylogenetic trees showing the two possible arrangements of the three bryophyte lineages and vascular plants, with green algae as an outgroup. (**A**) Shows the bryophytes as a paraphyletic group, with liverworts diverging first, followed by mosses then hornworts. (**B**) Shows bryophytes as a monophyletic group, with liverworts and mosses sharing a more recent common ancestor than with hornworts. Hypothetical origins of EXPAs and EXPBs are indicated

The lack of EXPB genes in *M. polymorpha* and *C. conicum* suggests liverworts lack the EXPB gene family. The timing of the origin of EXPBs, given this absence, depends on the evolutionary relationship between bryophytes; however, there are multiple competing hypotheses for the evolutionary relationships among bryophytes (Qiu and Mishler 2024). Of those competing hypotheses, the two with the most support suggest that bryophytes are either a paraphyletic group with hornworts sister to vascular plants or a monophyletic group sister to vascular plants (Liu et al. 2014; Gitzendanner et al. 2018). If bryophytes are monophyletic, EXPBs are ancestral to all bryophyte lineages but have been lost in liverworts. If bryophytes are a paraphyletic group, EXPBs arose in the common ancestor of mosses, hornworts, and vascular plants, postdating the divergence of liverworts (Fig. 6). Because the exact phylogenetic relationships between bryophyte lineages remain unclear, favoring one of the hypotheses for the lack of EXPBs within *M. polymorpha* is challenging.

### Ancestral genes

The internal structure within liverwort gene family suggests that there are at least 19 EXPA genes in the common ancestor of liverworts. Analysis of the mosses reveals a minimum of 10 ancestral EXPA genes and four ancestral EXPB genes. Based on *A. agrestis’s* structure, there are at least five ancestral EXPA genes and two ancestral EXPB genes.

When considering all bryophytes and their internal structure within the EXPA phylogenetic tree, there appears to be a minimum of three ancestral EXPA genes in the common ancestor of all bryophytes. These three ancestral lineages are clade X, the clade containing AaEXPA7 as well as *S. moellendorffii* and liverwort clades B, and the clade containing liverwort clade A and moss clade AB. Three ancestral EXPAs in bryophytes is likely an underestimate and this number will probably increase as more sequences are analyzed. Given the uncertainty in the evolutionary relationships between bryophytes, determining the number of EXPB ancestral genes in the common ancestor is challenging. If their relationship is paraphyletic, then the ancestor of all bryophytes had zero EXPB genes. However, if bryophytes are monophyletic, the common ancestor would have two EXPB genes. As for the common ancestor of mosses and hornworts, if their relationship is paraphyletic, their common ancestor likely had 1 EXPB gene. If they are monophyletic, it seems more likely that the ancestor had 2 EXPB genes.

### Algae phylogenetics, distances, & sequence logo

The algae EXP genes contain all the characteristics common to expansins, including a cleaved signal peptide at the amino terminus, a series of cysteine residues within patterned motifs of domain 1, an “HFD” motif, and a series of aromatic and tryptophan residues within patterned motifs of domain 2 (Cosgrove et al. 2002; Kende et al. 2004). However, despite being expansins, they do not clearly meet the criteria to be characterized as either EXPAs or EXPBs based on sequence logo and PC distance analyses (Figs. 4 and 5).

Phylogenetic analysis reveals that both *C. braunii* and *S. muscicola* branch separately from EXPA and EXPB families, with the exception of CbEXPs 8 and 11, which branch at the base of the EXPB family with good support (bpp = 0.992, Fig. 3). CbEXPs 8 and 11 share an insertion typically found within EXPBs at residues 151–159; however, the insertion found in the CbEXPs has a divergent sequence compared to EXPBs. This, together with their location in the phylogenetic tree, may suggest that they are evolving characteristics typical of EXPBs, but still lack a complete set of characteristics associated with the EXPB family, such as the conserved insertion in the C-terminal domain at residues 251–253 and the conserved tryptophan at residue 195.

The average PC distance between algae EXPs and land plant EXPAs is not much less than the average distance between algae EXPs and land plant EXPBs. The PC distance data seem to confirm what was observed in the phylogenetic and sequence analyses, that algae EXPs are neither EXPAs nor EXPBs.

## Conclusion

The absence of EXPBs in *M. polymorpha* and *C. conicum* suggests that the EXPB gene family is absent in all liverworts. The most parsimonious explanation of this observation suggests that the origin of the EXPB family postdates the divergence of liverworts from other bryophytes. Investigation of green algae expansins reveals a diverse gene family that, although it shares the common characteristics of expansins, lacks a complete set of characteristic features from either EXPAs or EXPBs. The exact timing of the origination of EXPAs remains unclear. It is possible that the early stages of EXPA and EXPB divergence are already occurring in green algae, but there is no evidence of true EXPAs or EXPBs outside of land plants.

The functional selection pressure that is leading to the dynamic nature of the expansin gene superfamily is mostly unclear. In the grasses there has been a large expansion of the EXPB family (Sampedro and Cosgrove 2005). This expansion may be a result of the greater efficacy that EXPB proteins have in causing extension in the type II cell walls of grasses (Li et al. 2003; Sampedro et al. 2015). The differences between type I and type II walls in terms of their hemicelluloses may be responsible for this difference. EXLA and EXLB genes, while found in all gymnosperms and angiosperms, are absent in lycophytes, bryophytes, and green algae (Carey and Cosgrove 2007; Vannerum et al. 2011; Wang et al. 2024). A recent study has shown that EXLA proteins interact with pectin and are able to cause wall extension (Men et al. 2026). It is possible that a change in cell wall composition is responsible for the appearance of the EXL families, but there is currently no evidence to support this. Similarly, cell wall composition may underlie expansin family differences in bryophytes. Studies of *P. patens* suggest its cell wall composition is similar to other plants based on cell wall biosynthesis genes (Ye and Zhong 2022). Even less is known about the cell walls of liverworts than mosses, making hypotheses about the cause of EXPB family absence difficult to formulate. Without more detail on how bryophyte and algae cell walls differ from those of other plant lineages, it is difficult to determine the biochemical changes potentially driving the expansin gene evolution described in this study.

To improve this analysis and better resolve the evolutionary history of early land plant expansins, a broader range of genomes should be analyzed, including more green algae, bryophyte, and pteridophyte species. Such data will likely refine the conclusions presented here, while investigations of gene collinearity within bryophytes could further clarify early phylogenies. Additionally, including gymnosperm species would provide a critical link between early land plants and angiosperm expansins.

## Supplementary information

Below is the link to the electronic supplementary material.

Supplementary File 1 (PDF 449 KB) Contains the alignments created for phylogenetic, PC distance, and sequence logo analyses.

Supplementary File 2 (XLSX 22.5 KB) Provides the accession numbers and ranges or chromosome coordinates for each expansin.

Supplementary File 3 (XLSX 20.3 KB) Contains information on the intron patterns found within each expansin.

## Data Availability

Sources of all data and gene sequences are given in the methods section of the manuscript. All accession numbers and other information critical to retrieving the data are given in Supplementary File 2.
