# Supplementary material for: Evolution of the expansin superfamily in bryophytes and green algae
Source: Protoplasma. 2026 Apr 23;263(5):1525–36. doi: 10.1007/s00709-026-02200-2 (PMC13493406; doi:10.1007/s00709-026-02200-2)
Supplement: Supplementary file 1 [file 709_2026_2200_MOESM1_ESM.pdf]

EXPA Tree

>CcEXPA1 [A]

W--SQ----AHATFYGGSD--ASGTN----N-GACGYS-NVFALGYGTMTAALS--------APLFNDGLACGACFQIQC

TGDP----------ACYS---NSIVVTATNLCPQ------GS--NGGWC-DYPKAHFDLSQPAFSQFA-------QTIA-

GHVTVNYQRVNCN-RRGGVHFKI----DGHTY-FMQVLLYNVGGPGDVQSVQVK-GTNT-G-WI--GMTRGWGQNWQ-TG

QV---LDGQSLSFAVKTS-DGRTLYHNNVADSNWRYGQTFE-G---SQF

>CcEXPA10 [AB]

W--TD----SHATFYGSQD--GQGST----G-AACGYGDNLEKMGYGMENTALS--------TTLFNQGLTCGACFEIKC

RVEE--------SRWCYP-DAGSVKVTATNFCPP-NANRPID----GWC-NPPRTHFDMSVPMFTKLA-------QYVG-

GIIPIKFRRIPCV-KQGGIRFVM----NGNPW-FNLVLVYNVAGDGNVMKMEIK-GTNT-A-WL--SMKANWGQNWE-LH

RI---LRGQALSFRVTDQ-SGKVIEAKDVVPSDWEFTQTFE-AT--TNF

>CcEXPA11 [AB]

W--TD----SHATFYGGND--GSGTM----G-GACGYG-NLHARGYGLQTAALS--------STLFNKGLTCGACFEIKC

KVKD--------TRWCYP-NAGSIKVTATNLCPA-NPNRPTN--NGGWC-NPPRTHFDLAVPMFTKLA-------KYVG-

GIIPVQFRRIPCV-KTGGIRFVL----NGNPW-FNLVLVYNAAGGGNVVNMQMK-GSKT-T-WY--IMRQNWGQNWE-LK

QK---LQGQALSFRVTTG-NGKVLVATNVAPANWKFGQTFE-DD--KNF

>CcEXPA12 [AB]

W--TD----SHATFYGGND--GSGTM----G-GACGYG-NLHSRGYGLQTAALS--------STLFNKGLTCGACFEIKC

KVKD--------TRWCYP-NAGSIKVTATNLCPA-NPNRPTN--NGGWC-NPPRTHFDLAVPMFTTLA-------KYVG-

GIIPVQFRRIPCV-KAGGIRFVL----NGNPW-FNLVLVYNVAGGGNVLNMQMK-GSKT-T-WY--TMRQNWGQNWE-LK

QK---LQGQALSFRVTAG-NGKVLVATNVAPANWKFGQTFE-DD--KNF

>CcEXPA13 [AB]

W--TD----SHATFYGGND--GSGTM----G-GACGYG-NLHSRGYGLQTAALS--------
STLFNKGLTCGACFEIKC

KVKD--------TRWCYP-NAGSIKVTATNLCPA-NPNRPTN--NGGWC-NPPRTHFDLAVPMFTTLA-------
KYVG-

GIIPVQFRRIPCV-KAGGIRFVL----NGNPW-FNLVLVYNVAGGGNVVNMQMK-GSKT-T-WY--
TMRQNWGQNWE-LK

QK---LQGQALSFRVTTG-NGKVLVATNVAPANWKFGQIFE-DD--KNF

>CcEXPA14 [AB]

W--TD----SHATFYGGND--GSGTM----G-GACGYG-NLHSRGYGLESAALS--------
STLFNKGLTCGACFEIQC

KVKD--------TKWCYP-NAGSIKVTATNMCPA-NPARPTN--NGGWC-NPPRTHFDLSVPMFTKLA------
-KYVG-

GIIPVQFRRIPCL-KAGGIRFVI----NGNPW-FNLVLVYNVAGGGNVVTMQME-GTKT-A-WY--
TMRQNWGQNWE-LK

QK---LQGQAISFRVTTG-NGKVLVASNVAPANWKFGQTFE-DD--KNF

>CcEXPA15 [ABF]

W--TD----SHATFYGGND--GSETM----G-GACEYG-NLHSRGYGLETAALS--------
STLFNKGLTCGACFEILS

KVKD--------SRWCYP-NAGSIKVTATNLCPT-NPARSTN--NGGWC-NPPRIHFDLSEPMFTKLA-------
KYVG-

GIIPVQFRRIPRV-KAAGIRFVM----NGNPW-FNLALVYNVAKA-NVVNMQMK------G-------
SRNWGQNPE-LK

EK---LQGRSLSFRVTTG-NGKVLVVNNVALANWKFGQTFE-D---DNL

>CcEXPA16 [AB]

W--TN----SHATFYGGND--GSGTM----G-GACGFG-NLNLRGYGLETTALS--------
STLFNKGLTCGACFEIQC

RVNE--------TRWCYP-NAGSIKITATNLCPA-NPSRPTN--NGGWC-NPPRTHFDLSVPMFTKLA-------
KYVG-

GIIPVQFRRIPCV-KTGGIRFVL----NGNPW-FNLVLVYNVAGDGNVEAMQMK-GNNT-S-WF--
TMTRNWGQNWE-LK

QK---LFGQALSFKVTVG-NGTVLVANNVAPADWKFGQTFE-DD--KNF

>CcEXPA17 [AB]

W--TP----SHVTFYGDIT--GAATM----G-GSCGYG-NLWWRGYGLNTAALS--------
DTLLNYGLTCGACFEIKC

DISS----GTYSTRWCYP-QNPSIIVTATNHCPP-NWDRPTD--NGGWC-NPPRTHFDLPVVMFMRLA----
---QTIA-

GIIPVQYRRVPCV-KTGGLKFTL----NGNPW-FNLVLIWNVAGGGDVMNMQMR-GEKT-V-WY--
TMRRNWGQYWE-CP

IR---FTGQKLSFIVTLG-NGQVRVVNNLTPSIWYFGQSFE-AL--TNF

>CcEXPA18 [AB]

W--TD----SHATFYGNDS--GEATM----G-GACGYG-NLWWRGYGLSTTALS--------
DTLLNDGLTCGACFEMKC

DIDR---PNSDSTRFCHS-SNPVIKVTATNRCPP-NPDRPTD--NGGWC-NPPRTHFDLSLNMFRRIA----
---RPVA-

GIVPVQYRRTSCV-KTGGIKFYL----GGNPW-FNLVLVYNVGGWGSVSKMEMR-GSKT-D-WM--
TMTRNWGQNWE-SG

IN---YTNQSLSFIVTLS-DGNVRVIRNLTPTVWYQGQTYE-AN--SNF

>CcEXPA19 [AB]

W--TP----SHATFYGSYD--GVETM----G-GACGYG-NLWWRGYGLNTAALS--------
DTLLNNGLTCGACFQLRC

DLSH----DGYSTRWCYK-NVAPITVTATNHCPA-NWARPTN--DGGWC-NPPRTHFDLPLVMFNRMA--
-----QPVA-

GIIPIQYRRVPCK-KSGGIKFYL----GGNPW-FNLVLVYNVAGAGDVKSMRMR-GSKT-V-WY--
TMKRNWGQFWE-CP

IK---FTGQRLSFQVTVS-SGYVRIINNLTPTNWYFGQTYE-AS--TNF

>CcEXPA2 [A]

W--ND----AHATYYGGSD--ASGTN----N-GACGYS-NVFSLGYGTMTAALS--------
APLFNEGKTCGACFQIRC

AGEE----------ACYG---NVITVTATNLCPQ------GS--NGGWC-DYPKPHFDLSQPAFSQIA-------KTIA-

GHVTLQYQKVNCA-RTGGIRFNL----QGHTY-FIQALIYNVGGAGDVSAVSIK-GSKT-G-WI--
QMYRGWGQNWQ-TG

TV---LDGQALSFMVTTS-DGRTVYSNNVADSYWQYGQTFE-G---SQF

>CcEXPA20 [AB]

W--AD----AHATFYGGQD--ASGTM----G-GACGYG-NLYSVGYGTSSTALS--------
NALFNNGLSCGACFQIIC

RLGE--------SKYCYQ--GASIVVTATNACPP------GS--EGGWC-DPPKAHFDLSYPAFQRLA-------
QPVG-

GVIPVKYKRVSCN-RNGGIRFTM----RGNPY-FDLVLVTNVGEFGAVQSLQLK-GERT-G-WN--
YMRQNWGANWQ-SN

VN---VQGQALTFKVTLD-NGRTGEFWNVAPRNWGFGQTFE-AD--YNF

>CcEXPA21 [A]

W--EV----AHATYYGGSD--AQGTN----G-GACGYQ-NVFALGYGAMTTAIS--------
PQLYGVGNTCGACFEVRC

SGDK----------ACYD---NSVAVTATNQGPQ-------------WSTEYPGPHFDLSQPAFNKIA-------DLIA-

GHIPIQYRRVNCV-KKGGILFAV----SGQTY-FMQVLVYNVGGKGDVNALAVK-GERS-G-WI--
EMNRLWGQYWS-TQ

AI---LDGQAMCFRVTTS-DGICLVSYNAAPSSWRYGQTYE-G---LQF

>CcEXPA22 [AB]

W--TD----AHATFYGGQD--ASGTM----G-GACGYG-NLYSRGYGTASTALS--------
NALFNAGLTCGACFQITC

KTSE--------TRWCYN-GGQSITVTATNQCPQ------GS--EGGWC-DYPRSHFDLSFPMFQQLA-------
QRVG-

GVIPVLYRRVSCQ-RAGGIRFSI----NGNPY-FNLVLVTNVGGKGDVAAMWMR-GSKT-D-WQ--
PMKQNWGQNWQ-CD

TK---LVGQSLSFKAMLG-SGETKEFWNVADSNWGFGQTFE-AD--YNF

>CcEXPA23 [AB]

W--TD----SHATFYGGND--GSGTM----G-GACGYG-NMYTTGYGLQTTALS--------
PTLFNDGLTCGACFQLKC

KVED--------TQWCYP---NRITVTATNLCPA-NPAKPND--DGGWC-NPPRTHFDLTYSMFSILA-------
QPVG-

GIIPVQFRRVSCI-KQGGVRFTL----NGNPW-FNLVLVYNVGGDGNIVSARMK-GSDT-S-WF--
TMTQNWGQNWE-LN

EE---LLGQALSFQLTTG-FGKTMIFSNVADSNWGFGQTWE-GE--TNF

>CcEXPA24 [AB]

W--TD----SHATFYGGND--GSGTM----G-GACGYG-NLYTTGYGLQTTALS--------
STLFNEGLSCGACFEIKC

KVDD--------TQWCYP-NAASITVTATNLCPE-NSDRPTD--NGGWC-NPPRTHFDLTYNMFSILA-------
KPVG-

GIIPVQFRRVSCI-KQGGLRFTL----NGNPW-FNLVLIHNVGGGGNIVSAQMK-GSDT-S-WF--TMTQNWGQNWE-LK

QK---LQGQAISFQLTTG-LGRTIVFSNVADSNWSFDQTWE-AD--TNF

>CcEXPA25 [AB]

W--TD----SHATFYGGND--GSGTM----G-GACGYG-NLYNTGYSLQTTALS--------PTLFNQGFSCGACFELKC

KVED--------TRWCYP-NATSITVTATNLCPA-NPDRPTD--NGGWC-NPPRTHFDLSYTMFSVLA-------QPVG-

GIIPVQFRRVSCI-KQGGVRLTL----NGNPW-FNLVLIYNVAGDGNIVSAQLK-GSNT-A-WF--TMTQNWGQNWE-LK

QK---LQGQALSFQLTTG-LGETMIFANVADSNWSFGQTWE-AD--TNF

>CcEXPA26 [AB]

W--TD----SHATFYGGND--GSGTM----G-GACGYG-NMYNTGYGLETTALS--------STLFNEGLSCGACFEIKC

KVDD--------SRWCYP-SAGSITITATNLCPA-NPDRPTD--NGGWC-NPPRTHFDLAYTMFSVLA-------QPVG-

GIIPVQFRRVTCI-KQGGVRFTL----NGNPW-FNLVLIYNVAGDGNIVSAQMK-GSDT-A-WF--TMTQNWGQNWE-LK

QK---LQGQALSFQLTTG-LGTTMVFANVADSNWSFSQTWE-AD--TNF

>CcEXPA27 [AB]

M---D----AKATFYGGTD--ASGTM----S--GCGYA-NPFSLGYGAQTAALS--------TPLWNKGLTCGGCFEIKC

KITA----TDYARKWCYS-NAPSIYITATNLCPP------GS--YGGWC-DPPKLHFDLAYAAFTKLA-------NQAA-

GVIPVQYRRTPCR-KTGGVKFQM----NGNPY-WALVLIYNVAGGGNIHAAAIK-GSNT-N-FY--TMNQNWGMNWE-AY

VS---LRGQALTFRLTLG-NGRSVTFYNVCPSYWQIGQTCQ-SA--YNF

>CcEXPA28 [AB]

M---D----AKATFYGGQD--ASGTM------GGCGYA-NPFTLGYGAQTAALS--------TPLWNKGLTCGACFQIRC

KISA----SAYAKKWCYA-SNPAITITATNLCPP------GS--YGGWC-DPPKLHFDLAQAAFTRLA-------NQVA-

GVIPIQYRRTPCR-KSGGIKFTM----NGNAY-WILALVHNVAGGGNVHAMQIK-GSRT-N-FM--SMRQNWGQNWE-SY

SQ---LVGQSITFKVTLG-NGRTITFWNVVGSGWRFGQTWQ-AR--YNF

>CcEXPA29 [AB]

W--GE----AHATFYGGQD--ASRTM----G-GACGYG-NLYSTGYGTANTELCIIQRWTYMRSMLYSGLQIGGKHMVLF

-------------------GGKSIIVTATNSCPP------GS--EGGWC-DPPRLHFDLSYPMFQALA-------QPVG-

GVIPVRYKRVPCA-GQGGVRFNL----LGNQW-FNYVLIYNVKGGDNVASILIK-GTKT-N-WV--EMYHNWGQFWS-AN

EN---LVGQALSFKVILG-DGSSLVFQDVTPDHWQFGQTYE-ADNNQNF

>CcEXPA3 [AB]

F--GD----ATATFYGGND--AAGTA----G-GSCGYE-NPFTRGYGVTTAALS--------NSLLRDNLNCGACFQVKC

KYYA----SAYTQKWCYK--DKTAIVTATNQSPP------GL--NGEHH-------FDLTYPMFTKIS-------NSIA-

GRIPIQYRRVPCK-KQGGIKFTL----NGNR--FNLVLVYNVGGAGNVYAMSMK-GSKT-G-WY--PMGRNWGQNWQ-AM

VR---LTGQSLSFRVSLG-NGKSKDFYNVAAAGWNFGQSYM-AS--SNF

>CcEXPA30 [AB]

W--GD----AHATFYGGQD--ASGTM----G-GACGYG-NLYSTGYGTSTTALS--------KRLFNHGLTCGACFMLVC

KLEQ--------AEWCYS-GGKSITVTATNACPP------NS--EGGWC-DPPRLHFDLSYPMFQKLA-------SPIA-

GAIPIKYKRVSCV-RHGGVRFDL----LGNPW-FNYVRVYNVAGGGDVASASIK-GSKT-N-WV--EMYHNWGQYWS-SS

PC---LVGQALSFKITLG-NGNSQVFWDITPENWKFGETYQ-ALNPQNF

>CcEXPA31 [AB]

W--GD----AHATFYGSED--GSQTM----G-GACGYG-DLYSTGYGIANTALS--------NGLFNNGFTCGACFKLVC

KLEE--------TKWCYP-GEKSITVTATNACPP------GS--EGGWC-DPPRLHFDLSYPMFRILA-------QPIG-

GVIPVMYKRVSCI-KQGGIRFYT----LGHPT-FNYFLVYNVGGGGDVGAVSIK-GSKT-S-WL--ELNHNWGQYWS-GN

EY---LIGQALSFKVTLG-DGEFLLIFDVIPENWQFGMTYE-APNNQNF

>CcEXPA32 [AB]

W--SN----ATATWYGGVD--GSGTM----G-GACGYG-DLYSTGYGLATAALS--------
STLFKQGLTCGACFELKC

RLNE--------SRWCYP-NVGSIKITATNFCPE-NLNRPTD----GWC-NGDRLHFDLTNKMFTTLA-------
QYVA-

GIIPVNYRRVPCV-KQGGIRFTI----NGNAH-FNLVLVHNVGGSGNVLSVQMK-GLKT-M-WY--
TMNHNWGQYWD-CG

IT---FVGQSLSFRVTTG-YGQNFTFWNVASQSWLFGQTYE-AS--SNV

>CcEXPA4 [AB]

W--TS----AHATFYGGAD--ASGTQ----G-GACGYG-NMYTRGYGLKTTALS--------
YSLFNKGLTCGACFQIKC

DLAL-------KPRWCYT-NNPSVTVTATNSCPP-NWSKPSN--DGGWC-NPPRVHFDLSYPMFTQLS----
---QAVA-

GIIPVRYKRVKCY-KTGGIRFTV----NGNPY-FNLVLVHNVGGYGNVVAIYIR-NTNS-V-YY--
KMTQNWGANWE-DH

HK---LVGQKLTFKATLG-TGQTMIFKDVTDTYWRHGQTWE-AS--NNF

>CcEXPA5 [B]

F--GD----ATATFYGGND--AAGTA----G-GFCGYE-NPFTRGYGVTTVAIS--------
DGLLQNGINCGACFEVKC

KWDS----SDYTKKWCYK--DKTTVVTATNLSPP------GL--NGEYH-------FDLTYPMFTQIS-------
NMIA-

GRIPIEYRRVPCV-KQGGIRFTV----VGNPF-WNLVLVFNVAGGGNVHSLYAI-NQNY-D-WC--
YMNRNWGQNWQ-TS

TM---LTGRSLSFRVALG-NGVTQDFYDVADSNWRHGQTYE-AN--SNF

>CcEXPA6 [AB]

Y--GD----ATATFYGGND--ASGTM----G-GSCGYQ-NPFTLGYGVNTAALS--------
NSLLSDNLNCGACFEVKC

KYDA----SAYTRKWCYP-NSPTAIITATNLSPP------------GLN---GEHHFDLTYPMFTKIA-------NSIA-

GRIPIQYRRVRCY-RSGGIKFTM----TGNPF-FNLVLVYNVGGAGNVHGMAIK-GSST-N-FY--
PMSRNWGQYWQ-AM

VK---LTGQSVTFRVTLG-NGKTTTFWNVCPPNWQFGLTYA-AT--YNF

>CcEXPA7 [AB]

W--AD----AHATFYGGSD--ASGTM----G-GACGYG-NLYSLGYGTQSTALS--------NALFNNGLSCGACFMIQC

KLDG--------SKYCYP--GQSITVTATNACPP------GS--EGGWC-DPPRSHFDLSYPMFQKLA-------KPVG-

GVIPVRYKRTSCV-KSGGVRFTL----HGNPY-FNYVLVHNVAGWGDVSAVSIK-GDQT-G-WY--YMSQNWGQYWS-AS

TK---LTGESLSFKVTLG-DGKSLEFYNVADKNWGFGQTYE-TD--YNF

>CcEXPA8 [AB]

W--KA----GHATFYGGSN--AAGTM----G-GACGYG-NLYNTGYGLKTAALS--------PTLFNDGLTCGACFEMYC

NYRM-------ESRWCFA-SMPRIRVTATNLCPA-NWNRPTN--RGGWC-NPPRVHFDLPQPMFTKMA-------RYVG-

GIVPVVYRRVSCV-KQGGVKFTI----NGNPW-FNLILITNVGGPGDIKAVSVK-GSAQ-KYWT--YAKQNWGQKWE-VK

QK---LQGQALSFRLTSA-NGKVLVLYSAVPRTWRFGTTYQ-AR--SNW

>CcEXPA9 [AB]

W--RN----AHATFYGGSD--ASGTM----G-GACGYG-NLYSQGYGTDTTALS--------TSLFDNGAKCGACFAITC

YRSK----------WCLP-GKITIEVTATNFCPS-NPNVPSD--YGGWC-NPPRRHFDLSQPAFTRIA-------VYQG-

GIVPVLFKRVSCK-RSGQIRYTI----NGQRY-FNLVLISNVGGVGDVSAVSVK-AANS-KYWE--PMRRNWGQNWQ-SN

TI---LVGQSLSFQVTTS-DGKTVSVQDAVDSNWEFGQTFE-G---GQF

>MpEXPA1PRO

W--RN----AHATFYGGSD--ASGTM----G-GACGYG-NLYSQGYGTDTTALS--------AALFDNGAKCGACFAITC

YRSR----------WCLP-GKPILQVTATNFCPS-NPNLPND--NGGWC-NPPRRHFDLAQPAFTQIA-------IYEG-

GIVPVLFKRVPCK-RSGPIRYTI----NGQRY-FNLVLISNVGGVGDVSAVSVK-GANS-NYWE--PMRRNWGQNWQ-SD

TI---LVGQSLSFQVTTS-DGSTVTVLDAVASNWEFGQTFE-G---GQF

>MpEXPA2PRO

W--AD----AHATFYGGQD--ASGTM----G-GACGYG-NLYSVGYGTSSTALS--------NALFNNGLSCGACFQIVC

RLAE--------SKYCYP--GGSVVVTATNACPP------GS--EGGWC-DPPKAHFDLSYPAFQRLA-------QPVG-

GVIPVRYKRVSCS-RNGGIRFTI----RGNPY-FDLVLVTNVGEFGAVQSLQIK-GERT-G-WT--YMRQNWGANWQ-AN

VN---VQGQALAFKVTLD-NGRSLEFYNVAPKNWGFGQTFE-AD--YNF

>MpEXPA3PRO

W--TP----SHATFYGEYN--GVETM----G-GACGYG-NLWWRGYGLETAALS--------DTLLNNGLTCGACFQLKC

DLDH----GGYSTRWCYK-NVAPITVTATNHCPA-NWARPTN--NGGWC-NPPRTHFDLPLVMFNRMA-------QPVA-

GIIPIEYRRVPCE-KSGGLKFYL----SGNPW-FNLVLVYNVAGAGAVTSMKMR-GSQT-V-WY--SMKRNWGQYWE-CP

IK---FSNQRLSFQVTLS-SGHVRVINNLTPTNWYFGQTYE-AA--TNF

>MpEXPA4PRO

W--TP----SHATFYGDDS--GAATM----G-GACGYG-NLWWRGYGLATAALS--------DTLLNNGLTCGACFEIKC

NITG-----GESGKWCIQ-SNPSIKITATNQCPP-NFDRPTD--NGGWC-NPPRTHFDLSLNMFRRLA-------QTQA-

GIIPISYRRTRCI-KTGGIRFYL----GGNAH-FNLVLVYNVGGWGDVMKMQMR-GTKT-G-WI--TMKRNWGQNWE-SS

IN---YTGQSLSFIVTLG-NGQVRVIRNLTPTVWYFKQTYE-AD--TNF

>MpEXPA5PRO

W--TD----SHVTFYGASD--GIETM----G-GACGYG-NLWWRGYGLETAALS--------DTLLNDGLTCGACYQIKC

NIAS----GGYSTKWCYP-SNPTIQVTATNRCPP-NWDRPTN--AGGWC-NPPRTHFDLPLVMFERMA-------QAVA-

GIIPVKYRRVPCV-KSGGIKFFL----GGNPW-FNLVLVYNVAGGGNVKNMQMR-GDKT-V-WY--TMKRNWGQFWE-CP

IG---FRGQKLSFIVTLS-NGQVRVINNLTTELWYFGQTYE-AL--TNF

>MpEXPA6PRO

W--ST----AHATFYGGSD--ASGTN----N-GACGYS-NVFALGYGTMTAALS--------
APLFNDGLSCGACFQIQC

SGDA----------ACYS-N--SITVTATNLCPQG------S--YGGWC-DYPNAHFDLSQPAFSQLA-------
QTVA-

GHVTVNYQRVNCN-RQGGVRFQI----DGHTY-FMQTLIYNVGGSGDVVAVQVK-GSKT-G-WI--
SMTRGWGQNWQ-TG

TV---LDGQALSFSVKTS-DGKTLYHNNVADSNWKYGQTFE-G---AQF

>MpEXPA7PRO

F--GD----ATATFYGGND--AAGTA----G-GSCGYE-NPFTRGYGVTTVALS--------
NSLLSNNLNCGACFEVKC

KYTA----SAYTKKWCYN--NKSVVVTATNLSPP------GL--NGEHH-------FDLTYPMFTKIS-------NMIA-

GRIPIQYRRVPCK-KQGGIKFTL----NGNPY-FNLVLVYNVGGAGNVYAMSMK-GSKS-D-WY--
PMARNWGQNWQ-AM

VR---LTGQSLSFRVTLG-NGKTQTFSNVAAADWKFGQTYQ-AS--SNF

>MpEXPA8PRO

W--TK----AHATFYGGDD--ARGTQ----G-GACGYG-NMYTRGYGVKTVALS--------
YTVFNEGLTCGACFQIKC

DLSM-------KPRWCYA-NGGIITVTATNSCPP-NWARPTN--NGGWC-NPPRVHFDLSYPMFAQLA----
---QKVA-

GIIPIQYKRVKCV-RSGGIRFTI----NGNPY-FNIVLVHNVGGYGNVVTLYIK-GTNS-P-WF--
KMKQNWGANWE-DH

NK---LTNQKLSFKATLG-TGETMIFKDITGTYWSHGQTYE-AN--NNY

>MpEXPA9PRO

W--SP----AHATFYGGSD--AQGTQ----G-GACGYG-NLYRIGYGINTAALS--------
TALFANGLSCGACFELKC

NSPR----------WCKS-GRNPIRITATNLCPQ-NHAHPNN--AGGWC-NMPLQHFDMAQPAYEQIA------
-IYRA-

GIVPVMYRRVQCI-KFGGIHFTM----NGNRW-EVLVLLTNVGGAGNVHAVSVK-GPHT-N-WL--
YMRRNWGQNWQIND

KQ---LAGATLSFRVTTE-DGRVVTSWDVVPHYWRFGQTFE-G---LNF

>MpEXPA10PRO

W--NY----AHATFYGDET--AQDTM----Q-GACGYG-NMYQQGYGTATTALS--------
STLFDNGFACGRCFEIRC

VASW----------ACYL-GNPSTIVTATNFCPA-NWLKPTN--NGGWC-NPPRQHFDMSKPAFEKIA-------
AWRA-

GIVPVAYRRVPCS-KQGSMEFRI----EGNPW-WHIVFITNVGGPGDIGEVYIR-GSGT-KRWV--
RMSHNWGVGFQ-VF

GG---YLGQGLSFMVISSSNLETVMAWNVVPANWRLGQTFS-G---GQF

>MpEXPA11PRO

W--MS----AKATFYGGQD--ASGTM----N-GGCGYA-NPFTLGYGAQTAALS--------
NALFNKGLTCGACFEIKC

AITA----TAYAKQWCYA-NSPSIYITATNLCPQ------GS--YGGWC-NGANLHFDLAYAAFTKLA-------
RENA-

GVIPVNYRRTPCK-KSGGIKFQL----NGNTY-WNLVLIYNVGGGGNVAAAAIK-GSRS-N-FY--
TMNQNWGMNWE-SY

SN---LVGQALTFKITLG-NGRSATFYNVAPAGWRFGQTYQ-AA--YNF

>MpEXPA12PRO

M---D----AKATYYGGND--ASGTM----N-GGCGYA-NPFALGYGDQTVALS--------
TALWNKGLTCGACFEIKC

KISA----TAYAKKWCYP-KAGSIKITATNLCPP------GS--HGGWC-DPPKSHFDLAYAAFTRLA-------
NFQA-

GVIPIQYRRTPCR-KNGGVKFSM----NGNPY-WHLVLVHNVAGGGNVHAMQIK-GSRT-G-FM--
QMKQNWGQNWE-AY

CN---LVGQSVTFKVTLG-NGRTVTFWNAVASNWKFGQTYQ-TK--YNF

>MpEXPA13PRO

W--AE----AHATFYGGQD--AGGTM----G-GACGYG-NLYSLGYGTATTALS--------
NALFHNGLSCGACFLIEC

ILQG--------SKWCYP--GKSIVVTATNACPP------GS--EGGWC-DPPRPHFDLSFPMFQMLA-------
QPVA-

GVIPVRYRRVSCS-KQGGVRFTL----HGNPY-FNYILVHNVGGSGDVAAVALR-GENS-R-WN--
RMNQNWGQYWS-CG

GW---YLGQALSFKVTLG-SGRSLEFWNVVDKNWGFGQTYE-SD--YNF

>MpEXPA14PRO

W--AD----AHATFYGGQD--ASGTM----G-GACGYG-NLYSTAYGTASTALS--------
NALFNNGLSCGACFQIMC

KLDG--------SKYCYT-GGKSITVTATNACPP------GS--EGGWC-DPPKAHFDLSYPMFQQLA-------
EPVG-

GVIPVQYKRVSCQ-KNGGIRFTI----NGNAW-FDLVLVTNVGGGGDIAKLEMK-GAST-D-WC--
PMSQNWGQNWQ-AS

GHPE-LCGQTLSFRATLG-DGRSQEFYDVAPSNWGFGQTFE-AN--TNF

>MpEXPA15PRO

W--AD----AHATFYGGSD--ASGTM----G-GACGYG-NLYSLGYGTSSTALS--------
NALFNNGLSCGACFLIQC

KLEG--------SKYCYP--GQSITVTATNACPP------GS--EGGWC-DPPRSHFDLSFPMFQKLA-------
QPVG-

GVIPVRYKRVSCT-KQGGVRFTL----HGNPY-FNYVLVHNVAGWGDVSSLAIK-GDQT-G-WY--
YMSQNWGQYWS-AS

AK---LTGQALSFKVTLG-NGKSLEFYNVADKNWGFGQTYE-SD--YNF

>MpEXPA16PRO

W--GD----AHATFYGGQD--ASGTM----G-GACGYG-NLYSVGYGVSTAALS--------
YAMFNKGLTCGACYELVC

KLEG--------SKYCYP--GKSITITATNSCPP------GS--EGGWC-DPPKLHFDLSYPMFQKLA-------
QPVG-

GVIPIKYKRVACA-KSGGVRFTI----HGNPY-FNYILVHNVAGAGDVSALAIK-GEKT-G-WY--
YMSQNWGQFWS-AS

AN---VVGQALSFKITLG-NGVSTEFSNVAPKDWKFGQTFE-AD--NNF

>MpEXPA17PRO

WEVTEFTRGARATYYGGND--ASGTL----G-GACGYG-NLFENGYGVETTALS--------
TALFNDGLTCGGCFQIQC

IASE--------SDWCLANGTVSITVTATNFCPP------GS--FGGWC-DPPQPHFDLSYPMFATLA-------
QPVG-

GVIPVQYRRVSCL-KQGGVRFQI----NGNNW-FFLVLVMNVAGAGDVQAVAIK-GTRS-E-WI--
SMQRNWGQNWA-SD

GPPE-LVGQALSFRVTLGLTKETLDFIDVAPADWQFGQTFE-DDSGLNF

>MpEXPA18PRO

C--IP----ATATFYGGND--ASGTN----T-GACAYP-NVLAMGYGTMNAALS--------
SAIFDHGKTCGACFQLVC

TYVP------YGNLRCLR---GRITVTATNYCPQ------GS--KGGWC-DAPRSHFDLAEPAFVQIA-------
PYNA-

GVVHVDYQRVNCQ-RSGEIRYRL----YGFTY-FLQVLVYNVAGTGDVTAMSIK-GSSE-GRWI--
SMTRSWGQLWT-TG

VV---LEHQALSFMVTIS-DGRTITVNDVANEYWRYGQTYE-G---GQF

>MpEXPA19PRO

W--TD----SHATFYGGNN--AQGTM----G-GACGYG-NMYSRGYGLETTALS--------
STLFNKGLTCGACFEIKC

KVAD--------TRWCYA-NAGSIKVTATNLCPA-NPARPTN--NGGWC-NPPRTHFDLSYPMFTRLA-------
KAVG-

GIIPVQFRRVPCV-KVGGIRFVM----NGNPW-FNLVLVYNVAGAGNVVNMQMK-GSKT-N-WF--
TMKQNWGQNWE-LK

QK---LKGQSISFRVTLG-NGKVMIANNVAPANWNFQQTFE-SD--NNF

>MpEXPA20PRO

W--MD----AHATFHGGSA--VADTM----G-GACGYG-NLISRGYGLHTSALS--------
SSLFNSGLTCGACFKVRC

KAAS--------SMRCHA-TAGSITVTATDLYSE-NLATLIE--DERRS-NPSQPQFDLAVSAFQMLA-------
RPMV-

GLIPIEYQRVPCE-KEGGIRFTL----NGNSW-FNLVLVHNVGGDGNVVAVQVK-GSRA-SSWS--
MMNRRWGQNWE-LR

EN---LQGQILSFRITTG-FGKTVLLPDVAQADWRFGQTYE-SE--GNF

>MpEXPA21PRO

W--TD----SHATFYGGND--GSGTM----G-GACGYG-NMYSRGYGLKTAALS--------
STLFNNGLSCGACFELKC

KVAE--------SRWCYA-NAGSITVTATNLCPA-NPARPTN--NGGWC-NPPRTHFDLAYAMFIIMA-------
KPVG-

GIIPVQYRRVSCV-KTGGVRFTL----NGNPW-FNLVLVYNVAGDGNVVTIKMK-GSKS-NTWY--
PMKHNWGQNWE-LK

QK---LAGQALSFQLTTG-YGKTMTFTNCAAANWQFGQTYE-AD--GNF

>MpEXPA22PRO

W--TA----SDATFYGGNS--GAGTM----G-GACGYG-NMYNTGYGLNTAALS--------
PVLFNNGLSCGACFELKC

NLTG--------TKWCYP-SAGSITVTATNLCPA-NPARTTD----GWC-NPPRTHFDLSYTMFAILA-------
QPVA-

GIIPIQYRRVSCV-KTGGVRFVI----NGNPW-FNLVLIYNVGGDGNVVTAMMK-GENT-E-WY--
SMTRNWGQNWQ-LS

QK---LHGQALSFQLTTG-YGTTLVCSDVAPANWQFGQTFE-AY--TNM

>MpEXPA23PRO

A--IP----ATATFYGGND--ASGTN----T-GSCGYP-NVLTID-GPMNTALS--------
GALYNGGEACGACFKIQC

VSIP------FGNLQCLP---GSIVVTATNLCPQ------GS--TGGWC-DEPRSHFDLAEPAFQHLA-------
PPVA-

GVVNVEYERVSCL-RSGGIRFLI----QGHPY-FMQVLVYNVGGMGDVTAVSVK-GSSS-D-WI--
QMDRNWGQLWT-TG

TV---LDGQALSFSVTTS-DGRTVASNGVADSDWQYGQTFE-G---NQF

>MpEXPA24PRO

A--IP----ATATFYGGND--ASGTN----TAGSCGYP-NVLTID-GPMNTALS--------
GALYNGGEACGACFKIQC

VSIP------FGNLQCLP---GSIVVTATNLCPQ------GS--TGGWC-DEPRSHFDLAEPAFQHLA-------
PPVA-

GVVNVEYERVSCL-RSGGIRFLI----QGHPY-FMQVLVYNVGGMGDVTAVSVK-GSSS-D-WI--
QMDRNWGQLWT-TG

TV---LDGQALSFSVTTS-DGRTVASNGVADSDWQYGQTFE-G---NQF

>MpEXPA25PRO

W--TN----ATATFYGGDD--GAGTM----G-GACGYG-NMFTRGYGLETAALS--------
TVLFNNGLTCGACFMLKC

RLNE--------SRWCYS-NVSSITISATNFCPP-NLNRTTD----GWC-NLPRSHFDLSVKMFTTLA-------
RAVG-

GIIPVLYKRVPCV-KTGGVRFTL----NGNPW-FNLVLISNVGGQGNVVAAQMK-GSKT-A-WF--
NMRQNWGQNWE-LG

QK---LINQTLSFRLTLG-LGQTLTFNNLTTPDWKFGQTWE-AD--TNF

>MpEXPA26PRO

F--GD----ATATFYGGSD--ASGTQ----G-GSCGYQ-NPFALGYGIKTAALS--------
NSLLANNMNCGACFEVKC

KYDA-SAY---TKKWCYP-NSPTIIVTATNQSPP------GL--NG-------EHHFDLTEPMFMKIA-------NRIG-

GRIPIQYRRVRCY-KPGGIKFTL----TGNPF-FNLVLVYNVGGAGNVYGMAIK-GSRT-D-FY--PMSRNWGQYWQ-AF

VK---LTGQSLTFRVTLG-NGKSTTFWNVAPANWQFGQTVA-AT--YNF

>MpEXPA27PRO

F--GD----ATATFYGGTD--AAGTA----G-GSCGYE-NPFTRGYGVTTAALS--------NSLLSDGLNCGACFEVKC

NFGA----SDYTRKYCYN-GK-SVVITSTNLSPP------------GLH---GEHHFDLTYPMFTQIS-------NMIA-

GRIPVQYRRVPCK-KQGGIKFNI----NGNPY-FNLVLVYNVAGGGNVYAMAMK-GGNS-A-WC--TMGRNWGQNWQ-SS

VK---LTGQGLSFRVTLG-SGKMLVFNNVAPSSWQFGQTYQ-AS--SNF

>MpEXPA28PRO

F--GD----ATATFYGGSD--AAGTA----G-GFCGYE-NPFTRGYGVTTVALS--------DSLLSNGLNCGACYEVKC

KWEA----SDYTKKYCYQ--GMTTIVSATNLSPP------GL--NGEHH-------FDLTYPMFVKIS-------NPES-

GRIPIQYRRVPCQ-KQGGIRFSM----VGNPF-WNLVLVYNVGGWGNVHSLYVR-GSRT-D-WY--YMNRNWGQNWQ-CS

VN---LTGQKLSFRVVLG-NGQTSVFNNVADSNWKHGQTYE-AS--SNF

>MpEXPA29PRO

W--TD----SHATFYGGNN--AQGTM----G-GACGYG-NMYSRGYGLETTALS--------STLFNKGLTCGACFEIKC

KVAD--------TRWCFP-KAGSIKVTATNLCPA-NPARPTN--NGGWC-NPPRTHFDLSYPMFTRLA-------KAVG-

GIIPVQFRRVPCV-KVGGIRFVM----NGNPW-FNLVLVYNVAGGGNVVNMQMK-GSKT-N-WF--TMRQNWGQNWE-LK

QK---LKGQSISFRMTLG-NGKVMIANNVAPANWNFQQTFE-SD--SNF

>MpEXPA30PRO

W--TD----SHATFNDGNN--AQGTM----G-GACGYG-NISSRGYGLETTALS--------STLFNSGLTCGACFEIKC

KVEN--------STWCFP-NAGSIKVTATDLCPA-NPAMPTN--NGGWC-NPPRTHFDLSYQMFTRLA-------KAEG-

AIIPVQFRRVPCV-KVGGIRFTM----NGNPW-FNLVLVSNVAGAGNVVNLQMK-SSET-N-WFWMTARQISGQYWE-LN

QN---LMGQNISFRVTLG-NGKVMIANNVVPANWNFQQTFE-SD--INF

>MpEXPA31PRO

W--TD----SHATFYGGNN--AQGTM----G-GACGYG-NMYSRGYGLETTALS--------
STLFNKGLTCGACFEIKC

KLAD--------SRWCFP-KAGSIKVTATNLCPA-NPARPTN--NGGWC-NPPRTHFDLSYPMFTRLA-------
KAVG-

GIIPVQFRRVPCV-KVGGIRFVM----NGNPW-FNLVLVYNVAGAGNVVNMQMK-GSKT-N-WF--
TMRQNWGQNWE-LK

QK---LKGQSISFRVTLG-NGKVIIANNVAPANXNFQQTFE-SD--SNF

>MpEXPA32PRO

W--TD----AHATFYGGQD--ASGTM----G-GACGYG-NLYSRGYGTASTALS--------
NALFNSGLSCGACFEIIC

KLSE--------TKWCYT-GGKSIVVTATNQCPQ------GS--EGGWC-DYPRSHFDLSFPMFQSLA-------
QAVG-

GVIPVQYRRVSCS-RSGGIRFTI----NGNAY-FNLVLVTNVGGKGDVAALWMR-GSNT-D-WQ--
PMKQNWGQNWQ-CD

TK---LVGQALSFKAMLG-SGETKEFYNVADSNWLFDQTYE-GD--YNF

>MpEXPA33PRO

G--LK----AHATFLGGSD--AVGTN----N-GACGYS-NVLAMGYGTRTAALS--------
APIFREGKSCGACYKLRC

RGSE--------SVGCRS--NDGVVVTVTDVCPE------RS--VGGWC--DGQPHFRLSEPAFAQIA-------
EPKSG

GLVEVDYEKVSCD-RRGGMHFLL----VGHTH-FLQILVHNVGGCGDVAAVSVK-GGTG-P-WI--
RMSRSWGQLWH-TG

EV---LDGQALSISVTMA-DGKVCVSNNFAGADWKYGQTFE-G---AQL

>MpEXPA34PRO

A--LP----AHATFYGGSD--ASGTN----N-GACGYS-NVLSTPFGTMTAALS--------
SPLFNEGLSCGSCYKVRC

SGSS--------TGGCID---NEIMVTATNLCPH------GS--EGGWC--DGKPHFDMSQPAFSQIA-------
KPVA-

GVVEVEYTKVPCP-RKGGMQFTV----MGHTY-FLQVLIFNVGGSGDVTAVKVR-GGNG-E-WI--
AMTRSWGQLWQ-TG

KV---LDGQALSFQVTTS-NGKTCVSNYVAGSHWKYGQTFE-G---SQY

>MpEXPA35PRO

W--TD----SHATWYGGKD--GAGTM----G-GACGYG-NMNARGYGMKTAALS--------
TKLFNDGFTCGACFEIRC

KSSQ--------SKYCYS-STASVIVTATNYCPA-NPNRPTD----GWC-NGDRTHFDLTYQMFTTLA-------
IYEA-

GIIPVQYRRVPCA-KKGGIRFTI----NGNPN-FNLILLSNVGGSGNVVSVQMK-GENSYSGWV--
PMKQNWGQMWE-CG

TK---VTGQSISFKVTVG-YGQTFTFWTVAPKTWQFGQTYE-AS--SNV

>MpEXPA36PRO

W--GD----AHATFYGGND--ASGTM----G-GACGYG-NLYSLGYGTASTALS--------
NGLFNNGLSCGACYTIEC

KLEG--------SKWCYP--GKSITVTATNACPP------GS--EGGWC-DPPRLHFDLSYPMFQTLA-------
QPVG-

GVIPVKYKRVSCN-RQGGIRFTL----HGNAW-FNYVLVHNVAGDGDVAGVSIK-GDNG-G-WT--
TMYQNWGQFWS-AS

EH---LVGQALSFRVTLG-SGRTQEFYDVASPNWYFGQTYE-ADNNQNF

>MpEXPA37PRO

W--GN----AHATFYGGND--ASGTM----G-GACGYG-NLYSRGYGTATTALS--------
NGLFNNGLTCGACFTLVC

KLDG--------SRWCYS-GGKQITVTATNACPP------GS--EGGWC-DPPRLHFDLSYPMFQKLA-------
QPVG-

GVIPVRYKRVPCV-KQGGIRMSL----YGNPW-FDYVLVHNVAGGGDVAAVSIK-GSKT-N-WV--
VMHQNWGQYWS-AS

SY---LVGQALSFKVTLG-NGKSIVFWDIAPKNWQFGQTYE-ADNNQNF

>MpEXPA38PRO

W--KA----AHATFYGGSN--AQGTM----G-GACGYG-NLYNTGYGLETAALS--------
STLFNNGKTCGACYEMFC

NYRQ-------QSRWCYK-AQPHIKVTATNLCPA-NWNRPTN--RGGWC-NPPRVHFDLPQPMFIKMA---
----RYVG-

GIVPVAYRRVPCV-KKGGVKFTM----NGNPW-FNLILISNVAGPGDIKAVAVK-GSKQ-KSWT--
SARQNWGQKWE-VK

QK---LQGQALSFRLTSG-SGKVLVLYNAVPRTWRFGSTYM-AR--SNW

>PpEXPA1PRO

W--RK----AHATFYGGAD--ASGTM----G-GACGYG-NLYSTGYGVDSTALS--------
TALFNNGAKCGACFAIQC

YRSQ----------YCVP-GSPVITVTATNFCPP-NHKGDGT---PGWC-NPPMRHFDLAQPSFTKIA-------
KYRA-

GIVPVLFRRVPCE-KKGGVRFTI----NGNKY-FNLVLVHNVGGKGDVHAVDIK-GSNT-E-WI--
PMKRNWGMNWQ-TD

AV---MTGQALSFRVTTS-DGKTIVSMNATPSHWSFGQTFE-G---GQF

>PpEXPA2PRO

W--KE----AHITYYGTAN--GGGTQ----G-GACGYP-NTFAMGYGVMTAALS--------
YPLFQGGKSCGACYQLKC

KWLA---PTRTVHNWCWS-YSRTITITATNSCPP------GS--HGGWC--DWKPHFDLPMPAFMTLA------
-RREG-

GVAPVYYRKVRCA-KRGGIRFTL----GGNPY-FMMILIHNVGGAGDLRAVKIK-GRNG-Y-WV--
PMWQNWGALWT-CK

TK---LSG-ALSFQITTG-DGRTVTVNRAVGDYWKFGQTWE-G---SQF

>PpEXPA3PRO

WISGV----AHATFYGGVD--AQGTQ----G-GACGYG-NLYSTGYGTSTTALS--------
SALFNAGLSCGACFELKC

DSAN--------SKYCLP-GDKSITVTATNYCPQ------GS--DGGWC-DSPKQHFDLSHPMFTSLA-------
QEVG-

GVIPVTYRRAPCA-KKGGMRFTI----NGNPW-FVMILVTNCGGAGDVQQLQIR-GSDT-P-WY--
PCVRNWGQMWQ-MT

SDPN-LPGKALSFRATLS-DGSVAESLNAAPSNWGWGQTFE-GV--ATY

>PpEXPA4PRO

W--KD----AHITYYGSPN--GGGTQ----G-GACGYQ-NTYALGYGSFTAALS--------
APLFQGGAACGGCYQLKC

APVR---ETRTVHNWCWS-YSRSIVVTATNLCPP------GS--HGGWC--AWRPHFDLPMPAFTSLA------
-KQVG-

GVAPVFYRRVRCA-KRGGVRFTI----GGNPY-FLMVLIHNVGGAGDIRSVRIK-GQYS-G-WV--
TMFRNWGSLWT-CR

TK---LSG-PLSFMITTS-DGRTLVSNRAVGSWWKFGQTWE-G---SQF

>PpEXPA5PRO

W--GR----AHATFYGGAD--ASGTQ----G-GACGYG-NLYSTGYGTNTAALS--------
SSLFNSGLSCGACYELTC

DPSC--------SQYCLP--GGSAIITATNFCPT------GS--NGGWC-NPPKQHFDLAQPVFSKIA-------
RTVG-

GVIPINYRRVPCS-KSGGMRFTV----NGNPY-FLLVLVTNVGGAGDVQQLYIK-GAST-G-WL--
PLKRNWGQMWQ-FT

GNSG-MHGQAISFKAVTS-DGAEAISPNVAPANWGFGQTFE-G---SNF

>PpEXPA6PRO

W--RN----ARSTFYGGMD--AAGTM----S-GACGYG-NLYASGYGVHTTALS--------
SALFKNGMACGACFEVQC

GGKG---------KPCKP---GSVVVTATNFCPP-NPGQSAN--NGGWC-NPPNEHFDLSYPAFVKIA-------
DPKA-

GAVPLQYRRVPCQ-KQGGIRFTI----NGNCN-FILVTITNVGGSGVVTAAYLK-GDKT-E-WS--
PLSRNWGANWQ-CR

RN---YCGQGISIKIVTS-DNKVSVT-KLAKSDWCFGKTFI-G---KQV

>PpEXPA7PRO

W--GY----AHATYYXGAD--ASGTQ----GGGACGFG-NLYSTGYGTNTAALS--------
AALFNSGLSCGSCYELAC

DPNG--------SKYCLP-GGPTVTVTATNFCPH------GS--LGGWC-DAPKQHFDLAHPMFVSLA-------
REVG-

GVIPIKYRRVPCV-KSGGMRFTI----NGNPW-FLLVLVTNVAGAGDVQHMYIK-GSNT-P-WE--
PMSRNWGSMWQ-FT

GDSK-MKGQALSFKAVTS-DGSVAVSMDAAPGNWQFGQTFE-G---VNF

>PpEXPA8PRO

W--NE----AHATFYGGSD--AGGTTGTR-G-GACGYG-DLYSTGYGTSTVAIS--------
SALFDRGLACGACYQVKC

AGSS---------SECRS-DSPAIQVTVTNFCPP-NPSLPED--NGGWC-NLPLHHFDMSMPAFEQIA-------
TYKA-

GIVPVMYRRTSCV-RTGGIHFTM----SGHNF-MNLVLVTNVGGMGDVQSVSIR-GSKT-S-WV--
TMTRNFGQIWQ-ST

VN---MSGQSLSFMVTTS-DGKTVVSNNVAPPDWAFGQTYE-G---SQF

>PpEXPA9PRO

W--TD----AHATFYGGAD--ASGTQGGCAG-GACGFG-NLYSTGYGTNTAALS--------
QALFNSGLTCGACFELVC

DSSG--------SRYCVT--SSSVVVTATNFCPT------GS--TGGWC-DYPRQHFDLSQPVFTRIA-------
QPVG-

GVVTLKYRRVRCQ-KSGGIRFTI----TGNPY-FILVLVTNVGGAGDVQQLYIK-GSST-G-WN--
AMSRNWGQLWEIRN

AA---LMGQALSFRVVTS-DRAEVASFDAVPANWAFSQTFE-G---SNF

>PpEXPA10PRO

WVSGR----AHATFYGGAD--ASGTQ----G-GACGYG-NLYSTGYGTSTAALS--------
SALFNSGLSCGACYELTC

DTSG--------SKYCLP-GNPSIILTATNYCPQ------NS--NGGWC-DAPKQHFDLAHPMFVSLA-------
EERG-

GVIPVNYRRVPCA-KKGGMRFQM----NGNPW-FLLVLVTNVGGAGDVQQLSIK-GSNS-G-WY--
QMKRNWGQMWQ-LT

GNSN-MPGQALSFRAVLS-DGTTVESLDAAPANWHFGQMFE-G---SQA

>PpEXPA11PRO

W--GR----AHATYYGGAD--ASGTQ----G-GACGFG-NLYSSGYGTDTAALS--------
SALFNSGLSCGACYELTC

DPSG--------SKFCIP--GGSAIITVTNFCPT------GS--NGGWC-NPPKQHFDLAQPVFRKIA-------
RTVG-

GVVPINYRRVSCL-KDGGMRFTV----NGNPY-FLLVLVTNVGGAGDVQQLYMK-GSST-N-WQ--
PLKRNWGQMWQ-FT

GNSR-MHGQAISFKAVTS-DGSVAVSNNVAPPNWGFGQTF---------

>PpEXPA12PRO

W--ND----AHATYYGGAD--AS-------G-GACGFG-NLYSTGYGTSTAALS--------
QALFNSGLTCGACFELAC

DPSG--------SKYCYK--GSSIVVTATNFCPS------GS--EGGWC-DSPKQHFDLSQPVFNKIA-------
QQAG-

GVIPVKYRRVPCR-KSGGMRFTI----NGNPY-FILVLVTNVGGAGDVQQLSLK-GSST-G-WY--
TMSRNWGQQWE-FR

GNSA-LVGQALSFRVVTS-DGAEAVSYDAASENWSFSQTFE-G---INF

>PpEXPA13PRO

W--NE----ARATFYGGSD--AGGTT----G-GACGYG-DLYSTGYGTNTVATS--------
SAIFDRGLACGACYQVKC

AGSA---------SECQP-GTPAIQVTVTNFCPP-NPSLPEG--NGGWC-NLPLHHFDMAMPAFQQIA-------
SYRV-

GIVPILYRRASCV-RTGGIRFTM----SGHKF-MNLVLVTNVGGMGDVQTVFIQ-GSKT-K-LV--
AMIRNFGQIWQ-SS

VN---VSGQRLSFMVMTS-DGESVVSRNVAPSDWAYGQTYE-G---SQF

>PpEXPA14PRO

W--DK----GHATYYGEGD--ARGTM----G-GACGYS-NLYSTGYGVNTAALS--------
GPLFNGGATCGACYELTC

ILNE--------SKWCYR--GKNIIVTATNFCPS------GS--TGGWC-NPPQKHFDLSEPMFTTLA-------
NRVG-

GVIPVNFRRVACY-KQGGMRFTI----NGNPY-FFIVLVYNVAGAGDVQQVYIK-GPKT-Q-WL--
QMYRNWGSQWT-FN

GGPNNIVGSALSFRVHTS-DGRQVISYXAAPANWWFGXTFS-SG---AN

>PpEXPA15PRO

W--GY----AHATYYGGAD--ASGTQ----GGGACGFG-NLYSTGYGTNTAALS--------
AALFNSGLSCGSCYELAC

DPNG--------SKYCLP-GGRTVTVTATNFCPH------GS--LGGWC-DSPKQHFDLAHPMFVTLA-------
KEVG-

GVIPIKFRRVPCV-KSGGMHFTI----NGNPW-FLLVLVTNVAGAGDLQQVYIK-GSNT-P-WE--
PMSRNWGSMWQ-FT

GNSK-MKGQALSFKTITS-DGAVAISYDAAPNNWQFGQTFE-G---VNF

>PpEXPA16PRO

W--DT----AHATYYGGRD--AGGTM----G-GACGYG-NLYNTGYGVKTAALS--------
APLFKGGATCGACYELTC

ILSQ--------SKYCYQ--NKKILITATNFCPT------GS--TGGWC-NPPRKHFDLSEPMFTTLA-------
NRVG-

GVIPVNFRRVHCY-KKGGMRFTI----NGNPY-HMMVLVYNVAGAGDVQQMFIK-SPTT-G-WL--
QMSRNWGQIWT-YK

GGPRNIVGFALSFRVYTS-DRRQVVSYNAAPANWRFGQTFS-SG---AN

>PpEXPA17PRO

W--GR----AHATYYGGAD--ASGTQ----G-GACGFG-NLYSSGYGTSTAALS--------
SSLFNSGLSCGACYELTC

DPSG--------SQYCLP--GGSAIITVTNFCPT------GS--NGGWC-NPPRQHFDLAQPVFSKIA-------
RTVG-

GVIPINYRRVSCL-KSGGMRFTV----NGNPY-FLLVLVTNVGGAGDVQQLYIK-GSST-A-WL--
PLKRNWGQMWQ-FT

GNSG-MHGQAISFKAVTS-DGAEAISNNVASSNWGFGQTFE-G---SNF

>PpEXPA18PRO

W--NE----AHITYYGTAN--GGGTQ----G-GACGYP-NTFAMGYGAMTAALS--------
YPLFQGGKACGGCYQLRC

KWVT---PTRTVHNWCWS-YSRTITVTATNSCPP------GS--HGGWC--NWRPHFDLPMPAFLTLA------
-RREG-

GVAPVYYRKVRXA-KRGGIRFTI----GGNPY-FLMILIHNVGGAGDLKAVKVR-GGNG-Y-WV--
PMWRNWGALWT-CK

TR---MSG-ALSFQITTG-DGRTLTTYKAVGGYWRFGQTWE-G---SQF

>PpEXPA19PRO

W--GD----AHITYYGSPN--GAGTE----G-GACGYQ-NTYKLGYGSMTAALS--------
SRLFQGGKACGGCYQLRC

APNR-------GRNWCWS-YARAIVVTATNLCPQ------GS--HGGWC-DYPKSHFDLPMPAFTSLA-------
RREG-

GVAPVWYRKVRCA-KRGGVRFTI----GGNPW-FLMVLIHNVGGAGDVVSVKVK-CPYT-G-WV--
SAYRNWGCLWT-VR

TK---MTG-PLSFTLVTS-DGRTLYSMNAVRNGWKFGQTWE-G---SQF

>PpEXPA21PRO

X--RD----AHITYYGSPN--GGGTE----G-GACGYQ-NTYSLGYGFMTAALS--------
SSLFQGGSSCGACYQIRC

EPIR---VTRTVKNWCWS-YSRTITVTAINLCPP------RS--SGGCC-NPPLQHFDLPMPAFLSLA-------
RREG-

GVAPVXYTKVRSE-KRGGIRFTM----GGNLW-FLTILIHNVGGAGDVRSVRIK-SPHS-G-LI--
SMYRNWGSLWT-VR

AR---MSG-ALFFMITTS-HGRVLITRNAVGSGWRFGQTWE-----GNF

>PpEXPA22PRO

W--QD----AHITYYGSPN--GGGTQ----G-GACGYQ-NTVSLGYGFMTAALS--------
SPLFQGGKACGACFQLQC

ARVQ---ETRTVKNWCHD-YSKAITVTATNLCPP------GS--EGTWC-DPPRHHFDLPMPAFLSLA-------
RQEG-

GVAPVYYRRVQCL-KKGGIRFTM----GGNPW-FLMVLVHNVXGAGDVVXVKVK-CPSS-G-WY--
DMYRNWGALWT-VQ

KK---MVG-PLSFLLTTG-DGRRLTAYNAVGNGWTFGQTWE-G---AQY

>PpEXPA23PRO

W--IN----GRITYYGSPN--GGGTQ----G-GACGYQ-NTVSLGYGFMTAALS--------
TTLFKGGAACGACYQLQC

APVSETPSGLLKRNWCWQ-VGRSILVTATNLCPP------GS--SGGWC-NPPQHHFDLPMPAFLALA----
---RREG-

GVVPVYYRKVNCA-RKGGIRFTV----GGNPW-FLMILIHNVGGAGDVVAVKVK-CPTS-G-WY--
DMYRNWGALWT-VQ

KK---MNG-PLSFAITTS-DGRTVTTYNAVGNGWKFGQTWE-G---AQY

>PpEXPA24PRO

W--SS----GRITYYGSPN--GGGTQ----G-GACGYQ-NTVSLGYGFMTAALS--------
TPLFNGGAACGACYQLQC

APVHETPKNLLQRNWCWK-VGRRITITATNLCPP------GS--EGGWC-DPPQHHFDLPMPAFTALA-----
--KREG-

GVVPIYYRRVRCA-KQGGIRFTM----GGNPW-FLMILIHNVAGAGDVVAVKIK-CPTS-D-WC--
DMYRNWGAFWT-VQ

KK---MEG-PLSFQITTX-DRRKVTTHNAVGHGWQFGQTWE-G---AQY

>PpEXPA25PRO

W--RD----AHITYYGSPN--GGGTQ----G-GACAYQ-NTFSLGYGAMTAALS--------
SPLFEGGAACGACYQLQC

KRVQ---ETRTVKNWCWS-YSRTITITATNLCPP------GS--AGAWC-DPPRHHFDLTMPAFLTLA-------
RREG-

GVAPVLYRRVKCV-KRGGIRFTI----GGNPW-FLMILIHNVAGAGDVRAVRIK-TPST-D-WI--
PMYRNWGALWT-VQ

RK---LSG-PLSFQITAG-DRRQITINSAVGNAWKFGQTWE-G---HNF

>PpEXPA26PRO

W--NF----GRITYYGSPN--GGGTQ----G-GACGYQ-NTFALGYGTNTAALS--------
SRLFQGGAACGACYQLRC

IAPK------WGKNWCWN-YARSIVVTATNLCPS------GS--NGGWC--PSQAHFDXPMPAFTSLA-------
RKEG-

GVTPIMYRRVRCA-RRGGIRFTI----GGNPF-FLMVLIHNVGGAGDVRAVSIK-GQYT-G-WV--
GMYRNWGSLWT-CT

TK---IDG-ALTFRITTG-DGKTLILYNAVRKGWRFGQTWE-G---SQF

>PpEXPA27PRO

W--AT----ATATFYGGAD--AG-------G-GACGYG-NLYSTGYGASTTALS--------
APLXNGGSACGACYQLQC

ARSN----------HCYA--GRSITVTATNFCPT------GS--EGGWC-NPPRKHFDLSMPMFTTLA-------
RQVA-

GVVPVDYRRVACN-KKGGQRFLM----TGNPY-FIMVLVYNVAGAGDVQRFFVK-GSMT-G-WY--
ELRRNWGQIWT-CT

ADSR-LKGQALSFRTQTS-DGRQVVSIDAAPANWNFGQTFS-SG---VN

>CpEXPA1 [AB]

WIGGN----AHATYYGGVD--ASGTQ----G-GACGYG-NLYSTGYGTNTAALS--------
SALFNSGLTCGACYELQC

DSTG--------SKYCLP-GNPSITITATNYCPQ------NS--LGGWC-DAPRQHFDLAHPMFVSLA-------
QEGG-

GVIPVNYRRVPCV-KKGGMRFQL----NGNPW-FVLVLVTNVGGAGDVQQLYIK-GDKT-G-WY--
PMKRNWGQMWQ-LT

GNSN-MPGQALSFRAVLS-DGTNVESMDVAPANWRFSQLFE-G---SQA

>CpEXPA2 [X]

S--GT----GHITFYGTPN--GGGQA----SRGACGYQ-NTYALGYGSMTAALS--------
SPLYQGGAACGACFQLTC

MRIR---ETKTAKNWCWS-YSRSITVTATNLCPP------GS--RDGWC-NPPRNHFDLPYPAYLSLA-------
KREG-

GVAPVTWRRVPCR-KRGGIRFTV----GGNPW-FFMILIHNVGGAGDVRSVKIG-CPYT-G-WV--
PMYRNWGALWT-VR

RQ---MRGQ-LSFLITTS-DGRSVVARNAVGKYWKFGQTWECG---AQF

>CpEXPA3 [X]

S--GT----GHITFYGTPN--GGGQA----SRGACGYQ-NTYALGYGSMTAALS--------
SPLYQGGAACGACFQLTC

MRIR---ETKTAKNWCWS-YSRSITVTATNLCPP------GS--RDGWC-NPPRNHFDLPYPAYLSLA-------
KREG-

GVAPVTWRRVPCR-KRGGIRFTV----GGNPW-FFMILIHNVGGAGDVRSVKIG-CPYT-G-WV--
PMYRNWGALWT-VR

RQ---MRGQ-LSFLITTS-DGRSVVARNAVGKYWKFGQTWECG---AQF

>CpEXPA4 [AB]

W--GK----GKCTFYGGMD--AAGTM----S-GACGYG-NLYASGYGAYTAALS--------
SALFKNGMACGACYEVAC

SGNA---------KSCKR---GSVLVTITNFCPP-NPGRSAN--NGGWC-NGANQHFDLSYPAFAKIA-------
DPKA-

GVVDLNYRRVPCQ-KKGGIRFTL----NGNCN-FNIVTITNVGGSGVVTQAYMK-GDGM-D-WA--
PMTRNWGANWQ-CS

RN---YCGKGISIKIVTS--DKKVSVSRLADKTWAFGKTYI-G---QQV

>CpEXPA5 [AB]

W--NS----AHATYYGGAD--ASGTQGERRG-GACGFG-NLYSTGYGTSTAALS--------
QALFNSGLTCGACFELAC

DSSG--------SKYC-E-GAPSVVVTATNFCPS------GS--NGGWC-DQPKQHFDLAMPVFDKIA-------
QEVG-

GVIPVKYRRVPCQ-KKGGMRFTI----NGNPY-FILVLVTNVAGAGDVQQISIK-GDNT-D-WF--
AMKRNWGQQWQ-FS

GNSA-LVGQALSFKVVTS-DGAVATSMNAAPANWQFSQTFE-G---VNY

>CpEXPA6 [B]

W--RG---GGHITFYGGPN--GAGTQ----A-GACGYQ-NTYKLGYGSMTAALS--------
SPLFQGGAACGACFQLRC

APVR---RSRTARNWCYS-YAKTITVTATNLCPS------GS--TGGWC-NPPRAHFDLPMPAFLTLA-------
RREG-

GVAPVFYRRVRCA-KRGGIRFTI----GGNPW-FLMVLIHNVGGAGDVRSVRIK-GQYT-G-WY--
PMYRNWGALWT-VR

TK---LSG-ALTFAVTTS-DRRTVITRSV-GGNWRFGQTWE-G---ANF

>CpEXPA7 [B]

W--KG---GAHITFYGSPN--GGGTQ----A-GACGYQ-NTYKLGYGSMTAALS--------SPLFRGGSACGACYQIRC

APVR---RTRTARNWCYS-YARTITITATNLCPP------GS--TGGWC-NPPRAHFDLPMPAFLTLA-------RREG-

GVAPVYYRKVRCA-KRGGIRFTI----GGNPW-FLMVLIHNVGGAGDMRSVRVK-GQYT-N-WY--PMYRNWGALWT-VR

TK---LSG-ALSFAVTTS-NGRTVVARNVVGKWWKFGQTWE-AR--TNF

>CpEXPA8 [X]

W--GY----AHITFYGTPN--GGGQA----SRGACGYQ-NTYALGYGAMTAALS--------SPLYKGGAACGACFELKC

IRIR---ESRSAKNWCWS-YSRSITITATNLCPP------GS--TGGWC-NPPKHHFDLSYPAYATLA-------RREG-

GVAPVNYRRVPCR-KRGGVRFTV----GGNPW-FFMILIHNIAGAGDVRSVKIM-CPYT-G-WV--PMYRNWGALWT-VR

KQ---MRG-PLSFAITTS-DGRTIISRNAVGNYWKFGQTWE-GR--AQF

>CpEXPA9 [AB]

W--NT----AHATFYGGSD--AGGTE----G-GACGYG-DLYSSGYDTNTVAIS--------SALFDRGLACGACYQVKC

AGKA---------SECHP-DSPSIQVTVTNFCPP-NPSLPDN--NGGWC-NLPLHHFDMSEPAFQRIA-------TYKS-

AIVPVMYRRTACM-KSGGIRFTM----SGHNF-MNLVLVTNVGGMGDVQSVYIR-GSKT-K-WV--AMARNFGQIWQ-ST

VN---MSGQSLSFMVTTS-DGQTVVSNEVAPPDWAFGQTYE-G---SQF

>CpEXPA10 [AB]

W--RK----AHATFYGGAD--ASGTM----G-GACGYG-NLHSTGYGLDTTALS--------TALFNNGAKCGACFAIQC

YRSK----------YCLP-GTPIITVTATNFCPP---NRQGD-GTPGWC-NPPLRHFDLAQPSFVKVA-------KYRA-

GIVPVLFRRVPCQ-KKGGVRFTL----NGNKY-FNLVLVHNVGGKGDVRSVDIK-GSDT-E-WM--PMKRNWGMNWQ-TH

TV---MVGKALSFRVTTS-DGVTVTSMDAAPPQWRFGQTFE-G---AQF

>CpEXPA11 [AB]

W--SP----AHATFYGGAD--ASGTQ----G-GACGYE-NLYNQGYGINTAAVS--------APLFNDGAGCGACFELVC

DSSQ--------SDYCYS-NASSIIVTTTNFCPK---APEGT--ENAWC-DPPRIHFDLSEPIFLTFA-------QKVA-

GIVPVNYRRVPCL-KDGGIKFTI----NGHKY-FFLVLVTNVAGAGDIQELYIK-ASNT-D-WQ--PLTRNWGDNWE-LN

QNTLNFVGQALSFKAVAS-DKSEVVSMNVTPVDWEFSQTFE-G---SNF

>CpEXPA12 [A]

WTSGQ----AHATYYGGAD--AAGTQ----G-GACGFG-NLWSTGYGTDTAALS--------AALFNSGLSCGACYELKC

DTAG--------SKYCLP-GNPSITVTATNYCPQ------GS--EGGWC-DTPKQHFDLAHPAFTKLA-------HEGG-

GVFPVVYRRVPCA-KQGGMRFQI----NGNPW-FLLVLVTNVGGAGDVQQLQVK-GERT-G-WY--QMVRNWGQMWQ-FT

GDHT-LPGQALSFRATLS-DGSVVESWNCAPKNWGFQQVFE-GT--PAH

>CpEXPA13 [X]

W--RD---DGHITYYGSPN--GHGTQ----G-GACGYQ-NTFSLGYDHLTAALS--------TPLFQGGAACGACYELRC

KFIK---ESKTAKNWCWN-YSRSITSTATNLCPP------GS--TGGWC-DPPRNHFDLPMPAFLTLA-------RQNG-

GVAPVHYRRVPCQ-KKGGIQFTI----GGNPW-FYMILIHNVAGAGDVVRVQIK-SPTT-G-WT--PMYRNWGALWT-VR

KQ---LKG-ALSFQITTS-DGRVVVTHNAVGHGWRFGQTWE-G---AQY

>CpEXPA14 [AB]

L--GR----AHATYYGGPD--GQGTM----G-GACGYG-NLYNTGYGLSTAALS--------APLYNGGRACGACYRLTC

DTSG--------SKYCYG-GGKSITITVTNFCPS------GS--TGGWC-NPPKQHFDLSYPMFTTLA-------QAAG-

GVIPVNYRRVACN-KKGGMRFTI----SGNPY-FYQVLVFNVGGAGDVQRLFMK-GPRT-G-WI--AMKRNWGQVWQ-YR

GGPKGLVGQPLSFRAHTS-DGRYVISPNATPANWRFGQTFE-G---KNF

>CpEXPA15 [AB]

W--GY----AHATYYGGAD--ASGTQ----G-GACGFG-NLYSTGYGTNTAALS--------
AALFNTGLSCGSCYELAC

DPNG--------SKYCLP-GGRTVTVTATNFCPH------GS--LGGWC-DSPKQHFDLAHPMFVSLA-------
KEVG-

GVIPIKYRRVPCI-KSGGMRFTL----NGNPW-FILVLVTNVAGAGDVQQLYIK-GSRT-Q-WE--
RMSRNWGSMWQ-FT

GNSN-MKGQALSFKAITS-DGSVAISTDAAPGNWQFGQTFE-G---SNF

>CpEXPA16 [AB]

W--DN----GHATFYGGDN--GAGTM----G-GACGYG-NLYNTGYGLKTAALS--------
APLFNGGRACGACYQLTC

VTSG--------TKYCYG-GGKSITITATNFCPT------GS--TGGWC-NPPKKHFDLSYPMFAVLA-------
KKVG-

GVIPVNFQRVPCS-RKGGMRFTI----NGNPY-FFLVMVYNVAGAGDVQRLFIK-GPST-G-WF--
EMKRNWGQVWQ-YN

GGPKGMVGQALSFRAITS-DGRQVISTNAAPGNWRFGQTFE-G---RNX

>CpEXPA17 [AB]

W--GQ----AHATYYGGAD--ASGTQ----G-GACGFG-NLYNTGYGTNTAALS--------
AALFNSGLSCGACYELTC

DPSG--------SQYCLP--GGSAIITATNFCPT------GS--NGGWC-NTPKQHFDLAQPVFSKIA-------
RTVG-

GVIPINYRRVSCS-KSGGMRFTI----NGNPY-FLLVLVTNVGGAGDVQQLYIK-GAST-N-WL--
PLKRNWGQMWQ-FT

GNSG-MHGQAISFKAVTG-DGATAVSDNVAPPNWGFGQTFE-G---ANF

>CpEXPA18 [B]

W-RTD----GHITFYGSPN--GGGTQ----A-GACGYQ-NTYALGYGSMTAALS--------
TALFQGGAACGGCFEIKC

IPPR------SGKNWCFS-YAKSIIVTATNLCPP------GS--TGGWC-NPPRAHFDLPMPAFLTLA-------
QKTG-

GVAPVAFRRVRCL-KKGGVRFTV----GGNPY-FLMVLISNVGGVGDIRSVKIK-GSYT-N-WI--
PMYRNWGSLWT-VR

SK---ITG-ALSFLITLS-DGSTLPCTNAVQANFRFGQTWE-AR--ANL

>CpEXPA19 [X]

W--KD---DGHITFYGSPN--GGGTQ----A-GACGYQ-NTYKLGYGSMTAALS--------
TPLFQGGLACGACFELRC

KFIR---ETKSAKNWCYN-YSRSIIITATNLCPP------GS--TGGWC-DPPRKHFDLPMPAFETLA-------
KRVG-

GVAPVYHRRVPCQ-KKGGIRFTM----AGNPW-FFMILIHNVGGAGDVVAVKIK-DPSS-A-WA--
PMYRNWGAIWT-VR

KQ---MKG-ALSFQITTG-DGRTVTTNNAVGNGWKFGQTWE-G---AQM

>CpEXPA20 [X]

W--KD---DGHITFYGSPN--GGGTQ----G-GACGYQ-NTVSLGYGYMTAALS--------
TTLFQGGAACGACFELKC

KFIK---ETNSAKNWCYS-YSKSIVITATNLCPP------GS--TGGWC-NPPRHHFDLPMPAFNTLA-------
KQVG-

GVAPVLFRRVPCQ-RKGGIRFTV----GGNPW-FFMILIHNVGGAGDITKVMIK-CPYT-P-WY--
PMYRNWGSLWT-VR

TQ---MRG-SLSFQITTS-DGRTVTTNNAVGNGWKFGQTWE-G---AQM

>CpEXPA21 [B]

W-RND----GHITFYGSPN--GGGTQ----A-GACGYQ-NTYALGYGSMTAALS--------
SSLFQGGAACGACFQLQC

IRAN------RGKNWCYS-YARTITVTATNLCPP------GS--TGGWC-NPPRAHFDLPMPAFLTLA-------
QRVG-

GVAPVRFRRVRCA-KRGGVRFTI----GGNPW-FMMILIHNVGGAGDIRAVQIK-GPRT-G-WV--
PMYRNWGALWT-CR

TK---ISG-ALTFRITTS-DGRTLVTGG-PGNSWRFGQTWE-G---RNY

>CpEXPA22 [X]

W--RD---DGHITYYGSPN--GHGTQ----G-GACGYQ-NTFSLGYDHLTAALS--------
TPLFQGGAACGACYELRC

KFIK---ESKTAKNXCWN-YSRSITSTATNLCPP------GS--TGGWC-DPPRNHFDLPMPAFLTLA-------
RQNG-

GVAPVHYRRVPCQ-KKGGIQFTI----GGNPW-FYMILIHNVAGAGDVVRVQIK-SPTT-G-WT--
PMYRNWGALWT-VR

KQ---LKG-ALSFQITTS-DGRVVVTHNAVGHGWRFGQTWE-G---AQY

>AaEXPA1PRO

W--QD----GHATFYGGSD--ASGTM----G-GACGYS-NLYSQGYGLATAALS--------
TALFNNGLSCGACFEIKC

NQPE----------WCVA-GASSIVVTATNLCPP-NPSLPND--NGGWC-NEPLQHFDMSQPAFQQIA------
-QYKA-

GIVPVSYRRVSCV-KKGGVHFTV----NGHSY-FNLVLVSNVGGAGDVHAVSIK-GSNT-G-WE--
PMSRNWGQNWQ-SN

SK---LDGQSLSFQVTTS-DGQTLTFYDVASDNWSFGQTFE-GD--SNF

>AaEXPA2PRO

W--SS----AHATFYGGND--ASGTL----G-GSCGYG-NLYDRGYGTSTAALS--------
TALYNNGASCGACFEIQC

TGSR----------WCKG--SAPITVTATNFCPQ-NPALPNN--AGGWC-NLPLQHFDMSVPAFLEIA-------
EQVG-

GIVPVNYRRVSCV-KKGGIHFTI----NGHAY-FNLVLITNVGGAGDVHAVSVK-GSKT-G-WE--
PMTRNWGQNWQ-CN

SY---LNGQSLSFQVTTS--GGTLISYDVTPAGWSFGQTFS-G---AQF

>AaEXPA3PRO

W--NS----AHATFYGGND--ASGTM----G-GACGYG-NLYSEGYGTNTAALS--------
TALFDNGLSCGACYEIAC

DSSG--------TKYCTG--SNSITITATNFCPE-NLALPSD--NGGWC-NQPRQHFDMAVPAFDQLA-------
AHVA-

GIVPIKYRRVSCV-RKGGVRFHL----NGHDY-FLLLLVDNVGGNGAVHSVSIK-AGNG-P-WK--
ALSQNWGQNWQ-CN

DY---ISGQTLSISITTE-DGKTLVSNNVAPAGWKFGETYA-G---SQY

>AaEXPA4PRO

WVKGV----AHATFYGGSD--ASETN----G-GACGYG-NLYSQGYGTNTAALS--------
SALFGGGETCGACYQLKC

TGG------------CFS-GAPKIVVTATNYCPE------GS--TGGWC-NAPRVHFDLSEPAFTRMA-------
AAVT-

GVVPVNYRRVKCV-KEGGIKFQI----NGHSY-FNLILITNVGGVGDVKEVYIQVGSGS-S-WK--
ACKRNWGQNWE-ND

DY---LDGKSFSIKVITS-DNRFCNSPNVIPAGWKFGTTYE-G---RQF

>AaEXPA5PRO

W--KA----AHATFYGGND--ASGTN----G-GACGYG-NIYSFGYGIANAALS--------
YALFNSGAQCGACYEIKC

YGSK----------WCLP-GNPSITITATNSCPP-NWALPSD--NGGWC-NPPREHFDMSVPAWTSLA-------
VYVG-

GIVPVNYRRVPCS-RKGGIRFTI----SGNPN-FDLVLVHNVAGDGVVTALSIK-GNGN---WV--
PMKRNWGQNWQ-CG

AV---LTGQPLSFMVRTS-DGRSLILYNVAPANWSFGSTYQ-SA--QNF

>AaEXPA6PRO

---ND----AHATFYGDPS--GSATF----G-GACGYG-DLMAQGYGLQTTALS--------
EVLFSNGFSCGQCYQIQC

IDSK----------WCIP--NAVITVTATNLCPA-DYSKPND--NGGWC-NPPLKHFDLSMPAFHQIA-------
QEVA-

GIVPVTYRRVPCV-KLGGVKFTI----LGNTW-WHLVLITNVGGAGDIDTASIK-GSNT-D-WI--
PMSHNWGASFQ-CF

QS---LVNQALSFQLTST-TGQVLTTFNVAPADWSFGTTYE-G---GQY

>AaEXPA7PRO

W--TT---GATATYYGGSD--ATATN----G-GACGYG-NQIAL-YGTVSTALS--------
SAFFQNGVTCGACYEIQC

TGSS----------YCYS--GKTVKVTATNLCPD------GS--YGGWC-DGSKKHFDLSQPAFIQVA-------
QQVV-

GHFAAQYRRVPCT-RSGGVRFTI----NGNTY-YNLVLIQNVAGVGDVKAVSIK-GTST-G-WL--
SMSRNWGENWE-CD

TK---LVGQALSFQVTLS-DSTTKTFNNIAASNWAFGSTYS-SS--VQF

>AtEXPA7PRO

W--RY----AHATFYGDET--GGETM----G-GACGYG-NLFNSGYGLSTAALS--------
TTLFNDGYGCGQCFQITC

SKSP----------HCYS--GKSTVVTATNLCPP-NWYQDSN--AGGWC-NPPRTHFDMAKPAFMKLA-------
YWRA-

GIIPVAYRRVPCQ-RSGGMRFQF----QGNSY-WLLIFVMNVGGAGDIKSMAVK-GSRT-N-WI--
SMSHNWGASYQ-AF

SS---LYGQSLSFRVTSYTTGETIYAWNVAPANWSGGKTYK-ST--ANF

>AtEXPA8PRO

W--QG----GHATFYGGED--ASGTM----G-GACGYG-NLYGQGYGTNTAALS--------TALFNNGLTCGACYEMKC

NDDP---------RWCLG---STITVTATNFCPP-NPGLSND--NGGWC-NPPLQHFDLAEPAFLQIA-------QYRA-

GIVPVSFRRVPCM-KKGGIRFTI----NGHSY-FNLVLISNVGGAGDVHAVSIK-GSKT-QSWQ--AMSRNWGQNWQ-SN

SY---MNDQSLSFQVTTS-DGRTLVSNDVAPSNWQFGQTYQ-G---GQF

>AtEXPA11PRO

L--TN----GHATFYGGSD--ASGTM----G-GACGYG-DLYSAGYGTMTAALS--------TALFNDGASCGECYRITC

DHAA-------DSRWCLK--GASVVITATNFCPP-NFALPNN--NGGWC-NPPLKHFDMAQPAWEKIG-------IYRG-

GIVPVVFQRVSCY-KKGGVRFRI----NGRDY-FELVNIQNVGGAGSIKSVSIK-GSKT-G-WL--AMSRNWGANWQ-SN

AY---LDGQALSFSITTT-DGATRVFLNVVPSSWSFGQIYS-SN--VQF

>AtEXPA12PRO

W--IR----AHATYYGVND--SPASL----G-GACGYD-NPYHAGFGAHTAALS--------GELFRSGESCGGCYQVRC

DFPA-------DPKWCLR--GAAVTVTATNFCPT------NN--NNGWC-NLPRHHFDMSSPAFFRIA-------RRGNE

GIVPVFYRRVGCK-RRGGVRFTM----RGQGN-FNMVMISNVGGGGSVRSVAVR-GSKG-KTWL--QMTRNWGANWQ-SS

GD---LRGQRLSFKVTLT-DSKTQTFLNVVPSSWWFGQTFS-SRG-RQF

>AtEXPA13PRO

W--RP----ARATYYAATN--PRDAV----G-GACGYG-DLVKSGYGMATVGLS--------ETLFERGQICGACFELRC

VDDL---------RWCIP--GTSIILTATNFCAP-NYGFDPD--GGGHC-NPPNKHFVLPIEAFEKIA-------IWKA-

GNMPVQYRRINCR-KEGSMRFTV----DGGGI-FISVLITNVAGSGDIAAVKIK-GSRT-G-WL--PMGRNWGQNWH-IN

AD---LRNQALSFEVTSS-DRSTVTSYNVSPKNWNYGQTFE-G---KQF

>AtEXPA14PRO

W--VN----ARATFYGGAD--ASGTM----G-GACGYG-NLYSQGYGTNTAALS--------
TALFNGGQSCGACFQIKC

VDDP---------KWCIG---GTITVTGTNFCPP-NFAQANN--AGGWC-NPPQHHFDLAQPIFLRIA-------
QYKA-

GVVPVQYRRVACR-RKGGIRFTI----NGHSY-FNLVLITNVAGAGDVISVSIK-GTNT-R-WQ--
SMSRNWGQNWQ-SN

AK---LDGQALSFKVTTS-DGRTVISNNATPRNWSFGQTYT-G---KQF

>AtEXPA15PRO

W--VN----AHATFYGGSD--ASGTM----G-GACGYG-NLYSQGYGTNTAALS--------
TALFNNGLSCGACFEIKC

QSDG---------AWCLP---GAIIVTATNFCPP-NNALPNN--AGGWC-NPPLHHFDLSQPVFQRIA-------
QYKA-

GVVPVSYRRVPCM-RRGGIRFTI----NGHSY-FNLVLVTNVGGAGDVHSVAVK-GSRT-R-WQ--
QMSRNWGQNWQ-SN

NL---LNGQALSFKVTAS-DGRTVVSNNIAPASWSFGQTFT-G---RQF

>AtEXPA17PRO

W--LQ----AHATFYGGSD--ASGTM----G-GACGYG-NLYTDGYKTNTAALS--------
TALFNDGKSCGGCYQILC

DATK-------VPQWCLK--GKSITITATNFCPP-NFAQASD--NGGWC-NPPRPHFDMAQPAFLTIA-------
KYKA-

GIVPILYKKVGCR-RSGGMRFTI----NGRNY-FELVLISNVAGGGEISKVWIK-GSKS-NKWE--
TMSRNWGANYQ-SN

TY---LNGQSLSFKVQLS-DGSIKAALNVVPSNWRFGQSFK-SN--VNF

>AtEXPA20PRO

W--KI----ATATLSRDRD--GSSSVAT--G-GACGYG-DLRQSSFAGYSAGLS--------
GKLFNRGSSCGACLEVRC

VNHI---------RWCLQ-GSPSVVVTATDFCPP-NSGLSSD--YGGWC-NFPKEHLELSHAAFTGIA-------
ETRA-

EMIPIQYRRVKCG-RRGGLRFSL----SGSSH-FFQVLISNVGLDGEVVGVKVK-GHTT-A-WI--
PMARNWGQNWH-SS

LD---LIGQSLSFEVTLK-GGKTIASYDVAPPYWRFGMTYQ-G---KQF

>AtEXPA22PRO

W--YD----ARATFYGDIH--GGDTQ-------PCGYG-NLFRQGYGLATAALS--------
TALFNDGYTCGACYEIMC

TRDP---------QWCLP---GSVKITATNFCPA-NYSKTTD----LWC-NPPQKHFDLSLAMFLKIA-------
KYKA-

GVVPVRYRRIPCS-KTGGVKFET----KGNPY-FLMVLIYNVGGAGDIKYVQVK-GNKT-G-WI--
TMKKNWGQNWT-TI

TV---LTGQGLSFRVTTS-DGITKDFWNVMPKNWGFGQTFD-GR--INF

>OsEXPA4PRO

W--QS----AHATFYGGGD--ASGTM----G-GACGYG-NLYSQGYGTNTAALS--------
TALFNDGAACGSCYELRC

DNAG---------SSCLP---GSITVTATNFCPP-NYGLPSD--DGGWC-NPPRPHFDMAEPAFLHIA-------
QYRA-

GIVPVSFRRVPCV-KKGGVRFTV----NGHSY-FNLVLVTNVAGAGDVRSVSIK-GSRT-G-WQ--
PMSRNWGQNWQ-SN

AF---LDGQSLSFQVTAS-DGRTVTSNNVAHPGWQFGQTFE-G---GQF

>OsEXPA5PRO

W--SS----AHATFYGGGD--ASGTM----G-GACGYG-NLYSQGYGTNTAALS--------
TALFNNGLSCGACFEVRC

DAGG------GGSHSCLP---GSVVVTATNFCPP-NNALPSD--DGGWC-NPPRAHFDMSQPVFQRIA----
---LFKA-

GIVPVSYRRVACQ-KKGGIRFTI----NGHSY-FNLVLVTNVGGAGDVHAVAVK-SERS-AAWQ--
ALSRNWGQNWQ-SA

AL---LDGQALSFRVTTG-DGRSVVSNNAVPRGWSFGQTFS-G---AQF

>OsEXPA7PRO

W--QS----AHATFYGGSD--ASGTM----G-GACGYG-NLYSQGYGVNNAALS--------
TALFNSGQSCGACFEIKC

VNQP-------GWEWCHP-GSPSILITATNFCPP-NYALPSD--NGGWC-NPPRPHFDLAMPMFLHIA-----
--EYRA-

GIVPVSYRRVPCR-KKGGVRFTI----NGFRY-FNLVLITNVAGAGDIVRASVK-GTST-G-WM--
PMSRNWGQNWQ-SN

SV---LVGQALSFRVTGS-DRRTSTSWNAAPAGWHFGQTFE-G---KNF

>OsEXPA10PRO

W--RS----AKASYYAA-D--PEDAI----G-GACGFG-DLGKHGYGMATVGLS--------TALFERGAACGGCYEVKC

VDDL---------KYCLP--GTSIVVTATNFCAP-NFGLPAD--AGGVC-NPPNHHFLLPIQSFEKIA-------LWKA-

GVMPIQYRRVNCL-RDGGVRFAV----AGRSF-FLTVLISNVGGAGDVRSVKIK-GTES-G-WL--SMGRNWGQIWH-IN

SD---FRGQPLSFELTSS-DGKTLTNYNVVPKEWDFGKTYT-G---KQF

>OsEXPA11PRO

W--SS----GSATFYGGSD--ASGTM----G-GACGYG-NLYSAGYGTSTAALS--------TALFNNGQSCGACFEVRC

GGGG----------SCLA---GTVAVTATNLCPP-NYALAGD--AGGWC-NPPRPHFDMAEPAFTRIA-------QARA-

GVVPVQYRRVACA-KQGGIRFTI----TGHSY-FNLVLVTNVGGAGDVTAVSVK-GSRS-G-WQ--AMSHNWGANWQ-NG

AN---LDGQPLSFRVTAS-DGRTVTSDNVAPSGWSFGQTFS-G---GQF

>OsEXPA12PRO

A--LS----GTATFYGGSD--ASGTM----G-GACGYG-NLYSTGYGTNTAALS--------SALFNDGAACGECYQITC

DQSN--------SKWCKA--GTSVTITATNLCPP-DYSKPSN--DGGWC-NPPRQHFDMAQPAWEQIG-------VYRG-

GIVPVNFQRVSCT-RKGGVRFTI----NGNSY-FELVLITNVGGPGSIKSVQIK-GTKT-G-WV--TMSRNWGANWQ-AN

NY---LNNQAISFSVTST-AGKTLVFEDVAPSNWQFGQTFT-SG--VQF

>OsEXPA16PRO

W--RM----GSATYIKESLGHPLNDG----G-GACGYG-DLDIFRYGRYTAGVS--------GALFGRGSACGGCYEVRC

VNHV---------LWCLR-GSPTVVVTATDFCAP-NLGLSDD--YGGWC-NFPKEHFEMSEAAFLRVA-------KAKA-

DIVPVQFRRVSCD-RAGGMRFTI----TGGAS-FLQVLITNVAADGEVAAVKVK-GSRT-G-WI--PMGRNWGQNWQ-CD

AD---LRGQPLSFEVTGG-RGRTVVAYSVAPPDWMFAQTFE-G---KQF

>OsEXPA30PRO

W--TP----AHATFYGDET--ASETM----G-GACGYG-NLYASGYGTDTAALS--------
TTLFKDGYGCGTCYQMRC

VGTA----------SCYR-GSPAITVTATNLCPP-NWAEDPDRGGGGWC-NPPRAHFDLSKPAFMRMA-----
--DWRA-

GIVPVMYRRVPCA-RAGGLRFAL----QGNPY-WLLAYVMNVAGAGDVGDMWVKAGGGG-G-WV--
RMSHNWGASYQ-AF

AQ---LGGQALSFKVTSYTTGQTILAAGVTPASWCFGLTYQ-AR--VNF

>OsEXPA32PRO

W--KQ----AHATFYGGRD--GSGTL----D-GACGYK-DTSKEGYGVQTVAVS--------
TPLFGAGAGCGACYEVKC

VDSP---------DGCKV-GAAPLVVTATNLCPP-NPGQSND--NGGWC-NPPREHFDLSMPAFLQIA------
-QEKA-

GIVPISYRRVPCV-KVGGIRYTI----TGNPY-FNLVMVSNVGGAGDVAGLSVK-GNKRVK-WT--
PLKRNWGQEWQ-TS

EV---LTGESLTFRVMTG-DHRKATSWHVLPPDWQFGVTYQ-AT--KNF

>OsEXPA33PRO

W--TP----ATATFYGGSD--GAGTM----G-GACGYG-NLYNAGYGLNNAALS--------
SALFNDGAMCGACYTIAC

DTSQ--------STWCKP--GTSITITATNLCPP-NYAKKSD--AGGWC-NPPRKHFDMSQPAWTSIA-------
IYQA-

GIVPVNFKRVPCQ-KSGGIRFTI----SGRDY-FELVTVFNVGGSGVVAQVSIK-GSKT-D-WM--
AMSRNWGQNWQ-SN

AY---LNTQSLSFKVKLD-DAREVTVWNIAPSNWNFGTTYT-SN--INF

>SmEXPA1PRO

W--TS----AHATFYGGSD--AAGTM--V-G-GACGYG-NLYSQGYGNNNAALS--------
TALFNSGLSCGACFEIRC

DSAA-------DPRWCIA--GTSVVVTATNFCPP-NYALANN--NGGWC-NPPLEHFDMAQPAWEQIG----
---IYRG-

GIVPVQYRRVSCV-KKGGIHFTM----NGHTY-FNLVLISNVGGXGDVHAVSIK-GSGT-G-WQ--
DMSRNWGQNWQ-SN

XQ---FQGQSLSFKVTTS-DGKSVVSMDVAPADWQYGQTFE-G---SQF

>SmEXPA2PRO

W--TG----AHATFYGGSD--ASGTM----G-GACGYG-NLYSQGYGTNTAALS--------
TALFQSGLSCGACFEVKC

NGDP---------EWCLP--GSSVLVTATNFCPP-NXALPNN--NGGWC-NTPLQHFDMAQPAFEQIA------
-KYRG-

GIVPVLXRRVPCQ-RKGGIRFTM----XGHNY-FNLVLVTNVGGAGDVHSVSIK-GSNT-D-WL--
PMSRNWGQNWQ-SN

AI---LSGQSLSFKVTTS-DGRTVVSYDAAPPNWQYGQTYS-G---DQF

>SmEXPA3PRO

W--TD----AHATFYGGSD--ASGTM----G-GACGYG-NLYSQGYGXNTAALS--------
TXLFNSGLSCGACFEIKC

NAXK-------DPQWCRA--GASVTVTATNFCPP-NYAQAND--NXGWC-NPPLEHFDMAQPXWEQIG---
----IYRG-

GIVPVQYRRVSCV-KKGGIHFTL----NGNKY-FMLVLVSNVGGAGDVRAVSIK-GPSG-D-WQ--
PLSRNWGQNWQ-SD

SR---LIGQSLSFRVVTS-DNRAVTSLNVAPAGWSFGQTFS-G---EQF

>SmEXPA4PRO

W--TD----AHATFYGGSN--AAGTM----G-GACGYG-NLVSAGYGTNTAALS--------
TALFQDGLSCGACFEVKC

ASGS-------DPKWCLP---GSVVVTATNFCPP--SSQPSN--DGGWC-NSPLQHFDMAQPAFLKIA-------
QYSA-

GIVPISYRRVSCS-RSGGIRFTM----NGHAY-FNLVLITNVGGAGDVHAVSIK-GSGT-D-WI--
PMSRNWGQNWQ-SN

AL---LGGQALSFKVTTS-DGKTTIAYNVAGANWAYGQTFE-G---EQF

>SmEXPA5PRO

W--KQ----XFATFYGDET--ARETM----G-GACGYG-NLYQSGYGLMTAALS--------
STLFNSGYGCGQCYEITC

TLSK----------HCYF--GKSVVVTATNLCPP-NWSKPSN--NGGWC-NXPRVHFDMSKPAFMKIA-------
FWRA-

GIIPVSYR------RSGGMNFKL----XGNRW-WLMVFITNVGGSXDIKAVSVK-GSRT-G-WI--
AMTRNWGVGFQ-VF

KQ---LQGQSLSFMVTCYSTXKXXVHNNVAPANWQLGSTYS-A---KQL

>SmEXPA6PRO

W--RY----AHATFYGEYD--ALETM----G-GACGYG-NLYSQGYGTDTTALS--------
TVLFNSGYGCGGCYEISC

TQSK----------HCYP---GSTIVTATNLCPP-NWYKPSN--NGGWC-NPPRIHFDMSKPAFSKIA-------
YWRX-

GIVPVRYRRVPCX-RKGXIKFEL----KGNRW-WLIVFVSNVGGPGDIKRMAVK-GSKT-G-WL--
PMSRNWGVGFQ-VF

KS---LHGQSLSFMVTSFTTGKTVTAYDVVPANWRIGQAYS-G---GQM

>SmEXPA7PRO

W--LD---GAHATYYGGSD--ASGTN----S-GACGYG-NQLSAGYGYITTALS--------
TPLFENGDICGACFEIRC

AGGA----------GCLP-GNPSTVVTATNLCPX------GS--NXGWC-DPPKPHFDLSQPAFSRIA-------
SIXN-

GHVQLQYRRVACD-RQXGIRFTV----NGHTF-FNLVXVENVGGSGDVVAVEVK-GSAT-G-WR--
QMQRNWGQNWQ-DM

GD---LNGQALSXRVTGX-DGKVVTSMNVAPADWQFXRTYS-G---GQF

>SmEXPA8PRO

W--LD----AHATYYGGSD--ASGTN----S-GACGYG-NQLSAGYGYITTALS--------
TPLFXXGDICGACYEIRC

AGTG-----------XLP-RNPSTVVTATNLCPP------GS--NGGWC-DPPKQHFDLXQPAFSQIA-------
SIPY-

GHVLLQYRRVPCQ-RQGAIHYTI----NGXTF-FNLVLIENVGGSGDVVGVEIK-GSNT-N-WM--
PMARNWGQNWM-IG

GN---LGGQSLSFRVTGS-DGRKVTSLNVAPANWQFGRAYS-G---GQF

>SmEXPA9PRO

W--QD----AHATFYGGSD--ASGTM----G-GACGYG-NLYLQGYXVSXAALX--------
TPLFXEGWSCGSCFELKC

NAEA-------DPEWCLP-GNPSIVVTATNFCPP-NFALPSD--XGGWC-NPPREHFDLSQPAFELIA-------
KYRX-

GIVPVQYRRVPCE-XEGGIHFSI----NGHAY-FMLVLVWNVGGAGDVHAVAVM-GSRT-RRWQ--
PLVRNWGQNWQ-SP

XV---LLGQSLSFMVTTS-NGDTVTDYDVAPQDWKFGQTFV-G---NKN

>SmEXPA10PRO

W--TL----AHATYYGGSD--ASGTMGKX-S-GACGYG-NMYHEGFGVETTALS--------
TVLFQNGASCGACYELKC

HQDP---------KWCRP-GNLSITVTATNFCPP-NPARKSY--RGGWC-NXPQQHFDLSMPAFVHLA------
-NRTA-

GIIPVIYTRVECK-RQGGIRFTM----RGNKW-FILVMISNVGGAGDVRSVVVK-GSRS-P-WT--
PATRAWGQNWHISN

RS---MLEQGLSFVVSTS-DGESRIALDAVPQNWKFGQTFT-TA--AQF

>SmEXPA11PRO

W--ES----GHATFYGGSD--AAGTM----G-GACGYG-NLYSQGYGTNNAALS--------
SALYNNGLSCGACFEVKC

DAAA-------DPQWCIX--GRXVTVTATNFCPP-----------GSWC-NEPLKHFDMSQPAWEEIG-------
IYRG-

GIIPVYFRRVSCV-RKGGIXFTV----NGHAY-FNLILITNVGGAGDVHAVSVK-GSGT-G-WI--
PMSRNWGQNWQ-TN

AQ---LGGQSLSFMVTDS-SGKTVISNNAAPSNWQYGQTFE-G---EQF

>SmEXPA12PRO

W--SN----AHATFYGGSD--ASGTM----G-GACGYG-NVLSAGYGVNTAALS--------
TALFNGGATCGACFQMQC

VNSR----------WCRP--GKSVTVTATNFCPP-NNALXSD--NXGWC-NTPREHFDLSQPVWEQMA-----
--IYQG-

GIVPVQYRRVKCY-KQGGIIFTM----NXNPN-FNLVLIKNVAGWGDLRAVSIK-GSNT-G-WL--
PMKRNWGSNWE-YH

GV---LVGQSLSFLLTPS-MGGSLISYDVXPRNWQFGQSYS-G---RQF

>SmEXPA13PRO

W--TD---GAHATYYGGSD--ASGT-------GACGYG-NQLSAGYGVLTTALS--------
APLFNDGHVCGACFEVKC

SWGD---------SGCLA-GNPSIVVTATNLCPQ------GS--NGGWC-DSPKQHFDLAQPAFALIA-------
VTLN-

GHVPIQYRRVSCK-RDGGLRFTI----NGHVY-FNLVLIENVGGTGDVSAVSIK-GSKT-G-WR--
PMTRNWGQNWQ-DG

GD---LTGQSXSFEVTTS-DGNXITAYDVAPDYWQFGQTFS-G---GQF

>SmEXPA14PRO

W--G-----AHATYYGGSD--ASGTN----N-GXCGYG-NQLSAGYGTITTALS--------
TPLFRGGNVCGXCYQVRC

WGDP----------ACLP-GNPSVVVTATNLCXP------GS--NGGWC-DPPKPHFDLSQPAFSRIA-------
RIPN-

GHAQIQYRRVKCQ-RQGGIRFTI----NGHTY-FNLVLVTNVGGMGDVVGVSIK-GSSS-G-WR--
SMSRNWGQNWE-EG

SN---LNGQALSFRVTTS-DGRTVTAYNVAPGDWQFGRTYT-GNTASQY

>PpEXPB1PRO

W--LG----GHATWYGDPY--GEGSS----G-GACGYT-QLTGTPIGSKIAAGN--------
APIFQEGKGCGQCYEVKC

NYPS-----------CSP---QGTRIVITDLCP-----------GGQYC-
STDQPAFDFSGAAITAMALPGRDGELRNI-

GLYDIQYKRVPCEYPNQNIAFKV--DAGSSKY-WFSFTVKYLGGPGDINTVEVK-CGKN-GYFQ--
YAQHSWGANWM-LI

NYSGVPFQFPLTIKITTKLNDHTVVAEDVIPDWFGPGVQYE-SN--VQI

>CbEXP8 [AB]

W--TE---GATLTFYGDAN--GGGTL----A-GACGYG-NLLATPYKLMIAAGN--------
PPMFEEGLGCGRCWEIRC

VGNP----------GCTA----TVTITMTDLCP---------------C-
GSDGVHFDMSQPAFHALASGPAEAEQLVTA

GKLQGQYRRVPCD-LGLGKAFTAIVRAATNPS-FLSILMRDIPGAGDIKTAECAPGGGS-T-FS--
PMTQDFGAMYN-FN

GK----WTSPVSLRLTLG-DGQILTGISCIPAAWKPTTTYT-CK-----

>CbEXP10 [H]

W--IN----GHSTFYDDFG------R----G-GACGYG-RIGSQLYRNRFAAGS--------
PRVYLGGLACGMCMEVKC

VNSP----------ACRP-G--SVRVTITDLCPP-KYP------NLRWC-TGGKIHLDMARGAFPVIA-------
NPGA-

GHVPIQFRSVPCA-PFRLLKFTL----RGNRWGWLEVTTLTIPGSGRVLRLEYAMKGGA---WT--
GMSRAFGAAWA-TK

GK---FLRFPISIRIAVFGKFCKLVAKDCIKGQFWNGQQLS-CS--YSV

>CbEXP11 [x]

W--TE---GATLTFYGDAN--GGGTL----V-GACGYG-NLLATPCKLMIAAGN--------PPMFEEGFGCGRCWEIRC

VGNP----------GCTA----TVTITMTHLCP---------------C-GSDTVHFDMSQPAFHALASGPAEAEQLVTA

GKLQGQYRRVLCD-LGLGKAFTAIVREATNPS-FLSILMRDIPGAGDIKTAECAPGGGS-T-FS--PMTQDFGAMYN-FN

GK----WTSPVSLGLTLG-DGQILTGISCIPAAWKPATTYT-----CKX

EXPB Tree

>SmEXPB1PRO

WEPATA-TW--YG--SPNGAGT-D---GGACGYGSLPNT-PY-GSDVGAGSPILFMNGIGCGTCFEVIYVKCVDGQ----

LCSPQP--VNVVITDECPGG--------YCSGGRTHFDLSGTAFGKMASGS--ANIQHLLAAGVLNVLYRRAPCIYKS-Q

GVVFQVADGS-TPFWFETVIRYLDGPGDXATVELQQFGSS--------AWQPMSQVWGA--NWCLNAGGGTPLRA-PFSI

RLTA--LQ---TGEKXIAHN-VIPANWA---PQHSYSTGVNF

>SmEXPB2PRO

WQLATA-TW--YG--SPSGAGT-D---GGACGYGELPNT-PY-GLDVGAGSPVLFKNGRGCGACYKV--VRCLQQQ----

LCSGKA--VTVVITDECPGG--------YCAFGRTHFDLSGTAFGRMAM-A--GRTNQLLGSGVXQVLYKRXDCNYGS-R

PMEFQVNEGS-TPFWLSILVRYAAGPGDLGHVELMQGSR---------VWQPMTQVWGA--TWCFNGG---PLRG-PFSF

RVTT--LS---TSETVVARN-VIPXNWA---SNTCYRSRVNF

>PpEXPB1PRO

WLGGHA-TW--YG--DPYGEGS-S---GGACGYTQLTGT-PI-GSKIAAGNAPIFQEGKGCGQCYE---VKCNYPS----

-CSPQG--TRIVITDLCPGGQ-------YCSTDQPAFDFSGAAITAMAL-P--GRDGELRNIGLYDIQYKRVPCEYPN-Q

NIAFKVDAGS-SKYWFSFTVKYLGGPGDINTVEVKCGKNG--------YFQYAQHSWGA--NWMLINYSGVPFQF-PLTI

KITT--KL---NDHTVVAED-VIPDWFG---PGVQYESNVQI

>PpEXPB2PRO

WVRARG-TW--YG--DPYGEGS-S---GGNCGYTKLWGT-PI-GPKIVAGSRSIYANGQGCGQCYQ---IRCVDPNGGPR

LCNPQG--TNVVVTDFCPGGT-------YCSTGENAFDMSGAAINAMAL-R--GREGQLRNRGLYNLLYKRVPCRYRG-T

NIEFRVDNGS-SPFWLSILIKYVGGPGDIGQVYIRMANWY--------KFQPMRHAWGA--NWMIPNYDGKPFRG-PMDI

RIVS--RL---NRHTVLARG-VIPAYFR---PGTSYRSRVQM

>PpEXPB3PRO

WLPGHA-TW--YG--DPYGEGS-S---GGACGYTELAGT-PY-GLSVGAGSAVIYQNGQGCGECYE---VKCTYPS----

-CKPTP--SRIVITDFCPGGT-------FCSTGEPAFDLSGMAMTNMAL-P--GRDQELRNLGLYEIQYRRVPCYYPN-Q

NVAFKVDPGS-TPFWLSFTIEYQGGPGDIESVAIRQAGSS--------EFQQMQHNWGA--NYMLICYSGKPFKG-PYDV

MITA--KL---NGHVLIAQD-AIPEYFQ---PGGIYESNVQL

>PpEXPB4PRO

WVPGHA-TW--YG--DPYGEGS-S---GGXCGYLQLTGT-PI-GNKIAAGNEAIFQKGKGXXQCYE---VKCNYPV----

-CRPEG--TRIVITDLCPGXQ-------FCSGGNPAFDLSGAAISAMAK-D--GQDGALRNIGLYDIQYKRVPCEYPG-Q

NIVFKVDAGS-SPFWLSFTVKYMGGPGDIESVSISQRDG---------SFIPAQHSWGA--NWMLINYSGAPFQG-PYSV

KINC--ML---NGHTVVAKD-VIPAGFA---PGQEYESNVQI

>PpEXPB5PRO

WMAGHA-TW--YG--EPLGEGS-S---GGACGYTKLADT-PY-GPKIAAGNDPIFQGGSGCGACFE---VKCNYPS----

-CKSEP--TRIIITDQCPGGT-------YCSTSQPAFDLSGAAISDMAV-S--GKDGELRNIGLYDILYKRVPCEYPN-Q

NIAFQVDAGS-SAFWLSLLVKYMGGPGDIESVEIRTTGSS--------SFQPAKHNWGA--SWMLINTSGQPFKG-PYDV

KIVS--KL---NGHTVIAEK-AIPEFFE---PGKLYESNVQM

>PpEXPB6PRO

WVRARG-TW--YG--DPYGEGS-S---GGNCGYTRLXGT-PI-GPKIVAGSRSIYXNGLGCGQCYQ---NQCVDPXXGXK

LXNSFX--TQXXXTDFCPGGX-------YCSTGENAFDMSGAAINAMAL-P--SREGQLRNRGLYNLLYKRVPCRYRG-T

XIXXRVDXXS-SAYWLSILIKYVGGPGDIGQVYIKMANWF--------AFQPMKHAWGA--NWMMPSYDGKPFKG-PMDI

KIVS--KL---NRHSVVARG-VIPGYFR---PGTTYNSRVQM

>AaEXPB1PRO

WKPATA-TW--YG--APDGAGT-D---GGACGYGNLVGT-PY-GKRVTAGSPVLFKGGLGCGACYQ---VKCMIPK----

LCAKKA--VRVVITDECPGG--------YCSGLNTHFDLSGTAFSGMAN-P--GLGSSLLNLGVLPMLYKRVPCVYR--R

PMKIEVAAGS-SPFWFSVLIKFVRGAGELKSVELQQGGTN--------YWYSMQHIWGA--NWSVNVGS--GLKG-PFTL

RLTC--LL---TGQQIVIRN-VIPGNWA---PATTYLASVNF

>AaEXPB2PRO

WLSCKA-TF--YG--PQDGDGT-D---AGNCGYGALASS-PY-GKNIAAGSPELYQDGLGCGACFQ---IKCTNDG----

-CKDEA--STVVITDQCPGGE-------WCGVGKPHFDLRGTAFANLAV-A--GRSSDLFTRGVLDLQYQRVACSYGA-Q

TVAFAMDANC-NPNWIAFVVEFQAGDGDLHSVELKDASS---------DWQAMSHNWGA--NWQFSG----SLTG-PISV

RLTS--NT---ANQVLVAQD-AIPANWK---PGQTYKSQDQF

>AaEXPB3PRO

WLSCKA-TF--YG--PQDGDGT-D---AGNCGYGALASS-PY-GKNIAAGSPELYQDGLGCGACFQ---IKCTNDG----

-CKDEA--STVVITDQCPGGE-------WCGVGKPHFDLSGTAFANLAV-A--GRSSDLFTRGVLDLQFQRVACSYGA-Q

TVAFAMDANC-NPNWIAFVVEFQAGDGDLHSVELKDVSS---------DWQAMSHNWGA--NWQFSG----SLTG-PISV

RLTS--NT---ANQVLVAQD-AIPADWT---PGQTYQSQDQF

>AaEXPB4PRO

WLSCKA-TF--YG--PQDGDGT-D---AGNCGYGALASS-PY-GKNIAAGSPELYQDGLGCGACFQ---IKCTNDG----

-CKDEA--STVVITDQCPGGE-------WCGVGKPHFDLSGTAFANLAV-A--GRSSDLFTRGVLDLQYQRVACSYGA-Q

TVAFAMDANC-NPKWIAFVVEFQAGDGDLLSVELKDASS---------DWQAMSHNWGA--NWQFSG----SLTG-PISV

RLTS--NT---ANQVLIAQD-AIPANWK---PGQTYKSKQQF

>AaEXPB5PRO

WLSCKA-TF--YG--PQDGDGT-D---AGNCGYGALASS-PY-GKNIAAGSPELYQDGLGCGACFQ---IKCTNDG----

-CKDEA--STVVITDQCPGGE-------WCGVGKPHFDLSGTAFANLAV-A--GRSSDLFTRGILDLQYQRVACSYGA-Q

TVAFAMDANC-NPNWIAFVVEFQAGDGDLHSVELKDASS---------DWQAMSHNWGA--NWQFSG----SLTG-PISV

RLTS--NT---ANQVLIAQD-AIPANWK---PGQTYKSQDQF

>AaEXPB6PRO

WLACKA-TW--YG--EQDGDGS-D---GGACGYGELSSK-PY-GKNIAAGSPVLYQDGLGCGSCYQ---VKCTSDP----

ACSGDP--VTVVLTDICPGGS-------LCGIGKPHFDLSGTAMGNMAA-A--GKGSDLRGLGVVDILYKRVPCSYPE-Q

NVTIAVDQGS-NPSWLELTVLYEGGPGDLSALEVKDATS---------QWQPMVHKWGA--NWGYDKAA--DLHG-ALSI

RLTS--GL---GSLPLVAQN-ALPADWA---PGQTYQTVAQF

>AaEXPB7PRO

WVACKA-TW--YG--EPDGDGS-DVNAGGACGYGELSSK-AY-GKSIAAGSPVLYQDGLGCGSCYQ---VKCTSNP----

ACSGDP--VTIVLTDICPGGS-------LCGVGKPHFDLSGTAIGNMAA-A--GKGSDLRGLGVVDILYKRVPCAYPE-Q

NVTIAVDQGS-NPSWLELTVLYEGGPGDLSALEVKVTSH---------SSNPALACRGS--QI---------VVC-VFAP

ELTTHAGL---GT----AQD-A-TSQWHRSGSRTLYIIPSRA

>AaEXPB8PRO

WLSCKA-TF--YG--PRDGDGT-D---AGNCGYGALASS-PY-GSNIFAGSPELYQDGLGCGDA-----
SRCTNDG----

-CNDTP--TTVVMTDQCPGGE-------WCGIGKPHFDLSGTAFGSLAA-A--
GRSSDLFTLGVLDIQYQRVPCSYST-Q

TVAFAMDANC-NPNWIAFVVEFQAGDGDLHSVELKQDATS--------DWQAMSHNWXA--NWQFSG--
--SLQG-PIPV

RLTS--NT---ANQVLVAKD-AIPADWT---PGQTYQFX---

>AtEXLA1PRO

LHRSKA-AY--FS--SASALSS------GACAYGSMATS-FF-AGHIAAAIPSIYKDGAGCGACFQ---
VRCKNPK----

LCSTKG--TIVMITD-------------LNKSNQTDLVLSSRAFRAMAK-
PIVGADKDLLKQGIVDIEYQRVPCDYGN-K

NMNVRVEEASKKPNYLEIKLLYQGGQTEVVSIDIAQVGSSP-------NWGYMTRSHGA--VWVTDK----
VPTG-AIQF

RFVVTGGY---DGKMIWSQS-VLPSNWE---AGKIYDAGVQI

>AtEXLA2PRO

LHSSKA-AY--FS--SASALSS------GACAYGSMATG-FF-AGHIAAALPSIYKDGSGCGACFQ---
VRCKNPT----

LCSSKG--TTVIVTD-------------LNKTNQTDLVLSSRAFRAMAK-
PVVGADRDLLKQGIVDIEYRRVPCDYGN-K

KMNVRVEESSKNPNYLAIKLLYQGGQTEVVAIYIAQVGSS--------HWSYMTRSHGA--VWVTDK----
VPNG-ALQF

RFVVTAGY---DGKMVWSQR-VLPANWE---AGKSYDAGVQI

>AtEXLA3PRO

LHRSKA-SY--FS--SASALSS------GACAYGPMATS-FF-AGHIAAAIPSIYKDGAGCGACFQ---
VRCKNPK----

LCNSKG--TIVMVTD-------------LNTSNQTDLVLSSRAFRAMAK-
PVVGVDKYLLKQGIVDVEYQRVPCNYGK-R

NLNVRVEEASKKPNYLAIKLLYQGGQTEVVGIDIAPVGSS--------QWSYMSRSHGA--VWATDK----
VPTG-ALQF

KFTVTGGY---DGKTVWSKR-VLPANWN---SGRIYDAGVQI

>AtEXLB1PRO

FVNSRA-TY--YG--SPDCKAN-P---RGHCGYGEFGRD-IN-NGEVSGVSWRLWNNGTGCGACYQ---
VRCKIPP----

HCSEEG--VYVVATD-------------SGEGDGTDFILSPKAYGRMAR-P--
GTENQLYSFGVVNVEYQRIPCRYAG-Y

NLVYKIHEKSYNPHYLAILVLYVGGVNDILAVEVWQEDCK--------EWRRMRRVFGA--VHDLQN----
PPRG-TLTL

RFLVYGSA---GINWIQSPN-AIPADWT---AGATYDSNILL

>AtEXPB2PRO

WSIAGS-TW--YG--NPTGYGS-D---GGACGYGNAVAQPPF-SKMVSAGGPSLFKSGKGCGACYQ---
VKCTSKS----

ACSKNP--VTVVITDECPG----------CVKESVHFDLSGTAFGAMAI-S--
GQDSQLRNVGELQILYKKVECNYIG-K

TVTFQVDKGS-NANSFAVLVAYVNGDGEIGRIELKQALDSD-------KWLSMSQSWGA--VWKLDVSS--
PLRA-PLSL

RVTS--LE---SGKTVVASN-VIPANWQ---PGAIYKSNVNF

>AtEXPB3PRO

WLPAVA-TW--YG--SPNGDGS-D---GGACGYGTLVDVKPL-HARVGAVNPILFKNGEGCGACYK---
VRCLDKS----

ICSRRA--VTVIITDECPG----------CSKTSTHFDLSGAVFGRLAI-A--
GESGPLRNRGLIPVIYRRTACKYRG-K

NIAFHVNEGS-TDFWLSLLVEFEDGEGDIGSMHIRQAGAR--------EWLEMKHVWGA--NWCIIGG---
PLKG-PFSI

KLTT--LS---AGKTLSATD-VVPRNWA---PKATYSSRLNF

>OsEXLA1PRO

VRRSRA-AY--YT--SSLTLTA------GSCGYGTAAAT-FNGGGFLAAAGPALYRGGVGCGACYQ---
VRCKDKK----

LCSNAG--ARVVVTD-------------RARTNRTGLVLSSPAFAAMAR-P--
GMAASLTELAAVDVEYKRVPCEYRH-R

SLSVRVDERSRGPNELTISFLYQGGQTDIVAVDVAQVGSS--------SWKFMTREHGP--SWSMAN----
APPG-PLQM

RLVVTGGY---DGKWVWADREVLPRRWR---AGEVYDTGVQI

>OsEXLA2PRO

VRRSKA-GF--RD--SSIALNA------GSCGYGSLAAS-FN-GGHLAAASPALFRGGVGCGACFQ---VRCKDGK----

LCSTAG--AKVVVTDE------------ARSTNRTDLVLSAAAYAAMAR-P--GMAAQLRTRRAVDVEYKRVPCEYAAGR

NLSIRVEEKSRPPRELSIRFLYQGGQTDIVAVDVATVGSS--------NWKFMTRDYGP--AWSTAQ----APAG-PLQF

RVVVTGGY---DGKWVWADGEVLPRRWT---AGRVYDAGVQI

>OsEXLA3PRO

VRNGKA-AY--SPSLSPLPPGG-----GGGCGYGAMAMEMELNGGFLAAGGPRQHRGGLGCGRCFQ---MRCRNAE----

VCSNAG--VRVVLTD-------------FHRSNSTDFLLGGPAFAGLAK-P--GMAHKLKKLDALSVEYRRIPCDYKD-K

NLSILVEEQSKRPNNLVIKFLYQGGQTDILAVDVAQVGSS--------DWRFMTRVYGP--VWSIDR----APNG-PLQF

RAVVTGGY---DGKWVWADREVLPANWQ---PGQVYDTGARI

>OsEXLA4PRO

WCTPRHSTVSILP--TPTHAAHLT---GGACGFGAA----PM-ELNVAAVTADLFRHGHACGACYQ---LRCRDRR----

LCGEDG--VKVVVADMAKQPEQ-EGEMNRTAGGSLQFRITEDAFAAMAKQG--VSAHELTRQRTLEVDFRRIPCEYRESR

RLAVRVEEASRNPTHLAIRFLYQGGQTDIAAVEIAQANATPPSSSYYSSWRYMTRRDGAPGVWTTSR----APVG-PLRL

RVVVTAGS---GGKWLRSDGEVLPADWR---PGEVYDTGLRV

>OsEXLB1PRO

FTVSRA-AY--YP--NSDIKGT-E---NGACEYGAFGAT-LN-NGDVSA-SASLYRDGVGCGACYQ---VRCTNPY----

YCSPNG--VTIVITD-------------SGASDGTDFILSQHAFTRMAQST--DAGTALLTLGVVGIEYRRVSCTYPN-K

NIVFKITESSNFPNYLEFEIWYQQGNQDIIAVQLCETVNL--------TCQLLSRTHGA--VWAAVS----PPSG-PLSI

RMLFSSGAPRGGDTWLVPTN-IVPQNWT---AGATYDSGVQV

>OsEXPB15PRO

WSNGGA-TW--YG--AANGAGS-D---GGACGYQGAVFQAPF-SSMIAAGSPSIYKSGLGCGSCYQ---VKCTGNS----

ACSGNP--VTVVLTDECPGG--------PCLSEPVHFDLSGTAFGAMAN-P--GQADQLRAAGVLQIQYNRVPCNWGG-V

KLTFVVDVGS-NPNYFAVLVKYENGDGDLSGVELMQTGAGA-------AWTQMQQSWGA--VWKLNAGS--ALQA-PFSI

RLTS---S---SGKTLVASN-VIPSGWK---PGMSYISTVNF

>OsEXPB16PRO

WHPATA-TW--YG--SADGDGS-D---GGACGYGTLVDVVPM-KTRVGAVSPVLFKGGEGCGACYK---VRCLDAS----

ICSRRA--VTVIVTDECPGG--------VCAFGRTHFDLSGAAFARLAV-A--GHGGQLQNRGEISVVYRRTACKYGG-K

NIAFHVNEGS-TTFWLSLLVEFEDGDGDIGSMQLKQANSA--------QWQDMKHIWGA--TWSLTPG---PLVG-PFSV

RLTT--LT---TRQTLSAQD-VIPKNWT---PKATYTSRLNF

>CpEXPB1 [X]

WMAGHA-TW--YG--EPNGEGS-S---GGACGYTKLTGT-PY-GPRIAAGNDAIFQGGKGCGQCYE---VKCSYAE----

-CRQEA--TRIVITDQCPGGE-------YCSKAEPAFDLSGAAISAMAL-P--DKDGALRNIGLYDILYKRVPCEFQG-Q

NIAFQVDQGS-TGFWLSFVVKFLGGPGDIDSVEIRHSGSS--------SFQPAKHNWGA--SWMLINTSGQAFKG-PYDI

RVVS--KL---NGHAVTAEK-VIPEFFE---PGKLYESNVQL

>CpEXPB2 [A]

WVFGHG-TW--YG--DPYGEGS-S---GGNCGYTKLAGT-PY-GPKIVAGSPAIYANGLGCGQCYQ---VKCTDSKNGPR

LCNPSG--TQVVVTDFCPGGT-------YCSTGEKAFDFSGAALNAMAL-P--GKDGQLRNRGLYNVLYKRVPCKYKG-Q

NIAFRVDPGS-SPYWLSVLIKYVGGPGDIGAVSIKMANSY--------KFMPMKHAWGA--NWMLQNYDGKPFKG-PMDI

KIVS--RL---NGHSVVAKG-VIPGYFR---PGTTYTSRVQM

>CpEXPB3 [A]

WVYGHG-TW--YG--DPYGEGS-S---GGNCGYTKLAGT-PY-GPKIVAGSPAIYANGLGCGQCYQ---IKCLDSKYGPR

LCNSWG--TQVVVTDFCPGGT-------YCSSGQKAFDFSGAALNAMAL-P--GKDGQLRNRGLYNVLYKRVPCKYKG-Q

NIAFRVDPGS-SPYWLSVLIKYVGGPGDIGAVYIKMANSY--------RFMPMKQAWGA--NWMLQNYDGKPFKG-PMDI

KIVS--RL---NGHSVVAKG-VIPGYFR---PGTTYTSRVQM

>CpEXPB4 [X]

WMAGHA-TW--YG--EPNGEGS-S---GGACGYTKLTGT-PY-GPRIAAGNDAIFQGGKGCGQCYE---VKCSYAE----

-CRQEA--TRIVITDQCPGGE-------YCSKAEPAFDLSGAAISAMAL-T--DKDGALRNIGLYDILYKRVPCEFQG-Q

NIAFQVDQGS-TGFWLSFVVKFLGGPGDIDSVEIRHSGSS--------SFQPAKHNWGA--SWMLINTSGQAFKG-PYDI

RVVS--KL---NGHAVTAEK-VIPEFFE---PGKLYESNVQL

>CpEXPB5 [A]

WMAGHA-TW--YG--DPYGEGS-S---GGACGYTQLTGT-PI-GSKIAAGNNPIFQGGKGCGQCYE---VKCNYPS----

-CNPEP--TRIVITDLCPGGT-------YCSTGNPAFDFSGAAITAMAL-P--GRDGELKKIGLYDIQYKRVPCEYQN-Q

NIAFKVDFGA-SAYWLSFTVKYLGGPGDIESVEIRQVGSNG-------SFQPCQHSWGA--NWMLINYTGQPFDG-PYDV

KITA--KL---NGHSVIAYR-AIPKWFQ---PGALYNSNVQF

>CpEXPB6 [ACnFn]

WIAGHA-TW--YG--DPYGEGS-N---GGACGYTELAGT-PY-GLSVAAGSAIIFQNGQGCGQCYD---VKCSYPS----

-CKQEP--TRVVITDSCPGGT-------YCSTGEPAFDLSGMAMSNMAL-P--GRDQELRNLGLYDIQYKRVPCYYPN-Q

NIAFKVDPGS-TPYWLSFTVKYQGGPGDIQNVEIRQAGSS--------EFQPLQHNWGA--NYMMICYSGQPFRG-PYDV

KITA--KL---NGHVLIAEH-AIPEYFQ---PGGLYESNVQL

>PpEXPA1PRO

WRKAHA-TF--YG--GADASGT-M---GGACGYGNLYST-GY-GVDSTALSTALFNNGAKCGACFA---IQCYRSQ----

YCVPGSPVITVTATNFCPPNHKGDGTPGWCNPPMRHFDLAQPSFTKIAK----------YRAGIVPVLFRRVPCEKKG--

GVRFTING---NKYFNLVLVHNVGGKGDVHAVDIKGSNT---------EWIPMKRNWGM--NWQTDA----VMTGQALSF

RVTTS------DGKTIVSMN-ATPSHWS---FGQTFEGG-QF

>CbEXP8 [AB]

WTEGATLTF--YG--DANGGGT-L---AGACGYGNLLAT-PY-KLMIAAGNPPMFEEGLGCGRCWE---IRCVGNP----

GCTAT---VTITMTDLCP-----------CGSDGVHFDMSQPAFHALASGP--AEAEQLVTAGKLQGQYRRVPCDLGLGK

AFTAIVRAAT-NPSFLSILMRDIPGAGDIKTAECAPGGGS--------TFSPMTQDFGA--MYNFNG----KWTS-PVSL

RLTLG------DGQILTGIS-CIPAAWK---PTTTYTCK---

>CbEXP10 [H]

WINGHS-TF--YD--D---FGR-----GGACGYGRIGSQ-LY-RNRFAAGSPRVYLGGLACGMCME---VKCVNSP----

ACRPGS--VRVTITDLCPPKYP-NLR--WCTGGKIHLDMARGAFPVIAN-P--GA-------GHVPIQFRSVPCAPF--R

LLKFTLRGN--RWGWLEVTTLTIPGSGRVLRLEYAMKGG---------AWTGMSRAFGA--AWATKGK---FLRF-PISI

RIAVFGKF-----CKLVAKD-CIKGQFW---NGQQLSCSYSV

>CbEXP11 [x]

WTEGATLTF--YG--DANGGGT-L---VGACGYGNLLAT-PC-KLMIAAGNPPMFEEGFGCGRCWE---IRCVGNP----

GCTAT---VTITMTHLCP-----------CGSDTVHFDMSQPAFHALASGP--AEAEQLVTAGKLQGQYRRVLCDLGLGK

AFTAIVREAT-NPSFLSILMRDIPGAGDIKTAECAPGGGS--------TFSPMTQDFGA--MYNFNG----KWTS-PVSL

GLTLG------DGQILTGIS-CIPAAWK---PATTYTCKX--

Algae Tree

>PpEXPA1PRO

WR------KA--HATFYGGADASGTM-G---GACGYGNLYS-TGYGVDSTALSTALFNNGAKCGACFA-IQCYRSQ----

------YCVPGSP-VITVTATNFCPPNHK-GDGTPGWCNPPMR-HFDLAQPSFTKIAK--------YRAGIVPVLF-RRV

PCEKKG-GVRFT--I--NGNKY-FN---LVLVHNVGGKGDVHAVDIK-GSNT-EWIPMKRNWGMNWQ--TD-AVMTGQAL

SFRVTTS-DGKTIVSMNATPSHWSFGQTF-EG-GQF

>PpEXPA6PRO

WR------NA--RSTFYGGMDAAGTM-S---GACGYGNLYA-SGYGVHTTALSSALFKNGMACGACFE-VQCGGKGK---

------PCKPGS---VVVTATNFCPPNPGQSANNGGWCNPPNE-HFDLSYPAFVKIAD--------PKAGAVPLQY-RRV

PCQKQG-GIRFT--I--NGNCN-FI---LVTITNVGGSGVVTAAYLK-GDKT-EWSPLSRNWGANWQ--CR-RNYCGQGI

SIKIVT--SDNKVSVTKLAKSDWCFGKTF-IG-KQV

>PpEXPA7PRO

WG------YA--HATYYXGADASGTQGG---GACGFGNLYS-TGYGTNTAALSAALFNSGLSCGSCYE-LACDPNGS--K

------YCLPGGP-TVTVTATNFCP-----HGSLGGWCDAPKQ-HFDLAHPMFVSLAR--------EVGGVIPIKY-RRV

PCVKSG-GMRFT--I--NGNPW-FL---LVLVTNVAGAGDVQHMYIK-GSNT-PWEPMSRNWGSMWQF-TGDSKMKGQAL

SFKAVTS-DGSVAVSMDAAPGNWQFGQTF-EG-VNF

>PpEXPA13PRO

WN------EA--RATFYGGSDAGGTT-G---GACGYGDLYS-TGYGTNTVATSSAIFDRGLACGACYQ-VKCAGSAS---

------ECQPGTP-AIQVTVTNFCPPNPSLPEGNGGWCNLPLH-HFDMAMPAFQQIAS--------YRVGIVPILY-RRA

SCVRTG-GIRFT--M--SGHKF-MN---LVLVTNVGGMGDVQTVFIQ-GSKT-KLVAMIRNFGQIWQ--SS-VNVSGQRL

SFMVMTS-DGESVVSRNVAPSDWAYGQTY-EG-SQF

>PpEXPA14PRO

WD------KG--HATYYGEGDARGTM-G---GACGYSNLYS-TGYGVNTAALSGPLFNGGATCGACYE-
LTCILNESK--

------WCYRGK--NIIVTATNFCP-----SGSTGGWCNPPQK-HFDLSEPMFTTLAN--------RVGGVIPVNF-
RRV

ACYKQG-GMRFT--I--NGNPY-FF---IVLVYNVAGAGDVQQVYIK-GPKT-
QWLQMYRNWGSQWTFNGGPNNIVGSAL

SFRVHTS-DGRQVISYXAAPANWWFGXTFSSG-AN-

>PpEXPA24PRO

WS------SG--RITYYGSPNGGGTQ-G---GACGYQNTVS-LGYGFMTAALSTPLFNGGAACGACYQ-
LQCAPVHETPK

NLLQRNWCWKVGR-RITITATNLCP-----PGSEGGWCDPPQH-HFDLPMPAFTALAK--------
REGGVVPIYY-RRV

RCAKQG-GIRFT--M--GGNPW-FL---MILIHNVAGAGDVVAVKIK-CPTS-DWCDMYRNWGAFWT--
VQ-KKMEG-PL

SFQITTX-DRRKVTTHNAVGHGWQFGQTW-EG-AQY

>AtEXPA8PRO

WQ------GG--HATFYGGEDASGTM-G---GACGYGNLYG-QGYGTNTAALSTALFNNGLTCGACYE-
MKCNDDPR---

------WCLGST---ITVTATNFCPPNPGLSNDNGGWCNPPLQ-HFDLAEPAFLQIAQ--------
YRAGIVPVSF-RRV

PCMKKG-GIRFT--I--NGHSY-FN---LVLISNVGGAGDVHAVSIK-GSKTQSWQAMSRNWGQNWQ--
SN-SYMNDQSL

SFQVTTS-DGRTLVSNDVAPSNWQFGQTY-QG-GQF

>AtEXPA14PRO

WV------NA--RATFYGGADASGTM-G---GACGYGNLYS-QGYGTNTAALSTALFNGGQSCGACFQ-
IKCVDDPK---

------WCIGGT---ITVTGTNFCPPNFAQANNAGGWCNPPQH-HFDLAQPIFLRIAQ--------
YKAGVVPVQY-RRV

ACRRKG-GIRFT--I--NGHSY-FN---LVLITNVAGAGDVISVSIK-GTNT-RWQSMSRNWGQNWQ--SN-
AKLDGQAL

SFKVTTS-DGRTVISNNATPRNWSFGQTY-TG-KQF

>AtEXPA15PRO

WV------NA--HATFYGGSDASGTM-G---GACGYGNLYS-QGYGTNTAALSTALFNNGLSCGACFE-IKCQSDGA---

------WCLPGA---IIVTATNFCPPNNALPNNAGGWCNPPLH-HFDLSQPVFQRIAQ--------YKAGVVPVSY-RRV

PCMRRG-GIRFT--I--NGHSY-FN---LVLVTNVGGAGDVHSVAVK-GSRT-RWQQMSRNWGQNWQ--SN-NLLNGQAL

SFKVTAS-DGRTVVSNNIAPASWSFGQTF-TG-RQF

>OsEXPA4PRO

WQ------SA--HATFYGGGDASGTM-G---GACGYGNLYS-QGYGTNTAALSTALFNDGAACGSCYE-LRCDNAGS---

------SCLPGS---ITVTATNFCPPNYGLPSDDGGWCNPPRP-HFDMAEPAFLHIAQ--------YRAGIVPVSF-RRV

PCVKKG-GVRFT--V--NGHSY-FN---LVLVTNVAGAGDVRSVSIK-GSRT-GWQPMSRNWGQNWQ--SN-AFLDGQSL

SFQVTAS-DGRTVTSNNVAHPGWQFGQTF-EG-GQF

>OsEXPA5PRO

WS------SA--HATFYGGGDASGTM-G---GACGYGNLYS-QGYGTNTAALSTALFNNGLSCGACFE-VRCDAGGGGSH

------SCLPGS---VVVTATNFCPPNNALPSDDGGWCNPPRA-HFDMSQPVFQRIAL--------FKAGIVPVSY-RRV

ACQKKG-GIRFT--I--NGHSY-FN---LVLVTNVGGAGDVHAVAVK-SERSAAWQALSRNWGQNWQ--SA-ALLDGQAL

SFRVTTG-DGRSVVSNNAVPRGWSFGQTF-SG-AQF

>OsEXPA11PRO

WS------SG--SATFYGGSDASGTM-G---GACGYGNLYS-AGYGTSTAALSTALFNNGQSCGACFE-VRCGGGG----

------SCLAGT---VAVTATNLCPPNYALAGDAGGWCNPPRP-HFDMAEPAFTRIAQ--------ARAGVVPVQY-RRV

ACAKQG-GIRFT--I--TGHSY-FN---LVLVTNVGGAGDVTAVSVK-GSRS-GWQAMSHNWGANWQ--NG-ANLDGQPL

SFRVTAS-DGRTVTSDNVAPSGWSFGQTF-SG-GQF

>MpEXPA17PRO

WEVTEFTRGA--RATYYGGNDASGTL-G---GACGYGNLFE-NGYGVETTALSTALFNDGLTCGGCFQ-IQCIASES--D

------WCLANGTVSITVTATNFCP-----PGSFGGWCDPPQP-HFDLSYPMFATLAQ--------PVGGVIPVQY-RRV

SCLKQG-GVRFQ--I--NGNNW-FF---LVLVMNVAGAGDVQAVAIK-GTRS-EWISMQRNWGQNWAS-DGPPELVGQAL

SFRVTLGLTKETLDFIDVAPADWQFGQTFEDD-SGL

>MpEXPA24PRO

AN------AIPATATFYGGNDASGTNTA---GSCGYPNVLT-ID-GPMNTALSGALYNGGEACGACFK-IQCVSIPFGNL

------QCLPGS---IVVTATNLCP-----QGSTGGWCDEPRS-HFDLAEPAFQHLAP--------PVAGVVNVEY-ERV

SCLRSG-GIRFL--I--QGHPY-FM---QVLVYNVGGMGDVTAVSVK-GSSS-DWIQMDRNWGQLWT--TG-TVLDGQAL

SFSVTTS-DGRTVASNGVADSDWQYGQTF-EG-NQF

>MpEXPA26PRO

FG------DA--TATFYGGSDASGTQ-G---GSCGYQNPFA-LGYGIKTAALSNSLLANNMNCGACFE-VKCKYDASAYT

----KKWCYPNSP-TIIVTATNQSP-----PGLNG------EH-HFDLTEPMFMKIAN--------RIGGRIPIQY-RRV

RCYKPG-GIKFT--L--TGNPF-FN---LVLVYNVGGAGNVYGMAIK-GSRT-DFYPMSRNWGQYWQ--AF-VKLTGQSL

TFRVTLG-NGKSTTFWNVAPANWQFGQTVAAT-YNF

>MpEXPA30PRO

WT------DS--HATFNDGNNAQGTM-G---GACGYGNISS-RGYGLETTALSSTLFNSGLTCGACFE-IKCKVENST--

------WCFPNAG-SIKVTATDLCPANPAMPTNNGGWCNPPRT-HFDLSYQMFTRLAK--------AEGAIIPVQF-RRV

PCVKVG-GIRFT--M--NGNPW-FN---LVLVSNVAGAGNVVNLQMKSSETNWFWMTARQISGQYWE--LN-QNLMGQNI

SFRVTLG-NGKVMIANNVVPANWNFQQTFESD-INF

>MpEXPA36PRO

WG------DA--HATFYGGNDASGTM-G---GACGYGNLYS-LGYGTASTALSNGLFNNGLSCGACYT-IECKLEGS--K

------WCYPGK--SITVTATNACP-----PGSEGGWCDPPRL-HFDLSYPMFQTLAQ--------PVGGVIPVKY-KRV

SCNRQG-GIRFT--L--HGNAW-FN---YVLVHNVAGDGDVAGVSIK-GDNG-GWTTMYQNWGQFW---SASEHLVGQAL

SFRVTLG-SGRTQEFYDVASPNWYFGQTYEAD-NNQ

>AaEXPA2PRO

WS------SA--HATFYGGNDASGTL-G---GSCGYGNLYD-RGYGTSTAALSTALYNNGASCGACFE-IQCTGSR----

------WCKGSA--PITVTATNFCPQNPALPNNAGGWCNLPLQ-HFDMSVPAFLEIAE--------QVGGIVPVNY-RRV

SCVKKG-GIHFT--I--NGHAY-FN---LVLITNVGGAGDVHAVSVK-GSKT-GWEPMTRNWGQNWQ--CN-SYLNGQSL

SFQVTT--SGGTLISYDVTPAGWSFGQTF-SG-AQF

>AaEXPA4PRO

WV------KGVAHATFYGGSDASETN-G---GACGYGNLYS-QGYGTNTAALSSALFGGGETCGACYQ-LKCTG------

------GCFSGAP-KIVVTATNYCP-----EGSTGGWCNAPRV-HFDLSEPAFTRMAA--------AVTGVVPVNY-RRV

KCVKEG-GIKFQ--I--NGHSY-FN---LILITNVGGVGDVKEVYIQVGSGS-SWKACKRNWGQNWE--ND-DYLDGKSF

SIKVITS-DNRFCNSPNVIPAGWKFGTTY-EG-RQF

>AaEXPA6PRO

-N------DA--HATFYGDPSGSATF-G---GACGYGDLMA-QGYGLQTTALSEVLFSNGFSCGQCYQ-IQCIDSK----

------WCIPNA--VITVTATNLCPADYSKPNDNGGWCNPPLK-HFDLSMPAFHQIAQ--------EVAGIVPVTY-RRV

PCVKLG-GVKFT--I--LGNTW-WH---LVLITNVGGAGDIDTASIK-GSNT-DWIPMSHNWGASFQ--CF-QSLVNQAL

SFQLTST-TGQVLTTFNVAPADWSFGTTY-EG-GQY

>SmEXPA10PRO

WT------LA--HATYYGGSDASGTM-GKXSGACGYGNMYH-EGFGVETTALSTVLFQNGASCGACYE-LKCHQDP---K

------WCRPGNL-SITVTATNFCPPNPARKSYRGGWCNXPQQ-HFDLSMPAFVHLAN--------
RTAGIIPVIY-TRV

ECKRQG-GIRFT--M--RGNKW-FI---LVMISNVGGAGDVRSVVVK-GSRS-PWTPATRAWGQNWH--
ISNRSMLEQGL

SFVVSTS-DGESRIALDAVPQNWKFGQTFTTA-AQF

>SmEXPB2PRO

WQ------LA--TATWYGSPSGAGTD-G---GACGYGELPN-
TPYGLDVGAGSPVLFKNGRGCGACYKVVRCLQQQ----

------LCSGKA---VTVVITDECP---------GGYCAFGRT-HFDLSGTAFGRMAM-
AGRTNQLLGSGVXQVLY-KRX

DCNYGSRPMEFQ--VNEGSTPF-WL---SILVRYAAGPGDLGHVELMQGSRV--WQPMTQVWGATW---
CFNGGPLRGPF

SFRVTTLSTSETVVARNVIPXNWASNTCYRSR-VNF

>PpEXPB3PRO

WL------PG--HATWYGDPYGEGSS-G---GACGYTELAG-TPYGLSVGAGSAVIYQNGQGCGECYE-
VKCTYP-----

------SCKPTP---SRIVITDFCP--------GGTFCSTGEP-AFDLSGMAMTNMAL-PGRDQELRNLGLYEIQY-
RRV

PCYYPNQNVAFK--VDPGSTPF-WL---SFTIEYQGGPGDIESVAIR-
QAGSSEFQQMQHNWGANYMLICYSGKPFKGPY

DVMITAKLNGHVLIAQDAIPEYFQPGGIYESN-VQL

>AtEXPB2PRO

WS------IA--GSTWYGNPTGYGSD-G---GACGYGNAVAQPPFSKMVSAGGPSLFKSGKGCGACYQ-
VKCTSKS----

------ACSKNP---VTVVITDECPG-----------CVKESV-HFDLSGTAFGAMAI-SGQDSQLRNVGELQILY-
KKV

ECNYIGKTVTFQ--VDKGSNAN-SF---AVLVAYVNGDGEIGRIELKQALDSDKWLSMSQSWGAVWK--
LDVSSPLRAPL

SLRVTSLESGKTVVASNVIPANWQPGAIYKSN-VNF

>OsEXPB16PRO

WH------PA--TATWYGSADGDGSD-G---GACGYGTLVDVVPMKTRVGAVSPVLFKGGEGCGACYK-
VRCLDAS----

------ICSRRA---VTVIVTDECP---------GGVCAFGRT-HFDLSGAAFARLAV-AGHGGQLQNRGEISVVY-
RRT

ACKYGGKNIAFH--VNEGSTTF-WL---SLLVEFEDGDGDIGSMQLK-QANSAQWQDMKHIWGATWS--
LTPGPLVG-PF

SVRLTTLTTRQTLSAQDVIPKNWTPKATYTSR-LNF

>AaEXPB4PRO

WL------SC--KATFYGPQDGDGTD-A---GNCGYGALAS-SPYGKNIAAGSPELYQDGLGCGACFQ-
IKCTND-----

------GCKDEA---STVVITDQCP--------GGEWCGVGKP-HFDLSGTAFANLAV-
AGRSSDLFTRGVLDLQY-QRV

ACSYGAQTVAFA--MDANCNPK-WI---AFVVEFQAGDGDLLSVELK-DASS-DWQAMSHNWGANW--
-QFSGSLTG-PI

SVRLTSNTANQVLIAQDAIPANWKPGQTYKSK-QQF

>CbEXP1 [x]

WQ------DG--RATYYGSEAFGGDNEH--NGACGYGTLSA-AFVGHNYAAASPVIFESGRACGGCFE-
VQCRGNGY---

------PCRAGT---ARVTVTDLCPP----IPPNLRHCAGGKA-HLDLGVRVFPIIAD--------PRAGVVDIRF-
RTV

PCGRFN-AITFF--I--RGNVYGWL---SLVVMGVPASGRVVGMEVK-ASGSTNWEALRHDYGAAY---
MRTGRPLRTPI

SVRLSIFGKLCRMTAMNCIPTGFKDRQKVSCS-YRS

>CbEXP2 [H]

WI------NG--HSTFY---DDHGR--G---GACGYGALGS-QLYGTRFAAGSPRVYLGGLACGMCLE-
VRCVGSR----

------ACRRSS---VRLTITDLCPP----GYPNTQWCGGGKL-HLDIGKDAFPIIAD--------PRAGHVPIQF-
RSV

PCAPYK-VLKLN--L--RGHRW-WL---EVTALAIPGSGRVLKMEVA-GKGG-RWVSMKRAFGAAW---
STAGSTLQTPI

SVRISIFGKLCKLIAKDCINGQIYNGRQLSCA-YTV

>CbEXP3 [H]

WI------SG--HSTFY---DDHGR--G---GACGYGALGS-QLYGTRFAAGSPRVYLGGLACGMCLE-
VRCVGSR----

------ACRRSS---VRLTITDLCPP----GYPNTQWCGGGKL-HLDIGKDAFPIIAD--------PRAGHVPIQF-RSV

PCAPYK-VLKLN--L--RGHRW-WL---EVTVLAIPGSGRVLKMEVA-GKGG-RWVSLKRAFGAAW---STAGSTLQTPI

SVRISIFGKFCKLIAKDCIKGQIYNGRQLSCA-YTV

>CbEXP4 [H]

WI------NG--HSTFY---DDYGR--G---GACGYGRLGS-QLYGTRFAAGSPRVYLGGLACGMCLE-VRCVGSR----

------ACRRSS---VRLTITDLCPP----GYPNTQWC---QI-HLDIGKNAFPVIAD--------PRAGHVPIQF-RTV

PCAPYK-VLKLS--L--RGHRW-WL---EVTALAIPGSGRVLKMEVA-GRGG-RWVSLKRAFGAAW---ATAGATLQTPI

SVRISIFGKLCKLIAKDCIKGQIYNGRQLSCA-YFV

>CbEXP5 [H]

WL------NG--HSTFY---DDYGR--G---GACGYGALGG-QLFGTRFAAGSPSVYLGGLACGMCLE-VRCVGST----

------ACRRSS---VRLTITDLCPP----GYPNTQWCGGGKL-HLDIGKDAFPIIAD--------PRAGHVPIQF-RSV

PCAPYK-VLKLN--L--RGHRW-WL---EVTALAIPGSGRVLKMEVA-GKGG-PWVSMTRAFGAAW---STAGSTLQTPI

SVRISIFGKLCKLIAKDCIDGQIYNGRQLSCA-YTV

>CbEXP6 [H]

WI------NG--HSTFY---DDHGR--G---GACGYGPLGS-QLFANRFAAGSPRVYLGGLACGMCLE-VRCVGSR----

------ACRRSS---VRLTVTDLCPP----GYPNTQWCGGGKL-HLDISKNAFPIIAD--------PRAGHVPIQF-RSV

PCAPYR-ILRLN--L--RGHMW-WL---EVTALAIPGSGRILRMEVA-GRGG-RWRSMKRSFGAAW---STTGASLRLPI

SVRISILGKFCKLIARDCIKGQIYNGKQLSCR-YSV

>CbEXP7 [H]

WL------SG--KATFY----SGGDQ-G---NACGYGRLTG-AIFGRNFAAVSPRIYLGGLACGMCLE-VKCVGHP----

------GCRRGV---ARVTVTDLCPA----RPPNMAFCAGGKL-HLDLGRDVFPVICN--------PRVGVAPIRF-RAV

PCARYR-TLNII--L--NGNRYGYM---AIVVMSIPGSGRVIRMDVR-GRGGRNWEPLRRDWGATF---MRTGRPLRYPV

SIRVSILGKLCRLIARNCIKGQFFNGRKLSCS-YSA

>CbEXP8 [AB]

WT------EG-ATLTFYGDANGGGTL-A---GACGYGNLLA-TPYKLMIAAGNPPMFEEGLGCGRCWE-IRCVGNP----

------GCTAT----VTITMTDLCP------------CGSDGV-HFDMSQPAFHALASGPAEAEQLVTAGKLQGQY-RRV

PCDLGL-GKAFTAIVRAATNPS-FL---SILMRDIPGAGDIKTAECAPGGGS-TFSPMTQDFGAMY---NFNGKWTS-PV

SLRLTLG-DGQILTGISCIPAAWKPTTTYTCK----

>CbEXP9 [H]

WQ------SG--TGTFY-----SGGH-G---GACGFAGLGY-SIFAENYVAVSTALWNSGAACGTCLE-VRCKGPSS---

------ACIAGA--TIKVTVTDFCPN----TYPNLAHCGTDKH-HLDLGENVFPKIAA--------RSVGVIDLEY-RRV

PCVQAP-SITVS--V--EGNQYGYL---ALNLMRLPGAGTVAQLEIK-GANG-AYEQCRRDWGNTF---VRTGKPIEAPL

SLRVSVG-GICTFEAKDCIKS-LNFGSSVTCS-YNX

>CbEXP10 [H]

WI------NG--HSTFY---DDFGR--G---GACGYGRIGS-QLYRNRFAAGSPRVYLGGLACGMCME-VKCVNSP----

------ACRPGS---VRVTITDLCPP----KYPNLRWCTGGKI-HLDMARGAFPVIAN--------PGAGHVPIQF-RSV

PCAPFR-LLKFT--L--RGNRWGWL---EVTTLTIPGSGRVLRLEYA-MKGG-AWTGMSRAFGAAW---ATKGKFLRFPI

SIRIAVFGKFCKLVAKDCIKGQFWNGQQLSCS-YSV

>CbEXP11 [x]

WT------EG-ATLTFYGDANGGGTL-V---GACGYGNLLA-TPCKLMIAAGNPPMFEEGFGCGRCWE-IRCVGNP----

------GCTAT----VTITMTHLCP------------CGSDTV-HFDMSQPAFHALASGPAEAEQLVTAGKLQGQY-RRV

LCDLGL-GKAFTAIVREATNPS-FL---SILMRDIPGAGDIKTAECAPGGGS-TFSPMTQDFGAMY---NFNGKWTS-PV

SLGLTLG-DGQILTGISCIPAAWKPATTYTCK-X--

>CbEXP12 [H]

WT------GG--TATFY-----SGGR-G---GACGFGGLGY-AIYGERYAAASMKIWQNGAACGMCVE-IKCMGPS----

------GCYAGK--IVKVTITDFCPN----THPNLAHCGPEKN-HIDMGQNSFPVIAN--------PRVGVINIQY-RVV

PCNAYG-NFQVQ--I--NGNQYGWL---SLLIAQIPGSGRVVGCEVQ-GAGMNYWEGLRRVWGSNW---MRTGTPLKTPI

HVRLSLLGQICKVTSRNCIPNGFYFGQAVKCN-LRX

>CbEXP13 [H]

WT------SG--TATFY-----SGGS-G---GACGFGGLGY-AIYGERYAAASMRIWQNGAACGLCVE-IRCTGPS----

------GCYPGK--VVKVTITDFCPN----TPPNLAHCGPDKN-HIDMGQNSFPVIAD--------ARVGVIDIEY-RTV

SCTPYS-NFQVQ--I--NGNQFGWL---SLLIAQIPGSGRVVGCEVQ-GSGNDNWEALKRTWGSNW---VRTGTPLKAPI

HVRLTLWGQICKVTSHNCIPSGFSFGQVVQCN-LEX

>CbEXP14 [H]

WT------AG--TATFY-----SGGR-G---GACGFGGLGY-AIYGERYAAASMKIWQNGAACGMCVE-IKCMGPK----

------GCYAGK--VVKVTITDFCPN----TPPNLAHCGPEKN-HIDMGQNSFPVIAN--------PKVGVINTQY-RVV

PCNPYT-TFQVQ--V--NGNQFGWL---SLLIAQIPGSGRVVGCEVQ-GTGMNYWEGLKRTWGSNW---VRTGKPLKAPI

NVRLSLLGQICKVTSRNCIPSGFYFGQVVKCN-LRX

>CbEXP15 [x]

WI------SG--HATFY---DDHGR--G---GACGYGRLGS-ELFGGRYAAGSPRVYLGGMACGMCLE-VKCVGVP----

------ACKPAT---VRVTVTDLCPD----RPPNTQWCGGGKL-HLDMATAAFPVIAN--------PRAGHVPIQF-
RSV

PCAPYK-MLKLH--L--RGNMFGWL---EVTALGIPGSGKVLKMEVA-GSDG-SWVVMKRAFGAAW---
AVAGRPMRSPI

SVRIAIFGKLCKLVAKNCISGQNFNGVDLSCA-YSV

>CbEXP16 [H]

WI------NA--HATFY---DDGGR--G---GACGYGSLGS-QLFSNRFAAGSPAVYLGGLTCGMCVE-
VKCVESP----

------ACKAGS---VRLTITDLCPP----GAPNTQWCGGGKR-HLDLAKGAFPVIAN--------PGAGHVPIQF-
RTV

PCAPYN-VLTLN--L--RGHQW-WL---EVTAVGIPGSGRILRVEVA-GNGS-PWTVMKRAYGAAW---
ATAGSTLRSPI

SVRVSIFGNLCKLTARDCIKGPIYNGRKVTCG-YSV

>CbEXP17 [H]

WI------NA--HATFY---DDGGR--G---GACGYGSLGS-QLFSNRFAAGSPAVYLGGLTCGMCVE-
VKCVGSR----

------ACKAGS---VRVTITDLCPP----GAPNTQWCGGGKQRHLDLAKGAFPVVAN--------
PGAGHVPIQF-RTV

PCAPYK-VLTLN--L--RGHQW-WL---EVTAVGIPGSGRILRVEVA-GNGS-PWRVMKRAYGAAW---
ATAGSALRSPI

SVRISIFGNLCKLTARDCIKGPIYNGRKVT------

>CbEXP18 [H]

WI------NA--HATFY---DNGGR--G---GACGYGSLGS-QLFSNRFAAGSPAVYLGGLTCGMCVE-
VKCVGSR----

------ACKAGS---VRVTITDLCPP----GAPNTQWCGGGKR-HLDLAKGAFPVIAN--------
PGAGHVPIQF-RTV

PCAPYK-VLTLN--L--RGHQW-WL---EVTAVGIPGSGRILRVEVA-GNGS-PWRVMKRAYGAAW---
ATAGSTLRSPI

SVRISIFGNLCKLTARDCIKGPIYNGRKVTCG-YSV

>CbEXP19 [F]

WR------GG--TATCY-----TGGR-G---GACGFSGALA-LIYGQSYAAVSTRLWENGKVCGQCYE-
VRCVGSG----

------GCRRGS---VKVTVTDFCPD----TQANRRHCGATKQ-HIDLGRNAFPVIAN--------PSVGVINVQF-RTV

PCAAYH-LLRIS--V--RGNRNGWLSKGLFLTNENRKADRVFAVEVK-GRYRNYWERMTRTWGATY---DRRGRPLVGPI

SVRVSLFARFCKVVATNCIPAKWYSGSTYHCT-FRS

>CbEXP20 [B]

WS-----------AVLYICISA-----G---GACGYGPTLS-Q-YGPYLTALSVSLYQTGLACGGCFE-VKCVDSP----

------HCNSGK--VVKVTAVDECP--------EGGYCVTGKM-HFDLSQPAFKTIGN--------QQAGVIPILWRRRV

QCERNG-AIELH--M--QGGPY-WL---SILVKNTGGDGGVKSVQVKPSDKN-EWYQMTHVWGQKWDL-AGPMMPLKGPL

SIKLISLYSGQVATGWNCISQQWQSGQTYFIS-QQF

>CbEXP21 [AB]

WS------TG--RATFYGAPDGSGLD-G---GNCGFGPL-A-NSYGRYVAAISISLYQNGKGCGGCYE-VKCVDSQ----

------WCTGKS---VIISATDQCP--------DGGYCDNNQA-HFDMSHPAFEMIAQ--------KIAGVIPVSW-RRV

PCVRTG-PIQFV--I--NGNPF-WF---TIMPKNVAGAGDVKSVEVQIGG---RWVPMIQLWGQNWL--LSGMGQVNGAI

SVRVTTLLTGEVITVPNVIPSGWKSGTPYTSGMVNF

>CbEXP22 [x]

WI------NG--QATFY---DDHGR--G---GACGYGSLGS-QLFGGRYAAGSPRVYLGGLACGMCFE-VKCVRST----

------ACKAGS---VRVTITDLCPP----RAPNSRWCGGGKL-HLDMGRQAFPVIAN--------PRAGAVPIQF-RSI

PCAPYK-VLKLH--L--RGSRF-WL---EVTALAIPGSGRILKMEVA-AAGG-KWVAMKRAFGAAW---AVTGQPMRTPI

SVKISIFGKLCKLIARNCIKGQSFNGQNLNCG-YSV

>SpmEXP1 [ABF]

LVLADDTIDA--DTTYYGGANAGGAQ-A---GACGYGNLFT-AGYGTAITAVSTPLWKGGAACGACYE-LSCKPGTS---

------GCYNTK---LVVTVTDQCP-------DGGEWCQPDKP-HFDLSQPVFAQLAQ--------ISLGHFPVTA-RRV

TCPRTG-GVKVV--A--QGNSN-WL---LVKFMNVAGGGTVNAAWVK-GSNS-DWIPMSFNWGVGYQ--IT-GNIPQGPY

SFQLRDSYTGQVFTTDACLPSGWNVGQTYSCD-GNA

>SpmEXP2 [x]

WN------DA--HATFYGGADASGTM-G---GACGYGNLYS-SGYGTDTAAASSALFNDGMSCGQCYQ-LQCVGSS----

------ACKPGM---ITVTVTNLCPNDFSKPSDAGGWCNYPRQ-HFDLSQPAYQKIGV--------YGAGIVPIQY-RRV

PCLRTG-DIMFS--M--GGNPF-YL---DVTIKNVGGSGDLSAVWVK-GNSG-QWRQLSQNWGMQWS--AD-IAVQGQAL

SFRLQSQTTGQILTVENAVPSSWSPGSTY-QA-GQF

>SpmEXP3 [x]

WN------DA--HATFYGGADASGTM-G---GACGYGNLYS-SGYGTDTAAASSALFNDGMSCGQCYQ-LQCVSSS----

------VCKPGM---ITVTVTNLCPNDFSKPSDAGGWCNYPRQ-HFDLSQPAYEKIGV--------YGAGIVPIQY-RRV

PCLRTG-DIMFS--M--GGNPF-YL---DVTIKNVGGSGDLDAVWVK-GSSG-QWRQLSQNWGMQWS--AD-IAVQGQAL

SFRLRSQTTGQVLTVEDAVPSSWSPGSTY-QA-GQF

>SpmEXP4 [CBF]

WQ------PFNMTATFYGGPNAENT--G---GACGYGDMFT-AGFGTSTVAASSMLFKDGQSCGACYQ-VRCVNAV----

------GCIKDA--YIQITVTNYCPPRT--DMSTPGWCDGDNM-HFDMSQPAFTQIAE--------YSAGVVPVEV-MRV

PCTRGG-NIQWQ--V--GGNPN-WL---QLNVRNIAGDGDIAAISVK-GTNT-DWVTMTQSFGVTWQ--AD-GMFQGQAL

SFRVTSLLTGKVLQVDDAVPAGWTVGSVY-TA-GQY

>SpmEXP5 [x]

WK------TV--TASFYGGADASGNM-G---GACGYGNDVS-STYGLYTAAASTALYSEGLACGQCFQ-VQCANSK----

------ACIAGK--SVTVTVTNFCPNNWALPSNAGGWCNFPRQ-HLDMSQPAFTQIAQ--------YSAGIVPVQI-RRV

PCLRSG-SIAFG--L--GGNTF-WL---QVNIRNVGGSGDISSVQVK-GNGA--WLTMDHEWGQNWS--VD-AKLGGQSL

SFRLTTMTTGETLEIDNAAPSNWQVGKTY-QA-GQF

>SpmEXP6 [CBF]

WQ------PFNMTATFYGGPNAENT--G---GACGYGDMFT-AGFGTSTVAASSMLFKDGQSCGACYQ-VRCVNAV----

------GCIKDT--YIQITVTNYCPPRT--DMSTPGWCDGDNM-HFDMSQPAFTQIAD--------YSAGVVPVEV-MRV

PCTRDG-NIQWQ--V--GGNPN-WL---QLNIRNIAGDGDIAAISVK-GTNT-DWVTMTQSFGVTWQ--AD-GMFQGQPL

SFRVTSLLTGKVLQVDDAVPAGWTVGSVY-TA-GQY

>SpmEXP7 [ABF]

LGLADDTIDA--DTTYYGGANAGGAQ-A---GACGYGNLFS-AGYGTSITAVSTPLWKGGAACGACYE-LSCKPGTS---

------GCYNTK---LVVTVTDQCP-------DGGEWCQPDKA-HFDLSQPVFAQLAQ--------ISLGHFPVTA-RRV

SCPRSG-GVKVV--A--QGNSN-WL---LVKFMNVAGGGTINAAWVK-GSNS-DWIPMSFNWGVGYE--IT-GNIPQGPY

SFQLRDSYTGQVFTTDACLPSGWNVGQTYSCD-GNA

>SpmEXP8 [x]

WK------TV--TASFYGGADASGNM-G---GACGYGNDVS-STYGLYTAAASTALYSEGLACGQCFQ-VQCANSN----

------ACIVGK--TVTVTVTNFCPNNWALPSNAGGWCNFPRQ-HLDMSQPAFTQIAQ--------YSAGIVPVQV-RRV

PCLRSG-SITFG--L--GGNTF-WL---QVNIRNVGGSGDISSVQVK-GSGA--WLTMDHEWGQNWS--VD-AKLGGQSL

SFRLTTMTTGETLEVANAAPSSWQVGKTY-QA-GQF

>SpmEXP9 [x]

WN------DA--HATFYGGADASGTM-G---GACGYGNLYS-SGYGTDTAAASSALFNDGMSCGQCYQ-LQCVGSS----

------ACKPGM---ITVTVTNLCPNDFSKPSDAGGWCNYPRQ-HFDLSQPAYEKIGV--------YGAGIVPIQY-RRV

PCLRTG-DIMFS--M--GGNPF-YL---DVTIKNVGGSGDLDAVWVK-GSSG-QWRQLSQNWGMQWS--AD-IAVQGQAL

SFRLRSQTTGQILTVENAVPSSWKPGSTY-QA-GQF

>SpmEXP10 [ABF]

LVLADDTIDA--DTTYYGGANAGGAQ-A---GACGYGNLFS-AGYGTSITAVSTPLWKGGAACGACYE-LTCKPGTS---

------GCYNTK---LVVTVTDQCP-------DGGEWCQPDKP-HFDLSQPVFAQLAQ--------ISLGHFPVMA-RRV

SCPRSG-GVKVV--A--QGNSN-WL---LVKFMNVAGGGTINAAWVK-GSNS-DWIPMSFNWGVGYQ--IT-GNIPQGPY

SFQLRDSYTGQVFTTDACLPSGWNVGQTYSCD-GNA

>SpmEXP11 [CBF]

WQ------PFNMTATFYGGPNAENT--G---GACGYGDMFT-AGYGTSTVAASSMLFKDGQSCGACYQ-VRCVNAV----

------GCIKDT--YIQITVTNYCPPRT--DMSTPGWCDGDNM-HFDMSQPAFTQIAD--------YSAGVVPVEV-MRV

PCTRDG-NIQWQ--V--GGNPN-WL---QLNIRNIAGDGDIAAISVK-GTNT-DWVTMTQSFGVTWQ--AD-GMFQGQAL

SFRVTSLLTGKVLQVDDAVPAGWTVGSVY-TA-GQY

>SpmEXP12 [x]

WK------TV--TATFYGGADASGNM-G---GACGYGNDVS-STYGLYTAAASTALYSEGLACGQCFQ-VQCANSN----

------ACIAGK--TVTVTVTNFCPNNWALPSNAGGWCNFPRQ-HLDMSQPAFTQIAQ--------YSAGIVPVQV-RRV

PCLRSG-SIAFG--L--GGNTF-WL---QVNIRNVGGSGDISSVQVK-GSGA--WLAMDHEWGQNWS--VD-AKLGGQSL

SFRLTTMTTGETLEIDNAAPLSWQVGKTY-QA-GQF

Sequence Logo Alignment

>SpmEXP1 [ABF]

L--VLADDTID-ADTTYY--GGANAGGAQ----A-GACGY-GNLFT-AGYGTAITAVSTPLWKGGAACGACYELSCKP--

-------GTS----G-CYN-T--KLVVTVTDQCP---------DGGEWCQPDKP-HFDLSQPVFAQLAQ-I------SL-

GHFPVTA-RRVTCPRTG-GVKVV--AQGN--SN-WL---LVKFMNVAGGGTVNAAW--VKGSN--S-D-WI---PMSFNW

GVGYQ-ITGN---IPQGPYSFQLRD--SYTGQVFTTDACLPSGW--NVGQTYSCDGNASPI

>SpmEXP2 [x]

W--N------D-AHATFY--GGADASGTM----G-GACGY-GNLYS-SGYGTDTAAASSALFNDGMSCGQCYQLQCVG--

--------SS----A-CKP-G--MITVTVTNLCPNDFSKPSD--AGGWCNYPRQ-HFDLSQPAYQKIGV-Y------GA-

GIVPIQY-RRVPCLRTG-DIMFS--MGGN--PF-YL---DVTIKNVGGSGDLSAVW--VKGNS----GQWR---QLSQNW

GMQWS-ADIA---VQGQALSFRLQS--QTTGQILTVENAVPSSW--SPGSTYQAG----QF

>SpmEXP3 [x]

W--N------D-AHATFY--GGADASGTM----G-GACGY-GNLYS-SGYGTDTAAASSALFNDGMSCGQCYQLQCVS--

--------SS----V-CKP-G--MITVTVTNLCPNDFSKPSD--AGGWCNYPRQ-HFDLSQPAYEKIGV-Y------GA-

GIVPIQY-RRVPCLRTG-DIMFS--MGGN--PF-YL---DVTIKNVGGSGDLDAVW--VKGSS----GQWR---QLSQNW

GMQWS-ADIA---VQGQALSFRLRS--QTTGQVLTVEDAVPSSW--SPGSTYQAG----QF

>SpmEXP4 [CBF]

WQPF------N-MTATFY--GGPNAENT-----G-GACGY-GDMFT-AGFGTSTVAASSMLFKDGQSCGACYQVRCVN--

--------AV----G-CIK-DA-YIQITVTNYCPPRTDMSTP----GWCDGDNM-HFDMSQPAFTQIAE-Y------SA-

GVVPVEV-MRVPCTRGG-NIQWQ--VGGN--PN-WL---QLNVRNIAGDGDIAAIS--VKGTN--T-D-WV---TMTQSF

GVTWQ-ADGM---FQGQALSFRVTS--LLTGKVLQVDDAVPAGW--TVGSVYTAG----QY

>SpmEXP5 [x]

W--K------T-VTASFY--GGADASGNM----G-GACGY-GNDVS-STYGLYTAAASTALYSEGLACGQCFQVQCAN--

--------SK----A-CIA-GK-SVTVTVTNFCPNNWALPSN--AGGWCNFPRQ-HLDMSQPAFTQIAQ-Y------SA-

GIVPVQI-RRVPCLRSG-SIAFG--LGGN--TF-WL---QVNIRNVGGSGDISSVQ--VKGNG----A-WL---TMDHEW

GQNWS-VDAK---LGGQSLSFRLTT--MTTGETLEIDNAAPSNW--QVGKTYQAG----QF

>SpmEXP6 [CBF]

WQPF------N-MTATFY--GGPNAENT-----G-GACGY-GDMFT-AGFGTSTVAASSMLFKDGQSCGACYQVRCVN--

--------AV----G-CIK--DTYIQITVTNYCPPRTDMSTP----GWCDGDNM-HFDMSQPAFTQIAD-Y------SA-

GVVPVEV-MRVPCTRDG-NIQWQ--VGGN--PN-WL---QLNIRNIAGDGDIAAIS--VKGTN--T-D-WV---TMTQSF

GVTWQ-ADGM---FQGQPLSFRVTS--LLTGKVLQVDDAVPAGW--TVGSVYTAG----QY

>SpmEXP7 [ABF]

L--GLADDTID-ADTTYY--GGANAGGAQ----A-GACGY-GNLFS-AGYGTSITAVSTPLWKGGAACGACYELSCKP--

-------GTS----G-CYN-T--KLVVTVTDQCP---------DGGEWCQPDKA-HFDLSQPVFAQLAQ-I------SL-

GHFPVTA-RRVSCPRSG-GVKVV--AQGN--SN-WL---LVKFMNVAGGGTINAAW--VKGSN--S-D-WI---PMSFNW

GVGYE-ITGN---IPQGPYSFQLRD--SYTGQVFTTDACLPSGW--NVGQTYSCDGNASPI

>SpmEXP8 [x]

W--K------T-VTASFY--GGADASGNM----G-GACGY-GNDVS-STYGLYTAAASTALYSEGLACGQCFQVQCAN--

--------SN----A-CIV-GK-TVTVTVTNFCPNNWALPSN--AGGWCNFPRQ-HLDMSQPAFTQIAQ-Y------SA-

GIVPVQV-RRVPCLRSG-SITFG--LGGN--TF-WL---QVNIRNVGGSGDISSVQ--VKGSG----A-WL---TMDHEW

GQNWS-VDAK---LGGQSLSFRLTT--MTTGETLEVANAAPSSW--QVGKTYQAG----QF

>SpmEXP9 [x]

W--N------D-AHATFY--GGADASGTM----G-GACGY-GNLYS-SGYGTDTAAASSALFNDGMSCGQCYQLQCVG--

--------SS----A-CKP-G--MITVTVTNLCPNDFSKPSD--AGGWCNYPRQ-HFDLSQPAYEKIGV-Y------GA-

GIVPIQY-RRVPCLRTG-DIMFS--MGGN--PF-YL---DVTIKNVGGSGDLDAVW--VKGSS----GQWR---QLSQNW

GMQWS-ADIA---VQGQALSFRLRS--QTTGQILTVENAVPSSW--KPGSTYQAG----QF

>SpmEXP10 [ABF]

L--VLADDTID-ADTTYY--GGANAGGAQ----A-GACGY-GNLFS-
AGYGTSITAVSTPLWKGGAACGACYELTCKP--

-------GTS----G-CYN-T--KLVVTVTDQCP---------DGGEWCQPDKP-HFDLSQPVFAQLAQ-I------SL-

GHFPVMA-RRVSCPRSG-GVKVV--AQGN--SN-WL---LVKFMNVAGGGTINAAW--VKGSN--S-D-WI-
--PMSFNW

GVGYQ-ITGN---IPQGPYSFQLRD--SYTGQVFTTDACLPSGW--NVGQTYSCDGNASPI

>SpmEXP11 [CBF]

WQPF------N-MTATFY--GGPNAENT-----G-GACGY-GDMFT-
AGYGTSTVAASSMLFKDGQSCGACYQVRCVN--

--------AV----G-CIK--DTYIQITVTNYCPPRTDMSTP----GWCDGDNM-HFDMSQPAFTQIAD-Y------
SA-

GVVPVEV-MRVPCTRDG-NIQWQ--VGGN--PN-WL---QLNIRNIAGDGDIAAIS--VKGTN--T-D-WV---
TMTQSF

GVTWQ-ADGM---FQGQALSFRVTS--LLTGKVLQVDDAVPAGW--TVGSVYTAG----QY

>SpmEXP12 [x]

W--K------T-VTATFY--GGADASGNM----G-GACGY-GNDVS-
STYGLYTAAASTALYSEGLACGQCFQVQCAN--

--------SN----A-CIA-GK-TVTVTVTNFCPNNWALPSN--AGGWCNFPRQ-HLDMSQPAFTQIAQ-Y-----
-SA-

GIVPVQV-RRVPCLRSG-SIAFG--LGGN--TF-WL---QVNIRNVGGSGDISSVQ--VKGSG----A-WL---
AMDHEW

GQNWS-VDAK---LGGQSLSFRLTT--MTTGETLEIDNAAPLSW--QVGKTYQAG----QF

>CbEXP1 [x]

W--Q------D-GRATYYGSEAFGGDNEH----N-GACGY-GTLSA-
AFVGHNYAAASPVIFESGRACGGCFEVQCRG--

-------NGY----P-CRA-GT--ARVTVTDLCP-----PIPP-NLRHCAGGKA-HLDLGVRVFPIIAD-P------RA-

GVVDIRF-RTVPCGRFN-AITFF--IRGN--VYGWL---SLVVMGVPASGRVVGME--VKASG--STN-WE---
ALRHDY

GAAYM-RTGR---PLRTPISVRLSI--FGKLCRMTAMNCIPTGF--KDRQKVSCSY-----

>CbEXP10 [H]

W--I------N-GHSTFY-----DDFGR-----G-GACGY-GRIGS-
QLYRNRFAAGSPRVYLGGLACGMCMEVKCVN--

-------SPA------CRP-GS--VRVTITDLCP-----PKYP-NLRWCTGGKI-HLDMARGAFPVIAN-P------GA-

GHVPIQF-RSVPCAPFR-LLKFT--LRGN--RWGWL---EVTTLTIPGSGRVLRLEYAMKGGA------WT---
GMSRAF

GAAWA-TKGK---FLRFPISIRIAV--FGKFCKLVAKDCIKGQF--WNGQQLSCSY-----

>CbEXP11 [x]

W--T------EGATLTFY--GDANGGGTL----V-GACGY-GNLLA-
TPCKLMIAAGNPPMFEEGFGCGRCWEIRCVG--

--------NP----G-CTA----TVTITMTHLCP--------------CGSDTV-HFDMSQPAFHALASGPAEAEQLVTA

GKLQGQY-RRVLCDLGL-GKAFTAIVREATNPS-FL---SILMRDIPGAGDIKTAE-CAPGGG----STFS---
PMTQDF

GAMYN-FNGK----WTSPVSLGLTL--G-DGQILTGISCIPAAW--KPATTYTCKX-----

>CbEXP12 [H]

W--T------G-GTATFY-------SGGR----G-GACGF-GGLGY-
AIYGERYAAASMKIWQNGAACGMCVEIKCMG--

--------PS----G-CYA-GK-IVKVTITDFCP-----NTHP-NLAHCGPEKN-HIDMGQNSFPVIAN-P------RV-

GVINIQY-RVVPCNAYG-NFQVQ--INGN--QYGWL---SLLIAQIPGSGRVVGCE--VQGAG--MNY-WE--
-GLRRVW

GSNWM-RTGT---PLKTPIHVRLSL--LGQICKVTSRNCIPNGF--YFGQAVKCNL---RX

>CbEXP13 [H]

W--T------S-GTATFY-------SGGS----G-GACGF-GGLGY-
AIYGERYAAASMRIWQNGAACGLCVEIRCTG--

--------PS----G-CYP-GK-VVKVTITDFCP-----NTPP-NLAHCGPDKN-HIDMGQNSFPVIAD-A------
RV-

GVIDIEY-RTVSCTPYS-NFQVQ--INGN--QFGWL---SLLIAQIPGSGRVVGCE--VQGSG--NDN-WE---
ALKRTW

GSNWV-RTGT---PLKAPIHVRLTL--WGQICKVTSHNCIPSGF--SFGQVVQCNL---EX

>CbEXP14 [H]

W--T------A-GTATFY-------SGGR----G-GACGF-GGLGY-
AIYGERYAAASMKIWQNGAACGMCVEIKCMG--

--------PK----G-CYA-GK-VVKVTITDFCP-----NTPP-NLAHCGPEKN-HIDMGQNSFPVIAN-P------KV-

GVINTQY-RVVPCNPYT-TFQVQ--VNGN--QFGWL---SLLIAQIPGSGRVVGCE--VQGTG--MNY-WE--
-GLKRTW

GSNWV-RTGK---PLKAPINVRLSL--LGQICKVTSRNCIPSGF--YFGQVVKCNL---RX

>CbEXP15 [x]

W--I------S-GHATFY-----DDHGR-----G-GACGY-GRLGS-
ELFGGRYAAGSPRVYLGGMACGMCLEVKCVG--

-------VPA------CKP-AT--VRVTVTDLCP-----DRPP-NTQWCGGGKL-HLDMATAAFPVIAN-P------RA-

GHVPIQF-RSVPCAPYK-MLKLH--LRGN--MFGWL---EVTALGIPGSGKVLKME--VAGSD----GSWV---
VMKRAF

GAAWA-VAGR---PMRSPISVRIAI--FGKLCKLVAKNCISGQN--FNGVDLSCAY-----

>CbEXP16 [H]

W--I------N-AHATFY------DDGGR----G-GACGY-GSLGS-
QLFSNRFAAGSPAVYLGGLTCGMCVEVKCVE--

-------SPA------CKA-GS--VRLTITDLCP-----PGAP-NTQWCGGGKR-HLDLAKGAFPVIAN-P------GA-

GHVPIQF-RTVPCAPYN-VLTLN--LRGH--QW-WL---EVTAVGIPGSGRILRVE--VAGNG----SPWT---
VMKRAY

GAAWA-TAGS---TLRSPISVRVSI--FGNLCKLTARDCIKGPI--YNGRKVTCGY-----

>CbEXP17 [H]

W--I------N-AHATFY------DDGGR----G-GACGY-GSLGS-
QLFSNRFAAGSPAVYLGGLTCGMCVEVKCVG--

--------SR----A-CKA-G--SVRVTITDLCP-----PGAP-NTQWCGGGKQRHLDLAKGAFPVVAN-P------
GA-

GHVPIQF-RTVPCAPYK-VLTLN--LRGH--QW-WL---EVTAVGIPGSGRILRVE--VAGNG----SPWR---
VMKRAY

GAAWA-TAGS---ALRSPISVRISI--FGNLCKLTARDCIKGPI--YNGRKVT--------

>CbEXP18 [H]

W--I------N-AHATFY------DNGGR----G-GACGY-GSLGS-
QLFSNRFAAGSPAVYLGGLTCGMCVEVKCVG--

--------SR----A-CKA-GS--VRVTITDLCP-----PGAP-NTQWCGGGKR-HLDLAKGAFPVIAN-P------
GA-

GHVPIQF-RTVPCAPYK-VLTLN--LRGH--QW-WL---EVTAVGIPGSGRILRVE--VAGNG----SPWR---
VMKRAY

GAAWA-TAGS---TLRSPISVRISI--FGNLCKLTARDCIKGPI--YNGRKVTCGY-----

>CbEXP19 [F]

W--R------G-GTATCY-------TGGR----G-GACGF-SGALA-
LIYGQSYAAVSTRLWENGKVCGQCYEVRCVG--

-------S-G----G-CRR-GS--VKVTVTDFCP-----DTQA-NRRHCGATKQ-HIDLGRNAFPVIAN-P------SV-

GVINVQF-RTVPCAAYH-LLRIS--VRGN--RNGWLSKGLFLTNENRKADRVFAVE--VKGRY--RNY-WE--
-RMTRTW

GATYD-RRGR---PLVGPISVRVSL--FARFCKVVATNCIPAKW--YSGSTYHC-----TF

>CbEXP2 [H]

W--I------N-GHSTFY-----DDHGR-----G-GACGY-GALGS-
QLYGTRFAAGSPRVYLGGLACGMCLEVRCVG--

--------SR----A-CRR---SSVRLTITDLCP-----PGYP-NTQWCGGGKL-HLDIGKDAFPIIAD-P------RA-

GHVPIQF-RSVPCAPYK-VLKLN--LRGH--RW-WL---EVTALAIPGSGRVLKME--VAGKG----GRWV---
SMKRAF

GAAWS-TAGS---TLQTPISVRISI--FGKLCKLIAKDCINGQI--YNGRQLSCAY-----

>CbEXP20 [B]

W---------S---AVLY--ICISA--------G-GACGY-GPTLS--QYGPYLTALSVSLYQTGLACGGCFEVKCVD--

--------SP----H-CNS-GK-VVKVTAVDECP----------EGGYCVTGKM-HFDLSQPAFKTIGN-Q------QA-

GVIPILWRRRVQCERNG-AIELH--MQGG--PY-WL---SILVKNTGGDGGVKSVQ--VKPSD--KNE-WY--
-QMTHVW

GQKWD-LAGPMMPLKG-PLSIKLIS--LYSGQVATGWNCISQQW--QSGQTYFISQ---QF

>CbEXP21 [AB]

W--S------T-GRATFY--GAPDGSGLD----G-GNCGF-GPLAN--
SYGRYVAAISISLYQNGKGCGGCYEVKCVD--

--------SQ----W-C-T-GK-SVIISATDQCP----------DGGYCDNNQA-HFDMSHPAFEMIAQ-K------IA-

GVIPVSW-RRVPCVRTG-PIQFV--INGN--PF-WF---TIMPKNVAGAGDVKSVE--VQIGG----R-WV---
PMIQLW

GQNWL-LSGMG--QVNGAISVRVTT--LLTGEVITVPNVIPSGW--KSGTPYTSGMV--NF

>CbEXP22 [x]

W--I------N-GQATFY-----DDHGR-----G-GACGY-GSLGS-
QLFGGRYAAGSPRVYLGGLACGMCFEVKCVR--

--------ST----A-CKA-GS--VRVTITDLCP-----PRAP-NSRWCGGGKL-HLDMGRQAFPVIAN-P------
RA-

GAVPIQF-RSIPCAPYK-VLKLH--LRGS--RF-WL---EVTALAIPGSGRILKME--VAAAG----GKWV---
AMKRAF

GAAWA-VTGQ---PMRTPISVKISI--FGKLCKLIARNCIKGQS--FNGQNLNCGY-----

>CbEXP3 [H]

W--I------S-GHSTFY-----DDHGR-----G-GACGY-GALGS-
QLYGTRFAAGSPRVYLGGLACGMCLEVRCVG--

--------SR----A-CRR---SSVRLTITDLCP-----PGYP-NTQWCGGGKL-HLDIGKDAFPIIAD-P------RA-

GHVPIQF-RSVPCAPYK-VLKLN--LRGH--RW-WL---EVTVLAIPGSGRVLKME--VAGKG----GRWV---
SLKRAF

GAAWS-TAGS---TLQTPISVRISI--FGKFCKLIAKDCIKGQI--YNGRQLSCAY-----

>CbEXP4 [H]

W--I------N-GHSTFY-----DDYGR-----G-GACGY-GRLGS-
QLYGTRFAAGSPRVYLGGLACGMCLEVRCVG--

--------SR----A-CRR---SSVRLTITDLCP-----PGYP-NTQWC---QI-HLDIGKNAFPVIAD-P------RA-

GHVPIQF-RTVPCAPYK-VLKLS--LRGH--RW-WL---EVTALAIPGSGRVLKME--VAGRG----GRWV---
SLKRAF

GAAWA-TAGA---TLQTPISVRISI--FGKLCKLIAKDCIKGQI--YNGRQLSCAY----F

>CbEXP5 [H]

W--L------N-GHSTFY-----DDYGR-----G-GACGY-GALGG-
QLFGTRFAAGSPSVYLGGLACGMCLEVRCVG--

-------STA------CRR---SSVRLTITDLCP-----PGYP-NTQWCGGGKL-HLDIGKDAFPIIAD-P------RA-

GHVPIQF-RSVPCAPYK-VLKLN--LRGH--RW-WL---EVTALAIPGSGRVLKME--VAGKG----GPWV---
SMTRAF

GAAWS-TAGS---TLQTPISVRISI--FGKLCKLIAKDCIDGQI--YNGRQLSCAY-----

>CbEXP6 [H]

W--I------N-GHSTFY-----DDHGR-----G-GACGY-GPLGS-
QLFANRFAAGSPRVYLGGLACGMCLEVRCVG--

--------SR----A-CRR---SSVRLTVTDLCP-----PGYP-NTQWCGGGKL-HLDISKNAFPIIAD-P------RA-

GHVPIQF-RSVPCAPYR-ILRLN--LRGH--MW-WL---EVTALAIPGSGRILRME--VAGRG----GRWR---
SMKRSF

GAAWS-TTGA---SLRLPISVRISI--LGKFCKLIARDCIKGQI--YNGKQLSCRY-----

>CbEXP7 [H]

W--L------S-GKATFY------SGGDQ----G-NACGY-GRLTG-
AIFGRNFAAVSPRIYLGGLACGMCLEVKCVG--

--------HP----G-CRR-GV--ARVTVTDLCP-----ARPP-NMAFCAGGKL-HLDLGRDVFPVICN-P------
RV-

GVAPIRF-RAVPCARYR-TLNII--LNGN--RYGYM---AIVVMSIPGSGRVIRMD--VRGRG--GRN-WE---
PLRRDW

GATFM-RTGR---PLRYPVSIRVSI--LGKLCRLIARNCIKGQF--FNGRKLSCSY-----

>CbEXP8 [AB]

W--T------EGATLTFY--GDANGGGTL----A-GACGY-GNLLA-
TPYKLMIAAGNPPMFEEGLGCGRCWEIRCVG--

--------NP----G-CTA----TVTITMTDLCP-------------CGSDGV-HFDMSQPAFHALASGPAEAEQLVTA

GKLQGQY-RRVPCDLGL-GKAFTAIVRAATNPS-FL---SILMRDIPGAGDIKTAE-CAPGGG----STFS---
PMTQDF

GAMYN-FNGK----WTSPVSLRLTL--G-DGQILTGISCIPAAW--KPTTTYTCK------

>CbEXP9 [H]

W--Q------S-GTGTFY-------SGGH----G-GACGF-AGLGY-
SIFAENYVAVSTALWNSGAACGTCLEVRCKG--

-------PSS----A-CIA-GA-TIKVTVTDFCP-----NTYP-NLAHCGTDKH-HLDLGENVFPKIAA-R------SV-

GVIDLEY-RRVPCVQAP-SITVS--VEGN--QYGYL---ALNLMRLPGAGTVAQLE--IKGAN----GAYE---
QCRRDW

GNTFV-RTGK---PIEAPLSLRVSV--G-GICTFEAKDCIKSL---NFGSSVTCSY-----

>CpEXPA1 [AB]

WIGG------N-AHATYY--GGVDASGTQ----G-GACGY-GNLYS-
TGYGTNTAALSSALFNSGLTCGACYELQCDS--

------TGSK----Y-CLP-GNPSITITATNYCP-----QNS--LGGWCDAPRQ-HFDLAHPMFVSLAQ-E------
GG-

GVIPVNY-RRVPCVKKG-GMRFQ--LNGN--PW-FV---LVLVTNVGGAGDVQQLY--IKGDK--T-G-WY---
PMKRNW

GQMWQ-LTGNSN-MPGQALSFRAVL--S-DGTNVESMDVAPANW--RFSQLFE-GS---QA

>CpEXPA2 [X]

---S------GTGHITFY--GTPNGGGQA----SRGACGY-QNTYA-
LGYGSMTAALSSPLYQGGAACGACFQLTCMR--

-----IRETKTAKNW-CWS-YSRSITVTATNLCP-----PGS--RDGWCNPPRN-HFDLPYPAYLSLAK-R------
EG-

GVAPVTW-RRVPCRKRG-GIRFT--VGGN--PW-FF---MILIHNVGGAGDVRSVK--IGCPY--T-G-WV---
PMYRNW

GALWT-VRRQ---MRGQ-LSFLITT--S-DGRSVVARNAVGKYW--KFGQTWECGA---QF

>CpEXPA3 [X]

SG-T--------GHITFY--GTPNGGGQA----SRGACGY-QNTYA-
LGYGSMTAALSSPLYQGGAACGACFQLTCMR--

-----IRETKTAKNW-CWS-YSRSITVTATNLCP-----PGS--RDGWCNPPRN-HFDLPYPAYLSLAK-R------
EG-

GVAPVTW-RRVPCRKRG-GIRFT--VGGN--PW-FF---MILIHNVGGAGDVRSVK--IGCPY--T-G-WV---
PMYRNW

GALWT-VRRQ---MRGQ-LSFLITT--S-DGRSVVARNAVGKYW--KFGQTWECGA---QF

>CpEXPA4 [AB]

W--G------K-GKCTFY--GGMDAAGTM----S-GACGY-GNLYA-
SGYGAYTAALSSALFKNGMACGACYEVACSG--

-------NAK----S-CKR-G--SVLVTITNFCPPNPGRSAN--NGGWCNGANQ-HFDLSYPAFAKIAD-P-----
-KA-

GVVDLNY-RRVPCQKKG-GIRFT--LNGN--CN-FN---IVTITNVGGSGVVTQAY--MKGDG--M-D-WA---
PMTRNW

GANWQ-CSRN---YCGKGISIKIVT----SDKKVSVSRLADKTW--AFGKTYI-GQ---QV

>CpEXPA5 [AB]

W--N------S-AHATYY--GGADASGTQGERRG-GACGF-GNLYS-
TGYGTSTAALSQALFNSGLTCGACFELACDS--

------SGSK----Y-CE--GAPSVVVTATNFCP-----SGS--NGGWCDQPKQ-HFDLAMPVFDKIAQ-E------
VG-

GVIPVKY-RRVPCQKKG-GMRFT--INGN--PY-FI---LVLVTNVAGAGDVQQIS--IKGDN--T-D-WF---
AMKRNW

GQQWQ-FSGNSA-LVGQALSFKVVT--S-DGAVATSMNAAPANW--QFSQTFE-GV---NY

>CpEXPA6 [B]

W-RG------G-GHITFY--GGPNGAGTQ----A-GACGY-QNTYK-
LGYGSMTAALSSPLFQGGAACGACFQLRCAP-V

RRSRT--ARN----W-CYS-YAKTITVTATNLCP-----SGS--TGGWCNPPRA-HFDLPMPAFLTLAR-R------
EG-

GVAPVFY-RRVRCAKRG-GIRFT--IGGN--PW-FL---MVLIHNVGGAGDVRSVR--IKGQY--T-G-WY---
PMYRNW

GALWT-VRTK---LSG-ALTFAVTT----SDRRTVITRSVGGNW--RFGQTWEGA----NF

>CpEXPA7 [B]

W--K------GGAHITFY--GSPNGGGTQ----A-GACGY-QNTYK-
LGYGSMTAALSSPLFRGGSACGACYQIRCAP-V

RRTRT--ARN----W-CYS-YARTITITATNLCP-----PGS--TGGWCNPPRA-HFDLPMPAFLTLAR-R------
EG-

GVAPVYY-RKVRCAKRG-GIRFT--IGGN--PW-FL---MVLIHNVGGAGDMRSVR--VKGQY--T-N-WY---
PMYRNW

GALWT-VRTK---LSG-ALSFAVTT--S-NGRTVVARNVVGKWW--KFGQTWEART---NF

>CpEXPA8 [X]

W--G------Y-AHITFY--GTPNGGGQA----SRGACGY-QNTYA-
LGYGAMTAALSSPLYKGGAACGACFELKCIR--

-----IRESRSAKNW-CWS-YSRSITITATNLCP-----PGS--TGGWCNPPKH-HFDLSYPAYATLAR-R------
EG-

GVAPVNY-RRVPCRKRG-GVRFT--VGGN--PW-FF---MILIHNIAGAGDVRSVK--IMCPY--T-G-WV---
PMYRNW

GALWT-VRKQ---MRG-PLSFAITT--S-DGRTIISRNAVGNYW--KFGQTWEGRA---QF

>CpEXPA9 [AB]

W--N------T-AHATFY--GGSDAGGTE----G-GACGY-GDLYS-
SGYDTNTVAISSALFDRGLACGACYQVKCAG--

------KASE------CHP-DSPSIQVTVTNFCPPNPSLPDN--NGGWCNLPLH-HFDMSEPAFQRIAT-Y-----
-KS-

AIVPVMY-RRTACMKSG-GIRFT--MSGH--NF-MN---LVLVTNVGGMGDVQSVY--IRGSK--T-K-WV---
AMARNF

GQIWQ-STVN---MSGQSLSFMVTT--S-DGQTVVSNEVAPPDW--AFGQTYE-GS---QF

>CpEXPA10 [AB]

W--R------K-AHATFY--GGADASGTM----G-GACGY-GNLHS-
TGYGLDTTALSTALFNNGAKCGACFAIQCYR--

--------SK----Y-CLP-GTPIITVTATNFCPPNRQGDG---TPGWCNPPLR-HFDLAQPSFVKVAK-Y------
RA-

GIVPVLF-RRVPCQKKG-GVRFT--LNGN--KY-FN---LVLVHNVGGKGDVRSVD--IKGSD----TEWM---
PMKRNW

GMNWQ-THTV---MVGKALSFRVTT--S-DGVTVTSMDAAPPQW--RFGQTFE-GA---QF

>CpEXPA11 [AB]

W--S------P-AHATFY--GGADASGTQ----G-GACGY-ENLYN-
QGYGINTAAVSAPLFNDGAGCGACFELVCDS--

------SQSD----Y-CYS-NASSIIVTTTNFCP--KAPEGT--ENAWCDPPRI-HFDLSEPIFLTFAQ-K------VA-

GIVPVNY-RRVPCLKDG-GIKFT--INGH--KY-FF---LVLVTNVAGAGDIQELY--IKASN--T-D-WQ---
PLTRNW

GDNWE-LNQNTLNFVGQALSFKAVA--S-DKSEVVSMNVTPVDW--EFSQTFE-GS---NF

>CpEXPA12 [A]

WTSG------Q-AHATYY--GGADAAGTQ----G-GACGF-GNLWS-
TGYGTDTAALSAALFNSGLSCGACYELKCDT--

------AGSK----Y-CLP-GNPSITVTATNYCP-----QGS--EGGWCDTPKQ-HFDLAHPAFTKLAH-E------
GG-

GVFPVVY-RRVPCAKQG-GMRFQ--INGN--PW-FL---LVLVTNVGGAGDVQQLQ--VKGER--T-G-WY--
-QMVRNW

GQMWQ-FTGDHT-LPGQALSFRATL--S-DGSVVESWNCAPKNW--GFQQVFE-GT---PA

>CpEXPA13 [X]

W-RD------D-GHITYY--GSPNGHGTQ----G-GACGY-QNTFS-
LGYDHLTAALSTPLFQGGAACGACYELRCKF--

-----IKESKTAKNW-CWN-YSRSITSTATNLCP-----PGS--TGGWCDPPRN-HFDLPMPAFLTLAR-Q-----
-NG-

GVAPVHY-RRVPCQKKG-GIQFT--IGGN--PW-FY---MILIHNVAGAGDVVRVQ--IKSPT--T-G-WT---
PMYRNW

GALWT-VRKQ---LKG-ALSFQITT--S-DGRVVVTHNAVGHGW--RFGQTWE-GA---QY

>CpEXPA14 [AB]

L--G------R-AHATYY--GGPDGQGTM----G-GACGY-GNLYN-
TGYGLSTAALSAPLYNGGRACGACYRLTCDT--

------SGSK----Y-CYG-GGKSITITVTNFCP-----SGS--TGGWCNPPKQ-HFDLSYPMFTTLAQ-A------AG-

GVIPVNY-RRVACNKKG-GMRFT--ISGN--PY-FY---QVLVFNVGGAGDVQRLF--MKGPR--T-G-WI---AMKRNW

GQVWQ-YRGGPKGLVGQPLSFRAHT--S-DGRYVISPNATPANW--RFGQTFE-GK---NF

>CpEXPA15 [AB]

W--G------Y-AHATYY--GGADASGTQ----G-GACGF-GNLYS-TGYGTNTAALSAALFNTGLSCGSCYELACDP--

------NGSK----Y-CLP-GGRTVTVTATNFCP-----HGS--LGGWCDSPKQ-HFDLAHPMFVSLAK-E------VG-

GVIPIKY-RRVPCIKSG-GMRFT--LNGN--PW-FI---LVLVTNVAGAGDVQQLY--IKGSR--T-Q-WE---RMSRNW

GSMWQ-FTGNSN-MKGQALSFKAIT--S-DGSVAISTDAAPGNW--QFGQTFE-GS---NF

>CpEXPA16 [AB]

W--D------N-GHATFY--GGDNGAGTM----G-GACGY-GNLYN-TGYGLKTAALSAPLFNGGRACGACYQLTCVT--

------SGTK----Y-CYG-GGKSITITATNFCP-----TGS--TGGWCNPPKK-HFDLSYPMFAVLAK-K------VG-

GVIPVNF-QRVPCSRKG-GMRFT--INGN--PY-FF---LVMVYNVAGAGDVQRLF--IKGPS--T-G-WF---EMKRNW

GQVWQ-YNGGPKGMVGQALSFRAIT--S-DGRQVISTNAAPGNW--RFGQTFE-GR---NX

>CpEXPA17 [AB]

W--G------Q-AHATYY--GGADASGTQ----G-GACGF-GNLYN-TGYGTNTAALSAALFNSGLSCGACYELTCDP--

------SGSQ----Y-CLP-GG-SAIITATNFCP-----TGS--NGGWCNTPKQ-HFDLAQPVFSKIAR-T------VG-

GVIPINY-RRVSCSKSG-GMRFT--INGN--PY-FL---LVLVTNVGGAGDVQQLY--IKGAS--T-N-WL---PLKRNW

GQMWQ-FTGNSG-MHGQAISFKAVT--G-DGATAVSDNVAPPNW--GFGQTFE-GA---NF

>CpEXPA18 [B]

W-RT------D-GHITFY--GSPNGGGTQ----A-GACGY-QNTYA-LGYGSMTAALSTALFQGGAACGGCFEIKCIP--

--PRS--GKN----W-CFS-YAKSIIVTATNLCP-----PGS--TGGWCNPPRA-HFDLPMPAFLTLAQ-K------TG-

GVAPVAF-RRVRCLKKG-GVRFT--VGGN--PY-FL---MVLISNVGGVGDIRSVK--IKGSY--T-N-WI---PMYRNW

GSLWT-VRSK---ITG-ALSFLITL--S-DGSTLPCTNAVQANF--RFGQTWEARA---NL

>CpEXPA19 [X]

W-KD------D-GHITFY--GSPNGGGTQ----A-GACGY-QNTYK-LGYGSMTAALSTPLFQGGLACGACFELRCKF--

-----IRETKSAKNW-CYN-YSRSIIITATNLCP-----PGS--TGGWCDPPRK-HFDLPMPAFETLAK-R------VG-

GVAPVYH-RRVPCQKKG-GIRFT--MAGN--PW-FF---MILIHNVGGAGDVVAVK--IKDPS--S-A-WA---PMYRNW

GAIWT-VRKQ---MKG-ALSFQITT--G-DGRTVTTNNAVGNGW--KFGQTWEGAQM--RF

>CpEXPA20 [X]

W--K------DDGHITFY--GSPNGGGTQ----G-GACGY-QNTVS-LGYGYMTAALSTTLFQGGAACGACFELKCKF--

-----IKETNSAKNW-CYS-YSKSIVITATNLCP-----PGS--TGGWCNPPRH-HFDLPMPAFNTLAK-Q------VG-

GVAPVLF-RRVPCQRKG-GIRFT--VGGN--PW-FF---MILIHNVGGAGDITKVM--IKCPY--T-P-WY---PMYRNW

GSLWT-VRTQ---MRG-SLSFQITT--S-DGRTVTTNNAVGNGW--KFGQTWE-GA-QMTY

>CpEXPA21 [B]

W-RN------D-GHITFY--GSPNGGGTQ----A-GACGY-QNTYA-LGYGSMTAALSSSLFQGGAACGACFQLQCIR--

----ANRGKN----W-CYS-YARTITVTATNLCP-----PGS--TGGWCNPPRA-HFDLPMPAFLTLAQ-R------VG-

GVAPVRF-RRVRCAKRG-GVRFT--IGGN--PW-FM---MILIHNVGGAGDIRAVQ--IKGPR--T-G-WV---PMYRNW

GALWT-CRTK---ISG-ALTFRITT--S-DGRTLVT-GGPGNSW--RFGQTWE-GR---NY

>CpEXPA22 [X]

W-RD------D-GHITYY--GSPNGHGTQ----G-GACGY-QNTFS-LGYDHLTAALSTPLFQGGAACGACYELRCKF--

-----IKESKTAKNX-CWN-YSRSITSTATNLCP-----PGS--TGGWCDPPRN-HFDLPMPAFLTLAR-Q------NG-

GVAPVHY-RRVPCQKKG-GIQFT--IGGN--PW-FY---MILIHNVAGAGDVVRVQ--IKSPT--T-G-WT---
PMYRNW

GALWT-VRKQ---LKG-ALSFQITT--S-DGRVVVTHNAVGHGW--RFGQTWEGA----QY

>PpEXPA1PRO

W--R------K-AHATFY--GGADASGTM----G-GACGY-GNLYS-
TGYGVDSTALSTALFNNGAKCGACFAIQCYR--

--------SQ----Y-CVP-GSPVITVTATNFCPPNHKGDG---TPGWCNPPMR-HFDLAQPSFTKIAK-Y------
RA-

GIVPVLF-RRVPCEKKG-GVRFT--INGN--KY-FN---LVLVHNVGGKGDVHAVD--IKGSN----TEWI---
PMKRNW

GMNWQ-TDAV---MTGQALSFRVTT--S-DGKTIVSMNATPSHW--SFGQTFEGG----QF

>PpEXPA2PRO

W--K------E-AHITYY--GTANGGGTQ----G-GACGY-PNTFA-
MGYGVMTAALSYPLFQGGKSCGACYQLKCKW-L

APTRT--VHN----W-CWS-YSRTITITATNSCP-----PGS--HGGWCDW-KP-HFDLPMPAFMTLAR-R-----
-EG-

GVAPVYY-RKVRCAKRG-GIRFT--LGGN--PY-FM---MILIHNVGGAGDLRAVK--IKGRN----GYWV---
PMWQNW

GALWT-CKTK---LSG-ALSFQITT--G-DGRTVTVNRAVGDYW--KFGQTWE-GS---QF

>PpEXPA3PRO

WISG------V-AHATFY--GGVDAQGTQ----G-GACGY-GNLYS-
TGYGTSTTALSSALFNAGLSCGACFELKCDS--

------ANSK----Y-CLP-GDKSITVTATNYCP-----QGS--DGGWCDSPKQ-HFDLSHPMFTSLAQ-E------
VG-

GVIPVTY-RRAPCAKKG-GMRFT--INGN--PW-FV---MILVTNCGGAGDVQQLQ--IRGSD--T-P-WY---
PCVRNW

GQMWQ-MTSDPN-LPGKALSFRATL--S-DGSVAESLNAAPSNW--GWGQTFEGVA---TY

>PpEXPA4PRO

W--K------D-AHITYY--GSPNGGGTQ----G-GACGY-QNTYA-
LGYGSFTAALSAPLFQGGAACGGCYQLKCAP-V

RETRT--VHN----W-CWS-YSRSIVVTATNLCP-----PGS--HGGWCAW-RP-HFDLPMPAFTSLAK-Q----
--VG-

GVAPVFY-RRVRCAKRG-GVRFT--IGGN--PY-FL---MVLIHNVGGAGDIRSVR--IKGQY--S-G-WV---
TMFRNW

GSLWT-CRTK---LSG-PLSFMITT--S-DGRTLVSNRAVGSWW--KFGQTWE-GS---QF

>PpEXPA5PRO

W--G------R-AHATFY--GGADASGTQ----G-GACGY-GNLYS-
TGYGTNTAALSSSLFNSGLSCGACYELTCDP--

------SCSQ----Y-CLP-GG-SAIITATNFCP-----TGS--NGGWCNPPKQ-HFDLAQPVFSKIAR-T------VG-

GVIPINY-RRVPCSKSG-GMRFT--VNGN--PY-FL---LVLVTNVGGAGDVQQLY--IKGAS--T-G-WL---
PLKRNW

GQMWQ-FTGNSG-MHGQAISFKAVT--S-DGAEAISPNVAPANW--GFGQTFE-GS---NF

>PpEXPA6PRO

W--R------N-ARSTFY--GGMDAAGTM----S-GACGY-GNLYA-
SGYGVHTTALSSALFKNGMACGACFEVQCGG--

--KG-----K----P-CKP-G--SVVVTATNFCPPNPGQSAN--NGGWCNPPNE-HFDLSYPAFVKIAD-P-----
-KA-

GAVPLQY-RRVPCQKQG-GIRFT--INGN--CN-FI---LVTITNVGGSGVVTAAY--LKGDK--T-E-WS---
PLSRNW

GANWQ-CRRN---YCGQGISIKIVT----SDNKVSVTKLAKSDW--CFGKTFI-GK---QV

>PpEXPA7PRO

W--G------Y-AHATYY--XGADASGTQ----GGGACGF-GNLYS-
TGYGTNTAALSAALFNSGLSCGSCYELACDP--

------NGSK----Y-CLP-GGPTVTVTATNFCP-----HGS--LGGWCDAPKQ-HFDLAHPMFVSLAR-E------
VG-

GVIPIKY-RRVPCVKSG-GMRFT--INGN--PW-FL---LVLVTNVAGAGDVQHMY--IKGSN--T-P-WE---
PMSRNW

GSMWQ-FTGDSK-MKGQALSFKAVT--S-DGSVAVSMDAAPGNW--QFGQTFE-GV---NF

>PpEXPA8PRO

W--N------E-AHATFY--GGSDAGGTTGTR-G-GACGY-GDLYS-
TGYGTSTVAISSALFDRGLACGACYQVKCAG--

-------SSS----E-CRS-DSPAIQVTVTNFCPPNPSLPED--NGGWCNLPLH-HFDMSMPAFEQIAT-Y-----
-KA-

GIVPVMY-RRTSCVRTG-GIHFT--MSGH--NF-MN---LVLVTNVGGMGDVQSVS--IRGSK--T-S-WV---
TMTRNF

GQIWQ-STVN---MSGQSLSFMVTT--S-DGKTVVSNNVAPPDW--AFGQTYEGS----QF

>PpEXPA9PRO

W--T------D-AHATFY--GGADASGTQGGCAG-GACGF-GNLYS-
TGYGTNTAALSQALFNSGLTCGACFELVCDS--

------SGSR----Y-CVT--SSSVVVTATNFCP-----TGS--TGGWCDYPRQ-HFDLSQPVFTRIAQ-P------
VG-

GVVTLKY-RRVRCQKSG-GIRFT--ITGN--PY-FI---LVLVTNVGGAGDVQQLY--IKGSS--T-G-WN---
AMSRNW

GQLWEIRNAA---LMGQALSFRVVT--S-DRAEVASFDAVPANW--AFSQTFE-GS---NF

>PpEXPA10PRO

WVSG------R-AHATFY--GGADASGTQ----G-GACGY-GNLYS-
TGYGTSTAALSSALFNSGLSCGACYELTCDT--

------SGSK----Y-CLP-GNPSIILTATNYCP-----QNS--NGGWCDAPKQ-HFDLAHPMFVSLAE-E------
RG-

GVIPVNY-RRVPCAKKG-GMRFQ--MNGN--PW-FL---LVLVTNVGGAGDVQQLS--IKGSN--S-G-WY--
-QMKRNW

GQMWQ-LTGNSN-MPGQALSFRAVL--S-DGTTVESLDAAPANW--HFGQMFE-GS---QA

>PpEXPA11PRO

W--G------R-AHATYY--GGADASGTQ----G-GACGF-GNLYS-
SGYGTDTAALSSALFNSGLSCGACYELTCDP--

------SGSK----F-CIP-GG-SAIITVTNFCP-----TGS--NGGWCNPPKQ-HFDLAQPVFRKIAR-T------VG-

GVVPINY-RRVSCLKDG-GMRFT--VNGN--PY-FL---LVLVTNVGGAGDVQQLY--MKGSS--T-N-WQ---
PLKRNW

GQMWQ-FTGNSR-MHGQAISFKAVT--S-DGSVAVSNNVAPPNW--GFGQTF---------

>PpEXPA12PRO

W--N------D-AHATYY--GGADAS-------G-GACGF-GNLYS-
TGYGTSTAALSQALFNSGLTCGACFELACDP--

------SGSK----Y-CYK-GS-SIVVTATNFCP-----SGS--EGGWCDSPKQ-HFDLSQPVFNKIAQ-Q------
AG-

GVIPVKY-RRVPCRKSG-GMRFT--INGN--PY-FI---LVLVTNVGGAGDVQQLS--LKGSS--T-G-WY---
TMSRNW

GQQWE-FRGNSA-LVGQALSFRVVT--S-DGAEAVSYDAASENW--SFSQTFE-GI---NF

>PpEXPA13PRO

W--N------E-ARATFY--GGSDAGGTT----G-GACGY-GDLYS-
TGYGTNTVATSSAIFDRGLACGACYQVKCAG--

------SASE------CQP-GTPAIQVTVTNFCPPNPSLPEG--NGGWCNLPLH-HFDMAMPAFQQIAS-Y----
--RV-

GIVPILY-RRASCVRTG-GIRFT--MSGH--KF-MN---LVLVTNVGGMGDVQTVF--IQGSK--T-K-LV---
AMIRNF

GQIWQ-SSVN---VSGQRLSFMVMT--S-DGESVVSRNVAPSDW--AYGQTYE-GS---QF

>PpEXPA14PRO

W--D------K-GHATYY--GEGDARGTM----G-GACGY-SNLYS-
TGYGVNTAALSGPLFNGGATCGACYELTCIL--

------NESK----W-CYR-GK-NIIVTATNFCP-----SGS--TGGWCNPPQK-HFDLSEPMFTTLAN-R------
VG-

GVIPVNF-RRVACYKQG-GMRFT--INGN--PY-FF---IVLVYNVAGAGDVQQVY--IKGPK--T-Q-WL---
QMYRNW

GSQWT-FNGGPNNIVGSALSFRVHT--S-DGRQVISYXAAPANW--WFGXTFSSGA---N-

>PpEXPA15PRO

W--G------Y-AHATYY--GGADASGTQ----GGGACGF-GNLYS-
TGYGTNTAALSAALFNSGLSCGSCYELACDP--

------NGSK----Y-CLP-GGRTVTVTATNFCP-----HGS--LGGWCDSPKQ-HFDLAHPMFVTLAK-E------
VG-

GVIPIKF-RRVPCVKSG-GMHFT--INGN--PW-FL---LVLVTNVAGAGDLQQVY--IKGSN--T-P-WE---
PMSRNW

GSMWQ-FTGNSK-MKGQALSFKTIT--S-DGAVAISYDAAPNNW--QFGQTFE-GV---NF

>PpEXPA16PRO

W--D------T-AHATYY--GGRDAGGTM----G-GACGY-GNLYN-
TGYGVKTAALSAPLFKGGATCGACYELTCIL--

------SQSK----Y-CYQ-NK-KILITATNFCP-----TGS--TGGWCNPPRK-HFDLSEPMFTTLAN-R------VG-

GVIPVNF-RRVHCYKKG-GMRFT--INGN--PY-HM---MVLVYNVAGAGDVQQMF--IKSPT--T-G-WL---
QMSRNW

GQIWT-YKGGPRNIVGFALSFRVYT--S-DRRQVVSYNAAPANW--RFGQTFSSGA----N

>PpEXPA17PRO

W--G------R-AHATYY--GGADASGTQ----G-GACGF-GNLYS-
SGYGTSTAALSSSLFNSGLSCGACYELTCDP--

------SGSQ----Y-CLP-GG-SAIITVTNFCP-----TGS--NGGWCNPPRQ-HFDLAQPVFSKIAR-T------
VG-

GVIPINY-RRVSCLKSG-GMRFT--VNGN--PY-FL---LVLVTNVGGAGDVQQLY--IKGSS--T-A-WL---
PLKRNW

GQMWQ-FTGNSG-MHGQAISFKAVT--S-DGAEAISNNVASSNW--GFGQTFE-GS---NF

>PpEXPA18PRO

W--N------E-AHITYY--GTANGGGTQ----G-GACGY-PNTFA-
MGYGAMTAALSYPLFQGGKACGGCYQLRCKW-V

TPTRT--VHN----W-CWS-YSRTITVTATNSCP-----PGS--HGGWCNW-RP-HFDLPMPAFLTLAR-R-----
-EG-

GVAPVYY-RKVRXAKRG-GIRFT--IGGN--PY-FL---MILIHNVGGAGDLKAVK--VRGGN----GYWV---
PMWRNW

GALWT-CKTR---MSG-ALSFQITT--G-DGRTLTTYKAVGGYW--RFGQTWEGS----QF

>PpEXPA19PRO

W--G------D-AHITYY--GSPNGAGTE----G-GACGY-QNTYK-
LGYGSMTAALSSRLFQGGKACGGCYQLRCAP--

-----NRGRN----W-CWS-YARAIVVTATNLCP-----QGS--HGGWCDYPKS-HFDLPMPAFTSLAR-R-----
-EG-

GVAPVWY-RKVRCAKRG-GVRFT--IGGN--PW-FL---MVLIHNVGGAGDVVSVK--VKCPY--T-G-WV---
SAYRNW

GCLWT-VRTK---MTG-PLSFTLVT--S-DGRTLYSMNAVRNGW--KFGQTWE-GS---QF

>PpEXPA21PRO

X--R------D-AHITYY--GSPNGGGTE----G-GACGY-QNTYS-
LGYGFMTAALSSSLFQGGSSCGACYQIRCEP--

-----IRVTRTVKNW-CWS-YSRTITVTAINLCP-----PRS--SGGCCNPPLQ-HFDLPMPAFLSLAR-R------
EG-

GVAPVXY-TKVRSEKRG-GIRFT--MGGN--LW-FL---TILIHNVGGAGDVRSVR--IKSPH--S-G-LI---
SMYRNW

GSLWT-VRAR---MSG-ALFFMITT--S-HGRVLITRNAVGSGW--RFGQTWE-G----NF

>PpEXPA22PRO

W--Q------D-AHITYY--GSPNGGGTQ----G-GACGY-QNTVS-
LGYGFMTAALSSPLFQGGKACGACFQLQCAR--

-----VQETRTVKNW-CHD-YSKAITVTATNLCP-----PGS--EGTWCDPPRH-HFDLPMPAFLSLAR-Q----
--EG-

GVAPVYY-RRVQCLKKG-GIRFT--MGGN--PW-FL---MVLVHNVXGAGDVVXVK--VKCPS--S-G-WY---
DMYRNW

GALWT-VQKK---MVG-PLSFLLTT--G-DGRRLTAYNAVGNGW--TFGQTWE-GA---QY

>PpEXPA23PRO

W--I------N-GRITYY--GSPNGGGTQ----G-GACGY-QNTVS-
LGYGFMTAALSTTLFKGGAACGACYQLQCAPVS

ETPSGLLKRN----W-CWQ-VGRSILVTATNLCP-----PGS--SGGWCNPPQH-HFDLPMPAFLALAR-R--
----EG-

GVVPVYY-RKVNCARKG-GIRFT--VGGN--PW-FL---MILIHNVGGAGDVVAVK--VKCPT--S-G-WY---
DMYRNW

GALWT-VQKK---MNG-PLSFAITT--S-DGRTVTTYNAVGNGW--KFGQTWE-GA---QY

>PpEXPA24PRO

W--S------S-GRITYY--GSPNGGGTQ----G-GACGY-QNTVS-
LGYGFMTAALSTPLFNGGAACGACYQLQCAPVH

ETPKNLLQRN----W-CWK-VGRRITITATNLCP-----PGS--EGGWCDPPQH-HFDLPMPAFTALAK-R---
---EG-

GVVPIYY-RRVRCAKQG-GIRFT--MGGN--PW-FL---MILIHNVAGAGDVVAVK--IKCPT--S-D-WC---
DMYRNW

GAFWT-VQKK---MEG-PLSFQITT--X-DRRKVTTHNAVGHGW--QFGQTWEGA----QY

>PpEXPA25PRO

W--R------D-AHITYY--GSPNGGGTQ----G-GACAY-QNTFS-
LGYGAMTAALSSPLFEGGAACGACYQLQCKR--

-----VQETRTVKNW-CWS-YSRTITITATNLCP-----PGS--AGAWCDPPRH-HFDLTMPAFLTLAR-R------
EG-

GVAPVLY-RRVKCVKRG-GIRFT--IGGN--PW-FL---MILIHNVAGAGDVRAVR--IKTPS--T-D-WI---
PMYRNW

GALWT-VQRK---LSG-PLSFQITA--G-DRRQITINSAVGNAW--KFGQTWE-GH---NF

>PpEXPA26PRO

W--N------F-GRITYY--GSPNGGGTQ----G-GACGY-QNTFA-
LGYGTNTAALSSRLFQGGAACGACYQLRCIA--

--------PKWGKNW-CWN-YARSIVVTATNLCP-----SGS--NGGWC-PSQA-HFDXPMPAFTSLAR-K----
--EG-

GVTPIMY-RRVRCARRG-GIRFT--IGGN--PF-FL---MVLIHNVGGAGDVRAVS--IKGQY--T-G-WV---
GMYRNW

GSLWT-CTTK---IDG-ALTFRITT--G-DGKTLILYNAVRKGW--RFGQTWE-GS---QF

>PpEXPA27PRO

W--A------T-ATATFY--GGADAG-------G-GACGY-GNLYS-
TGYGASTTALSAPLXNGGSACGACYQLQCAR--

--------SN----H-CYA-GR-SITVTATNFCP-----TGS--EGGWCNPPRK-HFDLSMPMFTTLAR-Q------
VA-

GVVPVDY-RRVACNKKG-GQRFL--MTGN--PY-FI---MVLVYNVAGAGDVQRFF--VKGSM--T-G-WY---
ELRRNW

GQIWT-CTADSR-LKGQALSFRTQT--S-DGRQVVSIDAAPANW--NFGQTFSSGV---N-

>MpEXPA1PRO

W--R------N-AHATFY--GGSDASGTM----G-GACGY-GNLYS-
QGYGTDTTALSAALFDNGAKCGACFAITCYR--

--------SR----W-CLP-GKPILQVTATNFCPSNPNLPND--NGGWCNPPRR-HFDLAQPAFTQIAI-Y------
EG-

GIVPVLF-KRVPCKRSG-PIRYT--INGQ--RY-FN---LVLISNVGGVGDVSAVS--VKGAN--SNY-WE---
PMRRNW

GQNWQ-SDTI---LVGQSLSFQVTT--S-DGSTVTVLDAVASNW--EFGQTFEGG----QF

>MpEXPA2PRO

W--A------D-AHATFY--GGQDASGTM----G-GACGY-GNLYS-
VGYGTSSTALSNALFNNGLSCGACFQIVCRL--

------AESK----Y-CYP-GG-SVVVTATNACP-----PGS--EGGWCDPPKA-HFDLSYPAFQRLAQ-P------
VG-

GVIPVRY-KRVSCSRNG-GIRFT--IRGN--PY-FD---LVLVTNVGEFGAVQSLQ--IKGER--T-G-WT---
YMRQNW

GANWQ-ANVN---VQGQALAFKVTL--D-NGRSLEFYNVAPKNW--GFGQTFEADY---NF

>MpEXPA3PRO

W--T------P-SHATFY--GEYNGVETM----G-GACGY-GNLWW-
RGYGLETAALSDTLLNNGLTCGACFQLKCDL--

--DHGGYSTR----W-CYK-NVAPITVTATNHCPANWARPTN--NGGWCNPPRT-HFDLPLVMFNRMAQ-
P------VA-

GIIPIEY-RRVPCEKSG-GLKFY--LSGN--PW-FN---LVLVYNVAGAGAVTSMK--MRGSQ----TVWY---
SMKRNW

GQYWE-CPIK---FSNQRLSFQVTL--S-SGHVRVINNLTPTNW--YFGQTYEAAT---NF

>MpEXPA4PRO

W--T------P-SHATFY--GDDSGAATM----G-GACGY-GNLWW-
RGYGLATAALSDTLLNNGLTCGACFEIKCNI--

---TGGESGK----W-CIQ-SNPSIKITATNQCPPNFDRPTD--NGGWCNPPRT-HFDLSLNMFRRLAQ-T--
----QA-

GIIPISY-RRTRCIKTG-GIRFY--LGGN--AH-FN---LVLVYNVGGWGDVMKMQ--MRGTK--T-G-WI---
TMKRNW

GQNWE-SSIN---YTGQSLSFIVTL--G-NGQVRVIRNLTPTVW--YFKQTYEADT---NF

>MpEXPA5PRO

W--T------D-SHVTFY--GASDGIETM----G-GACGY-GNLWW-
RGYGLETAALSDTLLNDGLTCGACYQIKCNI--

--ASGGYSTK----W-CYP-SNPTIQVTATNRCPPNWDRPTN--AGGWCNPPRT-HFDLPLVMFERMAQ-
A------VA-

GIIPVKY-RRVPCVKSG-GIKFF--LGGN--PW-FN---LVLVYNVAGGGNVKNMQ--MRGDK----TVWY---
TMKRNW

GQFWE-CPIG---FRGQKLSFIVTL--S-NGQVRVINNLTTELW--YFGQTYEALT---NF

>MpEXPA6PRO

W--S------T-AHATFY--GGSDASGTN----N-GACGY-SNVFA-
LGYGTMTAALSAPLFNDGLSCGACFQIQCSG--

-------DAA------CYS---NSITVTATNLCP-----QGS--YGGWCDYPNA-HFDLSQPAFSQLAQ-T------VA-

GHVTVNY-QRVNCNRQG-GVRFQ--IDGH--TY-FM---QTLIYNVGGSGDVVAVQ--VKGSK--T-G-WI---
SMTRGW

GQNWQ-TGTV---LDGQALSFSVKT--S-DGKTLYHNNVADSNW--KYGQTFE-GA---QF

>MpEXPA7PRO

F--G------D-ATATFY--GGNDAAGTA----G-GSCGY-ENPFT-
RGYGVTTVALSNSLLSNNLNCGACFEVKCKY--

--TASAYTKK----W-CYN--NKSVVVTATNLSP-----PGL--NGEH-------HFDLTYPMFTKISN-M------IA-

GRIPIQY-RRVPCKKQG-GIKFT--LNGN--PY-FN---LVLVYNVGGAGNVYAMS--MKGSK--S-D-WY---
PMARNW

GQNWQ-AMVR---LTGQSLSFRVTL--G-NGKTQTFSNVAAADW--KFGQTYQASS---NF

>MpEXPA8PRO

W--T------K-AHATFY--GGDDARGTQ----G-GACGY-GNMYT-
RGYGVKTVALSYTVFNEGLTCGACFQIKCDL--

-----SMKPR----W-CYA-NGGIITVTATNSCPPNWARPTN--NGGWCNPPRV-HFDLSYPMFAQLAQ-K-
-----VA-

GIIPIQY-KRVKCVRSG-GIRFT--INGN--PY-FN---IVLVHNVGGYGNVVTLY--IKGTN----SPWF---
KMKQNW

GANWE-DHNK---LTNQKLSFKATL--G-TGETMIFKDITGTYW--SHGQTYEANN---NY

>MpEXPA9PRO

W--S------P-AHATFY--GGSDAQGTQ----G-GACGY-GNLYR-
IGYGINTAALSTALFANGLSCGACFELKCNS--

--------PR----W-CKS-GRNPIRITATNLCPQNHAHPNN--AGGWCNMPLQ-HFDMAQPAYEQIAI-Y----
--RA-

GIVPVMY-RRVQCIKFG-GIHFT--MNGN--RW-EV---LVLLTNVGGAGNVHAVS--VKGPH--T-N-WL---
YMRRNW

GQNWQINDKQ---LAGATLSFRVTT--E-DGRVVTSWDVVPHYW--RFGQTFE-GL---NF

>MpEXPA10PRO

W--N------Y-AHATFY--GDETAQDTM----Q-GACGY-GNMYQ-
QGYGTATTALSSTLFDNGFACGRCFEIRCVA--

---------S----WACYL-GNPSTIVTATNFCPANWLKPTN--NGGWCNPPRQ-HFDMSKPAFEKIAA-W----
--RA-

GIVPVAY-RRVPCSKQG-SMEFR--IEGN--PW-WH---IVFITNVGGPGDIGEVY--IRGSG--TKR-WV---
RMSHNW

GVGFQ-VFGG---YLGQGLSFMVIS--SSNLETVMAWNVVPANW--RLGQTFSGG----QF

>MpEXPA11PRO

W--M------S-AKATFY--GGQDASGTM----N-GGCGY-ANPFT-
LGYGAQTAALSNALFNKGLTCGACFEIKCAI--

--TATAYAKQ----W-CYA-NSPSIYITATNLCP-----QGS--YGGWCNGANL-HFDLAYAAFTKLAR-E------
NA-

GVIPVNY-RRTPCKKSG-GIKFQ--LNGN--TY-WN---LVLIYNVGGGGNVAAAA--IKGSR--S-N-FY---
TMNQNW

GMNWE-SYSN---LVGQALTFKITL--G-NGRSATFYNVAPAGW--RFGQTYQAAY---NF

>MpEXPA12PRO

---M------D-AKATYY--GGNDASGTM----N-GGCGY-ANPFA-
LGYGDQTVALSTALWNKGLTCGACFEIKCKI--

--SATAYAKK----W-CYP-KAGSIKITATNLCP-----PGS--HGGWCDPPKS-HFDLAYAAFTRLAN-F------
QA-

GVIPIQY-RRTPCRKNG-GVKFS--MNGN--PY-WH---LVLVHNVAGGGNVHAMQ--IKGSR--T-G-FM---
QMKQNW

GQNWE-AYCN---LVGQSVTFKVTL--G-NGRTVTFWNAVASNW--KFGQTYQTKY---NF

>MpEXPA13PRO

W--A------E-AHATFY--GGQDAGGTM----G-GACGY-GNLYS-
LGYGTATTALSNALFHNGLSCGACFLIECIL--

------QGSK----W-CYP-GK-SIVVTATNACP-----PGS--EGGWCDPPRP-HFDLSFPMFQMLAQ-P------
VA-

GVIPVRY-RRVSCSKQG-GVRFT--LHGN--PY-FN---YILVHNVGGSGDVAAVA--LRGEN--S-R-WN---
RMNQNW

GQYWS-CGGW---YLGQALSFKVTL--G-SGRSLEFWNVVDKNW--GFGQTYESDY---NF

>MpEXPA14PRO

W--A------D-AHATFY--GGQDASGTM----G-GACGY-GNLYS-
TAYGTASTALSNALFNNGLSCGACFQIMCKL--

------DGSK----Y-CYT-GGKSITVTATNACP-----PGS--EGGWCDPPKA-HFDLSYPMFQQLAE-P------
VG-

GVIPVQY-KRVSCQKNG-GIRFT--INGN--AW-FD---LVLVTNVGGGGDIAKLE--MKGAS--T-D-WC---
PMSQNW

GQNWQ-ASGHPE-LCGQTLSFRATL--G-DGRSQEFYDVAPSNW--GFGQTFEANT---NF

>MpEXPA15PRO

W--A------D-AHATFY--GGSDASGTM----G-GACGY-GNLYS-
LGYGTSSTALSNALFNNGLSCGACFLIQCKL--

--EG----SK----Y-CYP-GQ-SITVTATNACP-----PGS--EGGWCDPPRS-HFDLSFPMFQKLAQ-P------
VG-

GVIPVRY-KRVSCTKQG-GVRFT--LHGN--PY-FN---YVLVHNVAGWGDVSSLA--IKGDQ--T-G-WY---
YMSQNW

GQYWS-ASAK---LTGQALSFKVTL--G-NGKSLEFYNVADKNW--GFGQTYESDY---NF

>MpEXPA16PRO

W--G------D-AHATFY--GGQDASGTM----G-GACGY-GNLYS-
VGYGVSTAALSYAMFNKGLTCGACYELVCKL--

------EGSK----Y-CYP-GK-SITITATNSCP-----PGS--EGGWCDPPKL-HFDLSYPMFQKLAQ-P------VG-

GVIPIKY-KRVACAKSG-GVRFT--IHGN--PY-FN---YILVHNVAGAGDVSALA--IKGEK--T-G-WY---
YMSQNW

GQFWS-ASAN---VVGQALSFKITL--G-NGVSTEFSNVAPKDW--KFGQTFEADN---NF

>MpEXPA17PRO

W--EVTEFTRG-ARATYY--GGNDASGTL----G-GACGY-GNLFE-
NGYGVETTALSTALFNDGLTCGGCFQIQCIA--

------SESD----W-CLANGTVSITVTATNFCP-----PGS--FGGWCDPPQP-HFDLSYPMFATLAQ-P------
VG-

GVIPVQY-RRVSCLKQG-GVRFQ--INGN--NW-FF---LVLVMNVAGAGDVQAVA--IKGTR--S-E-WI---
SMQRNW

GQNWA-SDGPPE-LVGQALSFRVTL--GLTKETLDFIDVAPADW--QFGQTFEDDSGL-NF

>MpEXPA18PRO

C--I------P-ATATFY--GGNDASGTN----T-GACAY-PNVLA-
MGYGTMNAALSSAIFDHGKTCGACFQLVCTY--

---VPYGNLR------CLR-GR--ITVTATNYCP-----QGS--KGGWCDAPRS-HFDLAEPAFVQIAP-Y------
NA-

GVVHVDY-QRVNCQRSG-EIRYR--LYGF--TY-FL---QVLVYNVAGTGDVTAMS--IKGSS--EGR-WI---
SMTRSW

GQLWT-TGVV---LEHQALSFMVTI--S-DGRTITVNDVANEYW--RYGQTYE-GG---QF

>MpEXPA19PRO

W--T------D-SHATFY--GGNNAQGTM----G-GACGY-GNMYS-
RGYGLETTALSSTLFNKGLTCGACFEIKCKV--

------ADTR----W-CYA-NAGSIKVTATNLCPANPARPTN--NGGWCNPPRT-HFDLSYPMFTRLAK-A----
--VG-

GIIPVQF-RRVPCVKVG-GIRFV--MNGN--PW-FN---LVLVYNVAGAGNVVNMQ--MKGSK----TNWF--
-TMKQNW

GQNWE-LKQK---LKGQSISFRVTL--G-NGKVMIANNVAPANW--NFQQTFESDN---NF

>MpEXPA20PRO

W--M------D-AHATFH--GGSAVADTM----G-GACGY-GNLIS-
RGYGLHTSALSSSLFNSGLTCGACFKVRCKA--

--AS----SM----R-CHA-TAGSITVTATDLYSENLATLIE--DERRSNPSQP-QFDLAVSAFQMLAR-P------
MV-

GLIPIEY-QRVPCEKEG-GIRFT--LNGN--SW-FN---LVLVHNVGGDGNVVAVQ--VKGSR--ASS-WS---
MMNRRW

GQNWE-LREN---LQGQILSFRITT--G-FGKTVLLPDVAQADW--RFGQTYESEG---NF

>MpEXPA21PRO

W--T------D-SHATFY--GGNDGSGTM----G-GACGY-GNMYS-
RGYGLKTAALSSTLFNNGLSCGACFELKCKV--

------AESR----W-CYA-NAGSITVTATNLCPANPARPTN--NGGWCNPPRT-HFDLAYAMFIIMAK-P------
VG-

GIIPVQY-RRVSCVKTG-GVRFT--LNGN--PW-FN---LVLVYNVAGDGNVVTIK--MKGSK--SNT-WY---
PMKHNW

GQNWE-LKQK---LAGQALSFQLTT--G-YGKTMTFTNCAAANW--QFGQTYEADG---NF

>MpEXPA22PRO

W--T------A-SDATFY--GGNSGAGTM----G-GACGY-GNMYN-
TGYGLNTAALSPVLFNNGLSCGACFELKCNL--

------TGTK----W-CYP-SAGSITVTATNLCPANPARTTD----GWCNPPRT-HFDLSYTMFAILAQ-P------
VA-

GIIPIQY-RRVSCVKTG-GVRFV--INGN--PW-FN---LVLIYNVGGDGNVVTAM--MKGEN----TEWY---
SMTRNW

GQNWQ-LSQK---LHGQALSFQLTT--G-YGTTLVCSDVAPANW--QFGQTFEAYT---NM

>MpEXPA23PRO

A--I------P-ATATFY--GGNDASGTN----T-GSCGY-PNVLT--
IDGPMNTALSGALYNGGEACGACFKIQCVS--

---IPFGNLQ------CLP-GS--IVVTATNLCP-----QGS--TGGWCDEPRS-HFDLAEPAFQHLAP-P------
VA-

GVVNVEY-ERVSCLRSG-GIRFL--IQGH--PY-FM---QVLVYNVGGMGDVTAVS--VKGSS----SDWI---
QMDRNW

GQLWT-TGTV---LDGQALSFSVTT--S-DGRTVASNGVADSDW--QYGQTFE-GN---QF

>MpEXPA24PRO

A--I------P-ATATFY--GGNDASGTN----TAGSCGY-PNVLT--
IDGPMNTALSGALYNGGEACGACFKIQCVS--

---IPFGNLQ------CLP-GS--IVVTATNLCP-----QGS--TGGWCDEPRS-HFDLAEPAFQHLAP-P------
VA-

GVVNVEY-ERVSCLRSG-GIRFL--IQGH--PY-FM---QVLVYNVGGMGDVTAVS--VKGSS----SDWI---
QMDRNW

GQLWT-TGTV---LDGQALSFSVTT--S-DGRTVASNGVADSDW--QYGQTFE-GN---QF

>MpEXPA25PRO

W--T------N-ATATFY--GGDDGAGTM----G-GACGY-GNMFT-
RGYGLETAALSTVLFNNGLTCGACFMLKCRL--

------NESR----W-CYS-NVSSITISATNFCPPNLNRTTD----GWCNLPRS-HFDLSVKMFTTLAR-A------
VG-

GIIPVLY-KRVPCVKTG-GVRFT--LNGN--PW-FN---LVLISNVGGQGNVVAAQ--MKGSK----TAWF---
NMRQNW

GQNWE-LGQK---LINQTLSFRLTL--G-LGQTLTFNNLTTPDW--KFGQTWEADT---NF

>MpEXPA26PRO

F--G------D-ATATFY--GGSDASGTQ----G-GSCGY-QNPFA-
LGYGIKTAALSNSLLANNMNCGACFEVKCKY--

--DASAYTKK----W-CYP-NSPTIIVTATNQSP-----PGL--NG------EH-HFDLTEPMFMKIAN-R------IG-

GRIPIQY-RRVRCYKPG-GIKFT--LTGN--PF-FN---LVLVYNVGGAGNVYGMA--IKGSR--T-D-FY---
PMSRNW

GQYWQ-AFVK---LTGQSLTFRVTL--G-NGKSTTFWNVAPANW--QFGQTVAATY---NF

>MpEXPA27PRO

F--G------D-ATATFY--GGTDAAGTA----G-GSCGY-ENPFT-
RGYGVTTAALSNSLLSDGLNCGACFEVKCNF--

--GASDYTRK----Y-CYN-GK-SVVITSTNLSP-----PGL--HGE------H-HFDLTYPMFTQISN-M------IA-

GRIPVQY-RRVPCKKQG-GIKFN--INGN--PY-FN---LVLVYNVAGGGNVYAMA--MKGGN--S-A-WC---
TMGRNW

GQNWQ-SSVK---LTGQGLSFRVTL--G-SGKMLVFNNVAPSSW--QFGQTYQASS---NF

>MpEXPA28PRO

F--G------D-ATATFY--GGSDAAGTA----G-GFCGY-ENPFT-
RGYGVTTVALSDSLLSNGLNCGACYEVKCKW--

--EASDYTKK----Y-CYQ-GM-TTIVSATNLSP-----PGL--NGEH-------HFDLTYPMFVKISN-P------ES-

GRIPIQY-RRVPCQKQG-GIRFS--MVGN--PF-WN---LVLVYNVGGWGNVHSLY--VRGSR--T-D-WY---
YMNRNW

GQNWQ-CSVN---LTGQKLSFRVVL--G-NGQTSVFNNVADSNW--KHGQTYEASS---NF

>MpEXPA29PRO

W--T------D-SHATFY--GGNNAQGTM----G-GACGY-GNMYS-
RGYGLETTALSSTLFNKGLTCGACFEIKCKV--

------ADTR----W-CFP-KAGSIKVTATNLCPANPARPTN--NGGWCNPPRT-HFDLSYPMFTRLAK-A-----
-VG-

GIIPVQF-RRVPCVKVG-GIRFV--MNGN--PW-FN---LVLVYNVAGGGNVVNMQ--MKGSK----TNWF--
-TMRQNW

GQNWE-LKQK---LKGQSISFRMTL--G-NGKVMIANNVAPANW--NFQQTFESDS---NF

>MpEXPA30PRO

W--T------D-SHATFN--DGNNAQGTM----G-GACGY-GNISS-
RGYGLETTALSSTLFNSGLTCGACFEIKCKV--

------ENST----W-CFP-NAGSIKVTATDLCPANPAMPTN--NGGWCNPPRT-HFDLSYQMFTRLAK-A----
--EG-

AIIPVQF-RRVPCVKVG-GIRFT--MNGN--PW-FN---LVLVSNVAGAGNVVNLQ--MKSSE--TNWFWM-
--TARQIS

GQYWE-LNQN---LMGQNISFRVTL--G-NGKVMIANNVVPANW--NFQQTFESDI---NF

>MpEXPA31PRO

W--T------D-SHATFY--GGNNAQGTM----G-GACGY-GNMYS-
RGYGLETTALSSTLFNKGLTCGACFEIKCKL--

------ADSR----W-CFP-KAGSIKVTATNLCPANPARPTN--NGGWCNPPRT-HFDLSYPMFTRLAK-A----
--VG-

GIIPVQF-RRVPCVKVG-GIRFV--MNGN--PW-FN---LVLVYNVAGAGNVVNMQ--MKGSK----TNWF--
-TMRQNW

GQNWE-LKQK---LKGQSISFRVTL--G-NGKVIIANNVAPANX--NFQQTFESDS---NF

>MpEXPA32PRO

W--T------D-AHATFY--GGQDASGTM----G-GACGY-GNLYS-
RGYGTASTALSNALFNSGLSCGACFEIICKL--

------SETK----W-CYT-GGKSIVVTATNQCP-----QGS--EGGWCDYPRS-HFDLSFPMFQSLAQ-A------
VG-

GVIPVQY-RRVSCSRSG-GIRFT--INGN--AY-FN---LVLVTNVGGKGDVAALW--MRGSN--T-D-WQ---
PMKQNW

GQNWQ-CDTK---LVGQALSFKAML--G-SGETKEFYNVADSNW--LFDQTYEGDY---NF

>MpEXPA33PRO

G--L------K-AHATFL--GGSDAVGTN----N-GACGY-SNVLA-
MGYGTRTAALSAPIFREGKSCGACYKLRCRG--

------SESV----G-CRS-ND-GVVVTVTDVCP-----ERS--VGGWCD-GQP-HFRLSEPAFAQIAE-P------
KSG

GLVEVDY-EKVSCDRRG-GMHFL--LVGH--TH-FL---QILVHNVGGCGDVAAVS--VKGGT----GPWI---
RMSRSW

GQLWH-TGEV---LDGQALSISVTM--A-DGKVCVSNNFAGADW--KYGQTFE-GA---QL

>MpEXPA34PRO

A--L------P-AHATFY--GGSDASGTN----N-GACGY-SNVLS-
TPFGTMTAALSSPLFNEGLSCGSCYKVRCSG--

------SSTG----G-CID---NEIMVTATNLCP-----HGS--EGGWCD-GKP-HFDMSQPAFSQIAK-P------
VA-

GVVEVEY-TKVPCPRKG-GMQFT--VMGH--TY-FL---QVLIFNVGGSGDVTAVK--VRGGN----GEWI---
AMTRSW

GQLWQ-TGKV---LDGQALSFQVTT--S-NGKTCVSNYVAGSHW--KYGQTFE-GS---QY

>MpEXPA35PRO

W--T------D-SHATWY--GGKDGAGTM----G-GACGY-GNMNA-
RGYGMKTAALSTKLFNDGFTCGACFEIRCKS--

------SQSK----Y-CYS-STASVIVTATNYCPANPNRPTD----GWCNGDRT-HFDLTYQMFTTLAI-Y------
EA-

GIIPVQY-RRVPCAKKG-GIRFT--INGN--PN-FN---LILLSNVGGSGNVVSVQ--MKGEN--SYSGWV---
PMKQNW

GQMWE-CGTK---VTGQSISFKVTV--G-YGQTFTFWTVAPKTW--QFGQTYEASS---NV

>MpEXPA36PRO

W--G------D-AHATFY--GGNDASGTM----G-GACGY-GNLYS-
LGYGTASTALSNGLFNNGLSCGACYTIECKL--

------EGSK----W-CYP-GK-SITVTATNACP-----PGS--EGGWCDPPRL-HFDLSYPMFQTLAQ-P------
VG-

GVIPVKY-KRVSCNRQG-GIRFT--LHGN--AW-FN---YVLVHNVAGDGDVAGVS--IKGDN--G-G-WT---
TMYQNW

GQFWS-ASEH---LVGQALSFRVTL--G-SGRTQEFYDVASPNW--YFGQTYEADNNQ-NF

>MpEXPA37PRO

W--G------N-AHATFY--GGNDASGTM----G-GACGY-GNLYS-
RGYGTATTALSNGLFNNGLTCGACFTLVCKL--

------DGSR----W-CYS-GGKQITVTATNACP-----PGS--EGGWCDPPRL-HFDLSYPMFQKLAQ-P------
VG-

GVIPVRY-KRVPCVKQG-GIRMS--LYGN--PW-FD---YVLVHNVAGGGDVAAVS--IKGSK--T-N-WV---
VMHQNW

GQYWS-ASSY---LVGQALSFKVTL--G-NGKSIVFWDIAPKNW--QFGQTYEADNNQ-NF

>MpEXPA38PRO

W--K------A-AHATFY--GGSNAQGTM----G-GACGY-GNLYN-
TGYGLETAALSSTLFNNGKTCGACYEMFCNY--

-----RQQSR----W-CYK-AQPHIKVTATNLCPANWNRPTN--RGGWCNPPRV-HFDLPQPMFIKMAR-Y-
-----VG-

GIVPVAY-RRVPCVKKG-GVKFT--MNGN--PW-FN---LILISNVAGPGDIKAVA--VKGSK--QKS-WT---
SARQNW

GQKWE-VKQK---LQGQALSFRLTS--G-SGKVLVLYNAVPRTW--RFGSTYMARS---NW

>AaEXPA1PRO

W--Q------D-GHATFY--GGSDASGTM----G-GACGY-SNLYS-
QGYGLATAALSTALFNNGLSCGACFEIKCNQ--

--------PE----W-CVA-GASSIVVTATNLCPPNPSLPND--NGGWCNEPLQ-HFDMSQPAFQQIAQ-Y----
--KA-

GIVPVSY-RRVSCVKKG-GVHFT--VNGH--SY-FN---LVLVSNVGGAGDVHAVS--IKGSN--T-G-WE---
PMSRNW

GQNWQ-SNSK---LDGQSLSFQVTT--S-DGQTLTFYDVASDNW--SFGQTFEGDS---NF

>AaEXPA2PRO

W--S------S-AHATFY--GGNDASGTL----G-GSCGY-GNLYD-
RGYGTSTAALSTALYNNGASCGACFEIQCTG--

--------SR----W-CKG-SA-PITVTATNFCPQNPALPNN--AGGWCNLPLQ-HFDMSVPAFLEIAE-Q------
VG-

GIVPVNY-RRVSCVKKG-GIHFT--INGH--AY-FN---LVLITNVGGAGDVHAVS--VKGSK--T-G-WE---
PMTRNW

GQNWQ-CNSY---LNGQSLSFQVTT----SGGTLISYDVTPAGW--SFGQTFS-GA---QF

>AaEXPA3PRO

W--N------S-AHATFY--GGNDASGTM----G-GACGY-GNLYS-
EGYGTNTAALSTALFDNGLSCGACYEIACDS--

------SGTK----Y-CT--GSNSITITATNFCPENLALPSD--NGGWCNQPRQ-HFDMAVPAFDQLAA-H-----
-VA-

GIVPIKY-RRVSCVRKG-GVRFH--LNGH--DY-FL---LLLVDNVGGNGAVHSVS--IKAGN----GPWK---
ALSQNW

GQNWQ-CNDY---ISGQTLSISITT--E-DGKTLVSNNVAPAGW--KFGETYA-GS---QY

>AaEXPA4PRO

WVKG------V-AHATFY--GGSDASETN----G-GACGY-GNLYS-
QGYGTNTAALSSALFGGGETCGACYQLKCTG--

--------------G-CFS-GAPKIVVTATNYCP-----EGS--TGGWCNAPRV-HFDLSEPAFTRMAA-A------VT-

GVVPVNY-RRVKCVKEG-GIKFQ--INGH--SY-FN---LILITNVGGVGDVKEVY--IQVGS--GSS-WK---
ACKRNW

GQNWE-NDDY---LDGKSFSIKVIT--S-DNRFCNSPNVIPAGW--KFGTTYE-GR---QF

>AaEXPA5PRO

W--K------A-AHATFY--GGNDASGTN----G-GACGY-GNIYS-
FGYGIANAALSYALFNSGAQCGACYEIKCYG--

--------SK----W-CLP-GNPSITITATNSCPPNWALPSD--NGGWCNPPRE-HFDMSVPAWTSLAV-Y-----
-VG-

GIVPVNY-RRVPCSRKG-GIRFT--ISGN--PN-FD---LVLVHNVAGDGVVTALS--IKGNG----N-WV---
PMKRNW

GQNWQ-CGAV---LTGQPLSFMVRT--S-DGRSLILYNVAPANW--SFGSTYQSAQ---NF

>AaEXPA6PRO

---N------D-AHATFY--GDPSGSATF----G-GACGY-GDLMA-
QGYGLQTTALSEVLFSNGFSCGQCYQIQCID--

--------SK----W-CIP-NA-VITVTATNLCPADYSKPND--NGGWCNPPLK-HFDLSMPAFHQIAQ-E------
VA-

GIVPVTY-RRVPCVKLG-GVKFT--ILGN--TW-WH---LVLITNVGGAGDIDTAS--IKGSN--T-D-WI---
PMSHNW

GASFQ-CFQS---LVNQALSFQLTS--T-TGQVLTTFNVAPADW--SFGTTYE-GG---QY

>AaEXPA7PRO

W--T------TGATATYY--GGSDATATN----G-GACGY-GNQIA--
LYGTVSTALSSAFFQNGVTCGACYEIQCTG--

--------SS----Y-CYS-GK-TVKVTATNLCP-----DGS--YGGWCDGSKK-HFDLSQPAFIQVAQ-Q------VV-

GHFAAQY-RRVPCTRSG-GVRFT--INGN--TY-YN---LVLIQNVAGVGDVKAVS--IKGTS--T-G-WL---
SMSRNW

GENWE-CDTK---LVGQALSFQVTL--S-DSTTKTFNNIAASNW--AFGSTYSSSV---QF

>AaEXPA8PRO

W--Q------D-GHATFY--GGSDASGTM----G-GACGY-SNLYS-
QGYGLATAALSTALFNNGLSCGACFEIKCNQ--

--------PE----W-CVA-GASSIVVTATNLCPPNPSLPND---GGWCNEPLQ-HFDMSQPAFQQIAQ-Y-----
-KA-

GIVPVSY-RRVSCVKKG-GVHFT--VNGH--SY-FN---LVLVSNVGGAGDVHAVS--IKGSN--T-G-WE---
PMSRNW

GQNWQ-SNSK---LDGQSLSFQVTT--S-DGQTLTFYDVASDNW--SFGQTFEGDS---NF

>AtEXPA7PRO

W--R------Y-AHATFY--GDETGGETM----G-GACGY-GNLFN-
SGYGLSTAALSTTLFNDGYGCGQCFQITCSK--

--------SP----H-CYS-GK-STVVTATNLCPPNWYQDSN--AGGWCNPPRT-HFDMAKPAFMKLAY-W---
---RA-

GIIPVAY-RRVPCQRSG-GMRFQ--FQGN--SY-WL---LIFVMNVGGAGDIKSMA--VKGSR----TNWI---
SMSHNW

GASYQ-AFSS---LYGQSLSFRVTS--YTTGETIYAWNVAPANW--SGGKTYKSTA---NF

>AtEXPA8PRO

W--Q------G-GHATFY--GGEDASGTM----G-GACGY-GNLYG-
QGYGTNTAALSTALFNNGLTCGACYEMKCND--

-------DPR----W-CLG---STITVTATNFCPPNPGLSND--NGGWCNPPLQ-HFDLAEPAFLQIAQ-Y------
RA-

GIVPVSF-RRVPCMKKG-GIRFT--INGH--SY-FN---LVLISNVGGAGDVHAVS--IKGSK--TQS-WQ---
AMSRNW

GQNWQ-SNSY---MNDQSLSFQVTT--S-DGRTLVSNDVAPSNW--QFGQTYQGG----QF

>AtEXPA11PRO

L--T------N-GHATFY--GGSDASGTM----G-GACGY-GDLYS-
AGYGTMTAALSTALFNDGASCGECYRITCDH--

-----AADSR----W-CLK-GA-SVVITATNFCPPNFALPNN--NGGWCNPPLK-HFDMAQPAWEKIGI-Y----
--RG-

GIVPVVF-QRVSCYKKG-GVRFR--INGR--DY-FE---LVNIQNVGGAGSIKSVS--IKGSK--T-G-WL---
AMSRNW

GANWQ-SNAY---LDGQALSFSITT--T-DGATRVFLNVVPSSW--SFGQIYSSNV---QF

>AtEXPA12PRO

W--I------R-AHATYY--GVNDSPASL----G-GACGY-DNPYH-
AGFGAHTAALSGELFRSGESCGGCYQVRCDF--

---PA--DPK----W-CLR-GA-AVTVTATNFCP-----TNN--NNGWCNLPRH-HFDMSSPAFFRIAR-R------
GNE

GIVPVFY-RRVGCKRRG-GVRFT--MRGQ--GN-FN---MVMISNVGGGGSVRSVA--VRGSK--GKT-WL-
--QMTRNW

GANWQ-SSGD---LRGQRLSFKVTL--T-DSKTQTFLNVVPSSW--WFGQTFSSRGR--QF

>AtEXPA13PRO

W--R------P-ARATYY--AATNPRDAV----G-GACGY-GDLVK-
SGYGMATVGLSETLFERGQICGACFELRCVD--

-------DLR----W-CIP-GT-SIILTATNFCAPNYGFDPD--GGGHCNPPNK-HFVLPIEAFEKIAI-W------
KA-

GNMPVQY-RRINCRKEG-SMRFT--VDGG--GI-FI---SVLITNVAGSGDIAAVK--IKGSR--T-G-WL---
PMGRNW

GQNWH-INAD---LRNQALSFEVTS--S-DRSTVTSYNVSPKNW--NYGQTFE-GK---QF

>AtEXPA14PRO

W--V------N-ARATFY--GGADASGTM----G-GACGY-GNLYS-
QGYGTNTAALSTALFNGGQSCGACFQIKCVD--

-------DPK----W-CIG-G--TITVTGTNFCPPNFAQANN--AGGWCNPPQH-HFDLAQPIFLRIAQ-Y------
KA-

GVVPVQY-RRVACRRKG-GIRFT--INGH--SY-FN---LVLITNVAGAGDVISVS--IKGTN--T-R-WQ---
SMSRNW

GQNWQ-SNAK---LDGQALSFKVTT--S-DGRTVISNNATPRNW--SFGQTYT-GK---QF

>AtEXPA15PRO

W--V------N-AHATFY--GGSDASGTM----G-GACGY-GNLYS-
QGYGTNTAALSTALFNNGLSCGACFEIKCQS--

--------DG---AW-CLP-G--AIIVTATNFCPPNNALPNN--AGGWCNPPLH-HFDLSQPVFQRIAQ-Y------
KA-

GVVPVSY-RRVPCMRRG-GIRFT--INGH--SY-FN---LVLVTNVGGAGDVHSVA--VKGSR--T-R-WQ---
QMSRNW

GQNWQ-SNNL---LNGQALSFKVTA--S-DGRTVVSNNIAPASW--SFGQTFTGR----QF

>AtEXPA17PRO

W--L------Q-AHATFY--GGSDASGTM----G-GACGY-GNLYT-
DGYKTNTAALSTALFNDGKSCGGCYQILCDA--

-----TKVPQ----W-CLK-GK-SITITATNFCPPNFAQASD--NGGWCNPPRP-HFDMAQPAFLTIAK-Y------
KA-

GIVPILY-KKVGCRRSG-GMRFT--INGR--NY-FE---LVLISNVAGGGEISKVW--IKGSK--SNK-WE---
TMSRNW

GANYQ-SNTY---LNGQSLSFKVQL--S-DGSIKAALNVVPSNW--RFGQSFKSNV---NF

>AtEXPA20PRO

W--K------I-ATATLS--RDRDGSSSVAT--G-GACGY-GDLRQ-
SSFAGYSAGLSGKLFNRGSSCGACLEVRCVN--

-------HIR----W-CLQ-GSPSVVVTATDFCPPNSGLSSD--YGGWCNFPKE-HLELSHAAFTGIAE-T------
RA-

EMIPIQY-RRVKCGRRG-GLRFS--LSGS--SH-FF---QVLISNVGLDGEVVGVK--VKGHT--T-A-WI---
PMARNW

GQNWH-SSLD---LIGQSLSFEVTL--K-GGKTIASYDVAPPYW--RFGMTYQ-GK---QF

>AtEXPA22PRO

W--Y------D-ARATFY--GDIHGGDTQ-------PCGY-GNLFR-
QGYGLATAALSTALFNDGYTCGACYEIMCTR--

-------DPQ----W-CLP-G--SVKITATNFCPANYSKTTD----LWCNPPQK-HFDLSLAMFLKIAK-Y------
KA-

GVVPVRY-RRIPCSKTG-GVKFE--TKGN--PY-FL---MVLIYNVGGAGDIKYVQ--VKGNK--T-G-WI---
TMKKNW

GQNWT-TITV---LTGQGLSFRVTT--S-DGITKDFWNVMPKNW--GFGQTFDGRI---NF

>OsEXPA4PRO

W--Q------S-AHATFY--GGGDASGTM----G-GACGY-GNLYS-
QGYGTNTAALSTALFNDGAACGSCYELRCDN--

------AGSS------CLP-G--SITVTATNFCPPNYGLPSD--DGGWCNPPRP-HFDMAEPAFLHIAQ-Y------
RA-

GIVPVSF-RRVPCVKKG-GVRFT--VNGH--SY-FN---LVLVTNVAGAGDVRSVS--IKGSR--T-G-WQ---
PMSRNW

GQNWQ-SNAF---LDGQSLSFQVTA--S-DGRTVTSNNVAHPGW--QFGQTFEGG----QF

>OsEXPA5PRO

W--S------S-AHATFY--GGGDASGTM----G-GACGY-GNLYS-
QGYGTNTAALSTALFNNGLSCGACFEVRCDA--

-----GGGGS----HSCLP-G--SVVVTATNFCPPNNALPSD--DGGWCNPPRA-HFDMSQPVFQRIAL-F--
----KA-

GIVPVSY-RRVACQKKG-GIRFT--INGH--SY-FN---LVLVTNVGGAGDVHAVA--VKSER--SAA-WQ---
ALSRNW

GQNWQ-SAAL---LDGQALSFRVTT--G-DGRSVVSNNAVPRGW--SFGQTFS-GA---QF

>OsEXPA7PRO

W--Q------S-AHATFY--GGSDASGTM----G-GACGY-GNLYS-
QGYGVNNAALSTALFNSGQSCGACFEIKCVN--

-----QPGWE----W-CHP-GSPSILITATNFCPPNYALPSD--NGGWCNPPRP-HFDLAMPMFLHIAE-Y---
---RA-

GIVPVSY-RRVPCRKKG-GVRFT--INGF--RY-FN---LVLITNVAGAGDIVRAS--VKGTS--T-G-WM---
PMSRNW

GQNWQ-SNSV---LVGQALSFRVTG--S-DRRTSTSWNAAPAGW--HFGQTFE-GK---NF

>OsEXPA10PRO

W--R------S-AKASYY---AADPEDAI----G-GACGF-GDLGK-
HGYGMATVGLSTALFERGAACGGCYEVKCVD--

-------DLK----Y-CLP-GT-SIVVTATNFCAPNFGLPAD--AGGVCNPPNH-HFLLPIQSFEKIAL-W------
KA-

GVMPIQY-RRVNCLRDG-GVRFA--VAGR--SF-FL---TVLISNVGGAGDVRSVK--IKGTE--S-G-WL---
SMGRNW

GQIWH-INSD---FRGQPLSFELTS--S-DGKTLTNYNVVPKEW--DFGKTYT-GK---QF

>OsEXPA11PRO

W--S------S-GSATFY--GGSDASGTM----G-GACGY-GNLYS-
AGYGTSTAALSTALFNNGQSCGACFEVRCGG--

-------GGS------CLA-G--TVAVTATNLCPPNYALAGD--AGGWCNPPRP-HFDMAEPAFTRIAQ-A------
RA-

GVVPVQY-RRVACAKQG-GIRFT--ITGH--SY-FN---LVLVTNVGGAGDVTAVS--VKGSR--S-G-WQ---
AMSHNW

GANWQ-NGAN---LDGQPLSFRVTA--S-DGRTVTSDNVAPSGW--SFGQTFSGG----QF

>OsEXPA12PRO

A--L------S-GTATFY--GGSDASGTM----G-GACGY-GNLYS-
TGYGTNTAALSSALFNDGAACGECYQITCDQ--

------SNSK----W-CKA-GT-SVTITATNLCPPDYSKPSN--DGGWCNPPRQ-HFDMAQPAWEQIGV-Y---
---RG-

GIVPVNF-QRVSCTRKG-GVRFT--INGN--SY-FE---LVLITNVGGPGSIKSVQ--IKGTK--T-G-WV---
TMSRNW

GANWQ-ANNY---LNNQAISFSVTS--T-AGKTLVFEDVAPSNW--QFGQTFTSGV---QF

>OsEXPA16PRO

W--R------M-GSATYI----KESLGHPLNDGG-GACGY-GDLDI-
FRYGRYTAGVSGALFGRGSACGGCYEVRCVN--

-------HVL----W-CLR-GSPTVVVTATDFCAPNLGLSDD--YGGWCNFPKE-HFEMSEAAFLRVAK-A----
--KA-

DIVPVQF-RRVSCDRAG-GMRFT--ITGG--AS-FL---QVLITNVAADGEVAAVK--VKGSR--T-G-WI---
PMGRNW

GQNWQ-CDAD---LRGQPLSFEVTG--G-RGRTVVAYSVAPPDW--MFAQTFE-GK---QF

>OsEXPA30PRO

W--T------P-AHATFY--GDETASETM----G-GACGY-GNLYA-
SGYGTDTAALSTTLFKDGYGCGTCYQMRCVG--

--------TA----S-CYR-GSPAITVTATNLCPPNWAEDPDRGGGGWCNPPRA-HFDLSKPAFMRMAD-W-
-----RA-

GIVPVMY-RRVPCARAG-GLRFA--LQGN--PY-WL---LAYVMNVAGAGDVGDMW--VKAGG--GGG-
WV---RMSHNW

GASYQ-AFAQ---LGGQALSFKVTS--YTTGQTILAAGVTPASW--CFGLTYQARV---NF

>OsEXPA32PRO

W--K------Q-AHATFY--GGRDGSGTL----D-GACGY-KDTSK-EGYGVQTVAVSTPLFGAGAGCGACYEVKCVD--

-------SPD----G-CKV-GAAPLVVTATNLCPPNPGQSND--NGGWCNPPRE-HFDLSMPAFLQIAQ-E------KA-

GIVPISY-RRVPCVKVG-GIRYT--ITGN--PY-FN---LVMVSNVGGAGDVAGLS--VKGNK--RVK-WT---PLKRNW

GQEWQ-TSEV---LTGESLTFRVMT--G-DHRKATSWHVLPPDW--QFGVTYQATK---NF

>OsEXPA33PRO

W--T------P-ATATFY--GGSDGAGTM----G-GACGY-GNLYN-AGYGLNNAALSSALFNDGAMCGACYTIACDT--

------SQST----W-CKP-GT-SITITATNLCPPNYAKKSD--AGGWCNPPRK-HFDMSQPAWTSIAI-Y------QA-

GIVPVNF-KRVPCQKSG-GIRFT--ISGR--DY-FE---LVTVFNVGGSGVVAQVS--IKGSK--T-D-WM---AMSRNW

GQNWQ-SNAY---LNTQSLSFKVKL--D-DAREVTVWNIAPSNW--NFGTTYTSNI---NF

>CpEXPB1 [X]

W--M------A-GHATWY--GEPNGEGSS----G-GACGY-TKLTG-TPYGPRIAAGNDAIFQGGKGCGQCYEVKCSY--

--------AE------CRQ---EATRIVITDQCP----------GGEYCSKAEP-AFDLSGAAISAMAL-PDKDGALRNI

GLYDILY-KRVPCEFQGGQNIAFQ--VDQGSTGF-WL---SFVVKFLGGPGDIDSVE--IRHSG--SSS-FQ---PAKHNW

GASWM-LINTSGQAFKGPYDIRVVS--KLNGHAVTAEKVIPEFF--EPGKLYESNV---QL

>CpEXPB2 [A]

W--V------F-GHGTWY--GDPYGEGSS----G-GNCGY-TKLAG-TPYGPKIVAGSPAIYANGLGCGQCYQVKCTD--

----SKNGPR----L-CNP---SGTQVVVTDFCP----------GGTYCSTGEK-AFDFSGAALNAMAL-PGKDGQLRNR

GLYNVLY-KRVPCKYKGQNIAFR--VDPGSSPY-WL---SVLIKYVGGPGDIGAVS--IKMAN--SYK-FM---PMKHAW

GANWM-LQNYDGKPFKGPMDIKIVS--RLNGHSVVAKGVIPGYF--RPGTTYTSRV---QM

>CpEXPB3 [A]

W--V------Y-GHGTWY--GDPYGEGSS----G-GNCGY-TKLAG-TPYGPKIVAGSPAIYANGLGCGQCYQIKCLD--

----SKYGPR----L-CNS---WGTQVVVTDFCP----------GGTYCSSGQK-AFDFSGAALNAMAL-
PGKDGQLRNR

GLYNVLY-KRVPCKYKGQNIAFR--VDPGSSPY-WL---SVLIKYVGGPGDIGAVY--IKMAN--SYR-FM---
PMKQAW

GANWM-LQNYDGKPFKGPMDIKIVS--RLNGHSVVAKGVIPGYF--RPGTTYTSRV---QM

>CpEXPB4 [A]

W--V------F-GHGTWY--GDPYGEGSSGMFNG-GNCGY-TKLAG-
TPYGPKIVAGSPAIYANGLGCGQCYQVKCTD--

----SKNGPR----L-CNP---SGTQVVVTDFCP----------GGTYCSTGEK-AFDFSGAALNAMAL-
PGKDGQLRNR

GLYNVLY-KRVPCKYKGQNIAFR--VDPGSSPY-WL---SVLIKYVGGPGDIGAVS--IKMAN--SYK-FM---
PMKHAW

GANWM-LQNYDGKPFKGPMDIKIVS--RLNGHSVVAKGVIPGYF--RPGTTYTSRV---QM

>CpEXPB5 [A]

W--V------Y-GHGTWY--GDPYGEGSSGM--G-GNCGY-TKLAG-
TPYGPKIVAGSPAIYANGLGCGQCYQIKCLD--

----SKYGPR----L-CNS---WGTQVVVTDFCP----------GGTYCSSGQK-AFDFSGAALNAMAL-
PGKDGQLRNR

GLYNVLY-KRVPCKYKGQNIAFR--VDPGSSPY-WL---SVLIKYVGGPGDIGAVY--IKMAN--SYR-FM---
PMKQAW

GANWM-LQNYDGKPFKGPMDIKIVS--RLNGHSVVAKGVIPGYF--RPGTTYTSRV---QM

>CpEXPB6 [X]

W--M------A-GHATWY--GEPNGEGSS----G-GACGY-TKLTG-
TPYGPRIAAGNDAIFQGGKGCGQCYEVKCSY--

--------AE------CRQ---EATRIVITDQCP----------GGEYCSKAEP-AFDLSGAAISAMAL-TDKDGALRNI

GLYDILY-KRVPCEFQGQNIAFQ--VDQGSTGF-WL---SFVVKFLGGPGDIDSVE--IRHSG--SSS-FQ---
PAKHNW

GASWM-LINTSGQAFKGPYDIRVVS--KLNGHAVTAEKVIPEFF--EPGKLYESNV---QL

>CpEXPB7 [A]

W--M------A-GHATWY--GDPYGEGSS----G-GACGY-TQLTG-
TPIGSKIAAGNNPIFQGGKGCGQCYEVKCNY--

--------PS------CNP---EPTRIVITDLCP----------GGTYCSTGNP-AFDFSGAAITAMAL-PGRDGELKKI

GLYDIQY-KRVPCEYQNQNIAFK--VDFGASAY-WL---SFTVKYLGGPGDIESVE--IRQVG--SNGSFQ---PCQHSW

GANWM-LINYTGQPFDGPYDVKITA--KLNGHSVIAYRAIPKWF--QPGALYNSNV---QF

>CpEXPB8 [ACnFn]

W--I------A-GHATWY--GDPYGEGSN----G-GACGY-TELAG-TPYGLSVAAGSAIIFQNGQGCGQCYDVKCSY--

--------PS------CKQ---EPTRVVITDSCP----------GGTYCSTGEP-AFDLSGMAMSNMAL-PGRDQELRNL

GLYDIQY-KRVPCYYPNQNIAFK--VDPGSTPY-WL---SFTVKYQGGPGDIQNVE--IRQAG--SSE-FQ---PLQHNW

GANYM-MICYSGQPFRGPYDVKITA--KLNGHVLIAEHAIPEYF--QPGGLYESNV---QL

>PpEXPB1PRO

W--L------G-GHATWY--GDPYGEGSS----G-GACGY-TQLTG-TPIGSKIAAGNAPIFQEGKGCGQCYEVKCNY--

--------PS------CSP---QGTRIVITDLCP----------GGQYCSTDQP-AFDFSGAAITAMAL-PGRDGELRNI

GLYDIQY-KRVPCEYPNQNIAFK--VDAGSSKY-WF---SFTVKYLGGPGDINTVE--VKCGK--NGY-FQ---YAQHSW

GANWM-LINYSGVPFQFPLTIKITT--KLNDHTVVAEDVIPDWF--GPGVQYESNV---QI

>PpEXPB2PRO

W--V------R-ARGTWY--GDPYGEGSS----G-GNCGY-TKLWG-TPIGPKIVAGSRSIYANGQGCGQCYQIRCVD--

----PNGGPR----L-CNP---QGTNVVVTDFCP----------GGTYCSTGEN-AFDMSGAAINAMAL-RGREGQLRNR

GLYNLLY-KRVPCRYRGTNIEFR--VDNGSSPF-WL---SILIKYVGGPGDIGQVY--IRMAN------WYKFQPMRHAW

GANWM-IPNYDGKPFRGPMDIRIVS--RLNRHTVLARGVIPAYF--RPGTSYRSRV---QM

>PpEXPB3PRO

W--L------P-GHATWY--GDPYGEGSS----G-GACGY-TELAG-TPYGLSVGAGSAVIYQNGQGCGECYEVKCTY--

--------PS------CKP---TPSRIVITDFCP----------GGTFCSTGEP-AFDLSGMAMTNMAL-PGRDQELRNL

GLYEIQY-RRVPCYYPNQNVAFK--VDPGSTPF-WL---SFTIEYQGGPGDIESVA--IRQAG--SSE-FQ---QMQHNW

GANYM-LICYSGKPFKGPYDVMITA--KLNGHVLIAQDAIPEYF--QPGGIYESNV---QL

>PpEXPB4PRO

W--V------P-GHATWY--GDPYGEGSS----G-GXCGY-LQLTG-
TPIGNKIAAGNEAIFQKGKGXXQCYEVKCNY--

--------PV------CRP---EGTRIVITDLCP----------GXQFCSGGNP-AFDLSGAAISAMAK-DGQDGALRNI

GLYDIQY-KRVPCEYPGQNIVFK--VDAGSSPF-WL---SFTVKYMGGPGDIESVS--ISQRD----GSFI---
PAQHSW

GANWM-LINYSGAPFQGPYSVKINC--MLNGHTVVAKDVIPAGF--APGQEYESNV---QI

>PpEXPB5PRO

W--M------A-GHATWY--GEPLGEGSS----G-GACGY-TKLAD-
TPYGPKIAAGNDPIFQGGSGCGACFEVKCNY--

--------PS------CKS---EPTRIIITDQCP----------GGTYCSTSQP-AFDLSGAAISDMAV-SGKDGELRNI

GLYDILY-KRVPCEYPNQNIAFQ--VDAGSSAF-WL---SLLVKYMGGPGDIESVE--IRTTG--SSS-FQ---
PAKHNW

GASWM-LINTSGQPFKGPYDVKIVS--KLNGHTVIAEKAIPEFF--EPGKLYESNV---QM

>PpEXPB6PRO

W--V------R-ARGTWY--GDPYGEGSS----G-GNCGY-TRLXG-
TPIGPKIVAGSRSIYXNGLGCGQCYQNQCVD--

----PXXGXK----L-XNS---FXTQXXXTDFCP----------GGXYCSTGEN-AFDMSGAAINAMAL-
PSREGQLRNR

GLYNLLY-KRVPCRYRGTXIXXR--VDXXSSAY-WL---SILIKYVGGPGDIGQVY--IKMANWFA---FQ---
PMKHAW

GANWM-MPSYDGKPFKGPMDIKIVS--KLNRHSVVARGVIPGYF--RPGTTYNSRV---QM

>AaEXPB1PRO

W--K------P-ATATWY--GAPDGAGTD----G-GACGY-GNLVG-
TPYGKRVTAGSPVLFKGGLGCGACYQVKCMI--

--------PK----L-C---AKKAVRVVITDECP-----------GGYCSGLNT-HFDLSGTAFSGMAN-
PGLGSSLLNL

GVLPMLY-KRVPCVYRR-PMKIE--VAAGSSPF-WF---SVLIKFVRGAGELKSVE--LQQGG--TNY-WY---
SMQHIW

GANWSVNVGS---GLKGPFTLRLTC--LLTGQQIVIRNVIPGNW--APATTYLASV---NF

>AaEXPB2PRO

W--L------S-CKATFY--GPQDGDGTD----A-GNCGY-GALAS-
SPYGKNIAAGSPELYQDGLGCGACFQIKCTN--

---------D----G-CKD---EASTVVITDQCP----------GGEWCGVGKP-HFDLRGTAFANLAV-
AGRSSDLFTR

GVLDLQY-QRVACSYGAQTVAFA--MDANCNPN-WI---AFVVEFQAGDGDLHSVE--LKDAS----
SDWQ---AMSHNW

GANWQ-FSGS---LTG-PISVRLTS--NTANQVLVAQDAIPANW--KPGQTYKSQD---QF

>AaEXPB3PRO

W--L------S-CKATFY--GPQDGDGTD----A-GNCGY-GALAS-
SPYGKNIAAGSPELYQDGLGCGACFQIKCTN--

---------D----G-CKD---EASTVVITDQCP----------GGEWCGVGKP-HFDLSGTAFANLAV-
AGRSSDLFTR

GVLDLQF-QRVACSYGAQTVAFA--MDANCNPN-WI---AFVVEFQAGDGDLHSVE--LKDVS----
SDWQ---AMSHNW

GANWQ-FSGS---LTG-PISVRLTS--NTANQVLVAQDAIPADW--TPGQTYQSQD---QF

>AaEXPB4PRO

W--L------S-CKATFY--GPQDGDGTD----A-GNCGY-GALAS-
SPYGKNIAAGSPELYQDGLGCGACFQIKCTN--

---------D----G-CKD---EASTVVITDQCP----------GGEWCGVGKP-HFDLSGTAFANLAV-
AGRSSDLFTR

GVLDLQY-QRVACSYGAQTVAFA--MDANCNPK-WI---AFVVEFQAGDGDLLSVE--LKDAS----
SDWQ---AMSHNW

GANWQ-FSGS---LTG-PISVRLTS--NTANQVLIAQDAIPANW--KPGQTYKSKQ---QF

>AaEXPB5PRO

W--L------S-CKATFY--GPQDGDGTD----A-GNCGY-GALAS-
SPYGKNIAAGSPELYQDGLGCGACFQIKCTN--

---------D----G-CKD---EASTVVITDQCP----------GGEWCGVGKP-HFDLSGTAFANLAV-
AGRSSDLFTR

GILDLQY-QRVACSYGAQTVAFA--MDANCNPN-WI---AFVVEFQAGDGDLHSVE--LKDAS----
SDWQ---AMSHNW

GANWQ-FSGS---LTG-PISVRLTS--NTANQVLIAQDAIPANW--KPGQTYKSQD---QF

>AaEXPB6PRO

W--L------A-CKATWY--GEQDGDGSD----G-GACGY-GELSS-
KPYGKNIAAGSPVLYQDGLGCGSCYQVKCTS--

-------DPA------C-S-GD-PVTVVLTDICP----------GGSLCGIGKP-HFDLSGTAMGNMAA-
AGKGSDLRGL

GVVDILY-KRVPCSYPEQNVTIA--VDQGSNPS-WL---ELTVLYEGGPGDLSALE--VKDAT----SQWQ---
PMVHKW

GANWGYDKAA---DLHGALSIRLTS--GLGSLPLVAQNALPADW--APGQTYQTVA---QF

>AaEXPB7PRO

W--V------A-CKATWY--GEPDGDGSDVNA-G-GACGY-GELSS-
KAYGKSIAAGSPVLYQDGLGCGSCYQVKCTS--

--------NP----A-C-S-GD-PVTIVLTDICP----------GGSLCGVGKP-HFDLSGTAIGNMAA-
AGKGSDLRGL

GVVDILY-KRVPCAYPEQNVTIA--VDQGSNPS-WL---ELTVLYEGGPGDLSALE--VKVTS--HSS--N---
PALACR

GSQI----------VVCVFAPELTTHAGLGT----AQDA-TSQWHRSGSRTLYIIP---SR

>AaEXPB8PRO

W--L------S-CKATFY--GPRDGDGTD----A-GNCGY-GALAS-SPYGSNIFAGSPELYQDGLGCGDAS--
RCTN--

---------D----G-CND--T-PTTVVMTDQCP----------GGEWCGIGKP-HFDLSGTAFGSLAA-
AGRSSDLFTL

GVLDIQY-QRVPCSYSTQTVAFA--MDANCNPN-WI---AFVVEFQAGDGDLHSVE--LKSRF--TSD-WQ-
--AMSHNW

XANWQ-FSGS---LQG-PIPVRLTS--NTANQVLVAKDAIPADW--TPGQTY-------QF

>AtEXPB2PRO

W--S------I-AGSTWY--GNPTGYGSD----G-GACGY-
GNAVAQPPFSKMVSAGGPSLFKSGKGCGACYQVKCTS--

--------KS----A-C---SKNPVTVVITDECP-------------GCVKESV-HFDLSGTAFGAMAI-SGQDSQLRNV

GELQILY-KKVECNYIGKTVTFQ--VDKGSNAN-SF---AVLVAYVNGDGEIGRIE--LKQAL--DSDKWL---
SMSQSW

GAVWKLDVSS---PLRAPLSLRVTS--LESGKTVVASNVIPANW--QPGAIYKSNV---NF

>AtEXPB3PRO

W--L------P-AVATWY--GSPNGDGSD----G-GACGY-
GTLVDVKPLHARVGAVNPILFKNGEGCGACYKVRCLD--

--------KS----I-CSR---RAVTVIITDECP-------------GCSKTST-HFDLSGAVFGRLAI-AGESGPLRNR

GLIPVIY-RRTACKYRGKNIAFH--VNEGSTDF-WL---SLLVEFEDGEGDIGSMH--IRQAG--ARE-WL---EMKHVW

GANWC-IIGG---PLKGPFSIKLTT--LSAGKTLSATDVVPRNW--APKATYSSRL---NF

>OsEXPB15PRO

W--S------N-GGATWY--GAANGAGSD----G-GACGYQGAVFQ-APFSSMIAAGSPSIYKSGLGCGSCYQVKCTG--

--------NS----A-CSG-N--PVTVVLTDECP-----------GGPCLSEPV-HFDLSGTAFGAMAN-PGQADQLRAA

GVLQIQY-NRVPCNWGGVKLTFV--VDVGSNPN-YF---AVLVKYENGDGDLSGVE--LMQTG--AGAAWT---QMQQSW

GAVWKLNAGS---ALQAPFSIRLTS--S-SGKTLVASNVIPSGW--KPGMSYISTV---NF

>OsEXPB16PRO

W--H------P-ATATWY--GSADGDGSD----G-GACGY-GTLVDVVPMKTRVGAVSPVLFKGGEGCGACYKVRCLD--

--------AS----I-CSR-R--AVTVIVTDECP-----------GGVCAFGRT-HFDLSGAAFARLAV-AGHGGQLQNR

GEISVVY-RRTACKYGGKNIAFH--VNEGSTTF-WL---SLLVEFEDGDGDIGSMQ--LKQAN--SAQ-WQ---DMKHIW

GATWS-LTPG---PLVGPFSVRLTT--LTTRQTLSAQDVIPKNW--TPKATYTSRL---NF

PC Distance Alignment

>SpmEXP1 [ABF]

V-------------LADDTIDADTTYYGGAN--AGGAQ----A-GACGY-GNLFTA-GYGTAITAVSTPLWKGGAACGAC

YE-L--SCKP------G---TS-G--CYN-T---KLVVTVTDQC---PD---------GGEWCQPDKPHFDLSQPVFAQL

AQ-------ISL-GHFPVTA-RRVTCPRTG-GVKVVA----QGNSN-WL---LVKFMNVAGGGTVNAAWVK-GSN--SD-

WI--PMSFNWGVGYQITGNI---PQG-PYSFQLRD--SYTGQVFTTDACLPSGW--NVGQTYSCDGNASPI

>SpmEXP2 [x]

W-------------ND-----AHATFYGGAD--ASGTM----G-GACGY-GNLYSS-GYGTDTAAASSALFNDGMSCGQC

YQ-L--QCVG----------SS-A--CKP-G---MITVTVTNLC---PNDFSKPSD--
AGGWCNYPRQHFDLSQPAYQKI

GV-------YGA-GIVPIQY-RRVPCLRTG-DIMFSM----GGNPF-YL---DVTIKNVGGSGDLSAVWVK-GNS-
-GQ-

WR--QLSQNWGMQWS-ADIA---VQGQALSFRLQS--QTTGQILTVENAVPSSW--SPGSTYQ----AGQF

>SpmEXP3 [x]

W-------------ND-----AHATFYGGAD--ASGTM----G-GACGY-GNLYSS-
GYGTDTAAASSALFNDGMSCGQC

YQ-L--QCVS----------SS-V--CKP-G---MITVTVTNLC---PNDFSKPSD--
AGGWCNYPRQHFDLSQPAYEKI

GV-------YGA-GIVPIQY-RRVPCLRTG-DIMFSM----GGNPF-YL---DVTIKNVGGSGDLDAVWVK-GSS-
-GQ-

WR--QLSQNWGMQWS-ADIA---VQGQALSFRLRS--QTTGQVLTVEDAVPSSW--SPGSTYQ----
AGQF

>SpmEXP4 [CBF]

W-------------QP---FNMTATFYGGPN--AENT-----G-GACGY-GDMFTA-
GFGTSTVAASSMLFKDGQSCGAC

YQ-V--RCVN----------AV-G--CIK-DA--YIQITVTNYC---PPRTDMS----
TPGWCDGDNMHFDMSQPAFTQI

AE-------YSA-GVVPVEV-MRVPCTRGG-NIQWQV----GGNPN-WL---QLNVRNIAGDGDIAAISVK-
GTN--TD-

WV--TMTQSFGVTWQ-ADGM---FQGQALSFRVTS--LLTGKVLQVDDAVPAGW--TVGSVYT----AGQY

>SpmEXP5 [x]

W-------------KT-----VTASFYGGAD--ASGNM----G-GACGY-GNDVSS-
TYGLYTAAASTALYSEGLACGQC

FQ-V--QCAN----------SK-A--CIA-GK--SVTVTVTNFC---PNNWALPSN--
AGGWCNFPRQHLDMSQPAFTQI

AQ-------YSA-GIVPVQI-RRVPCLRSG-SIAFGL----GGNTF-WL---QVNIRNVGGSGDISSVQVK-GNG-
--A-

WL--TMDHEWGQNWS-VDAK---LGGQSLSFRLTT--MTTGETLEIDNAAPSNW--QVGKTYQ----AGQF

>SpmEXP6 [CBF]

W-------------QP---FNMTATFYGGPN--AENT-----G-GACGY-GDMFTA-
GFGTSTVAASSMLFKDGQSCGAC

YQ-V--RCVN----------AV-G--CIK-DT--YIQITVTNYC---PPRTDMS----
TPGWCDGDNMHFDMSQPAFTQI

AD-------YSA-GVVPVEV-MRVPCTRDG-NIQWQV----GGNPN-WL---QLNIRNIAGDGDIAAISVK-
GTN--TD-

WV--TMTQSFGVTWQ-ADGM---FQGQPLSFRVTS--LLTGKVLQVDDAVPAGW--TVGSVYT----AGQY

>SpmEXP7 [ABF]

G-------------LADDTIDADTTYYGGAN--AGGAQ----A-GACGY-GNLFSA-
GYGTSITAVSTPLWKGGAACGAC

YE-L--SCKP------G---TS-G--CYN-T---KLVVTVTDQC---PD---------
GGEWCQPDKAHFDLSQPVFAQL

AQ-------ISL-GHFPVTA-RRVSCPRSG-GVKVVA----QGNSN-WL---LVKFMNVAGGGTINAAWVK-
GSN--SD-

WI--PMSFNWGVGYEITGNI---PQG-PYSFQLRD--SYTGQVFTTDACLPSGW--NVGQTYSCDGNASPI

>SpmEXP8 [x]

W-------------KT-----VTASFYGGAD--ASGNM----G-GACGY-GNDVSS-
TYGLYTAAASTALYSEGLACGQC

FQ-V--QCAN----------SN-A--CIV-GK--TVTVTVTNFC---PNNWALPSN--
AGGWCNFPRQHLDMSQPAFTQI

AQ-------YSA-GIVPVQV-RRVPCLRSG-SITFGL----GGNTF-WL---QVNIRNVGGSGDISSVQVK-GSG-
--A-

WL--TMDHEWGQNWS-VDAK---LGGQSLSFRLTT--MTTGETLEVANAAPSSW--QVGKTYQ----AGQF

>SpmEXP9 [x]

W-------------ND-----AHATFYGGAD--ASGTM----G-GACGY-GNLYSS-
GYGTDTAAASSALFNDGMSCGQC

YQ-L--QCVG----------SS-A--CKP-G---MITVTVTNLC---PNDFSKPSD--
AGGWCNYPRQHFDLSQPAYEKI

GV-------YGA-GIVPIQY-RRVPCLRTG-DIMFSM----GGNPF-YL---DVTIKNVGGSGDLDAVWVK-GSS-
-GQ-

WR--QLSQNWGMQWS-ADIA---VQGQALSFRLRS--QTTGQILTVENAVPSSW--KPGSTYQ----AGQF

>SpmEXP10 [ABF]

V-------------LADDTIDADTTYYGGAN--AGGAQ----A-GACGY-GNLFSA-
GYGTSITAVSTPLWKGGAACGAC

YE-L--TCKP------G---TS-G--CYN-T---KLVVTVTDQC---PD---------
GGEWCQPDKPHFDLSQPVFAQL

AQ-------ISL-GHFPVMA-RRVSCPRSG-GVKVVA----QGNSN-WL---LVKFMNVAGGGTINAAWVK-
GSN--SD-

WI--PMSFNWGVGYQITGNI---PQG-PYSFQLRD--SYTGQVFTTDACLPSGW--NVGQTYSCDGNASPI

>SpmEXP11 [CBF]

W-------------QP---FNMTATFYGGPN--AENT-----G-GACGY-GDMFTA-
GYGTSTVAASSMLFKDGQSCGAC

YQ-V--RCVN----------AV-G--CIK-DT--YIQITVTNYC---PPRTDMS----
TPGWCDGDNMHFDMSQPAFTQI

AD-------YSA-GVVPVEV-MRVPCTRDG-NIQWQV----GGNPN-WL---QLNIRNIAGDGDIAAISVK-
GTN--TD-

WV--TMTQSFGVTWQ-ADGM---FQGQALSFRVTS--LLTGKVLQVDDAVPAGW--TVGSVYT----AGQY

>SpmEXP12 [x]

W-------------KT-----VTATFYGGAD--ASGNM----G-GACGY-GNDVSS-
TYGLYTAAASTALYSEGLACGQC

FQ-V--QCAN----------SN-A--CIA-GK--TVTVTVTNFC---PNNWALPSN--
AGGWCNFPRQHLDMSQPAFTQI

AQ-------YSA-GIVPVQV-RRVPCLRSG-SIAFGL----GGNTF-WL---QVNIRNVGGSGDISSVQVK-
GSG--A--

WL--AMDHEWGQNWS-VDAK---LGGQSLSFRLTT--MTTGETLEIDNAAPLSW--QVGKTYQ----AGQF

>CbEXP1 [x]

W-------------QD-----GRATYYGSEAFGGDNEH----N-GACGY-GTLSAA-
FVGHNYAAASPVIFESGRACGGC

FE-V--QCRG------N---GY-P--CRA-GT---ARVTVTDLC---PPIPP------NLRHCAGGKAHLDLGVRVFPII

AD-------PRA-GVVDIRF-RTVPCGRFN-AITFFI----RGNVYGWL---SLVVMGVPASGRVVGMEVK-
ASG--STN

WE--ALRHDYGAAYMRTGRP---LRT-PISVRLSI--FGKLCRMTAMNCIPTGF--KDRQKVS-----CSY

>CbEXP2 [H]

W-------------IN-----GHSTFYDDHG------R----G-GACGY-GALGSQ-
LYGTRFAAGSPRVYLGGLACGMC

LE-V--RCVG----------SR-A--CRR-S---SVRLTITDLC---PPGYP------NTQWCGGGKLHLDIGKDAFPII

AD-------PRA-GHVPIQF-RSVPCAPYK-VLKLNL----RGHRW-WL---EVTALAIPGSGRVLKMEVA-GKG--GR-

WV--SMKRAFGAAWSTAGST---LQT-PISVRISI--FGKLCKLIAKDCINGQI--YNGRQLS-----CAY

>CbEXP3 [H]

W-------------IS-----GHSTFYDDHG------R----G-GACGY-GALGSQ-LYGTRFAAGSPRVYLGGLACGMC

LE-V--RCVG----------SR-A--CRR-S---SVRLTITDLC---PPGYP------NTQWCGGGKLHLDIGKDAFPII

AD-------PRA-GHVPIQF-RSVPCAPYK-VLKLNL----RGHRW-WL---EVTVLAIPGSGRVLKMEVA-GKG--GR-

WV--SLKRAFGAAWSTAGST---LQT-PISVRISI--FGKFCKLIAKDCIKGQI--YNGRQLS-----CAY

>CbEXP4 [H]

W-------------IN-----GHSTFYDDYG------R----G-GACGY-GRLGSQ-LYGTRFAAGSPRVYLGGLACGMC

LE-V--RCVG----------SR-A--CRR-S---SVRLTITDLC---PPGYP------NTQWC---QIHLDIGKNAFPVI

AD-------PRA-GHVPIQF-RTVPCAPYK-VLKLSL----RGHRW-WL---EVTALAIPGSGRVLKMEVA-GRG--GR-

WV--SLKRAFGAAWATAGAT---LQT-PISVRISI--FGKLCKLIAKDCIKGQI--YNGRQLS-----CAY

>CbEXP5 [H]

W-------------LN-----GHSTFYDDYG------R----G-GACGY-GALGGQ-LFGTRFAAGSPSVYLGGLACGMC

LE-V--RCVG----------ST-A--CRR-S---SVRLTITDLC---PPGYP------NTQWCGGGKLHLDIGKDAFPII

AD-------PRA-GHVPIQF-RSVPCAPYK-VLKLNL----RGHRW-WL---EVTALAIPGSGRVLKMEVA-GKG--GP-

WV--SMTRAFGAAWSTAGST---LQT-PISVRISI--FGKLCKLIAKDCIDGQI--YNGRQLS-----CAY

>CbEXP6 [H]

W-------------IN-----GHSTFYDDHG------R----G-GACGY-GPLGSQ-LFANRFAAGSPRVYLGGLACGMC

LE-V--RCVG----------SR-A--CRR-S---SVRLTVTDLC---PPGYP------NTQWCGGGKLHLDISKNAFPII

AD-------PRA-GHVPIQF-RSVPCAPYR-ILRLNL----RGHMW-WL---EVTALAIPGSGRILRMEVA-GRG--GR-

WR--SMKRSFGAAWSTTGAS---LRL-PISVRISI--LGKFCKLIARDCIKGQI--YNGKQLS-----CRY

>CbEXP7 [H]

W-------------LS-----GKATFYSGGD------Q----G-NACGY-GRLTGA-
IFGRNFAAVSPRIYLGGLACGMC

LE-V--KCVG----------HP-G--CRR-G---VARVTVTDLC---PARPP------
NMAFCAGGKLHLDLGRDVFPVI

CN-------PRV-GVAPIRF-RAVPCARYR-TLNIIL----NGNRYGYM---AIVVMSIPGSGRVIRMDVR-GRG--
GRN

WE--PLRRDWGATFMRTGRP---LRY-PVSIRVSI--LGKLCRLIARNCIKGQF--FNGRKLS-----CSY

>CbEXP8 [AB]

W-------------TE----GATLTFYGDAN--GGGTL----A-GACGY-GNLLAT-
PYKLMIAAGNPPMFEEGLGCGRC

WE-I--RCVG----------NP-G--CTA-----TVTITMTDLC---P--------------CGSDGVHFDMSQPAFHAL

ASGPAEAEQLVTAGKLQGQY-RRVPCDLGL-GKAFTAIVRAATNPS-FL---
SILMRDIPGAGDIKTAECAPGGG--ST-

FS--PMTQDFGAMYN-FNGK---WTS-PVSLRLTL--G-DGQILTGISCIPAAW--KPTTTYT-----CK-

>CbEXP9 [H]

W-------------QS-----GTGTFYSGGH-----------G-GACGF-AGLGYS-
IFAENYVAVSTALWNSGAACGTC

LE-V--RCKG------P---SS-A--CIA-GA--TIKVTVTDFC---PNTYP------NLAHCGTDKHHLDLGENVFPKI

AA-------RSV-GVIDLEY-RRVPCVQAP-SITVSV----EGNQYGYL---ALNLMRLPGAGTVAQLEIK-GAN--
GA-

YE--QCRRDWGNTFVRTGKP---IEA-PLSLRVSV--G-GICTFEAKDCIKSL---NFGSSVT-----CSY

>CbEXP10 [H]

W-------------IN-----GHSTFYDDFG------R----G-GACGY-GRIGSQ-
LYRNRFAAGSPRVYLGGLACGMC

ME-V--KCVN----------SP-A--CRP-G---SVRVTITDLC---PPKYP------
NLRWCTGGKIHLDMARGAFPVI

AN-------PGA-GHVPIQF-RSVPCAPFR-LLKFTL----RGNRWGWL---EVTTLTIPGSGRVLRLEYA-
MKG--GA-

WT--GMSRAFGAAWA-TKGK---FLRFPISIRIAV--FGKFCKLVAKDCIKGQF--WNGQQLS-----CSY

>CbEXP11 [x]

W------------TEG-----ATLTFYGDAN--GGGTL----V-GACGY-GNLLAT-
PCKLMIAAGNPPMFEEGFGCGRC

WE-I--RCVG----------NP-G--CTA-----TVTITMTHLC---P--------------CGSDTVHFDMSQPAFHAL

ASGPAEAEQLVTAGKLQGQY-RRVLCDLGL-GKAFTAIVREATNPS-FL---
SILMRDIPGAGDIKTAECAPGGG--ST-

FS--PMTQDFGAMYN-FNGK---WTS-PVSLGLTL--G-DGQILTGISCIPAAW--KPATTYT-----CKX

>CbEXP12 [H]

W------------TG-----GTATFYSGGR-----------G-GACGF-GGLGYA-
IYGERYAAASMKIWQNGAACGMC

VE-I--KCMG----------PS-G--CYA-GK--IVKVTITDFC---PNTHP------
NLAHCGPEKNHIDMGQNSFPVI

AN-------PRV-GVINIQY-RVVPCNAYG-NFQVQI----NGNQYGWL---SLLIAQIPGSGRVVGCEVQ-
GAG--MNY

WE--GLRRVWGSNWMRTGTP---LKT-PIHVRLSL--LGQICKVTSRNCIPNGF--YFGQAVK-----CNL

>CbEXP13 [H]

W------------TS-----GTATFYSGGS-----------G-GACGF-GGLGYA-
IYGERYAAASMRIWQNGAACGLC

VE-I--RCTG----------PS-G--CYP-GK--VVKVTITDFC---PNTPP------
NLAHCGPDKNHIDMGQNSFPVI

AD-------ARV-GVIDIEY-RTVSCTPYS-NFQVQI----NGNQFGWL---SLLIAQIPGSGRVVGCEVQ-GSG-
-NDN

WE--ALKRTWGSNWVRTGTP---LKA-PIHVRLTL--WGQICKVTSHNCIPSGF--SFGQVVQ-----CNL

>CbEXP14 [H]

W------------TA-----GTATFYSGGR-----------G-GACGF-GGLGYA-
IYGERYAAASMKIWQNGAACGMC

VE-I--KCMG----------PK-G--CYA-GK--VVKVTITDFC---PNTPP------
NLAHCGPEKNHIDMGQNSFPVI

AN-------PKV-GVINTQY-RVVPCNPYT-TFQVQV----NGNQFGWL---SLLIAQIPGSGRVVGCEVQ-
GTG--MNY

WE--GLKRTWGSNWVRTGKP---LKA-PINVRLSL--LGQICKVTSRNCIPSGF--YFGQVVK-----CNL

>CbEXP15 [x]

W------------IS-----GHATFYDDHG------R----G-GACGY-GRLGSE-
LFGGRYAAGSPRVYLGGMACGMC

LE-V--KCVG----------VP-A--CKP-A---TVRVTVTDLC---PDRPP------
NTQWCGGGKLHLDMATAAFPVI

AN-------PRA-GHVPIQF-RSVPCAPYK-MLKLHL----RGNMFGWL---EVTALGIPGSGKVLKMEVA-GSD--GS-

WV--VMKRAFGAAWAVAGRP---MRS-PISVRIAI--FGKLCKLVAKNCISGQN--FNGVDLS-----CAY

>CbEXP16 [H]

W-------------IN-----AHATFYDDGG------R----G-GACGY-GSLGSQ-LFSNRFAAGSPAVYLGGLTCGMC

VE-V--KCVE----------SP-A--CKA-G---SVRLTITDLC---PPGAP------NTQWCGGGKRHLDLAKGAFPVI

AN-------PGA-GHVPIQF-RTVPCAPYN-VLTLNL----RGHQW-WL---EVTAVGIPGSGRILRVEVA-GNG--SP-

WT--VMKRAYGAAWATAGST---LRS-PISVRVSI--FGNLCKLTARDCIKGPI--YNGRKVT-----CGY

>CbEXP17 [H]

W-------------IN-----AHATFYDDGG------R----G-GACGY-GSLGSQ-LFSNRFAAGSPAVYLGGLTCGMC

VE-V--KCVG----------SR-A--CKA-G---SVRVTITDLC---PPGAP------NTQWCGGGKRHLDLAKGAFPVV

AN-------PGA-GHVPIQF-RTVPCAPYK-VLTLNL----RGHQW-WL---EVTAVGIPGSGRILRVEVA-GNG--SP-

WR--VMKRAYGAAWATAGSA---LRS-PISVRISI--FGNLCKLTARDCIKGPI--YNGRKVT-----CGY

>CbEXP18 [H]

W-------------IN-----AHATFYDNGG------R----G-GACGY-GSLGSQ-LFSNRFAAGSPAVYLGGLTCGMC

VE-V--KCVG----------SR-A--CKA-G---SVRVTITDLC---PPGAP------NTQWCGGGKRHLDLAKGAFPVI

AN-------PGA-GHVPIQF-RTVPCAPYK-VLTLNL----RGHQW-WL---EVTAVGIPGSGRILRVEVA-GNG--SP-

WR--VMKRAYGAAWATAGST---LRS-PISVRISI--FGNLCKLTARDCIKGPI--YNGRKVT-----CGY

>CbEXP19 [F]

W-------------RG-----GTATCYTGGR-----------G-GACGF-SGALAL-IYGQSYAAVSTRLWENGKVCGQC

YE-V--RCVG----------SG-G--CRR-G---SVKVTVTDFC---PDTQA------NRRHCGATKQHIDLGRNAFPVI

AN-------PSV-GVINVQF-RTVPCAAYH-LLRISV----RGNRNGWLSKGLFLTNENRKADRVFAVEVK-
GRY--RNY

WE--RMTRTWGATYDRRGRP---LVG-PISVRVSL--FARFCKVVATNCIPAKW--YSGSTYH-----CTF

>CbEXP20 [B]

W-------------------SAVLYICISA-----------G-GACGY-GPTLSQ--YGPYLTALSVSLYQTGLACGGC

FE-V--KCVD----------SP-H--CNS-GK--VVKVTAVDEC---P----------EGGYCVTGKMHFDLSQPAFKTI

GN-------QQA-GVIPILWRRRVQCERNG-AIELHM----QGGPY-WL---SILVKNTGGDGGVKSVQVK-
PSD--KNE

WY--QMTHVWGQKWD-LAGPMMPLKG-PLSIKLIS--LYSGQVATGWNCISQQW--QSGQTYF---
ISQQF

>CbEXP21 [AB]

W-------------ST-----GRATFYGAPD--GSGLD----G-GNCGF-GPLANS--
YGRYVAAISISLYQNGKGCGGC

YE-V--KCVD----------SQ-W--CTG-K---SVIISATDQC---P----------DGGYCDNNQAHFDMSHPAFEMI

AQ-------KIA-GVIPVSW-RRVPCVRTG-PIQFVI----NGNPF-WF---TIMPKNVAGAGDVKSVEVQIGGR-
----

WV--PMIQLWGQNWLLSGMG---QVNGAISVRVTT--LLTGEVITVPNVIPSGW--KSGTPYT--SGMVNF

>CbEXP22 [x]

W-------------IN-----GQATFYDDHG------R----G-GACGY-GSLGSQ-
LFGGRYAAGSPRVYLGGLACGMC

FE-V--KCVR----------ST-A--CKA-G---SVRVTITDLC---PPRAP------
NSRWCGGGKLHLDMGRQAFPVI

AN-------PRA-GAVPIQF-RSIPCAPYK-VLKLHL----RGSRF-WL---EVTALAIPGSGRILKMEVA-AAG--
GK-

WV--AMKRAFGAAWAVTGQP---MRT-PISVKISI--FGKLCKLIARNCIKGQS--FNGQNLN-----CGY

>AaEXPA1PRO

W-------------QD-----GHATFYGGSD--ASGTM----G-GACGY-SNLYSQ-
GYGLATAALSTALFNNGLSCGAC

FE-I--KCNQ----------PE-W--CVA-GAS-SIVVTATNLC---PPNPSLPND--
NGGWCNEPLQHFDMSQPAFQQI

AQ-------YKA-GIVPVSY-RRVSCVKKG-GVHFTV----NGHSY-FN---LVLVSNVGGAGDVHAVSIK-GSN-
-TG-

WE--PMSRNWGQNWQ-SNSK---LDGQSLSFQVTT--S-DGQTLTFYDVASDNW--SFGQTFE---GDSNF

>AaEXPA2PRO

W-------------SS-----AHATFYGGND--ASGTL----G-GSCGY-GNLYDR-GYGTSTAALSTALYNNGASCGAC

FE-I--QCTG----------SR-W--CKG-SA--PITVTATNFC---PQNPALPNN--AGGWCNLPLQHFDMSVPAFLEI

AE-------QVG-GIVPVNY-RRVSCVKKG-GIHFTI----NGHAY-FN---LVLITNVGGAGDVHAVSVK-GSK--TG-

WE--PMTRNWGQNWQ-CNSY---LNGQSLSFQVTT----SGGTLISYDVTPAGW--SFGQTFS-G---AQF

>AaEXPA3PRO

W-------------NS-----AHATFYGGND--ASGTM----G-GACGY-GNLYSE-GYGTNTAALSTALFDNGLSCGAC

YE-I--ACDS--SG------TK-Y--CTG--SN-SITITATNFC---PENLALPSD--NGGWCNQPRQHFDMAVPAFDQL

AA-------HVA-GIVPIKY-RRVSCVRKG-GVRFHL----NGHDY-FL---LLLVDNVGGNGAVHSVSIK-AGN--GP-

WK--ALSQNWGQNWQ-CNDY---ISGQTLSISITT--E-DGKTLVSNNVAPAGW--KFGETYA-G---SQY

>AaEXPA4PRO

W------------VKG----VAHATFYGGSD--ASETN----G-GACGY-GNLYSQ-GYGTNTAALSSALFGGGETCGAC

YQ-L--KCTG-------------G--CFS-GAP-KIVVTATNYC---PEGS-------TGGWCNAPRVHFDLSEPAFTRM

AA-------AVT-GVVPVNY-RRVKCVKEG-GIKFQI----NGHSY-FN---LILITNVGGVGDVKEVYIQVGSG--SS-

WK--ACKRNWGQNWE-NDDY---LDGKSFSIKVIT--S-DNRFCNSPNVIPAGW--KFGTTYE-G---RQF

>AaEXPA5PRO

W-------------KA-----AHATFYGGND--ASGTN----G-GACGY-GNIYSF-GYGIANAALSYALFNSGAQCGAC

YE-I--KCYG----------SK-W--CLP-GNP-SITITATNSC---PPNWALPSD--NGGWCNPPREHFDMSVPAWTSL

AV-------YVG-GIVPVNY-RRVPCSRKG-GIRFTI----SGNPN-FD---LVLVHNVAGDGVVTALSIK-GNG----N

WV--PMKRNWGQNWQ-CGAV---LTGQPLSFMVRT--S-DGRSLILYNVAPANW--SFGSTYQ---
SAQNF

>AaEXPA6PRO

--------------ND-----AHATFYGDPS--GSATF----G-GACGY-GDLMAQ-
GYGLQTTALSEVLFSNGFSCGQC

YQ-I--QCID----------SK-W--CIP-NA--VITVTATNLC---PADYSKPND--
NGGWCNPPLKHFDLSMPAFHQI

AQ-------EVA-GIVPVTY-RRVPCVKLG-GVKFTI----LGNTW-WH---LVLITNVGGAGDIDTASIK-GSN--
TD-

WI--PMSHNWGASFQ-CFQS---LVNQALSFQLTS--T-TGQVLTTFNVAPADW--SFGTTYE-G---GQY

>AaEXPA7PRO

W-------------TTG----ATATYYGGSD--ATATN----G-GACGY-GNQIAL--
YGTVSTALSSAFFQNGVTCGAC

YE-I--QCTG----------SS-Y--CYS-GK--TVKVTATNLC---PDGS-------
YGGWCDGSKKHFDLSQPAFIQV

AQ-------QVV-GHFAAQY-RRVPCTRSG-GVRFTI----NGNTY-YN---LVLIQNVAGVGDVKAVSIK-GTS--
TG-

WL--SMSRNWGENWE-CDTK---LVGQALSFQVTL--S-DSTTKTFNNIAASNW--AFGSTYS---SSVQF

>CpEXPA1 [AB]

W-----------IGGN-----AHATYYGGVD--ASGTQ----G-GACGY-GNLYST-
GYGTNTAALSSALFNSGLTCGAC

YE-L--QCDS----T-G---SK-Y--CLP-GNP-SITITATNYC---PQN-----S--
LGGWCDAPRQHFDLAHPMFVSL

AQ-------EGG-GVIPVNY-RRVPCVKKG-GMRFQL----NGNPW-FV---LVLVTNVGGAGDVQQLYIK-
GDK--TG-

WY--PMKRNWGQMWQLTGNS--NMPGQALSFRAVL--S-DGTNVESMDVAPANW--RFSQLFE-G---
SQA

>CpEXPA2 [X]

S-------------GT-----GHITFYGTPN--GGGQA----SRGACGY-QNTYAL-
GYGSMTAALSSPLYQGGAACGAC

FQ-L--TCMRIRETKTA---KN-W--CWS-YSR-SITVTATNLC---PPG-----S--
RDGWCNPPRNHFDLPYPAYLSL

AK-------REG-GVAPVTW-RRVPCRKRG-GIRFTV----GGNPW-FF---MILIHNVGGAGDVRSVKIG-CPY--TG-

WV--PMYRNWGALWT-VRRQ---MRG-QLSFLITT--S-DGRSVVARNAVGKYW--KFGQTWE---CGAQF

>CpEXPA3 [X]

S-------------GT-----GHITFYGTPN--GGGQA----SRGACGY-QNTYAL-GYGSMTAALSSPLYQGGAACGAC

FQ-L--TCMRIRETKTA---KN-W--CWS-YSR-SITVTATNLC---PPG-----S--RDGWCNPPRNHFDLPYPAYLSL

AK-------REG-GVAPVTW-RRVPCRKRG-GIRFTV----GGNPW-FF---MILIHNVGGAGDVRSVKIG-CPY--TG-

WV--PMYRNWGALWT-VRRQ---MRG-QLSFLITT--S-DGRSVVARNAVGKYW--KFGQTWE---CGAQF

>CpEXPA4 [AB]

W-------------GK-----GKCTFYGGMD--AAGTM----S-GACGY-GNLYAS-GYGAYTAALSSALFKNGMACGAC

YE-V--ACSG------N---AK-S--CKR-G---SVLVTITNFC---PPNPGRSAN--NGGWCNGANQHFDLSYPAFAKI

AD-------PKA-GVVDLNY-RRVPCQKKG-GIRFTL----NGNCN-FN---IVTITNVGGSGVVTQAYMK-GDG--MD-

WA--PMTRNWGANWQ-CSRN---YCGKGISIKIVT----SDKKVSVSRLADKTW--AFGKTYI--GQQVNX

>CpEXPA5 [AB]

W-------------NS-----AHATYYGGAD--ASGTQGERRG-GACGF-GNLYST-GYGTSTAALSQALFNSGLTCGAC

FE-L--ACDS------SG--SK-Y--CEG-AP--SVVVTATNFC---PSGS-------NGGWCDQPKQHFDLAMPVFDKI

AQ-------EVG-GVIPVKY-RRVPCQKKG-GMRFTI----NGNPY-FI---LVLVTNVAGAGDVQQISIK-GDN--TD-

WF--AMKRNWGQQWQFSGNS--ALVGQALSFKVVT--S-DGAVATSMNAAPANW--QFSQTFE-G---VNY

>CpEXPA6 [B]

W-------------RG----GGHITFYGGPN--GAGTQ----A-GACGY-QNTYKL-GYGSMTAALSSPLFQGGAACGAC

FQ-L--RCAPVRRSRTA---RN-W--CYS-YAK-TITVTATNLC---PSG-----S--
TGGWCNPPRAHFDLPMPAFLTL

AR-------REG-GVAPVFY-RRVRCAKRG-GIRFTI----GGNPW-FL---MVLIHNVGGAGDVRSVRIK-GQY-
-TG-

WY--PMYRNWGALWT-VRTK---LSG-ALTFAVTT----SDRRTVITRSVGGNW--RFGQTWE-G---ANF

>CpEXPA7 [B]

W------------KGG-----AHITFYGSPN--GGGTQ----A-GACGY-QNTYKL-
GYGSMTAALSSPLFRGGSACGAC

YQ-I--RCAPVRRTRTA---RN-W--CYS-YAR-TITITATNLC---PPG-----S--
TGGWCNPPRAHFDLPMPAFLTL

AR-------REG-GVAPVYY-RKVRCAKRG-GIRFTI----GGNPW-FL---MVLIHNVGGAGDMRSVRVK-
GQY--TN-

WY--PMYRNWGALWT-VRTK---LSG-ALSFAVTT--S-NGRTVVARNVVGKWW--KFGQTWE---ARTNF

>CpEXPA8 [X]

W-------------GY-----AHITFYGTPN--GGGQA----SRGACGY-QNTYAL-
GYGAMTAALSSPLYKGGAACGAC

FE-L--KCIRIRESRSA---KN-W--CWS-YSR-SITITATNLC---PPG-----S--
TGGWCNPPKHHFDLSYPAYATL

AR-------REG-GVAPVNY-RRVPCRKRG-GVRFTV----GGNPW-FF---MILIHNIAGAGDVRSVKIM-
CPY--TG-

WV--PMYRNWGALWT-VRKQ---MRG-PLSFAITT--S-DGRTIISRNAVGNYW--KFGQTWE---GRAQF

>CpEXPA9 [AB]

W-------------NT-----AHATFYGGSD--AGGTE----G-GACGY-GDLYSS-
GYDTNTVAISSALFDRGLACGAC

YQ-V--KCAG--KA-------S-E--CHP-DSP-SIQVTVTNFC---PPNPSLPDN--
NGGWCNLPLHHFDMSEPAFQRI

AT-------YKS-AIVPVMY-RRTACMKSG-GIRFTM----SGHNF-MN---LVLVTNVGGMGDVQSVYIR-GSK-
-TK-

WV--AMARNFGQIWQ-STVN---MSGQSLSFMVTT--S-DGQTVVSNEVAPPDW--AFGQTYE-G---SQF

>CpEXPA10 [AB]

W-------------RK-----AHATFYGGAD--ASGTM----G-GACGY-GNLHST-
GYGLDTTALSTALFNNGAKCGAC

FA-I--QCYR----------SK-Y--CLP-GTP-IITVTATNFC---PPNRQGDG---
TPGWCNPPLRHFDLAQPSFVKV

AK-------YRA-GIVPVLF-RRVPCQKKG-GVRFTL----NGNKY-FN---LVLVHNVGGKGDVRSVDIK-GSD-
-TE-

WM--PMKRNWGMNWQ-THTV---MVGKALSFRVTT--S-DGVTVTSMDAAPPQW--RFGQTFE-G---
AQF

>CpEXPA11 [AB]

W-------------SP-----AHATFYGGAD--ASGTQ----G-GACGY-ENLYNQ-
GYGINTAAVSAPLFNDGAGCGAC

FE-L--VCDS----S-Q---SD-Y--CYS-NAS-SIIVTTTNFCPKAPEG-----T--
ENAWCDPPRIHFDLSEPIFLTF

AQ-------KVA-GIVPVNY-RRVPCLKDG-GIKFTI----NGHKY-FF---LVLVTNVAGAGDIQELYIK-ASN--
TD-

WQ--PLTRNWGDNWE-LNQNTLNFVGQALSFKAVA--S-DKSEVVSMNVTPVDW--EFSQTFE-G---
SNF

>CpEXPA12 [A]

W-------------TS---GQAHATYYGGAD--AAGTQ----G-GACGF-GNLWST-
GYGTDTAALSAALFNSGLSCGAC

YE-L--KCDT----A-G---SK-Y--CLP-GNP-SITVTATNYC---PQG-----S--
EGGWCDTPKQHFDLAHPAFTKL

AH-------EGG-GVFPVVY-RRVPCAKQG-GMRFQI----NGNPW-FL---LVLVTNVGGAGDVQQLQVK-
GER--TG-

WY--QMVRNWGQMWQFTGDH--TLPGQALSFRATL--S-DGSVVESWNCAPKNW--GFQQVFE-G---
TPA

>CpEXPA13 [X]

W-------------RD----DGHITYYGSPN--GHGTQ----G-GACGY-QNTFSL-
GYDHLTAALSTPLFQGGAACGAC

YE-L--RCKFIKESKTA---KN-W--CWN-YSR-SITSTATNLC---PPG-----S--
TGGWCDPPRNHFDLPMPAFLTL

AR-------QNG-GVAPVHY-RRVPCQKKG-GIQFTI----GGNPW-FY---MILIHNVAGAGDVVRVQIK-SPT-
-TG-

WT--PMYRNWGALWT-VRKQ---LKG-ALSFQITT--S-DGRVVVTHNAVGHGW--RFGQTWE-G---AQY

>CpEXPA14 [AB]

L-------------GR-----AHATYYGGPD--GQGTM----G-GACGY-GNLYNT-
GYGLSTAALSAPLYNGGRACGAC

YR-L--TCDT----S-G---SK-Y--CYG-GGK-SITITVTNFC---PSG-----S--
TGGWCNPPKQHFDLSYPMFTTL

AQ-------AAG-GVIPVNY-RRVACNKKG-GMRFTI----SGNPY-FY---QVLVFNVGGAGDVQRLFMK-
GPR--TG-

WI--AMKRNWGQVWQYRGGP-KGLVGQPLSFRAHT--S-DGRYVISPNATPANW--RFGQTFE-G---
KNF

>CpEXPA15 [AB]

W-------------GY-----AHATYYGGAD--ASGTQ----G-GACGF-GNLYST-
GYGTNTAALSAALFNTGLSCGSC

YE-L--ACDP----N-G---SK-Y--CLP-GGR-TVTVTATNFC---PHG-----S--
LGGWCDSPKQHFDLAHPMFVSL

AK-------EVG-GVIPIKY-RRVPCIKSG-GMRFTL----NGNPW-FI---LVLVTNVAGAGDVQQLYIK-GSR--
TQ-

WE--RMSRNWGSMWQFTGNS--NMKGQALSFKAIT--S-DGSVAISTDAAPGNW--QFGQTFE-G---
SNF

>CpEXPA16 [AB]

W-------------DN-----GHATFYGGDN--GAGTM----G-GACGY-GNLYNT-
GYGLKTAALSAPLFNGGRACGAC

YQ-L--TCVT----S-G---TK-Y--CYG-GGK-SITITATNFC---PTG-----S--
TGGWCNPPKKHFDLSYPMFAVL

AK-------KVG-GVIPVNF-QRVPCSRKG-GMRFTI----NGNPY-FF---LVMVYNVAGAGDVQRLFIK-GPS-
-TG-

WF--EMKRNWGQVWQYNGGP-KGMVGQALSFRAIT--S-DGRQVISTNAAPGNW--RFGQTFE-G---
RNX

>CpEXPA17 [AB]

W-------------GQ-----AHATYYGGAD--ASGTQ----G-GACGF-GNLYNT-
GYGTNTAALSAALFNSGLSCGAC

YE-L--TCDP----S-G---SQ-Y--CLP-GGS-AI-ITATNFC---PTG-----S--
NGGWCNTPKQHFDLAQPVFSKI

AR-------TVG-GVIPINY-RRVSCSKSG-GMRFTI----NGNPY-FL---LVLVTNVGGAGDVQQLYIK-GAS--
TN-

WL--PLKRNWGQMWQFTGNS--GMHGQAISFKAVT--G-DGATAVSDNVAPPNW--GFGQTFE-G---ANF

>CpEXPA18 [B]

W------------RTD-----GHITFYGSPN--GGGTQ----A-GACGY-QNTYAL-GYGSMTAALSTALFQGGAACGGC

FE-I--KCIP---PRSG---KN-W--CFS-YAK-SIIVTATNLC---PPG-----S--TGGWCNPPRAHFDLPMPAFLTL

AQ-------KTG-GVAPVAF-RRVRCLKKG-GVRFTV----GGNPY-FL---MVLISNVGGVGDIRSVKIK-GSY--TN-

WI--PMYRNWGSLWT-VRSK---ITG-ALSFLITL--S-DGSTLPCTNAVQANF--RFGQTWE---ARANL

>CpEXPA19 [X]

W-------------KD----DGHITFYGSPN--GGGTQ----A-GACGY-QNTYKL-GYGSMTAALSTPLFQGGLACGAC

FE-L--RCKFIRETKSA---KN-W--CYN-YSR-SIIITATNLC---PPG-----S--TGGWCDPPRKHFDLPMPAFETL

AK-------RVG-GVAPVYH-RRVPCQKKG-GIRFTM----AGNPW-FF---MILIHNVGGAGDVVAVKIK-DPS--SA-

WA--PMYRNWGAIWT-VRKQ---MKG-ALSFQITT--G-DGRTVTTNNAVGNGW--KFGQTWE-G-AQMRF

>CpEXPA20 [X]

W-------------KD----DGHITFYGSPN--GGGTQ----G-GACGY-QNTVSL-GYGYMTAALSTTLFQGGAACGAC

FE-L--KCKFIKETNSA---KN-W--CYS-YSK-SIVITATNLC---PPG-----S--TGGWCNPPRHHFDLPMPAFNTL

AK-------QVG-GVAPVLF-RRVPCQRKG-GIRFTV----GGNPW-FF---MILIHNVGGAGDITKVMIK-CPY--TP-

WY--PMYRNWGSLWT-VRTQ---MRG-SLSFQITT--S-DGRTVTTNNAVGNGW--KFGQTWE-G-AQMTY

>CpEXPA21 [B]

W------------RND-----GHITFYGSPN--GGGTQ----A-GACGY-QNTYAL-GYGSMTAALSSSLFQGGAACGAC

FQ-L--QCI---RANRG---KN-W--CYS-YAR-TITVTATNLC---PPG-----S--TGGWCNPPRAHFDLPMPAFLTL

AQ-------RVG-GVAPVRF-RRVRCAKRG-GVRFTI----GGNPW-FM---MILIHNVGGAGDIRAVQIK-GPR--TG-

WV--PMYRNWGALWT-CRTK---ISG-ALTFRITT--S-DGRTLVT-GGPGNSW--RFGQTWE-G---RNY

>CpEXPA22 [X]

W-------------RD----DGHITYYGSPN--GHGTQ----G-GACGY-QNTFSL-GYDHLTAALSTPLFQGGAACGAC

YE-L--RCKFIKESKTA---KN-X--CWN-YSR-SITSTATNLC---PPG-----S--TGGWCDPPRNHFDLPMPAFLTL

AR-------QNG-GVAPVHY-RRVPCQKKG-GIQFTI----GGNPW-FY---MILIHNVAGAGDVVRVQIK-SPT--TG-

WT--PMYRNWGALWT-VRKQ---LKG-ALSFQITT--S-DGRVVVTHNAVGHGW--RFGQTWE-G---AQY

>MpEXPA1PRO

W-------------RN-----AHATFYGGSD--ASGTM----G-GACGY-GNLYSQ-GYGTDTTALSAALFDNGAKCGAC

FA-I--TCYR----------SR-W--CLP-GKP-ILQVTATNFC---PSNPNLPND--NGGWCNPPRRHFDLAQPAFTQI

AI-------YEG-GIVPVLF-KRVPCKRSG-PIRYTI----NGQRY-FN---LVLISNVGGVGDVSAVSVK-GAN--SNY

WE--PMRRNWGQNWQ-SDTI---LVGQSLSFQVTT--S-DGSTVTVLDAVASNW--EFGQTFE-G---GQF

>MpEXPA2PRO

W-------------AD-----AHATFYGGQD--ASGTM----G-GACGY-GNLYSV-GYGTSSTALSNALFNNGLSCGAC

FQ-I--VCRLAE--------SK-Y--CYP-GG--SVVVTATNAC---PPGS-------EGGWCDPPKAHFDLSYPAFQRL

AQ-------PVG-GVIPVRY-KRVSCSRNG-GIRFTI----RGNPY-FD---LVLVTNVGEFGAVQSLQIK-GER--TG-

WT--YMRQNWGANWQ-ANVN---VQGQALAFKVTL--D-NGRSLEFYNVAPKNW--GFGQTFE---ADYNF

>MpEXPA3PRO

W-------------TP-----SHATFYGEYN--GVETM----G-GACGY-GNLWWR-GYGLETAALSDTLLNNGLTCGAC

FQ-L--KCDLDHGGY-S---TR-W--CYK-NVA-PITVTATNHC---PANWARPTN--NGGWCNPPRTHFDLPLVMFNRM

AQ-------PVA-GIIPIEY-RRVPCEKSG-GLKFYL----SGNPW-FN---LVLVYNVAGAGAVTSMKMR-GSQ-
-TV-

WY--SMKRNWGQYWE-CPIK---FSNQRLSFQVTL--S-SGHVRVINNLTPTNW--YFGQTYE---AATNF

>MpEXPA4PRO

W-------------TP-----SHATFYGDDS--GAATM----G-GACGY-GNLWWR-
GYGLATAALSDTLLNNGLTCGAC

FE-I--KCNI-TGGE-S---GK-W--CIQ-SNP-SIKITATNQC---PPNFDRPTD--
NGGWCNPPRTHFDLSLNMFRRL

AQ-------TQA-GIIPISY-RRTRCIKTG-GIRFYL----GGNAH-FN---LVLVYNVGGWGDVMKMQMR-GTK-
-TG-

WI--TMKRNWGQNWE-SSIN---YTGQSLSFIVTL--G-NGQVRVIRNLTPTVW--YFKQTYE---ADTNF

>MpEXPA5PRO

W-------------TD-----SHVTFYGASD--GIETM----G-GACGY-GNLWWR-
GYGLETAALSDTLLNDGLTCGAC

YQ-I--KCNIASGGY-S---TK-W--CYP-SNP-TIQVTATNRC---PPNWDRPTN--
AGGWCNPPRTHFDLPLVMFERM

AQ-------AVA-GIIPVKY-RRVPCVKSG-GIKFFL----GGNPW-FN---LVLVYNVAGGGNVKNMQMR-
GDK--TV-

WY--TMKRNWGQFWE-CPIG---FRGQKLSFIVTL--S-NGQVRVINNLTTELW--YFGQTYE---ALTNF

>MpEXPA6PRO

W-------------ST-----AHATFYGGSD--ASGTN----N-GACGY-SNVFAL-
GYGTMTAALSAPLFNDGLSCGAC

FQ-I--QCSG----------DA-A--CYS-N---SITVTATNLC---PQGS-------
YGGWCDYPNAHFDLSQPAFSQL

AQ-------TVA-GHVTVNY-QRVNCNRQG-GVRFQI----DGHTY-FM---QTLIYNVGGSGDVVAVQVK-
GSK--TG-

WI--SMTRGWGQNWQ-TGTV---LDGQALSFSVKT--S-DGKTLYHNNVADSNW--KYGQTFE-G---AQF

>MpEXPA7PRO

F-------------GD-----ATATFYGGND--AAGTA----G-GSCGY-ENPFTR-
GYGVTTVALSNSLLSNNLNCGAC

FE-V--KCKYTASAY-----TKKW--CYN-NK--SVVVTATNLS---PPGL-------NG------EHHFDLTYPMFTKI

SN-------MIA-GRIPIQY-RRVPCKKQG-GIKFTL----NGNPY-FN---LVLVYNVGGAGNVYAMSMK-GSK-
-SD-

WY--PMARNWGQNWQ-AMVR---LTGQSLSFRVTL--G-NGKTQTFSNVAAADW--KFGQTYQ---
ASSNF

>MpEXPA8PRO

W-------------TK-----AHATFYGGDD--ARGTQ----G-GACGY-GNMYTR-
GYGVKTVALSYTVFNEGLTCGAC

FQ-I--KCDL---SM-K---PR-W--CYA-NGG-IITVTATNSC---PPNWARPTN--
NGGWCNPPRVHFDLSYPMFAQL

AQ-------KVA-GIIPIQY-KRVKCVRSG-GIRFTI----NGNPY-FN---IVLVHNVGGYGNVVTLYIK-GTN--
SP-

WF--KMKQNWGANWE-DHNK---LTNQKLSFKATL--G-TGETMIFKDITGTYW--SHGQTYE---ANNNY

>MpEXPA9PRO

W-------------SP-----AHATFYGGSD--AQGTQ----G-GACGY-GNLYRI-
GYGINTAALSTALFANGLSCGAC

FE-L--KCNS----------PR-W--CKS-GRN-PIRITATNLC---PQNHAHPNN--
AGGWCNMPLQHFDMAQPAYEQI

AI-------YRA-GIVPVMY-RRVQCIKFG-GIHFTM----NGNRW-EV---LVLLTNVGGAGNVHAVSVK-GPH-
-TN-

WL--YMRRNWGQNWQINDKQ---LAGATLSFRVTT--E-DGRVVTSWDVVPHYW--RFGQTFE-G---
LNF

>MpEXPA10PRO

W-------------NY-----AHATFYGDET--AQDTM----Q-GACGY-GNMYQQ-
GYGTATTALSSTLFDNGFACGRC

FE-I--RCV-----------AS-WA-CYL-GNP-STIVTATNFC---PANWLKPTN--
NGGWCNPPRQHFDMSKPAFEKI

AA-------WRA-GIVPVAY-RRVPCSKQG-SMEFRI----EGNPW-WH---IVFITNVGGPGDIGEVYIR-GSG-
-TKR

WV--RMSHNWGVGFQ-VFGG---YLGQGLSFMVIS--SSNLETVMAWNVVPANW--RLGQTFS-G---
GQF

>MpEXPA11PRO

W-------------MS-----AKATFYGGQD--ASGTM----N-GGCGY-ANPFTL-
GYGAQTAALSNALFNKGLTCGAC

FE-I--KCAITATAY-A---KQ-W--CYA-NSP-SIYITATNLC---PQG-----S--
YGGWCNGANLHFDLAYAAFTKL

AR-------ENA-GVIPVNY-RRTPCKKSG-GIKFQL----NGNTY-WN---LVLIYNVGGGGNVAAAAIK-GSR--SN-

FY--TMNQNWGMNWE-SYSN---LVGQALTFKITL--G-NGRSATFYNVAPAGW--RFGQTYQ---AAYNF

>MpEXPA12PRO

M-------------D-----AKATYYGGND--ASGTM----N-GGCGY-ANPFAL-GYGDQTVALSTALWNKGLTCGAC

FE-I--KCKISATAY-A---KK-W--CYP-KAG-SIKITATNLC---PPG-----S--HGGWCDPPKSHFDLAYAAFTRL

AN-------FQA-GVIPIQY-RRTPCRKNG-GVKFSM----NGNPY-WH---LVLVHNVAGGGNVHAMQIK-GSR--TG-

FM--QMKQNWGQNWE-AYCN---LVGQSVTFKVTL--G-NGRTVTFWNAVASNW--KFGQTYQ---TKYNF

>MpEXPA13PRO

W-------------AE-----AHATFYGGQD--AGGTM----G-GACGY-GNLYSL-GYGTATTALSNALFHNGLSCGAC

FL-I--ECILQG--------SK-W--CYP-GK--SIVVTATNAC---PPGS-------EGGWCDPPRPHFDLSFPMFQML

AQ-------PVA-GVIPVRY-RRVSCSKQG-GVRFTL----HGNPY-FN---YILVHNVGGSGDVAAVALR-GEN--SR-

WN--RMNQNWGQYWS-CGGW---YLGQALSFKVTL--G-SGRSLEFWNVVDKNW--GFGQTYE---SDYNF

>MpEXPA14PRO

W-------------AD-----AHATFYGGQD--ASGTM----G-GACGY-GNLYST-AYGTASTALSNALFNNGLSCGAC

FQ-I--MCKL----D-G---SK-Y--CYT-GGK-SITVTATNAC---PPG-----S--EGGWCDPPKAHFDLSYPMFQQL

AE-------PVG-GVIPVQY-KRVSCQKNG-GIRFTI----NGNAW-FD---LVLVTNVGGGGDIAKLEMK-GAS--TD-

WC--PMSQNWGQNWQASGHP--ELCGQTLSFRATL--G-DGRSQEFYDVAPSNW--GFGQTFE---ANTNF

>MpEXPA15PRO

W-------------AD-----AHATFYGGSD--ASGTM----G-GACGY-GNLYSL-GYGTSSTALSNALFNNGLSCGAC

FL-I--QCKLEG--------SK-Y--CYP-GQ--SITVTATNAC---PPGS-------
EGGWCDPPRSHFDLSFPMFQKL

AQ-------PVG-GVIPVRY-KRVSCTKQG-GVRFTL----HGNPY-FN---YVLVHNVAGWGDVSSLAIK-
GDQ--TG-

WY--YMSQNWGQYWS-ASAK---LTGQALSFKVTL--G-NGKSLEFYNVADKNW--GFGQTYE---SDYNF

>MpEXPA16PRO

W-------------GD-----AHATFYGGQD--ASGTM----G-GACGY-GNLYSV-
GYGVSTAALSYAMFNKGLTCGAC

YE-L--VCKLEG--------SK-Y--CYP-GK--SITITATNSC---PPGS-------
EGGWCDPPKLHFDLSYPMFQKL

AQ-------PVG-GVIPIKY-KRVACAKSG-GVRFTI----HGNPY-FN---YILVHNVAGAGDVSALAIK-GEK--
TG-

WY--YMSQNWGQFWS-ASAN---VVGQALSFKITL--G-NGVSTEFSNVAPKDW--KFGQTFE---ADNNF

>MpEXPA17PRO

W------------EVTEFTRGARATYYGGND--ASGTL----G-GACGY-GNLFEN-
GYGVETTALSTALFNDGLTCGGC

FQ-I--QCIA----S-E---SD-W--CLA-NGTVSITVTATNFC---PPG-----S--
FGGWCDPPQPHFDLSYPMFATL

AQ-------PVG-GVIPVQY-RRVSCLKQG-GVRFQI----NGNNW-FF---LVLVMNVAGAGDVQAVAIK-
GTR--SE-

WI--SMQRNWGQNWASDGPP--ELVGQALSFRVTL--GLTKETLDFIDVAPADW--QFGQTFE-
DDSGLNF

>MpEXPA18PRO

C-------------IP-----ATATFYGGND--ASGTN----T-GACAY-PNVLAM-
GYGTMNAALSSAIFDHGKTCGAC

FQ-L--VCTYVPYGNLR---------CLR-G---RITVTATNYC---PQGS-------
KGGWCDAPRSHFDLAEPAFVQI

AP-------YNA-GVVHVDY-QRVNCQRSG-EIRYRL----YGFTY-FL---QVLVYNVAGTGDVTAMSIK-GSS--
EGR

WI--SMTRSWGQLWT-TGVV---LEHQALSFMVTI--S-DGRTITVNDVANEYW--RYGQTYE-G---GQF

>MpEXPA19PRO

W------------TD-----SHATFYGGNN--AQGTM----G-GACGY-GNMYSR-
GYGLETTALSSTLFNKGLTCGAC

FE-I--KCKV-----AD---TR-W--CYA-NAG-SIKVTATNLC---PANPARPTN--
NGGWCNPPRTHFDLSYPMFTRL

AK-------AVG-GIIPVQF-RRVPCVKVG-GIRFVM----NGNPW-FN---LVLVYNVAGAGNVVNMQMK-
GSK--TN-

WF--TMKQNWGQNWE-LKQK---LKGQSISFRVTL--G-NGKVMIANNVAPANW--NFQQTFE---
SDNNF

>MpEXPA20PRO

W-------------MD-----AHATFHGGSA--VADTM----G-GACGY-GNLISR-
GYGLHTSALSSSLFNSGLTCGAC

FK-V--RCKA----A-S---SM-R--CHA-TAG-SITVTATDLY---SENLATLIE--
DERRSNPSQPQFDLAVSAFQML

AR-------PMV-GLIPIEY-QRVPCEKEG-GIRFTL----NGNSW-FN---LVLVHNVGGDGNVVAVQVK-
GSR--ASS

WS--MMNRRWGQNWE-LREN---LQGQILSFRITT--G-FGKTVLLPDVAQADW--RFGQTYE---SEGNF

>MpEXPA21PRO

W-------------TD-----SHATFYGGND--GSGTM----G-GACGY-GNMYSR-
GYGLKTAALSSTLFNNGLSCGAC

FE-L--KCKV-----AE---SR-W--CYA-NAG-SITVTATNLC---PANPARPTN--
NGGWCNPPRTHFDLAYAMFIIM

AK-------PVG-GIIPVQY-RRVSCVKTG-GVRFTL----NGNPW-FN---LVLVYNVAGDGNVVTIKMK-GSK-
-SNT

WY--PMKHNWGQNWE-LKQK---LAGQALSFQLTT--G-YGKTMTFTNCAAANW--QFGQTYE---
ADGNF

>MpEXPA22PRO

W-------------TA-----SDATFYGGNS--GAGTM----G-GACGY-GNMYNT-
GYGLNTAALSPVLFNNGLSCGAC

FE-L--KCNL-----TG---TK-W--CYP-SAG-SITVTATNLC---PANPARTTD----
GWCNPPRTHFDLSYTMFAIL

AQ-------PVA-GIIPIQY-RRVSCVKTG-GVRFVI----NGNPW-FN---LVLIYNVGGDGNVVTAMMK-GEN-
-TE-

WY--SMTRNWGQNWQ-LSQK---LHGQALSFQLTT--G-YGTTLVCSDVAPANW--QFGQTFE---
AYTNM

>MpEXPA23PRO

A------------IP-----ATATFYGGND--ASGTN----T-GSCGY-PNVLTI--
DGPMNTALSGALYNGGEACGAC

FK-I--QCVSIPFGNLQ---------CLP-G---SIVVTATNLC---PQGS-------
TGGWCDEPRSHFDLAEPAFQHL

AP-------PVA-GVVNVEY-ERVSCLRSG-GIRFLI----QGHPY-FM---QVLVYNVGGMGDVTAVSVK-
GSS--SD-

WI--QMDRNWGQLWT-TGTV---LDGQALSFSVTT--S-DGRTVASNGVADSDW--QYGQTFE-G---NQF

>MpEXPA24PRO

A------------IP-----ATATFYGGND--ASGTN----TAGSCGY-PNVLTI--
DGPMNTALSGALYNGGEACGAC

FK-I--QCVSIPFGNLQ---------CLP-G---SIVVTATNLC---PQGS-------
TGGWCDEPRSHFDLAEPAFQHL

AP-------PVA-GVVNVEY-ERVSCLRSG-GIRFLI----QGHPY-FM---QVLVYNVGGMGDVTAVSVK-
GSS--SD-

WI--QMDRNWGQLWT-TGTV---LDGQALSFSVTT--S-DGRTVASNGVADSDW--QYGQTFE-G---NQF

>MpEXPA25PRO

W------------TN-----ATATFYGGDD--GAGTM----G-GACGY-GNMFTR-
GYGLETAALSTVLFNNGLTCGAC

FM-L--KCRL----N-E---SR-W--CYS-NVS-SITISATNFC---PPNLNRTTD----
GWCNLPRSHFDLSVKMFTTL

AR-------AVG-GIIPVLY-KRVPCVKTG-GVRFTL----NGNPW-FN---LVLISNVGGQGNVVAAQMK-
GSK--TA-

WF--NMRQNWGQNWE-LGQK---LINQTLSFRLTL--G-LGQTLTFNNLTTPDW--KFGQTWE---ADTNF

>MpEXPA26PRO

F------------GD-----ATATFYGGSD--ASGTQ----G-GSCGY-QNPFAL-
GYGIKTAALSNSLLANNMNCGAC

FE-V--KCKYDASAY-T---KK-W--CYP-NSP-TIIVTATNQS---PPG-----L--NG------EHHFDLTEPMFMKI

AN-------RIG-GRIPIQY-RRVRCYKPG-GIKFTL----TGNPF-FN---LVLVYNVGGAGNVYGMAIK-GSR--
TD-

FY--PMSRNWGQYWQ-AFVK---LTGQSLTFRVTL--G-NGKSTTFWNVAPANW--QFGQTVA---ATYNF

>MpEXPA27PRO

F------------GD-----ATATFYGGTD--AAGTA----G-GSCGY-ENPFTR-
GYGVTTAALSNSLLSDGLNCGAC

FE-V--KCNFGASDY-----TRKY--CYN-GK--SVVITSTNLS---PPGL-------HG------EHHFDLTYPMFTQI

SN-------MIA-GRIPVQY-RRVPCKKQG-GIKFNI----NGNPY-FN---LVLVYNVAGGGNVYAMAMK-GGN--SA-

WC--TMGRNWGQNWQ-SSVK---LTGQGLSFRVTL--G-SGKMLVFNNVAPSSW--QFGQTYQ---ASSNF

>MpEXPA28PRO

F------------GD-----ATATFYGGSD--AAGTA----G-GFCGY-ENPFTR-GYGVTTVALSDSLLSNGLNCGAC

YE-V--KCKWEASDY-----TKKY--CYQ-GM--TTIVSATNLS---PPGL-------NG------EHHFDLTYPMFVKI

SN-------PES-GRIPIQY-RRVPCQKQG-GIRFSM----VGNPF-WN---LVLVYNVGGWGNVHSLYVR-GSR--TD-

WY--YMNRNWGQNWQ-CSVN---LTGQKLSFRVVL--G-NGQTSVFNNVADSNW--KHGQTYE---ASSNF

>MpEXPA29PRO

W------------TD-----SHATFYGGNN--AQGTM----G-GACGY-GNMYSR-GYGLETTALSSTLFNKGLTCGAC

FE-I--KCKV-----AD---TR-W--CFP-KAG-SIKVTATNLC---PANPARPTN--NGGWCNPPRTHFDLSYPMFTRL

AK-------AVG-GIIPVQF-RRVPCVKVG-GIRFVM----NGNPW-FN---LVLVYNVAGGGNVVNMQMK-GSK--TN-

WF--TMRQNWGQNWE-LKQK---LKGQSISFRMTL--G-NGKVMIANNVAPANW--NFQQTFE---SDSNF

>MpEXPA30PRO

W------------TD-----SHATFNDGNN--AQGTM----G-GACGY-GNISSR-GYGLETTALSSTLFNSGLTCGAC

FE-I--KCKV-----EN---ST-W--CFP-NAG-SIKVTATDLC---PANPAMPTN--NGGWCNPPRTHFDLSYQMFTRL

AK-------AEG-AIIPVQF-RRVPCVKVG-GIRFTM----NGNPW-FN---LVLVSNVAGAGNVVNLQMK-SSE--TN-

WFWMTARQISGQYWE-LNQN---LMGQNISFRVTL--G-NGKVMIANNVVPANW--NFQQTFE---SDINF

>MpEXPA31PRO

W------------TD-----SHATFYGGNN--AQGTM----G-GACGY-GNMYSR-GYGLETTALSSTLFNKGLTCGAC

FE-I--KCKL-----AD---SR-W--CFP-KAG-SIKVTATNLC---PANPARPTN--NGGWCNPPRTHFDLSYPMFTRL

AK-------AVG-GIIPVQF-RRVPCVKVG-GIRFVM----NGNPW-FN---LVLVYNVAGAGNVVNMQMK-GSK--TN-

WF--TMRQNWGQNWE-LKQK---LKGQSISFRVTL--G-NGKVIIANNVAPANX--NFQQTFE---SDSNF

>MpEXPA32PRO

W------------TD-----AHATFYGGQD--ASGTM----G-GACGY-GNLYSR-GYGTASTALSNALFNSGLSCGAC

FE-I--ICKL----S-E---TK-W--CYT-GGK-SIVVTATNQC---PQG-----S--EGGWCDYPRSHFDLSFPMFQSL

AQ-------AVG-GVIPVQY-RRVSCSRSG-GIRFTI----NGNAY-FN---LVLVTNVGGKGDVAALWMR-GSN--TD-

WQ--PMKQNWGQNWQ-CDTK---LVGQALSFKAML--G-SGETKEFYNVADSNW--LFDQTYE---GDYNF

>MpEXPA33PRO

G------------LK-----AHATFLGGSD--AVGTN----N-GACGY-SNVLAM-GYGTRTAALSAPIFREGKSCGAC

YK-L--RCRG----------SE-SVGCRS-ND--GVVVTVTDVC---PERS-------VGGWCD-GQPHFRLSEPAFAQI

AE-------PKSGGLVEVDY-EKVSCDRRG-GMHFLL----VGHTH-FL---QILVHNVGGCGDVAAVSVK-GGT--GP-

WI--RMSRSWGQLWH-TGEV---LDGQALSISVTM--A-DGKVCVSNNFAGADW--KYGQTFE-G---AQL

>MpEXPA34PRO

A------------LP-----AHATFYGGSD--ASGTN----N-GACGY-SNVLST-PFGTMTAALSSPLFNEGLSCGSC

YK-V--RCSG----------SS-TGGCID-N---EIMVTATNLC---PHGS-------EGGWCD-GKPHFDMSQPAFSQI

AK-------PVA-GVVEVEY-TKVPCPRKG-GMQFTV----MGHTY-FL---QVLIFNVGGSGDVTAVKVR-GGN--GE-

WI--AMTRSWGQLWQ-TGKV---LDGQALSFQVTT--S-NGKTCVSNYVAGSHW--KYGQTFE-G---SQY

>MpEXPA35PRO

W-------------TD-----SHATWYGGKD--GAGTM----G-GACGY-GNMNAR-
GYGMKTAALSTKLFNDGFTCGAC

FE-I--RCKS----S-Q---SK-Y--CYS-STA-SVIVTATNYC---PANPNRPTD----
GWCNGDRTHFDLTYQMFTTL

AI-------YEA-GIIPVQY-RRVPCAKKG-GIRFTI----NGNPN-FN---LILLSNVGGSGNVVSVQMK-
GENSYSG-

WV--PMKQNWGQMWE-CGTK---VTGQSISFKVTV--G-YGQTFTFWTVAPKTW--QFGQTYE---ASSNV

>MpEXPA36PRO

W-------------GD-----AHATFYGGND--ASGTM----G-GACGY-GNLYSL-
GYGTASTALSNGLFNNGLSCGAC

YT-I--ECKLEG--------SK-W--CYP-GK--SITVTATNAC---PPGS-------
EGGWCDPPRLHFDLSYPMFQTL

AQ-------PVG-GVIPVKY-KRVSCNRQG-GIRFTL----HGNAW-FN---YVLVHNVAGDGDVAGVSIK-
GDN--GG-

WT--TMYQNWGQFWS-ASEH---LVGQALSFRVTL--G-SGRTQEFYDVASPNW--YFGQTYE-
ADNNQNF

>MpEXPA37PRO

W-------------GN-----AHATFYGGND--ASGTM----G-GACGY-GNLYSR-
GYGTATTALSNGLFNNGLTCGAC

FT-L--VCKLDG--------SR-W--CYSGGK--QITVTATNAC---PPGS-------
EGGWCDPPRLHFDLSYPMFQKL

AQ-------PVG-GVIPVRY-KRVPCVKQG-GIRMSL----YGNPW-FD---YVLVHNVAGGGDVAAVSIK-
GSK--TN-

WV--VMHQNWGQYWS-ASSY---LVGQALSFKVTL--G-NGKSIVFWDIAPKNW--QFGQTYE-
ADNNQNF

>MpEXPA38PRO

W-------------KA-----AHATFYGGSN--AQGTM----G-GACGY-GNLYNT-
GYGLETAALSSTLFNNGKTCGAC

YE-M--FCNY---RQ-Q---SR-W--CYK-AQP-HIKVTATNLC---PANWNRPTN--
RGGWCNPPRVHFDLPQPMFIKM

AR-------YVG-GIVPVAY-RRVPCVKKG-GVKFTM----NGNPW-FN---LILISNVAGPGDIKAVAVK-GSK--
QKS

WT--SARQNWGQKWE-VKQK---LQGQALSFRLTS--G-SGKVLVLYNAVPRTW--RFGSTYM---
ARSNW

>PpEXPA1PRO

W-------------RK-----AHATFYGGAD--ASGTM----G-GACGY-GNLYST-
GYGVDSTALSTALFNNGAKCGAC

FA-I--QCYR----------SQ-Y--CVP-GSP-VITVTATNFC---PPNHKGDG---
TPGWCNPPMRHFDLAQPSFTKI

AK-------YRA-GIVPVLF-RRVPCEKKG-GVRFTI----NGNKY-FN---LVLVHNVGGKGDVHAVDIK-GSN--
TE-

WI--PMKRNWGMNWQ-TDAV---MTGQALSFRVTT--S-DGKTIVSMNATPSHW--SFGQTFE-G---GQF

>PpEXPA2PRO

W-------------KE-----AHITYYGTAN--GGGTQ----G-GACGY-PNTFAM-
GYGVMTAALSYPLFQGGKSCGAC

YQ-L--KCKWLAPTRTV---HN-W--CWS-YSR-TITITATNSC---PPG-----S--HGGWCDW-
KPHFDLPMPAFMTL

AR-------REG-GVAPVYY-RKVRCAKRG-GIRFTL----GGNPY-FM---MILIHNVGGAGDLRAVKIK-GRN-
--GY

WV--PMWQNWGALWT-CKTK---LSG-ALSFQITT--G-DGRTVTVNRAVGDYW--KFGQTWE-G---SQF

>PpEXPA3PRO

W------------ISG----VAHATFYGGVD--AQGTQ----G-GACGY-GNLYST-
GYGTSTTALSSALFNAGLSCGAC

FE-L--KCDS----A-N---SK-Y--CLP-GDK-SITVTATNYC---PQG-----S--
DGGWCDSPKQHFDLSHPMFTSL

AQ-------EVG-GVIPVTY-RRAPCAKKG-GMRFTI----NGNPW-FV---MILVTNCGGAGDVQQLQIR-
GSD--TP-

WY--PCVRNWGQMWQMTSDP--NLPGKALSFRATL--S-DGSVAESLNAAPSNW--GWGQTFE---
GVATY

>PpEXPA4PRO

W-------------KD-----AHITYYGSPN--GGGTQ----G-GACGY-QNTYAL-
GYGSFTAALSAPLFQGGAACGGC

YQ-L--KCAPVRETRTV---HN-W--CWS-YSR-SIVVTATNLC---PPG-----S--HGGWCAW-
RPHFDLPMPAFTSL

AK-------QVG-GVAPVFY-RRVRCAKRG-GVRFTI----GGNPY-FL---MVLIHNVGGAGDIRSVRIK-GQY--
SG-

WV--TMFRNWGSLWT-CRTK---LSG-PLSFMITT--S-DGRTLVSNRAVGSWW--KFGQTWE-G---SQF

>PpEXPA5PRO

W-------------GR-----AHATFYGGAD--ASGTQ----G-GACGY-GNLYST-
GYGTNTAALSSSLFNSGLSCGAC

YE-L--TCDP----S-C---SQ-Y--CLP-GGS-AI-ITATNFC---PTG-----S--
NGGWCNPPKQHFDLAQPVFSKI

AR-------TVG-GVIPINY-RRVPCSKSG-GMRFTV----NGNPY-FL---LVLVTNVGGAGDVQQLYIK-GAS--
TG-

WL--PLKRNWGQMWQFTGNS--GMHGQAISFKAVT--S-DGAEAISPNVAPANW--GFGQTFE-G---
SNF

>PpEXPA6PRO

W-------------RN-----ARSTFYGGMD--AAGTM----S-GACGY-GNLYAS-
GYGVHTTALSSALFKNGMACGAC

FE-V--QCGG------K---GK-P--CKP-G---SVVVTATNFC---PPNPGQSAN--
NGGWCNPPNEHFDLSYPAFVKI

AD-------PKA-GAVPLQY-RRVPCQKQG-GIRFTI----NGNCN-FI---LVTITNVGGSGVVTAAYLK-GDK--
TE-

WS--PLSRNWGANWQ-CRRN---YCGQGISIKIVT----SDNKVSVTKLAKSDW--CFGKTFI-G---KQV

>PpEXPA7PRO

W-------------GY-----AHATYYXGAD--ASGTQ----GGGACGF-GNLYST-
GYGTNTAALSAALFNSGLSCGSC

YE-L--ACDP----N-G---SK-Y--CLP-GGP-TVTVTATNFC---PHG-----S--
LGGWCDAPKQHFDLAHPMFVSL

AR-------EVG-GVIPIKY-RRVPCVKSG-GMRFTI----NGNPW-FL---LVLVTNVAGAGDVQHMYIK-GSN-
-TP-

WE--PMSRNWGSMWQFTGDS--KMKGQALSFKAVT--S-DGSVAVSMDAAPGNW--QFGQTFE-G---
VNF

>PpEXPA8PRO

W-------------NE-----AHATFYGGSD--AGGTTGTR-G-GACGY-GDLYST-
GYGTSTVAISSALFDRGLACGAC

YQ-V--KCAG------S---SS-E--CRS-DSP-AIQVTVTNFC---PPNPSLPED--
NGGWCNLPLHHFDMSMPAFEQI

AT-------YKA-GIVPVMY-RRTSCVRTG-GIHFTM----SGHNF-MN---LVLVTNVGGMGDVQSVSIR-GSK-
-TS-

WV--TMTRNFGQIWQ-STVN---MSGQSLSFMVTT--S-DGKTVVSNNVAPPDW--AFGQTYE-G---SQF

>PpEXPA9PRO

W-------------TD-----AHATFYGGAD--ASGTQGGCAG-GACGF-GNLYST-
GYGTNTAALSQALFNSGLTCGAC

FE-L--VCDSSG--------SR-Y--CVT-SS--SVVVTATNFC---PTGS-------
TGGWCDYPRQHFDLSQPVFTRI

AQ-------PVG-GVVTLKY-RRVRCQKSG-GIRFTI----TGNPY-FI---LVLVTNVGGAGDVQQLYIK-GSS--
TG-

WN--AMSRNWGQLWEIRNAA---LMGQALSFRVVT--S-DRAEVASFDAVPANW--AFSQTFE-G---SNF

>PpEXPA10PRO

W------------VSG----RAHATFYGGAD--ASGTQ----G-GACGY-GNLYST-
GYGTSTAALSSALFNSGLSCGAC

YE-L--TCDT----S-G---SK-Y--CLP-GNP-SIILTATNYC---PQN-----S--
NGGWCDAPKQHFDLAHPMFVSL

AE-------ERG-GVIPVNY-RRVPCAKKG-GMRFQM----NGNPW-FL---LVLVTNVGGAGDVQQLSIK-
GSN--SG-

WY--QMKRNWGQMWQLTGNS--NMPGQALSFRAVL--S-DGTTVESLDAAPANW--HFGQMFE-G---
SQA

>PpEXPA11PRO

W-------------GR-----AHATYYGGAD--ASGTQ----G-GACGF-GNLYSS-
GYGTDTAALSSALFNSGLSCGAC

YE-L--TCDP----S-G---SK-F--CIP-GGS-AI-ITVTNFC---PTG-----S--
NGGWCNPPKQHFDLAQPVFRKI

AR-------TVG-GVVPINY-RRVSCLKDG-GMRFTV----NGNPY-FL---LVLVTNVGGAGDVQQLYMK-
GSS--TN-

WQ--PLKRNWGQMWQFTGNS--RMHGQAISFKAVT--S-DGSVAVSNNVAPPNW--GFGQT---------F

>PpEXPA12PRO

W-------------ND-----AHATYYGGAD--AS-------G-GACGF-GNLYST-
GYGTSTAALSQALFNSGLTCGAC

FE-L--ACDP------SG--SK-Y--CYK-GS--SIVVTATNFC---PSGS-------
EGGWCDSPKQHFDLSQPVFNKI

AQ-------QAG-GVIPVKY-RRVPCRKSG-GMRFTI----NGNPY-FI---LVLVTNVGGAGDVQQLSLK-GSS-
-TG-

WY--TMSRNWGQQWEFRGNS--ALVGQALSFRVVT--S-DGAEAVSYDAASENW--SFSQTFE-G---INF

>PpEXPA13PRO

W-------------NE-----ARATFYGGSD--AGGTT----G-GACGY-GDLYST-
GYGTNTVATSSAIFDRGLACGAC

YQ-V--KCAG--SA-------S-E--CQP-GTP-AIQVTVTNFC---PPNPSLPEG--
NGGWCNLPLHHFDMAMPAFQQI

AS-------YRV-GIVPILY-RRASCVRTG-GIRFTM----SGHKF-MN---LVLVTNVGGMGDVQTVFIQ-GSK--
TK-

LV--AMIRNFGQIWQ-SSVN---VSGQRLSFMVMT--S-DGESVVSRNVAPSDW--AYGQTYE-G---SQF

>PpEXPA14PRO

W-------------DK-----GHATYYGEGD--ARGTM----G-GACGY-SNLYST-
GYGVNTAALSGPLFNGGATCGAC

YE-L--TCILNE--------SK-W--CYR-GK--NIIVTATNFC---PSGS-------
TGGWCNPPQKHFDLSEPMFTTL

AN-------RVG-GVIPVNF-RRVACYKQG-GMRFTI----NGNPY-FF---IVLVYNVAGAGDVQQVYIK-GPK-
-TQ-

WL--QMYRNWGSQWTFNGGP-NNIVGSALSFRVHT--S-DGRQVISYXAAPANW--WFGXTFS---
SGAN-

>PpEXPA15PRO

W-------------GY-----AHATYYGGAD--ASGTQ----GGGACGF-GNLYST-
GYGTNTAALSAALFNSGLSCGSC

YE-L--ACDP----N-G---SK-Y--CLP-GGR-TVTVTATNFC---PHG-----S--
LGGWCDSPKQHFDLAHPMFVTL

AK-------EVG-GVIPIKF-RRVPCVKSG-GMHFTI----NGNPW-FL---LVLVTNVAGAGDLQQVYIK-GSN--
TP-

WE--PMSRNWGSMWQFTGNS--KMKGQALSFKTIT--S-DGAVAISYDAAPNNW--QFGQTFE-G---
VNF

>PpEXPA16PRO

W-------------DT-----AHATYYGGRD--AGGTM----G-GACGY-GNLYNT-
GYGVKTAALSAPLFKGGATCGAC

YE-L--TCILSQ--------SK-Y--CYQ-NK--KILITATNFC---PTGS-------TGGWCNPPRKHFDLSEPMFTTL

AN-------RVG-GVIPVNF-RRVHCYKKG-GMRFTI----NGNPY-HM---MVLVYNVAGAGDVQQMFIK-
SPT--TG-

WL--QMSRNWGQIWTYKGGP-RNIVGFALSFRVYT--S-DRRQVVSYNAAPANW--RFGQTFS---SGAN-

>PpEXPA17PRO

W-------------GR-----AHATYYGGAD--ASGTQ----G-GACGF-GNLYSS-GYGTSTAALSSSLFNSGLSCGAC

YE-L--TCDP----S-G---SQ-Y--CLP-GGS-AI-ITVTNFC---PTG-----S--NGGWCNPPRQHFDLAQPVFSKI

AR-------TVG-GVIPINY-RRVSCLKSG-GMRFTV----NGNPY-FL---LVLVTNVGGAGDVQQLYIK-GSS--TA-

WL--PLKRNWGQMWQFTGNS--GMHGQAISFKAVT--S-DGAEAISNNVASSNW--GFGQTFE-G---SNF

>PpEXPA18PRO

W-------------NE-----AHITYYGTAN--GGGTQ----G-GACGY-PNTFAM-GYGAMTAALSYPLFQGGKACGGC

YQ-L--RCKWVTPTRTV---HN-W--CWS-YSR-TITVTATNSC---PPG-----S--HGGWCNW-RPHFDLPMPAFLTL

AR-------REG-GVAPVYY-RKVRXAKRG-GIRFTI----GGNPY-FL---MILIHNVGGAGDLKAVKVR-GGN---GY

WV--PMWRNWGALWT-CKTR---MSG-ALSFQITT--G-DGRTLTTYKAVGGYW--RFGQTWE-G---SQF

>PpEXPA19PRO

W-------------GD-----AHITYYGSPN--GAGTE----G-GACGY-QNTYKL-GYGSMTAALSSRLFQGGKACGGC

YQ-L--RCAP----NRG---RN-W--CWS-YAR-AIVVTATNLC---PQGS-------HGGWCDYPKSHFDLPMPAFTSL

AR-------REG-GVAPVWY-RKVRCAKRG-GVRFTI----GGNPW-FL---MVLIHNVGGAGDVVSVKVK-CPY--TG-

WV--SAYRNWGCLWT-VRTK---MTG-PLSFTLVT--S-DGRTLYSMNAVRNGW--KFGQTWE-G---SQF

>PpEXPA21PRO

Y-----------GXRD-----AHITYYGSPN--GGGTE----G-GACGY-QNTYSL-GYGFMTAALSSSLFQGGSSCGAC

YQ-I--RCEPIRVTRTV---KN-W--CWS-YSR-TITVTAINLC---PPR-----S--SGGCCNPPLQHFDLPMPAFLSL

AR-------REG-GVAPVXY-TKVRSEKRG-GIRFTM----GGNLW-FL---TILIHNVGGAGDVRSVRIK-SPH--SG-

LI--SMYRNWGSLWT-VRAR---MSG-ALFFMITT--S-HGRVLITRNAVGSGW--RFGQTWE-----GNF

>PpEXPA22PRO

W-------------QD-----AHITYYGSPN--GGGTQ----G-GACGY-QNTVSL-GYGFMTAALSSPLFQGGKACGAC

FQ-L--QCARVQETRTV---KN-W--CHD-YSK-AITVTATNLC---PPG-----S--EGTWCDPPRHHFDLPMPAFLSL

AR-------QEG-GVAPVYY-RRVQCLKKG-GIRFTM----GGNPW-FL---MVLVHNVXGAGDVVXVKVK-CPS--SG-

WY--DMYRNWGALWT-VQKK---MVG-PLSFLLTT--G-DGRRLTAYNAVGNGW--TFGQTWE-G---AQY

>PpEXPA23PRO

W-------------IN-----GRITYYGSPN--GGGTQ----G-GACGY-QNTVSL-GYGFMTAALSTTLFKGGAACGAC

YQ-L--QCAPVSETPSGLLKRN-W--CWQ-VGR-SILVTATNLC---PPGS-------SGGWCNPPQHHFDLPMPAFLAL

AR-------REG-GVVPVYY-RKVNCARKG-GIRFTV----GGNPW-FL---MILIHNVGGAGDVVAVKVK-CPT--SG-

WY--DMYRNWGALWT-VQKK---MNG-PLSFAITT--S-DGRTVTTYNAVGNGW--KFGQTWE-G---AQY

>PpEXPA24PRO

W-------------SS-----GRITYYGSPN--GGGTQ----G-GACGY-QNTVSL-GYGFMTAALSTPLFNGGAACGAC

YQ-L--QCAPVHETPKNLLQRN-W--CWK-VGR-RITITATNLC---PPGS-------EGGWCDPPQHHFDLPMPAFTAL

AK-------REG-GVVPIYY-RRVRCAKQG-GIRFTM----GGNPW-FL---MILIHNVAGAGDVVAVKIK-CPT--SD-

WC--DMYRNWGAFWT-VQKK---MEG-PLSFQITT--X-DRRKVTTHNAVGHGW--QFGQTWE-G---AQY

>PpEXPA25PRO

W-------------RD-----AHITYYGSPN--GGGTQ----G-GACAY-QNTFSL-GYGAMTAALSSPLFEGGAACGAC

YQ-L--QCKRVQETRTV---KN-W--CWS-YSR-TITITATNLC---PPG-----S--AGAWCDPPRHHFDLTMPAFLTL

AR-------REG-GVAPVLY-RRVKCVKRG-GIRFTI----GGNPW-FL---MILIHNVAGAGDVRAVRIK-TPS--TD-

WI--PMYRNWGALWT-VQRK---LSG-PLSFQITA--G-DRRQITINSAVGNAW--KFGQTWE-G---HNF

>PpEXPA26PRO

W-------------NF-----GRITYYGSPN--GGGTQ----G-GACGY-QNTFAL-GYGTNTAALSSRLFQGGAACGAC

YQ-L--RCI---APKWG---KN-W--CWN-YAR-SIVVTATNLC---PSG-----S--NGGWC-PSQAHFDXPMPAFTSL

AR-------KEG-GVTPIMY-RRVRCARRG-GIRFTI----GGNPF-FL---MVLIHNVGGAGDVRAVSIK-GQY--TG-

WV--GMYRNWGSLWT-CTTK---IDG-ALTFRITT--G-DGKTLILYNAVRKGW--RFGQTWE-G---SQF

>PpEXPA27PRO

W-------------AT-----ATATFYGGAD--AG-------G-GACGY-GNLYST-GYGASTTALSAPLXNGGSACGAC

YQ-L--QCAR----------SN-H--CYA-GR--SITVTATNFC---PTGS-------EGGWCNPPRKHFDLSMPMFTTL

AR-------QVA-GVVPVDY-RRVACNKKG-GQRFLM----TGNPY-FI---MVLVYNVAGAGDVQRFFVK-GSM--TG-

WY--ELRRNWGQIWTCTADS--RLKGQALSFRTQT--S-DGRQVVSIDAAPANW--NFGQTFS---SGVN-

>SmEXPA1PRO

W-------------TS-----AHATFYGGSD--AAGTM--V-G-GACGY-GNLYSQ-GYGNNNAALSTALFNSGLSCGAC

FE-I--RCDS----AAD---PR-W--CIA-GT--SVVVTATNFC---PPNYALANN--NGGWCNPPLEHFDMAQPAWEQI

GI-------YRG-GIVPVQY-RRVSCVKKG-GIHFTM----NGHTY-FN---LVLISNVGGXGDVHAVSIK-GSG--TG-

WQ--DMSRNWGQNWQ-SNXQ---FQGQSLSFKVTT--S-DGKSVVSMDVAPADW--QYGQTFE-G---SQF

>SmEXPA2PRO

W-------------TG-----AHATFYGGSD--ASGTM----G-GACGY-GNLYSQ-GYGTNTAALSTALFQSGLSCGAC

FE-V--KCNG----------DPEW--CLP-GS--SVLVTATNFC---PPNXALPNN--NGGWCNTPLQHFDMAQPAFEQI

AK-------YRG-GIVPVLX-RRVPCQRKG-GIRFTM----XGHNY-FN---LVLVTNVGGAGDVHSVSIK-GSN--TD-

WL--PMSRNWGQNWQ-SNAI---LSGQSLSFKVTT--S-DGRTVVSYDAAPPNW--QYGQTYS-G---DQF

>SmEXPA3PRO

W-------------TD-----AHATFYGGSD--ASGTM----G-GACGY-GNLYSQ-GYGXNTAALSTXLFNSGLSCGAC

FE-I--KCNA--XK--D---PQ-W--CRA--GA-SVTVTATNFC---PPNYAQAND--NXGWCNPPLEHFDMAQPXWEQI

GI-------YRG-GIVPVQY-RRVSCVKKG-GIHFTL----NGNKY-FM---LVLVSNVGGAGDVRAVSIK-GPS--GD-

WQ--PLSRNWGQNWQ-SDSR---LIGQSLSFRVVT--S-DNRAVTSLNVAPAGW--SFGQTFS-G---EQF

>SmEXPA4PRO

W-------------TD-----AHATFYGGSN--AAGTM----G-GACGY-GNLVSA-GYGTNTAALSTALFQDGLSCGAC

FE-V--KCAS--GS--D---PK-W--CLP--G--SVVVTATNFC---PPS-SQPSN--DGGWCNSPLQHFDMAQPAFLKI

AQ-------YSA-GIVPISY-RRVSCSRSG-GIRFTM----NGHAY-FN---LVLITNVGGAGDVHAVSIK-GSG--TD-

WI--PMSRNWGQNWQ-SNAL---LGGQALSFKVTT--S-DGKTTIAYNVAGANW--AYGQTFE-G---EQF

>SmEXPA5PRO

W-------------KQ-----XFATFYGDET--ARETM----G-GACGY-GNLYQS-GYGLMTAALSSTLFNSGYGCGQC

YE-I--TCTL----------SK-H--CYF-GK--SVVVTATNLC---PPNWSKPSN--NGGWCNXPRVHFDMSKPAFMKI

AF-------WRA-GIIPVSY-RR-----SG-GMNFKL----XGNRW-WL---MVFITNVGGSXDIKAVSVK-GSR--TG-

WI--AMTRNWGVGFQ-VFKQ---LQGQSLSFMVTC--YSTXKXXVHNNVAPANW--QLGSTYS---AKQLX

>SmEXPA6PRO

W-------------RY-----AHATFYGEYD--ALETM----G-GACGY-GNLYSQ-GYGTDTTALSTVLFNSGYGCGGC

YE-I--SCTQ----------SK-H--CYP-G---STIVTATNLC---PPNWYKPSN--NGGWCNPPRIHFDMSKPAFSKI

AY-------WRX-GIVPVRY-RRVPCXRKG-XIKFEL----KGNRW-WL---IVFVSNVGGPGDIKRMAVK-GSK-
-TG-

WL--PMSRNWGVGFQ-VFKS---LHGQSLSFMVTS--FTTGKTVTAYDVVPANW--RIGQAYS---GGQMV

>SmEXPA7PRO

W-------------LD----GAHATYYGGSD--ASGTN----S-GACGY-GNQLSA-
GYGYITTALSTPLFENGDICGAC

FE-I--RCAG----------GA-G--CLP-GNP-STVVTATNLC---PXGS-------
NXGWCDPPKPHFDLSQPAFSRI

AS-------IXN-GHVQLQY-RRVACDRQX-GIRFTV----NGHTF-FN---LVXVENVGGSGDVVAVEVK-
GSA--TG-

WR--QMQRNWGQNWQ-DMGD---LNGQALSXRVTG--X-DGKVVTSMNVAPADW--QFXRTYS-G---
GQF

>SmEXPA8PRO

W-------------LD-----AHATYYGGSD--ASGTN----S-GACGY-GNQLSA-
GYGYITTALSTPLFXXGDICGAC

YE-I--RCAG----------TG----XLP-RNP-STVVTATNLC---PPGS-------
NGGWCDPPKQHFDLXQPAFSQI

AS-------IPY-GHVLLQY-RRVPCQRQG-AIHYTI----NGXTF-FN---LVLIENVGGSGDVVGVEIK-GSN--
TN-

WM--PMARNWGQNWM-IGGN---LGGQSLSFRVTG--S-DGRKVTSLNVAPANW--QFGRAYS-G---
GQF

>SmEXPA9PRO

W-------------QD-----AHATFYGGSD--ASGTM----G-GACGY-GNLYLQ-
GYXVSXAALXTPLFXEGWSCGSC

FE-L--KCNA--EA--D---PE-W--CLP-GNP-SIVVTATNFC---PPNFALPSD--
XGGWCNPPREHFDLSQPAFELI

AK-------YRX-GIVPVQY-RRVPCEXEG-GIHFSI----NGHAY-FM---LVLVWNVGGAGDVHAVAVM-
GSR--TRR

WQ--PLVRNWGQNWQ-SPXV---LLGQSLSFMVTT--S-NGDTVTDYDVAPQDW--KFGQTFV-G---
NKN

>SmEXPA10PRO

W-------------TL-----AHATYYGGSD--ASGTMGKX-S-GACGY-GNMYHE-
GFGVETTALSTVLFQNGASCGAC

YE-L--KCHQ--DP-------K-W--CRP-GNL-SITVTATNFC---PPNPARKSY--
RGGWCNXPQQHFDLSMPAFVHL

AN-------RTA-GIIPVIY-TRVECKRQG-GIRFTM----RGNKW-FI---LVMISNVGGAGDVRSVVVK-GSR--
SP-

WT--PATRAWGQNWHISNRS---MLEQGLSFVVST--S-DGESRIALDAVPQNW--KFGQTFT---TAAQF

>SmEXPA11PRO

W-------------ES-----GHATFYGGSD--AAGTM----G-GACGY-GNLYSQ-
GYGTNNAALSSALYNNGLSCGAC

FE-V--KCDA----AAD---PQ-W--CIX-GR--XVTVTATNFC---PP----------
GSWCNEPLKHFDMSQPAWEEI

GI-------YRG-GIIPVYF-RRVSCVRKG-GIXFTV----NGHAY-FN---LILITNVGGAGDVHAVSVK-GSG--
TG-

WI--PMSRNWGQNWQ-TNAQ---LGGQSLSFMVTD--S-SGKTVISNNAAPSNW--QYGQTFE-G---
EQF

>SmEXPA12PRO

W-------------SN-----AHATFYGGSD--ASGTM----G-GACGY-GNVLSA-
GYGVNTAALSTALFNGGATCGAC

FQ-M--QCVN----------SR-W--CRP-GK--SVTVTATNFC---PPNNALXSD--
NXGWCNTPREHFDLSQPVWEQM

AI-------YQG-GIVPVQY-RRVKCYKQG-GIIFTM----NXNPN-FN---LVLIKNVAGWGDLRAVSIK-GSN--
TG-

WL--PMKRNWGSNWE-YHGV---LVGQSLSFLLTP--S-MGGSLISYDVXPRNW--QFGQSYS-G---RQF

>SmEXPA13PRO

W-------------TD----GAHATYYGGSD--ASGT-------GACGY-GNQLSA-
GYGVLTTALSAPLFNDGHVCGAC

FE-V--KCSW----------GDSG--CLA-GNP-SIVVTATNLC---PQGS-------
NGGWCDSPKQHFDLAQPAFALI

AV-------TLN-GHVPIQY-RRVSCKRDG-GLRFTI----NGHVY-FN---LVLIENVGGTGDVSAVSIK-GSK--
TG-

WR--PMTRNWGQNWQ-DGGD---LTGQSXSFEVTT--S-DGNXITAYDVAPDYW--QFGQTFS-G---
GQF

>SmEXPA14PRO

W--------------G-----AHATYYGGSD--ASGTN----N-GXCGY-GNQLSA-GYGTITTALSTPLFRGGNVCGXC

YQ-V--RCWG----------DP-A--CLP-GNP-SVVVTATNLC---XPGS-------NGGWCDPPKPHFDLSQPAFSRI

AR-------IPN-GHAQIQY-RRVKCQRQG-GIRFTI----NGHTY-FN---LVLVTNVGGMGDVVGVSIK-GSS--SG-

WR--SMSRNWGQNWE-EGSN---LNGQALSFRVTT--S-DGRTVTAYNVAPGDW--QFGRTYT-GNTASQY

>AtEXPA7PRO

W-------------RY-----AHATFYGDET--GGETM----G-GACGY-GNLFNS-GYGLSTAALSTTLFNDGYGCGQC

FQ-I--TCSK----------SP-H--CYS-GK--STVVTATNLC---PPNWYQDSN--AGGWCNPPRTHFDMAKPAFMKL

AY-------WRA-GIIPVAY-RRVPCQRSG-GMRFQF----QGNSY-WL---LIFVMNVGGAGDIKSMAVK-GSR--TN-

WI--SMSHNWGASYQ-AFSS---LYGQSLSFRVTS--YTTGETIYAWNVAPANW--SGGKTYK---STANF

>AtEXPA8PRO

W-------------QG-----GHATFYGGED--ASGTM----G-GACGY-GNLYGQ-GYGTNTAALSTALFNNGLTCGAC

YE-M--KCND------D---PR-W--CLG-S---TITVTATNFC---PPNPGLSND--NGGWCNPPLQHFDLAEPAFLQI

AQ-------YRA-GIVPVSF-RRVPCMKKG-GIRFTI----NGHSY-FN---LVLISNVGGAGDVHAVSIK-GSK--TQS

WQ--AMSRNWGQNWQ-SNSY---MNDQSLSFQVTT--S-DGRTLVSNDVAPSNW--QFGQTYQ-G---GQF

>AtEXPA11PRO

L-------------TN-----GHATFYGGSD--ASGTM----G-GACGY-GDLYSA-GYGTMTAALSTALFNDGASCGEC

YR-I--TCDHAAD-------SR-W--CLK-GA--SVVITATNFC---PPNFALPNN--NGGWCNPPLKHFDMAQPAWEKI

GI-------YRG-GIVPVVF-QRVSCYKKG-GVRFRI----NGRDY-FE---LVNIQNVGGAGSIKSVSIK-GSK--TG-

WL--AMSRNWGANWQ-SNAY---LDGQALSFSITT--T-DGATRVFLNVVPSSW--SFGQIYS---SNVQF

>AtEXPA12PRO

W------------IR-----AHATYYGVND--SPASL----G-GACGY-DNPYHA-
GFGAHTAALSGELFRSGESCGGC

YQ-V--RCDF---PA-D---PK-W--CLR-GA--AVTVTATNFC---PTN-----N--
NNGWCNLPRHHFDMSSPAFFRI

AR-------RGNEGIVPVFY-RRVGCKRRG-GVRFTM----RGQGN-FN---MVMISNVGGGGSVRSVAVR-
GSK--GKT

WL--QMTRNWGANWQ-SSGD---LRGQRLSFKVTL--T-DSKTQTFLNVVPSSW--WFGQTFS--
SRGRQF

>AtEXPA13PRO

W------------RP-----ARATYYAATN--PRDAV----G-GACGY-GDLVKS-
GYGMATVGLSETLFERGQICGAC

FE-L--RCVD------D---LR-W--CIP-GT--SIILTATNFC---APNYGFDPD--
GGGHCNPPNKHFVLPIEAFEKI

AI-------WKA-GNMPVQY-RRINCRKEG-SMRFTV----DGGGI-FI---SVLITNVAGSGDIAAVKIK-GSR--
TG-

WL--PMGRNWGQNWH-INAD---LRNQALSFEVTS--S-DRSTVTSYNVSPKNW--NYGQTFE-G---KQF

>AtEXPA14PRO

W------------VN-----ARATFYGGAD--ASGTM----G-GACGY-GNLYSQ-
GYGTNTAALSTALFNGGQSCGAC

FQ-I--KCVD----------DPKW--CIG-G---TITVTGTNFC---PPNFAQANN--
AGGWCNPPQHHFDLAQPIFLRI

AQ-------YKA-GVVPVQY-RRVACRRKG-GIRFTI----NGHSY-FN---LVLITNVAGAGDVISVSIK-GTN--
TR-

WQ--SMSRNWGQNWQ-SNAK---LDGQALSFKVTT--S-DGRTVISNNATPRNW--SFGQTYT-G---KQF

>AtEXPA15PRO

W------------VN-----AHATFYGGSD--ASGTM----G-GACGY-GNLYSQ-
GYGTNTAALSTALFNNGLSCGAC

FE-I--KCQS------D---GA-W--CLP-G---AIIVTATNFC---PPNNALPNN--
AGGWCNPPLHHFDLSQPVFQRI

AQ-------YKA-GVVPVSY-RRVPCMRRG-GIRFTI----NGHSY-FN---LVLVTNVGGAGDVHSVAVK-GSR-
-TR-

WQ--QMSRNWGQNWQ-SNNL---LNGQALSFKVTA--S-DGRTVVSNNIAPASW--SFGQTFT-G---
RQF

>AtEXPA17PRO

W-------------LQ-----AHATFYGGSD--ASGTM----G-GACGY-GNLYTD-
GYKTNTAALSTALFNDGKSCGGC

YQ-I--LCDATKV-------PQ-W--CLK-GK--SITITATNFC---PPNFAQASD--
NGGWCNPPRPHFDMAQPAFLTI

AK-------YKA-GIVPILY-KKVGCRRSG-GMRFTI----NGRNY-FE---LVLISNVAGGGEISKVWIK-GSK--
SNK

WE--TMSRNWGANYQ-SNTY---LNGQSLSFKVQL--S-DGSIKAALNVVPSNW--RFGQSFK---SNVNF

>AtEXPA20PRO

W-------------KI-----ATATLSRDRD--GSSSVAT--G-GACGY-GDLRQS-
SFAGYSAGLSGKLFNRGSSCGAC

LE-V--RCVN------H---IR-W--CLQ-GSP-SVVVTATDFC---PPNSGLSSD--
YGGWCNFPKEHLELSHAAFTGI

AE-------TRA-EMIPIQY-RRVKCGRRG-GLRFSL----SGSSH-FF---QVLISNVGLDGEVVGVKVK-GHT--
TA-

WI--PMARNWGQNWH-SSLD---LIGQSLSFEVTL--K-GGKTIASYDVAPPYW--RFGMTYQ-G---KQF

>AtEXPA22PRO

W-------------YD-----ARATFYGDIH--GGDTQ-------PCGY-GNLFRQ-
GYGLATAALSTALFNDGYTCGAC

YE-I--MCTR----------DPQW--CLP-G---SVKITATNFC---PANYSKT----
TDLWCNPPQKHFDLSLAMFLKI

AK-------YKA-GVVPVRY-RRIPCSKTG-GVKFET----KGNPY-FL---MVLIYNVGGAGDIKYVQVK-GNK--
TG-

WI--TMKKNWGQNWT-TITV---LTGQGLSFRVTT--S-DGITKDFWNVMPKNW--GFGQTFD---GRINF

>OsEXPA4PRO

W-------------QS-----AHATFYGGGD--ASGTM----G-GACGY-GNLYSQ-
GYGTNTAALSTALFNDGAACGSC

YE-L--RCDN----------AGSS--CLP-G---SITVTATNFC---PPNYGLPSD--
DGGWCNPPRPHFDMAEPAFLHI

AQ-------YRA-GIVPVSF-RRVPCVKKG-GVRFTV----NGHSY-FN---LVLVTNVAGAGDVRSVSIK-GSR--
TG-

WQ--PMSRNWGQNWQ-SNAF---LDGQSLSFQVTA--S-DGRTVTSNNVAHPGW--QFGQTFE-G---
GQF

>OsEXPA5PRO

W------------SS-----AHATFYGGGD--ASGTM----G-GACGY-GNLYSQ-
GYGTNTAALSTALFNNGLSCGAC

FE-V--RCDA------GGGGSH-S--CLP-G---SVVVTATNFC---PPNNALPSD--
DGGWCNPPRAHFDMSQPVFQRI

AL-------FKA-GIVPVSY-RRVACQKKG-GIRFTI----NGHSY-FN---LVLVTNVGGAGDVHAVAVK-SER--
SAA

WQ--ALSRNWGQNWQ-SAAL---LDGQALSFRVTT--G-DGRSVVSNNAVPRGW--SFGQTFS-G---
AQF

>OsEXPA7PRO

W------------QS-----AHATFYGGSD--ASGTM----G-GACGY-GNLYSQ-
GYGVNNAALSTALFNSGQSCGAC

FE-I--KCVN--QP--G---WE-W--CHP-GSP-SILITATNFC---PPNYALPSD--
NGGWCNPPRPHFDLAMPMFLHI

AE-------YRA-GIVPVSY-RRVPCRKKG-GVRFTI----NGFRY-FN---LVLITNVAGAGDIVRASVK-GTS--
TG-

WM--PMSRNWGQNWQ-SNSV---LVGQALSFRVTG--S-DRRTSTSWNAAPAGW--HFGQTFE-G---
KNF

>OsEXPA10PRO

W------------RS-----AKASYYAA-D--PEDAI----G-GACGF-GDLGKH-
GYGMATVGLSTALFERGAACGGC

YE-V--KCVD------D---LK-Y--CLP-GT--SIVVTATNFC---APNFGLPAD--
AGGVCNPPNHHFLLPIQSFEKI

AL-------WKA-GVMPIQY-RRVNCLRDG-GVRFAV----AGRSF-FL---TVLISNVGGAGDVRSVKIK-GTE-
-SG-

WL--SMGRNWGQIWH-INSD---FRGQPLSFELTS--S-DGKTLTNYNVVPKEW--DFGKTYT-G---KQF

>OsEXPA11PRO

W------------SS-----GSATFYGGSD--ASGTM----G-GACGY-GNLYSA-
GYGTSTAALSTALFNNGQSCGAC

FE-V--RCGG----------GG-S--CLA-G---TVAVTATNLC---PPNYALAGD--
AGGWCNPPRPHFDMAEPAFTRI

AQ-------ARA-GVVPVQY-RRVACAKQG-GIRFTI----TGHSY-FN---LVLVTNVGGAGDVTAVSVK-GSR--SG-

WQ--AMSHNWGANWQ-NGAN---LDGQPLSFRVTA--S-DGRTVTSDNVAPSGW--SFGQTFS-G---GQF

>OsEXPA12PRO

A-------------LS-----GTATFYGGSD--ASGTM----G-GACGY-GNLYST-GYGTNTAALSSALFNDGAACGEC

YQ-I--TCDQ-SN-------SK-W--CKA-GT--SVTITATNLC---PPDYSKPSN--DGGWCNPPRQHFDMAQPAWEQI

GV-------YRG-GIVPVNF-QRVSCTRKG-GVRFTI----NGNSY-FE---LVLITNVGGPGSIKSVQIK-GTK--TG-

WV--TMSRNWGANWQ-ANNY---LNNQAISFSVTS--T-AGKTLVFEDVAPSNW--QFGQTFT---SGVQF

>OsEXPA16PRO

W-------------RM-----GSATYIKESL--GHPLNDG--G-GACGY-GDLDIF-RYGRYTAGVSGALFGRGSACGGC

YE-V--RCVN------H---VL-W--CLR-GSP-TVVVTATDFC---APNLGLSDD--YGGWCNFPKEHFEMSEAAFLRV

AK-------AKA-DIVPVQF-RRVSCDRAG-GMRFTI----TGGAS-FL---QVLITNVAADGEVAAVKVK-GSR--TG-

WI--PMGRNWGQNWQ-CDAD---LRGQPLSFEVTG--G-RGRTVVAYSVAPPDW--MFAQTFE-G---KQF

>OsEXPA30PRO

W-------------TP-----AHATFYGDET--ASETM----G-GACGY-GNLYAS-GYGTDTAALSTTLFKDGYGCGTC

YQ-M--RCVG----------TA-S--CYR-GSP-AITVTATNLC---PPNWAEDPDRGGGGWCNPPRAHFDLSKPAFMRM

AD-------WRA-GIVPVMY-RRVPCARAG-GLRFAL----QGNPY-WL---LAYVMNVAGAGDVGDMWVKAGGG--GG-

WV--RMSHNWGASYQ-AFAQ---LGGQALSFKVTS--YTTGQTILAAGVTPASW--CFGLTYQ---ARVNF

>OsEXPA32PRO

W-------------KQ-----AHATFYGGRD--GSGTL----D-GACGY-KDTSKE-GYGVQTVAVSTPLFGAGAGCGAC

YE-V--KCVD----------SPDG--CKV-GAA-PLVVTATNLC---PPNPGQSND--
NGGWCNPPREHFDLSMPAFLQI

AQ-------EKA-GIVPISY-RRVPCVKVG-GIRYTI----TGNPY-FN---LVMVSNVGGAGDVAGLSVK-GNK--
RVK

WT--PLKRNWGQEWQ-TSEV---LTGESLTFRVMT--G-DHRKATSWHVLPPDW--QFGVTYQ---ATKNF

>OsEXPA33PRO

W------------TP-----ATATFYGGSD--GAGTM----G-GACGY-GNLYNA-
GYGLNNAALSSALFNDGAMCGAC

YT-I--ACDT------S---QSTW--CKP-GT--SITITATNLC---PPNYAKKSD--
AGGWCNPPRKHFDMSQPAWTSI

AI-------YQA-GIVPVNF-KRVPCQKSG-GIRFTI----SGRDY-FE---LVTVFNVGGSGVVAQVSIK-GSK--
TD-

WM--AMSRNWGQNWQ-SNAY---LNTQSLSFKVKL--D-DAREVTVWNIAPSNW--NFGTTYT---
SNINF

>AaEXPB1PRO

W------------KP-----ATATWYGAPD--GAGTD----G-GACGY-GNLVGT-
PYGKRVTAGSPVLFKGGLGCGAC

YQ-V--KCMI----------PK-L--CAK-K---AVRVVITDEC--------------PGGYCSGLNTHFDLSGTAFSGM

AN-PGLGSSLLNLGVLPMLY-KRVPCVYRR-PMKIEVAA--GSSPF-WF---SVLIKFVRGAGELKSVELQ-
QGG--TNY

WY--SMQHIWGANWSVNVGS--GLKG-PFTLRLTC--LLTGQQIVIRNVIPGNW--APATTYL---ASVNF

>AaEXPB2PRO

W------------LS-----CKATFYGPQD--GDGTD----A-GNCGY-GALASS-
PYGKNIAAGSPELYQDGLGCGAC

FQ-I--KCTN-----------D-G--CKD-E---ASTVVITDQC---P----------GGEWCGVGKPHFDLRGTAFANL

AV-AGRSSDLFTRGVLDLQY-QRVACSYGAQTVAFAMDA--NCNPN-WI---
AFVVEFQAGDGDLHSVELK-DAS--SD-

WQ--AMSHNWGANWQ-FSGS---LTG-PISVRLTS--NTANQVLVAQDAIPANW--KPGQTYK---SQDQF

>AaEXPB3PRO

W------------LS-----CKATFYGPQD--GDGTD----A-GNCGY-GALASS-
PYGKNIAAGSPELYQDGLGCGAC

FQ-I--KCTN-----------D-G--CKD-E---ASTVVITDQC---P----------GGEWCGVGKPHFDLSGTAFANL

AV-AGRSSDLFTRGVLDLQF-QRVACSYGAQTVAFAMDA--NCNPN-WI---AFVVEFQAGDGDLHSVELK-DVS--SD-

WQ--AMSHNWGANWQ-FSGS---LTG-PISVRLTS--NTANQVLVAQDAIPADW--TPGQTYQ---SQDQF

>AaEXPB4PRO

W-------------LS-----CKATFYGPQD--GDGTD----A-GNCGY-GALASS-PYGKNIAAGSPELYQDGLGCGAC

FQ-I--KCTN-----------D-G--CKD-E---ASTVVITDQC---P----------GGEWCGVGKPHFDLSGTAFANL

AV-AGRSSDLFTRGVLDLQY-QRVACSYGAQTVAFAMDA--NCNPK-WI---AFVVEFQAGDGDLLSVELK-DAS--SD-

WQ--AMSHNWGANWQ-FSGS---LTG-PISVRLTS--NTANQVLIAQDAIPANW--KPGQTYK---SKQQF

>AaEXPB5PRO

W-------------LS-----CKATFYGPQD--GDGTD----A-GNCGY-GALASS-PYGKNIAAGSPELYQDGLGCGAC

FQ-I--KCTN----------DG----CKD-E---ASTVVITDQC---P----------GGEWCGVGKPHFDLSGTAFANL

AV-AGRSSDLFTRGILDLQY-QRVACSYGAQTVAFAMDA--NCNPN-WI---AFVVEFQAGDGDLHSVELK-DAS--SD-

WQ--AMSHNWGANWQ-FSGS---LTG-PISVRLTS--NTANQVLIAQDAIPANW--KPGQTYK---SQDQF

>AaEXPB6PRO

W-------------LA-----CKATWYGEQD--GDGSD----G-GACGY-GELSSK-PYGKNIAAGSPVLYQDGLGCGSC

YQ-V--KCTS----------DP-A--CSG-D---PVTVVLTDIC---P----------GGSLCGIGKPHFDLSGTAMGNM

AA-AGKGSDLRGLGVVDILY-KRVPCSYPEQNVTIAVDQ--GSNPS-WL---ELTVLYEGGPGDLSALEVK-DAT--SQ-

WQ--PMVHKWGANWGYDKAA--DLHG-ALSIRLTS--GLGSLPLVAQNALPADW--APGQTYQ---TVAQF

>AaEXPB7PRO

W-------------VA-----CKATWYGEPD--GDGSDVNA-G-GACGY-GELSSK-AYGKSIAAGSPVLYQDGLGCGSC

YQ-V--KCTS----------NP-A--CSG-D---PVTIVLTDIC---P----------GGSLCGVGKPHFDLSGTAIGNM

AA-AGKGSDLRGLGVVDILY-KRVPCAYPEQNVTIAVDQ--GSNPS-WL---ELTVLYEGGPGDLSALEVK-VTS--HS-

SN--PALACRGSQI---------VVC-VFAPELTTHAGLGT----AQDA-TSQWHRSGSRTLY---IIPSR

>AaEXPB8PRO

W-------------LS-----CKATFYGPRD--GDGTD----A-GNCGY-GALASS-PYGSNIFAGSPELYQDGLGCGDA

---S--RCTN-----------D-G--CND-T---PTTVVMTDQC---P----------GGEWCGIGKPHFDLSGTAFGSL

AA-AGRSSDLFTLGVLDIQY-QRVPCSYSTQTVAFAMDA--NCNPN-WI---AFVVEFQAGDGDLHSVELK-QDA--TSD

WQ--AMSHNWXANWQ-FSGS---LQG-PIPVRLTS--NTANQVLVAKDAIPADW--TPGQTYQ-------F

>CpEXPB1 [X]

W-------------MA-----GHATWYGEPN--GEGSS----G-GACGY-TKLTGT-PYGPRIAAGNDAIFQGGKGCGQC

YE-V--KCSY----------AE----CRQ-E---ATRIVITDQC---P----------GGEYCSKAEPAFDLSGAAISAM

AL-PDKDGALRNIGLYDILY-KRVPCEFQGQNIAFQVDQ--GSTGF-WL---SFVVKFLGGPGDIDSVEIR-HSG--SSS

FQ--PAKHNWGASWMLINTSGQAFKG-PYDIRVVS--KLNGHAVTAEKVIPEFF--EPGKLYE---SNVQL

>CpEXPB2 [A]

W-------------VF-----GHGTWYGDPY--GEGSS----G-GNCGY-TKLAGT-PYGPKIVAGSPAIYANGLGCGQC

YQ-V--KCTDSKNG------PR-L--CNP-S---GTQVVVTDFC---P----------GGTYCSTGEKAFDFSGAALNAM

AL-PGKDGQLRNRGLYNVLY-KRVPCKYKGQNIAFRVDP--GSSPY-WL---SVLIKYVGGPGDIGAVSIK-MAN--SYK

FM--PMKHAWGANWMLQNYDGKPFKG-PMDIKIVS--RLNGHSVVAKGVIPGYF--RPGTTYT-SRVQMAY

>CpEXPB3 [A]

W-------------VY-----GHGTWYGDPY--GEGSS----G-GNCGY-TKLAGT-PYGPKIVAGSPAIYANGLGCGQC

YQ-I--KCLDSKYG------PR-L--CNS-W---GTQVVVTDFC---P----------GGTYCSSGQKAFDFSGAALNAM

AL-PGKDGQLRNRGLYNVLY-KRVPCKYKGQNIAFRVDP--GSSPY-WL---SVLIKYVGGPGDIGAVYIK-MAN--SYR

FM--PMKQAWGANWMLQNYDGKPFKG-PMDIKIVS--RLNGHSVVAKGVIPGYF--RPGTTYT-SRVQMAY

>CpEXPB4 [X]

W-------------MA-----GHATWYGEPN--GEGSS----G-GACGY-TKLTGT-
PYGPRIAAGNDAIFQGGKGCGQC

YE-V--KCSY----------AE----CRQ-E---ATRIVITDQC---P----------GGEYCSKAEPAFDLSGAAISAM

AL-TDKDGALRNIGLYDILY-KRVPCEFQGQNIAFQVDQ--GSTGF-WL---SFVVKFLGGPGDIDSVEIR-
HSG--SSS

FQ--PAKHNWGASWMLINTSGQAFKG-PYDIRVVS--KLNGHAVTAEKVIPEFF--EPGKLYE---SNVQL

>CpEXPB5 [A]

W-------------MA-----GHATWYGDPY--GEGSS----G-GACGY-TQLTGT-
PIGSKIAAGNNPIFQGGKGCGQC

YE-V--KCNY-----------P-S--CNP-E---PTRIVITDLC---P----------GGTYCSTGNPAFDFSGAAITAM

AL-PGRDGELKKIGLYDIQY-KRVPCEYQNQNIAFKVDF--GASAY-WL---
SFTVKYLGGPGDIESVEIRQVGS--NGS

FQ--PCQHSWGANWMLINYTGQPFDG-PYDVKITA--KLNGHSVIAYRAIPKWF--QPGALYN---SNVQF

>CpEXPB6 [ACnFn]

W-------------IA-----GHATWYGDPY--GEGSN----G-GACGY-TELAGT-
PYGLSVAAGSAIIFQNGQGCGQC

YD-V--KCSY-----------P-S--CKQ-E---PTRVVITDSC---P----------GGTYCSTGEPAFDLSGMAMSNM

AL-PGRDQELRNLGLYDIQY-KRVPCYYPNQNIAFKVDP--GSTPY-WL---SFTVKYQGGPGDIQNVEIR-
QAG--SSE

FQ--PLQHNWGANYMMICYSGQPFRG-PYDVKITA--KLNGHVLIAEHAIPEYF--QPGGLYE---SNVQL

>PpEXPB1PRO

W-------------LG-----GHATWYGDPY--GEGSS----G-GACGY-TQLTGT-
PIGSKIAAGNAPIFQEGKGCGQC

YE-V--KCNY----------PS----CSP-Q---GTRIVITDLC---P----------GGQYCSTDQPAFDFSGAAITAM

AL-PGRDGELRNIGLYDIQY-KRVPCEYPNQNIAFKVDA--GSSKY-WF---SFTVKYLGGPGDINTVEVK-
CGK--NGY

FQ--YAQHSWGANWMLINYSGVPFQF-PLTIKITT--KLNDHTVVAEDVIPDWF--GPGVQYE---SNVQI

>PpEXPB2PRO

W-------------VR-----ARGTWYGDPY--GEGSS----G-GNCGY-TKLWGT-
PIGPKIVAGSRSIYANGQGCGQC

YQ-I--RCVDPNGG------PR-L--CNP-Q---GTNVVVTDFC---P----------
GGTYCSTGENAFDMSGAAINAM

AL-RGREGQLRNRGLYNLLY-KRVPCRYRGTNIEFRVDN--GSSPF-WL---SILIKYVGGPGDIGQVYIR-MAN--WYK

FQ--PMRHAWGANWMIPNYDGKPFRG-PMDIRIVS--RLNRHTVLARGVIPAYF--RPGTSYR-SRVQMAF

>PpEXPB3PRO

W------------LP-----GHATWYGDPY--GEGSS----G-GACGY-TELAGT-PYGLSVGAGSAVIYQNGQGCGEC

YE-V--KCTY----------PS----CKP-T---PSRIVITDFC---P----------GGTFCSTGEPAFDLSGMAMTNM

AL-PGRDQELRNLGLYEIQY-RRVPCYYPNQNVAFKVDP--GSTPF-WL---SFTIEYQGGPGDIESVAIR-QAG--SSE

FQ--QMQHNWGANYMLICYSGKPFKG-PYDVMITA--KLNGHVLIAQDAIPEYF--QPGGIYE---SNVQL

>PpEXPB4PRO

W------------VP-----GHATWYGDPY--GEGSS----G-GXCGY-LQLTGT-PIGNKIAAGNEAIFQKGKGXXQC

YE-V--KCNY-----------P-V--CRP-E---GTRIVITDLC---P----------GXQFCSGGNPAFDLSGAAISAM

AK-DGQDGALRNIGLYDIQY-KRVPCEYPGQNIVFKVDA--GSSPF-WL---SFTVKYMGGPGDIESVSI--SQR--DGS

FI--PAQHSWGANWMLINYSGAPFQG-PYSVKINC--MLNGHTVVAKDVIPAGF--APGQEYE---SNVQI

>PpEXPB5PRO

W------------MA-----GHATWYGEPL--GEGSS----G-GACGY-TKLADT-PYGPKIAAGNDPIFQGGSGCGAC

FE-V--KCNY----------PS----CKS-E---PTRIIITDQC---P----------GGTYCSTSQPAFDLSGAAISDM

AV-SGKDGELRNIGLYDILY-KRVPCEYPNQNIAFQVDA--GSSAF-WL---SLLVKYMGGPGDIESVEIR-TTG--SSS

FQ--PAKHNWGASWMLINTSGQPFKG-PYDVKIVS--KLNGHTVIAEKAIPEFF--EPGKLYE---SNVQM

>PpEXPB6PRO

W------------VR-----ARGTWYGDPY--GEGSS----G-GNCGY-TRLXGT-PIGPKIVAGSRSIYXNGLGCGQC

YQ-N--QCVD------P---XX-G--XKL-XNSFXTQXXXTDFC---P----------GGXYCSTGENAFDMSGAAINAM

AL-PSREGQLRNRGLYNLLY-KRVPCRYRGTXIXXRVDX--XSSAY-WL---SILIKYVGGPGDIGQVYIK-MAN--WFA

FQ--PMKHAWGANWMMPSYDGKPFKG-PMDIKIVS--KLNRHSVVARGVIPGYF--RPGTTYN---
SRVQM

>SmEXPB1PRO

W-------------EP-----ATATWYGSPN--GAGTD----G-GACGY-GSLPNT-
PYGSDVGAGSPILFMNGIGCGTC

FEVIYVKCVD----------GQ-L--CSP-Q---PVNVVITDEC--------------PGGYCSGGRTHFDLSGTAFGKM

ASGSANIQHLLAAGVLNVLY-RRAPCIYKSQGVVFQVAD--GSTPF-WF---ETVIRYLDGPGDXATVELQ-
QFG--SSA

WQ--PMSQVWGANWCLNAGGGTPLRA-PFSIRLTA--LQTGEKXIAHNVIPANW--APQHSYS---
TGVNF

>SmEXPB2PRO

W-------------QL-----ATATWYGSPS--GAGTD----G-GACGY-GELPNT-
PYGLDVGAGSPVLFKNGRGCGAC

YKVV--RCLQ----------QQ-L--CSG-K---AVTVVITDEC--------------PGGYCAFGRTHFDLSGTAFGRM

AM-AGRTNQLLGSGVXQVLY-KRXDCNYGSRPMEFQVNE--GSTPF-WL---
SILVRYAAGPGDLGHVELMQGSR----V

WQ--PMTQVWGATWCFNGGP---LRG-PFSFRVTT--LSTSETVVARNVIPXNW--ASNTCYR---SRVNF

>OsEXPB15PRO

W-------------SN-----GGATWYGAAN--GAGSD----G-GACGYQGAVFQA-
PFSSMIAAGSPSIYKSGLGCGSC

YQ-V--KCTG----------NS-A--CSG-N---PVTVVLTDEC--------------PGGPCLSEPVHFDLSGTAFGAM

AN-PGQADQLRAAGVLQIQY-NRVPCNWGGVKLTFVVDV--GSNPN-YF---
AVLVKYENGDGDLSGVELMQTGA--GAA

WT--QMQQSWGAVWKLNAGS---ALQAPFSIRLTS--S-SGKTLVASNVIPSGW--KPGMSYI---STVNF

>OsEXPB16PRO

W-------------HP-----ATATWYGSAD--GDGSD----G-GACGY-
GTLVDVVPMKTRVGAVSPVLFKGGEGCGAC

YK-V--RCLD----------AS-I--CSR-R---AVTVIVTDEC--------------PGGVCAFGRTHFDLSGAAFARL

AV-AGHGGQLQNRGEISVVY-RRTACKYGGKNIAFHVNE--GSTTF-WL---
SLLVEFEDGDGDIGSMQLK-QAN--SAQ

WQ--DMKHIWGATWSLTPGP---LVG-PFSVRLTT--LTTRQTLSAQDVIPKNW--TPKATYT---SRLNF

>OsEXPB17PRO

W-------------RP-----ATATWYGDAE--GDGST----G-GACGY-GSLVDVVPMKARVGSVSPVLFKDGEGCGAC

YK-V--KCLD----------HG-I--CSR-R---AVTVIVTDEC--------------PGGLCAFGRTHFDLSGAAFSRM

AV-AGAGGHLRDRGQLSVVY-RRTACKYGGKNIAFRVNE--GSTNF-WL---SLLVEFEDGQGDIGSMQIK-QAN--SVE

WL--DMKHVWGATWCLVRGP---LVG-PFSVRLTT--LSAQKALTARDVIPRNW--KPTATYT---SRLNF

>OsEXPB18PRO

W-------------SN-----GGATWYGAAN--GAGSD----G-GACGY-QAAVDQAPFSSMIAAGSPSIYKSGLGCGSC

YQ-V--KCSG----------NS-A--CSG-N---PVTVVLTDEC--------------PGGPCLSEPVHFDLSGTAFGAM

AN-PGQADQLRAAGVLQIQY-NRVPCNWGGVMLTFAVDA--GSNPS-YF---AVLVKYENGDGDLSGMDLMQTGA--GAA

WT--PMQQSWGAVWKLSAGA---ALQAPLSIRLTS--S-SGKTLVASNVIPSGW--KPGASYT---STVNY

>AtEXPB1PRO

W-------------LP-----ATATWYGSAE--GDGSS----G-GACGY-GSLVDVKPFKARVGAVSPILFKGGEGCGAC

YK-V--RCLD----------KT-I--CSK-R---AVTIIATDQS---P----------SGPSAKAKHTHFDLSGAAFGHM

AI-PGHNGVIRNRGLLNILY-RRTACKYRGKNIAFHVNA--GSTDY-WL---SLLIEYEDGEGDIGSMHIR-QAG--SKE

WI--SMKHIWGANWCIVEGP---LKG-PFSVKLTT--LSNNKTLSATDVIPSNW--VPKATYT---SRLNF

>AtEXPB2PRO

W-------------SI-----AGSTWYGNPT--GYGSD----G-GACGY-GNAVAQPPFSKMVSAGGPSLFKSGKGCGAC

YQ-V--KCTS----------KS-A--CSK-N---PVTVVITDEC---PG-------------CVKESVHFDLSGTAFGAM

AI-SGQDSQLRNVGELQILY-KKVECNYIGKTVTFQVDK--GSNAN-SF---AVLVAYVNGDGEIGRIELKQALD--SDK

WL--SMSQSWGAVWKLDVSS--PLRA-PLSLRVTS--LESGKTVVASNVIPANW--QPGAIYK---SNVNF

>AtEXPB3PRO

W-------------LP-----AVATWYGSPN--GDGSD----G-GACGY-GTLVDVKPLHARVGAVNPILFKNGEGCGAC

YK-V--RCLD----------KS-I--CSR-R---AVTVIITDEC---PG-------------CSKTSTHFDLSGAVFGRL

AI-AGESGPLRNRGLIPVIY-RRTACKYRGKNIAFHVNE--GSTDF-WL---SLLVEFEDGEGDIGSMHIR-
QAG--ARE

WL--EMKHVWGANWCIIGGP---LKG-PFSIKLTT--LSAGKTLSATDVVPRNW--APKATYS---SRLNF

>AtEXPB4PRO

FCYCQNETIDVAGSGT-----AGVTWYGEPF--GAGST----G-GACGY-
GSAVANPPLYAMVSAGGPSLFNNGKGCGTC

YQ-V--VCIG----------HP-A--CSG-S---PITVTITDEC--------------PGGPCASEPVHIDLSGKAMGAL

AK-PGQADQLRSAGVIRVNY-KRAACLYRGTNIVFRMDA--GANPY-YI---SFVVEYENGDGDLSNVEIQ-
PAG--GS-

FI--SMQEMRSAVWKVNSGS--ALRG-PFNIRLTS--GESHKVIVAYNVIPANW--KPDESYR---SIVNF
